# Untargeted Metabolomic Analysis Combined with Chemometrics Revealed the Effects of Different Cooking Methods on *Lentinus edodes*

**DOI:** 10.3390/molecules28166009

**Published:** 2023-08-11

**Authors:** Jinrui Zhu, Li Zhou, Jiaxu Yao, Yueqi Hu, Zhenghui Li, Jikai Liu, Eric Marchioni

**Affiliations:** 1 National Demonstration Center for Experimental Ethnopharmacology Education, School of Pharmaceutical Sciences, South-Central Minzu University, Wuhan 430074, China; zjrui1998@126.com (J.Z.); 1214yjxu@sina.com (J.Y.); a2446860889@outlook.com (Y.H.); 2 Equipe de Chimie Analytique des Molécules Bioactives et Pharmacognoise, Institut Pluridisciplinaire Hubert Curien (UMR 7178, CNRS/UDS), 74 Route du Rhin, 67400 Illkirch, France; eric.marchioni@unistra.fr

**Keywords:** cooking methods, untargeted metabolomics, chemometrics, *Lentinus edodes*, Shiitake

## Abstract

Cooking methods affect the compositions of *Lentinus edodes* metabolites. Nevertheless, little information is available on the specific impact of different cooking methods on *Lentinus edodes* via metabolomic analysis. This study determined the influence of boiling, steaming, air-frying, and roasting on the metabolomic profiles of *Lentinus edodes* based on UHPLC-Q-Exactive Orbitrap MS/MS in combination with chemometrics. A total of 990 metabolites were detected and classified into 11 super-classes. Subsequently, the metabolites of the four cooking methods were distinguished using multivariate statistical analysis. The results showed that boiling caused a massive loss of metabolites while roasting and air-frying led to an evident upregulation. The upregulation of metabolites in the steaming groups was not as significant as in roasting and air-frying. This study provided reference data for a comprehensive understanding of the metabolites associated with domestic cooking methods and valuable guidance for the development of *Lentinus edodes* and its products in the future.

## 1. Introduction

Edible fungus is one of the most popular ingredients in the world. Their high protein, high dietary fiber, and unique flavor make them a delicacy and they are widely incorporated into a variety of dishes, especially in East Asia [1]. In recent decades, many chemical components have been extracted from different parts of edible fungus, and previous studies have confirmed that these extracts exhibit health-promoting benefits, especially in diseases associated with inflammation [2], including antitumor activities [3], antioxidant activities [4], antimicrobial and antibiofilm effects [5], and antihypertensive and cholesterol-lowering effects [6].

Previous research reported that China has about 967 species of edible fungi, accounting for nearly 50% of the worldwide population. Meanwhile, the production of edible fungi in China ranks first in the world. Among those edible fungi, *Lentinus edodes* (Shiitake) is the most consumed, and production exceeded 10 million tons in 2020 [7]. *Lentinus edodes* is always used as a “functional food”, as it contains many health-promoting compounds such as polysaccharides, dietary fiber, essential amino acids, and minerals [8]. Many studies have been conducted on *Lentinus edodes* in recent years, particularly regarding their nutritional and health benefits, ranging from improving health to preventing disease [9].

Generally, *Lentinus edodes* is always consumed in the food industry as a food or food-flavoring material in soups, sauces, salads, and meat dishes. Consumption of raw or undercooked mushrooms may lead to skin eruptions [10]. Therefore, *Lentinus edodes* is commonly cooked before consumption. Additionally, cooking can improve the taste and flavor of food and promote human digestion and absorption [11]. In the food industry and households, the most common and important food processing methods are thermal processing methods, causing Strecker degradation and the Maillard reaction which produce desirable flavor generation as well as nutritional changes.

The influence of thermal processing on different species of edible fungi has been studied, especially on the umami, aroma, bioactive compounds, and nutritional composition [12,13,14,15]. These results indicated that the chemical composition and biological activities varied according to the cooking methods, with each cooking method having a specific influence on food. In addition, some researchers have already investigated the influence of distinct cooking methods on *Lentinus edodes*. These studies concluded that it was hard to maintain all nutrients at their highest levels with any cooking method [16]. The changes in the nutritional content of *Lentinus edodes* caused by cooking were dependent on the type of cooking method [17]. Nevertheless, many reported studies focused on the specific compounds of *Lentinus edodes* and less information is available on the large-scale characterization of substances with distinct cooking methods.

The development of omics technology provided a new perspective. The wide application range and high sensitivity of omics technology have been favored by researchers. Over the past few decades, significant breakthroughs have been made in edible fungus studies with the application of omics technology, which has been flexibly combined with other analytical methods [18]. Rapidly evolving metabolomics have made it possible to characterize hundreds or thousands of metabolites in complex samples at high resolution in a single measurement. It has enabled the comprehensive and unbiased analysis of metabolites and has broadly been used in the research of edible fungi [19,20,21,22]. However, to our knowledge, few studies have been conducted on the influence of different cooking methods by using metabolomics assays on *Lentinus edodes*.

Considering the interest in human consumption of *Lentinus edodes* and the lack of large-scale information on related studies, untargeted metabolomics analysis was employed to reveal the change of metabolites in *Lentinus edodes* boiling, steaming, air-frying, and roasting. The aim of this research was to establish a multivariate approach to elucidate the effects of different cooking methods by characterizing the metabolome of *Lentinus edodes*. For this purpose, metabolomics was combined with chemometrics to provide reference data for comprehensively understanding the impact of various cooking methods on *Lentinus edodes* and to provide valuable guidance for the further processing of *Lentinus edodes* and its products.

## 2. Results and Discussions

### 2.1. Overview of the Metabolites in Lentinus edodes with Different Cooking Methods

**Figure 1:**
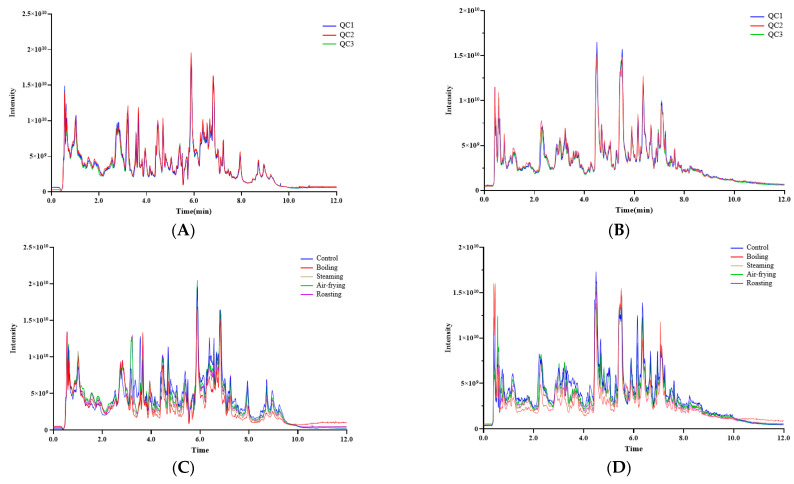
Total ion chromatography (TIC) of QC samples and the metabolites in *Lentinus edodes* with different cooking methods. (**A**,**C**) in ESI (+) mode, (**B**,**D**) in ESI (−) mode.

The metabolites of *Lentinus edodes* with distinct cooking methods (steaming, boiling, air-frying, and roasting) were compared and raw *Lentinus edodes* was used as a “control” sample. Simultaneously, Quality Control (QC) samples were prepared and used to monitor the reproducibility and stability of the system. The high overlap of total ion chromatography (TIC) for different QC samples (Figure 1A,B) and the agreement of retention time and peak intensity indicated that the mass spectrometer has high signal stability, which confirmed the stability and repeatability of the data. Moreover, coefficient of variation (CV) values for metabolites in the QC samples were calculated, and the results were under 20% (Figure S1). The results of these QC samples all confirmed the reliability of the data.

The TICs of metabolites of *Lentinus edodes* with different cooking methods are displayed in Figure 1C,D. A total of 990 metabolites were detected and 651 and 339 metabolites were acquired in both ESI (+) and ESI (−) modes, respectively (Table S1). These metabolites could be grouped into 11 super-classes (Figure S2), and the number of metabolites was relatively higher in the categories of organic acids and derivatives, lipids and lipid-like molecules, and organo-heterocyclic compounds, while the lowest amounts of metabolites were found in alkaloids and derivatives and organosulfur compounds.

### 2.2. Heatmap for All Identified Metabolites in Lentinus edodes

**Figure 2:**
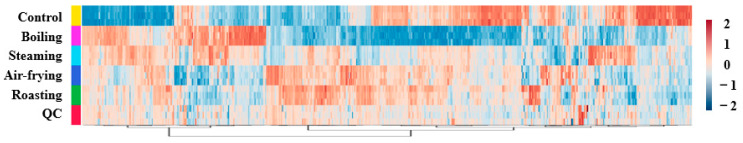
Heatmap visualization of all metabolites. The redder the color, the higher the level of metabolites.

A heatmap was employed to better analyze the relative expression abundances of *Lentinus edodes* metabolites under different cooking methods (Figure 2). The difference between the boiling groups and the control groups was obvious, with most metabolites downregulated, suggesting that boiling caused the loss of many metabolites present in *Lentinus edodes*. In the groups processed by steaming, the relative abundance of numerous substances differed from the control groups and they can be readily distinguished. Meanwhile, many upregulated metabolites were observed in the air-frying and roasting groups, illustrating that the two groups likely displayed a better retention rate for metabolites. Taken together, the results of the heatmap analysis indicated that cooking methods changed the metabolite profile of *Lentinus edodes*.

### 2.3. Multivariate Statistical Analysis of the Metabolites

In this study, the metabolites of *Lentinus edodes* under different cooking methods were analyzed using principal component analysis (PCA)-based unsupervised and orthogonal partial least squares discriminant analysis (OPLS-DA)-based supervised chemometric methods.

**Figure 3:**
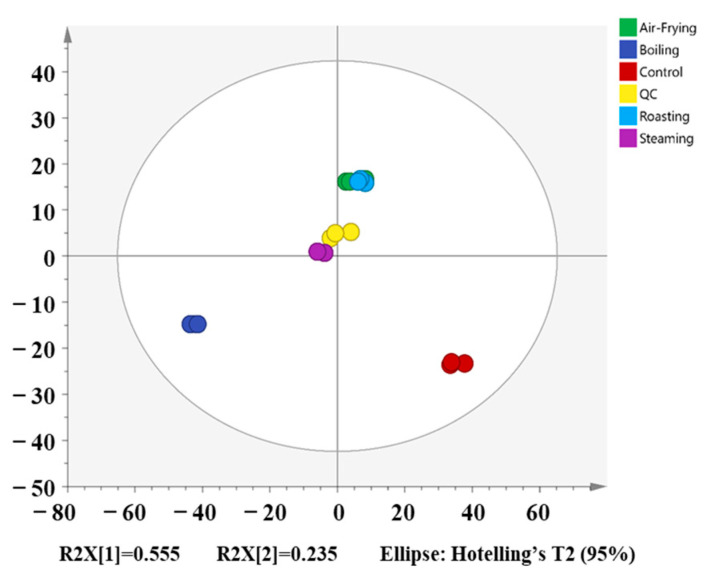
Principal component analysis (PCA) score scatter.

First, the overall variation of the metabolites in different samples and the degree of variation within groups were studied by PCA (Figure 3, PC1 = 55.5%, PC2 =23.5%). The QC samples were closely distributed near the origin coordinates, confirming the good stability of the whole process and the high reliability and repeatability of the obtained data. As demonstrated in the PCA plot, the samples of *Lentinus edodes* with different cooking methods were far from the control samples, suggesting that the process of cooking caused a large difference in the metabolites of *Lentinus edodes*. The air-fried samples were distributed closer to the roasted samples, and they were far from the boiling and steaming samples, illustrating that the metabolites in the air-frying groups had similar characteristics to the metabolites in the roasting groups. In addition, they had large differences from the metabolites in the boiling and steaming groups. These results suggest that differences in cooking methods could make a difference in metabolite composition.

**Figure 4:**
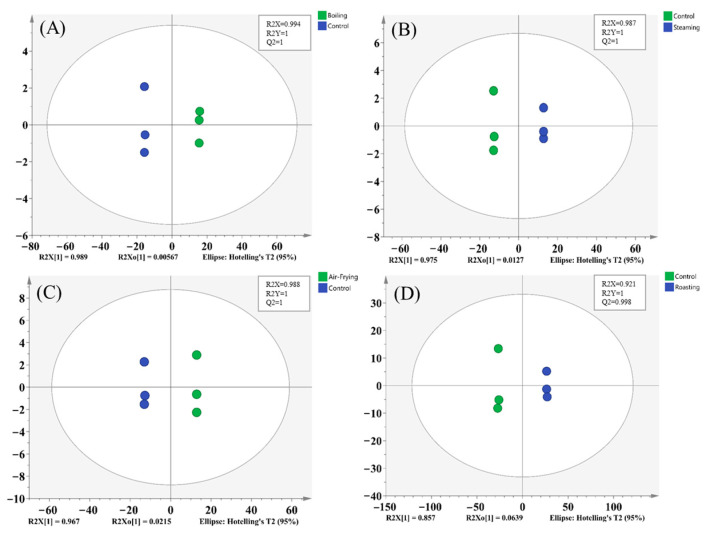
The score plots of OPLS-DA models. (**A**) Boiling vs. Control; (**B**) Steaming vs. Control; (**C**) Air-frying vs. Control; (**D**) Roasting vs. Control.

Subsequently, in order to overcome the limitations of PCA and to better distinguish these groups, we further constructed the supervised OPLS-DA model [23]. In this model, R2X(cum) and R2Y(cum) indicated the explanatory rate of the X and Y matrices, respectively. Q2(cum) denoted the prediction ability. The results of pairwise comparisons of raw and cooked *Lentinus edodes* are displayed in Figure 4. The result showed that the R2Y(cum) and Q2(cum) values of all the models were higher than 0.9, suggesting a high level of confidence [24]. Moreover, the results of the 200 permutations test (Figure S3) also exhibited good predictability for the OPLS-DA models (Q2 < 0.05, R2 values < original values) [25]. The result of OPLS-DA showed good separation among the four pairwise comparisons, implying remarkable differences between raw and cooked *Lentinus edodes* metabolites.

The variable importance in projection (VIP) value obtained from the OPLS-DA model was applied to further screen significantly different metabolites in *Lentinus edodes* with distinct cooking methods. In general, the variables with a VIP > 1 could be considered significant contributors to group discrimination, and a higher VIP score indicated better discrimination.

### 2.4. Screening of Significantly Different Metabolites in Lentinus edodes with Distinct Cooking Methods

Based on the combination of *p*-value (<0.05), the log_2_FC (≥1 or ≤−1), and VIP value (>1), significantly different metabolites were screened between the raw and four cooking groups. The results are displayed in Tables S2–S6. The amounts of downregulated metabolites and upregulated metabolites varied with the cooking method (Table S2).

**Figure 5:**
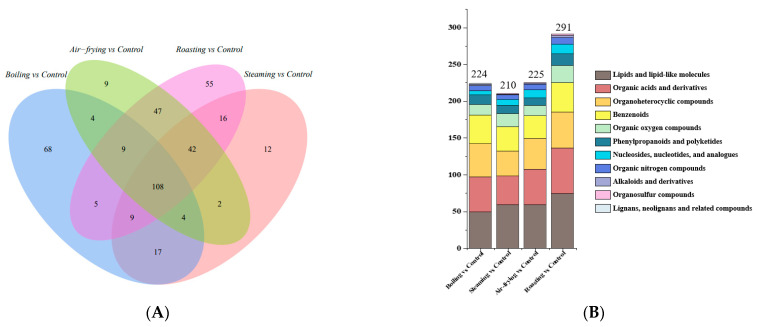
Analysis of the differential metabolites in *Lentinus edodes* treated by different cooking methods. (**A**) Venn diagram of the differential metabolites of pairwise comparisons. (**B**) Differential metabolite classification statistics.

A Venn diagram was established according to the screened different metabolites (Figure 5A). Common and unique metabolites among the four pairwise comparison groups were revealed. A total of 108 different metabolites were observed in raw and cooked *Lentinus edodes*. Furthermore, differentially expressed metabolites were grouped into 11 super-classes, including lipids and lipid-like molecules, organic acids and derivatives, organo-heterocyclic compounds, benzenoids, organic oxygen compounds, phenylpropanoids and polyketides, nucleosides, nucleotides and analogues, organic nitrogen compounds, alkaloids and derivatives, organosulfur compounds, lignans, and neo-lignans and related compounds (Figure 5B).

**Figure 6:**
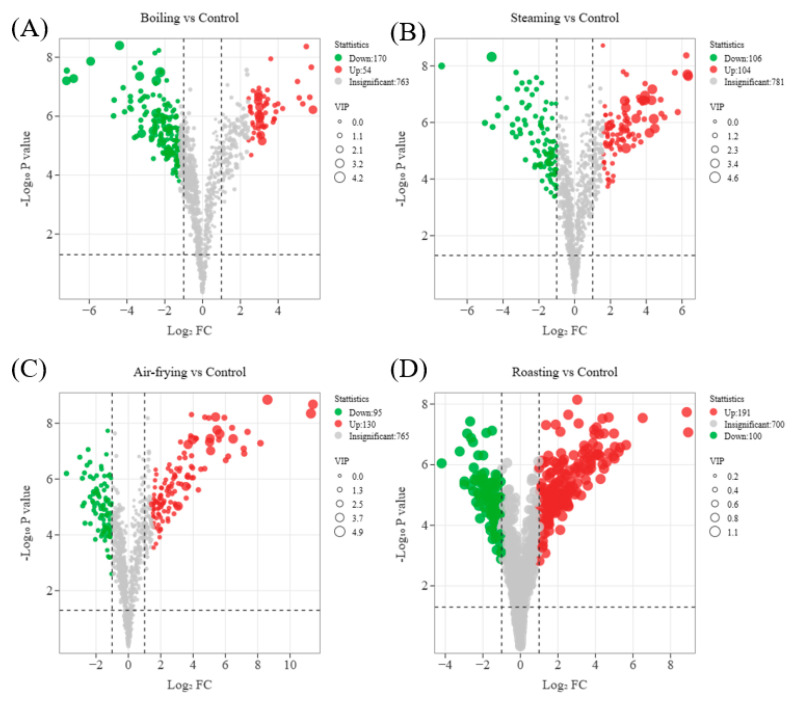
Volcano plot of the differential metabolites of (**A**) Boiling vs. Control; (**B**) Steaming vs. Control; (**C**) Air-frying vs. Control; (**D**) Roasting vs. Control.

The volcano plots obtained by pairwise comparisons of the differentially expressed metabolites are shown in Figure 6. As displayed in the plots, the following differential metabolites were identified: 224 (54 upregulated and 170 downregulated) in Boiling vs. Control, 210 (104 upregulated and 106 downregulated) in Steaming vs. Control, 225 (130 upregulated and 95 downregulated) in Air-frying vs. Control, and 291 (191 upregulated and 100 downregulated) in Roasting vs. Control were significantly regulated. The number of upregulated metabolites was significantly lower than downregulated metabolites in the comparison of boiling groups and control groups. On the contrary, roasting and air-frying caused more upregulated metabolites. Meanwhile, the changes in the metabolites were more distinct in roasted *Lentinus edodes* samples compared to other methods. The differences showed the large variety and complexity of the metabolites in *Lentinus edodes* when subjected to different cooking methods.

Further investigation for the relative express abundance of characteristic differential metabolites in *Lentinus edodes* under different cooking methods was conducted by heatmap analysis (Figure S4). It was clear that four pairwise comparisons of differential metabolites were evidently clustered into two color sections, implying that metabolites in each pairwise comparison were substantially different. These results reconfirmed that cooking-induced change in metabolite composition is dependent on the type of cooking method.

### 2.5. Changes in Different Metabolites in Lentinus edodes with Distinct Cooking Methods

As described in many previous studies, cooking will cause a series of reactions, such as the Maillard reaction, Strecker degradation, and other nutrient degradation processes. These reactions will consume reducing sugars, amino acids, proteins, carbohydrates, and polyphenols [26]. The screened different metabolites in *Lentinus edodes* with distinct cooking methods mainly focused on organo-heterocyclic compounds, organic acids and derivatives, lipids, and lipid-like molecules. The great importance of these compounds in *Lentinus edodes* has been confirmed by previous studies [27].

Although lipids are not the major component of mushrooms, they were a substantial part of the metabolite composition in many cases [28]. Lipids have an evident influence on food taste and appearance in addition to providing essential nutrients and metabolic energy [29]. After cooking, the most significant changes were found in lipids and lipid-like molecules, indicating their instability during thermal processing. More specifically, among the four cooking methods, boiling caused the most significant downregulation in *Lentinus edodes*, which might be related to the occurrence of hydrolysis during thermal processing, causing the denaturation and breakdown of lipids and their migration into boiling water [30]. Although both boiling and steaming groups used water as the cooking medium, *Lentinus edodes* was not in direct contact with water in the steaming groups compared to the boiling groups. In addition, the loss of metabolites in the steaming groups was not very severe [31].

In a conventional oven, food is cooked by a heat source that has no internal fan or other forced airflow device. Unlike this, the air fryer primarily uses the circulation of hot air to cook the food. Compared to conventional ovens, the hot air in an air fryer comes into contact with more food materials and has a higher heating efficiency [32]. Contrary to boiling, the upregulation of lipids and lipid-like molecules in roasted samples was greater than the downregulation compared with raw samples. This might be because of the breakdown of cell structures caused by heating, which released more lipid substances [33]. While in air-frying groups, the upregulation was less than in roasting groups, suggesting higher efficiency in the heat supply of the air-fryer led to a faster rate of lipid degradation [34].

As the results show, the changes of lipids and lipid-like molecules in *Lentinus edodes* treated with different cooking methods were obvious, but the obtained information was likely to be incomprehensive due to the limitations of the extraction method. Further lipidomic analysis is required to discuss the influence of different cooking methods on the lipid profile of *Lentinus edodes* in the future.

Numerous studies have confirmed that nucleotides and amino acids are the main contributors to the taste of edible fungi, especially umami taste [1,35,36]. The process of cooking might lead to the structural destruction of proteins, which were degraded into primary structures, secondary structures, or into peptides [16], which can be further hydrolyzed into amino acids [37]. Due to these changes, significant upregulation of many organic acids and derivatives (mainly including amino acids, peptides, and analogues) was observed during air-frying and roasting. Furthermore, some metabolites of nucleosides, nucleotides, and analogues were also upregulated. Taken together, the enhancement of the mushroom umami after cooking might be due to the upregulation of these metabolites. The amount of upregulated metabolites in the steaming and boiling groups was less than in the air-frying and roasting groups, and even greater downregulation was observed in the boiled samples. This is probably because these metabolites leached out of the samples and dissolved in the water during the cooking process.

Meanwhile, the Maillard reaction that occurs during cooking played a vital role in the generation of food color, aroma, flavor, and nutritional value [38]. The main products of the Maillard reaction are the oxygen-containing heterocyclic compounds, nitrogen-containing heterocyclic compounds, and volatile sulfur-containing compounds [39]. Therefore, these corresponding metabolites accounted for a large proportion of differentially expressed metabolites screened between the raw and the boiling, steaming, air-frying, and roasting groups. Compared to the air-frying and roasting groups, boiling and steaming caused lower levels of upregulated metabolites, suggesting a continuous loss of metabolites into the cooking medium. This has been proved by previous studies [40,41,42].

In summary, many reactions invoked by cooking processes, such as lipid denaturation and breakdown, protein denaturation and hydrolysis, amino acid alteration and destruction, and Maillard reactions, resulted in differences in metabolite expression [43]. Many downregulated substances were observed in the boiling groups, indicating that boiling is likely to induce the continuous loss of metabolites. Additionally, more upregulated metabolites were screened between the raw and roasting groups, suggesting that roasting may increase the concentration and display better retention of metabolites. The higher efficiency in the heat supply of air-fryers might depress the upregulation of metabolites compared to roasted samples. The process of steaming used water as a cooking medium just like boiling, but the loss of metabolites was not as severe. In addition, the upregulation of metabolites was not as significant as roasting and air-frying, and this is attributed to the mild temperatures of steaming.

### 2.6. Pathway and Enrichment Analysis Based on KEGG Annotation

**Figure 7:**
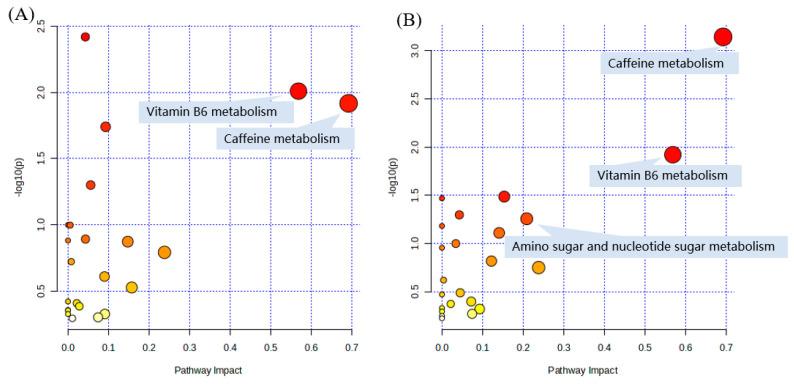

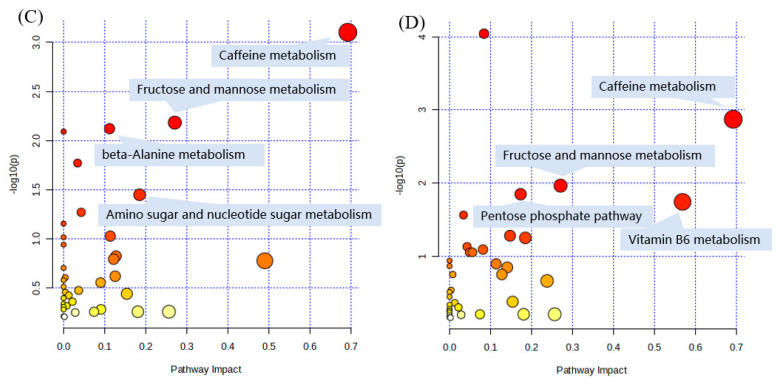
Pathway analysis of different metabolites of each pairwise comparison of *Lentinus edodes*. (**A**) Boiling vs. Control; (**B**) Steaming vs. Control; (**C**) Air-frying vs. Control; (**D**) Roasting vs. Control.

To further analyze the impact of different cooking methods on *Lentinus edodes*, these screened, differentially expressed metabolites were subjected to metabolic pathway analysis and a bubble chart was obtained (Figure 7). Each bubble represents a metabolic pathway [44]. The results showed that these different metabolites mainly mapped to metabolic pathways, including vitamin B6 metabolism, caffeine metabolism, amino sugar and nucleotide sugar metabolism, beta-alanine metabolism, fructose and mannose metabolism, and the pentose phosphate pathway. Among them, the bubbles of caffeine metabolism had the deepest color and the largest size in the four comparison groups, indicating that it was the most important channel in the analysis of pathways.

Subsequently, the different metabolites in four pairwise comparisons were mapped for enrichment analysis, and then significantly enriched pathways were obtained through *p*-value and pathway effect testing. The top 25 pathways are shown in Figure S5. The larger the enrichment ratio, the more reliable the enrichment significance. The integration of pathway analysis and enrichment analysis confirmed that the metabolic pathways were related to the cooking methods.

## 3. Materials and Methods

### 3.1. Chemicals and Reagents

Methanol (MeOH), acetonitrile (ACN), isopropyl alcohol (IPA), and ammonium hydroxide were of LC-MS grade and bought from Thermo Fisher Scientific (Rockford, IL, US). Ultrapure water was obtained from the Milli-Q system (Millipore, Bedford, MA, USA). Ammonium acetate used for mass spectroscopy was obtained from Aladdin Biochemical Technology Co., Ltd. (Shanghai, China).

### 3.2. Sample Preparation

The fungi used were identified as one species of the genus *Lentinula edodes* by analysis of the internal transcribed spacer (ITS) (max identity: 100%; query cover: 100%; accession: OR359298.1). The fresh samples were bought from a local market in Wuhan, China. Fresh samples were pretreated within 24 h of arrival at the laboratory.

Before being cooked, all *Lentinus edodes* samples were rinsed with mild ultrapure water to remove surface dust, and then ambiently air-dried. After that, the stalks were removed and the mushrooms were sliced into 0.5-cm-thick slices along the vertical axes. The raw *Lentinus edodes* were used as control samples to provide baseline comparisons. The cooking process was conducted by an experienced chef. The cooking methods were as follows:

For boiling, raw mushroom slices were poured into boiling ultrapure water and cooked for 10 min using a household electric cooker (MB-WFS4037, Midea, Foshan, Guangdong, China). For steaming, raw mushroom slices were placed on a steam grid when the steamer (MZ-ZG28Power501, Midea, Foshan, Guangdong, China) was full of steam and cooked for 10 min under atmospheric pressure. Air-frying was performed by a household air-fryer (8206, Shanben, Ningbo, Zhejiang, China). It has a removable bucket with a handle and a removable basket in the bucket. Raw mushroom slices were added to the basket during heating and cooked at 200 °C. It is necessary to shake the basket periodically to make sure the mushroom slices are cooked evenly. The mushroom slices were taken out after a total of 10 min of cooking. For roasting, a household electric oven (PT2531, Midea, Foshan, Guangdong, China) was employed. The mushroom slices were placed into the oven when the temperature reached the setting (200 °C). Each side took about 5 min for roasting, 10 min in total.

After cooking, the mushroom samples were directly frozen in liquid nitrogen and then kept at −80 °C. Later, the cooked and raw *Lentinus edodes* samples were freeze-dried and ground into powders for further experiments.

### 3.3. Extraction of Metabolites

To prepare the metabolic samples, the approach reported by Li et al. [45] was used with minor modifications. To be brief, 1.5 mL 80% aqueous methanol was added into 50 mg freeze-dried powder, and the mixture was vortexed for 2 min, processed by ultrasound for 30 min at low temperature, and then centrifuged at 13,000× *g* for 10 min (4 °C). After that, the supernatant was stored at 4 °C until analysis.

Meanwhile, the preparation of QC samples was conducted by equally mixing “raw”, “boiling”, “steaming”, “roasting”, and “air-frying” *Lentinus edodes* samples to monitor the reproducibility and stability of the system. All preparation of experimental samples was repeated three times.

### 3.4. Metabolomic Analysis

Untargeted metabolomic analysis of *Lentinus edodes* samples treated with different cooking methods was performed by a Vanquish UHPLC system equipped with a Q-Exactive™ Orbitrap Mass Spectrometer (Thermo Fisher Scientific, Waltham, MA, USA).

The metabolites extracted before were separated using a HILIC column (Waters, ACQUITY UPLC BEH Amide 1.7 μm, 2.1 mm × 100 mm). The injection volume was 2 μL, and a flow rate of 0.3 mL/min was used. A binary solvent system, comprising solvent A (water containing 25 mM ammonium hydroxide and 25 mM ammonium acetate) and solvent B (ACN). The elution gradient was set as follows: 0.0–1.5 min, 98% B; 1.5–12.0 min, 98% B decreasing linearly to 2%; 12.0–14.0 min, 2% B; 14.0–14.1 min, 2% B increasing linearly to 98%; and 14.1–17.0 min, 98% B. During the whole analysis, the temperature of the column chamber and the sample tray was 25 °C and 4 °C, respectively.

QC samples were injected at a 5-sample interval within each analytical set and were later used for batch correction during data analysis. To ensure the reliability and accuracy of the results, the injection order was randomized and extraction blanks were required.

The MS and MS/MS data were obtained using the Thermo Q-Exactive^TM^ Orbitrap mass spectrometer. The parameters of the ESI source were as follows: both Gas1 and Gas2 were 60 psi, curtain gas (CUR) was 35 psi, source temperature was set to 550 °C, and Ion Spray Voltage Floating (ISVF) were 5000 V and −5000 V (in positive mode and negative mode). In MS-only acquisition, the scanning range was 80–1200 Da, the resolution was set at 60,000 and the accumulation time was 100 ms. In auto-MS/MS-acquisition, the scanning range was 70–1200 Da, the resolution was 30,000, and the accumulation time was set at 50 ms, excluding time within 4 s.

### 3.5. Data Processing

The freely available software XCMS was used for peak grouping, alignment, and clustering. The detailed parameters for peak picking were as follows: centWave *m/z* = 25 ppm, peakwidth = c (10, 60), prefilter = c (10, 100); and the parameters for peak grouping were as follows: bw = 5, mzwid = 0.025, minfrac = 0.5. Isotope/adduct annotation was performed by the open-source software package CAMERA (Collection of Algorithms of MEtabolite pRofile Annotation). The integrity of the data was first checked in the extracted ion features. Variables with a missing value of more than 50% in the group would be removed and excluded from subsequent analysis. Metabolite identification was carried out by the comparison of the accuracy *m/z* value (<25 ppm) and MS/MS spectra with an established in-house database. Peak intensities were further normalized by constant sum, Log transformation, and Pareto Scaling.

### 3.6. Statistical Analysis

Unsupervised and supervised chemometric methods, such as principal component analysis (PCA) and orthogonal partial least squares discriminant analysis (OPLS-DA), were conducted by SIMCA 14.1 (MKS Umetrics AB, Malmö, Sweden). Additionally, SPSS V26.0 (SPSS Inc., Chicago, IL, USA) was used for one-way analysis of variance (ANOVA). Heatmaps and pathway analysis were performed using the online platform MetaboAnalyst 5.0 (https://www.metaboanalyst.ca/, accessed on 12 April 2023). The Kyoto Encyclopedia of Genes and Genomes (KEGG) compound database (https://www.kegg.jp/, accessed on 16 April 2022) was used for pathway annotation. GraphPad Prism 8.0 software (GraphPad Software Inc., La Jolla, CA, USA) and Origin Pro version 2021 (OriginLab Corporation, Northampton, MA, USA) were used to generate graphs.

## 4. Conclusions

Herein, metabolites of *Lentinus edodes* with distinct cooking methods (steaming, boiling, air-frying, and roasting) were investigated and compared based on untargeted metabolomics in combination with chemometrics. The results revealed that the four types of cooking methods had obvious and different effects on *Lentinus edodes*. Boiling appeared to result in a massive loss of metabolites, while air-frying and roasting seemed to have more upregulated metabolites. The number of upregulated and downregulated metabolites between raw and steamed *Lentinus edodes* was approximately the same. In addition, the results have been confirmed by pathway and enrichment analysis. This study provided basic knowledge on the effects of different cooking methods on *Lentinus edodes* metabolites and laid the foundation for better development and consumption of *Lentinus edodes* and its products.

## Data Availability

The data that support the findings of this study are available on request from the corresponding author.

## Supplementary Materials

The following supporting information can be downloaded at: https://www.mdpi.com/article/10.3390/molecules28166009/s1, Figure S1: CV values Distribution of all the features in untargeted metabolomics. Figure S2: Category statistics for all metabolites. Figure S3: Cross-validation plot of the OPLS-DA model with 200 permutation tests. (A) Boiling vs. Control; (B) Steaming vs. Control; (C) Air-frying vs. Control; (D) Roasting vs. Control. Figure S4: Heatmap visualization of differential metabolites classified in distinct categories in boiling, steaming, air-frying and roasting treatments, respectively. Figure S5: KEGG annotations and enrichment of differentially expressed metabolites of each pairwise comparison of *Lentinus edodes*. (A) Boiling vs. Control; (B) Steaming vs. Control; (C) Air-frying vs. Control; (D) Roasting vs. Control. Table S1: List of identified metabolites in *Lentinus edodes*. Table S2: Classification of differential metabolites in four pairwise comparisons. Table S3: List of differential metabolites between Boiling and Control. Table S4: List of differential metabolites between Steaming and Control. Table S5: List of differential metabolites between Air-frying and Control. Table S6: List of differential metabolites between Roasting and Control.
