# Supplementary material for: Untargeted Metabolomic Analysis Combined with Chemometrics Revealed the Effects of Different Cooking Methods on Lentinus edodes
Source: Molecules. 2023 Aug 11;28(16):6009. doi: 10.3390/molecules28166009 (PMC10458448; doi:10.3390/molecules28166009)
Supplement: Supplementary file 1 [file molecules-28-06009-s001.zip › supplymentary Figure.pdf]

Supplemental materials

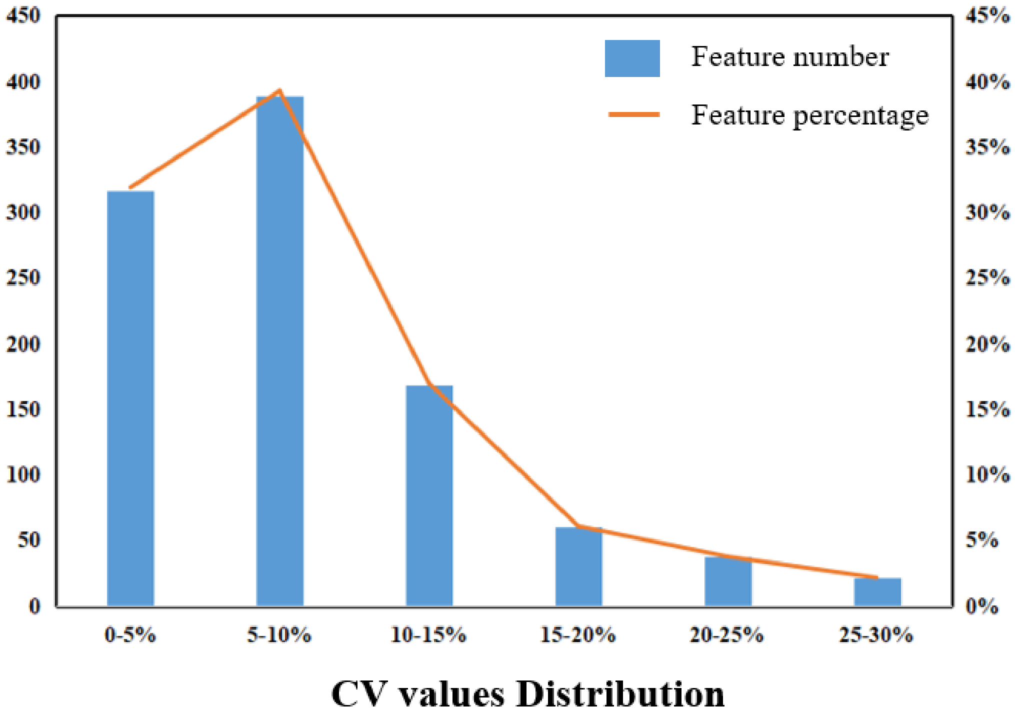

Figure S1 CV values Distribution of all the features in untargeted metabolomics.

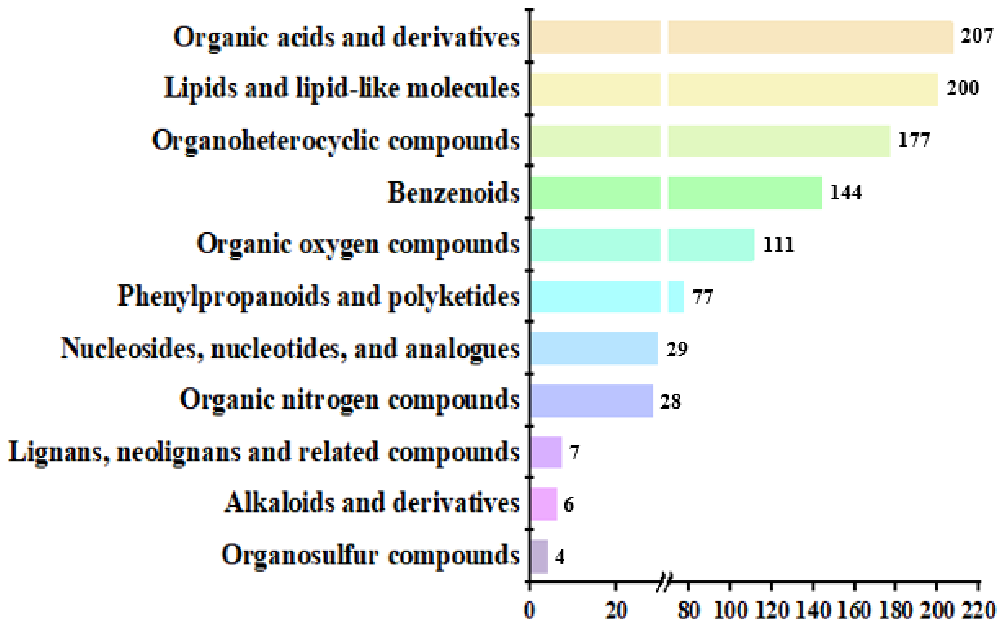

Figure S2 Category statistics for all metabolites.

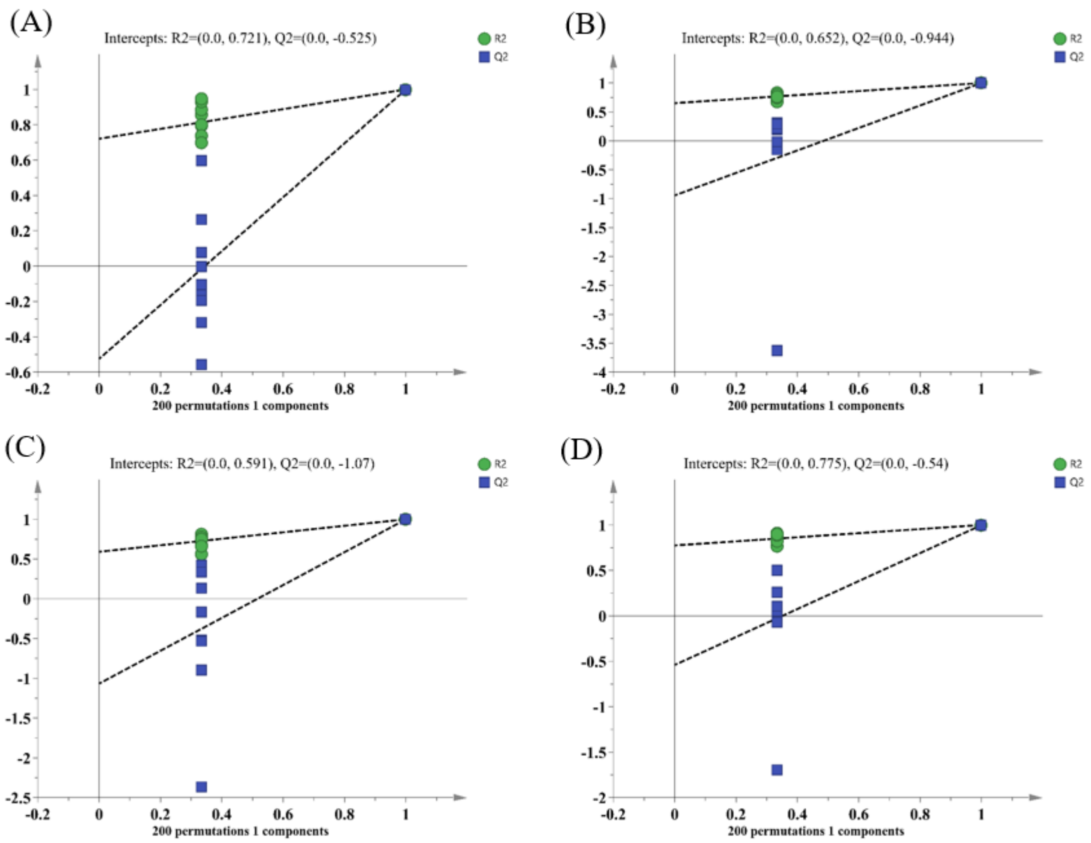

Figure S3 Cross-validation plot of the OPLS-DA model with 200 permutation tests. (A) Boiling vs. Control; (B) Steaming vs. Control; (C) Air-frying vs. Control; (D) Roasting vs. Control.

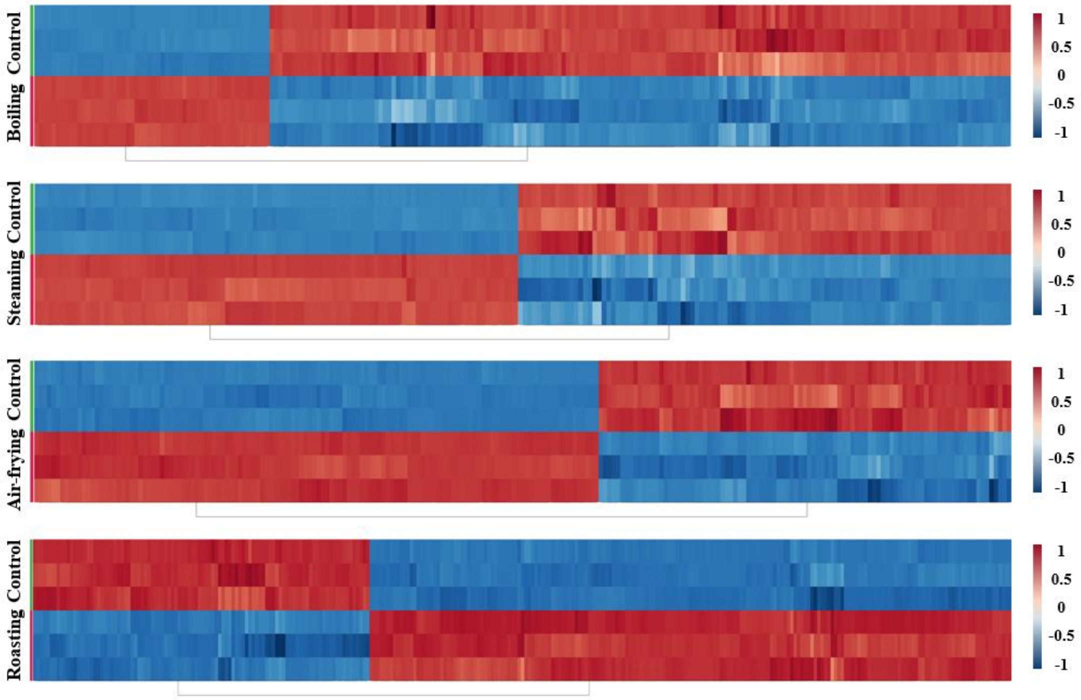

Figure S4 Heat map visualization of differential metabolites classified in distinct categories in boiling, steaming, air-frying and roasting treatments, respectively.

(A)

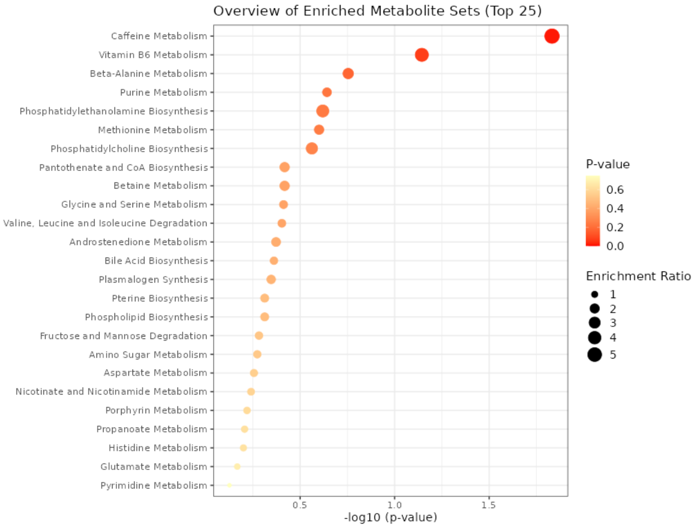

Overview of Enriched Metabolite Sets (Top 25)
Caffeine Metabolism
Vitamin B6 Metabolism
Beta-Alanine Metabolism
Purine Metabolism
Phosphatidylethanolamine Biosynthesis
Methionine Metabolism
Phosphatidylcholine Biosynthesis
Pantothenate and CoA Biosynthesis
Betaine Metabolism
Glycine and Serine Metabolism
Valine, Leucine and Isoleucine Degradation
Androstenedione Metabolism
Bile Acid Biosynthesis
Plasmalogen Synthesis
Pterine Biosynthesis
Phospholipid Biosynthesis
Fructose and Mannose Degradation
Amino Sugar Metabolism
Aspartate Metabolism
Nicotinate and Nicotinamide Metabolism
Porphyrin Metabolism
Propanoate Metabolism
Histidine Metabolism
Glutamate Metabolism
Pyrimidine Metabolism
0.5
1.0
1.5
-log10 (p-value)
P-value
0.6
0.4
0.2
0.0
Enrichment Ratio
1
2
3
4
5

(B)

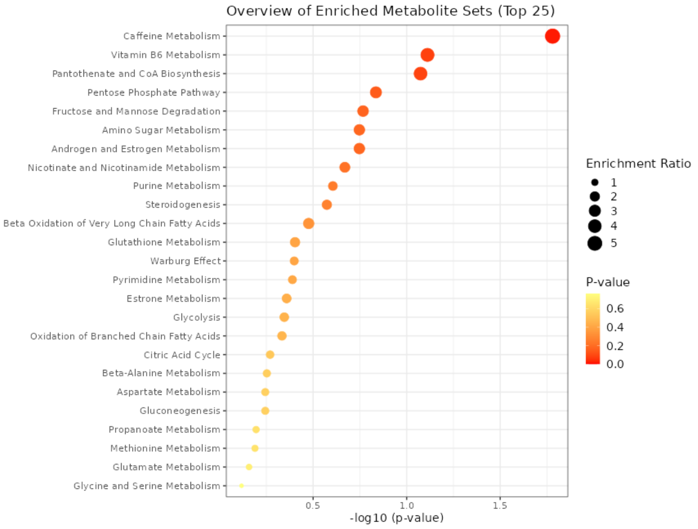

Overview of Enriched Metabolite Sets (Top 25)
Caffeine Metabolism
Vitamin B6 Metabolism
Pantothenate and CoA Biosynthesis
Pentose Phosphate Pathway
Fructose and Mannose Degradation
Amino Sugar Metabolism
Androgen and Estrogen Metabolism
Nicotinate and Nicotinamide Metabolism
Purine Metabolism
Steroidogenesis
Beta Oxidation of Very Long Chain Fatty Acids
Glutathione Metabolism
Warburg Effect
Pyrimidine Metabolism
Estrone Metabolism
Glycolysis
Oxidation of Branched Chain Fatty Acids
Citric Acid Cycle
Beta-Alanine Metabolism
Aspartate Metabolism
Gluconeogenesis
Propanoate Metabolism
Methionine Metabolism
Glutamate Metabolism
Glycine and Serine Metabolism
0.5
1.0
1.5
-log10 (p-value)
Enrichment Ratio
1
2
3
4
5
P-value
0.6
0.4
0.2
0.0

(C)

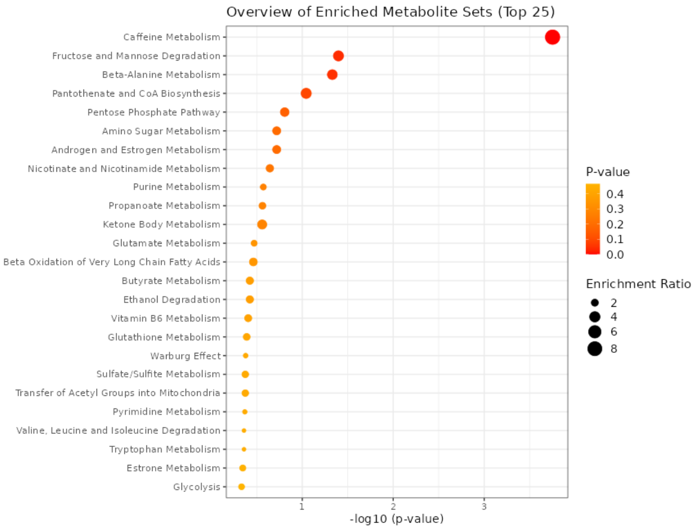

Overview of Enriched Metabolite Sets (Top 25)
Caffeine Metabolism
Fructose and Mannose Degradation
Beta-Alanine Metabolism
Pantothenate and CoA Biosynthesis
Pentose Phosphate Pathway
Amino Sugar Metabolism
Androgen and Estrogen Metabolism
Nicotinate and Nicotinamide Metabolism
Purine Metabolism
Propanoate Metabolism
Ketone Body Metabolism
Glutamate Metabolism
Beta Oxidation of Very Long Chain Fatty Acids
Butyrate Metabolism
Ethanol Degradation
Vitamin B6 Metabolism
Glutathione Metabolism
Warburg Effect
Sulfate/Sulfite Metabolism
Transfer of Acetyl Groups into Mitochondria
Pyrimidine Metabolism
Valine, Leucine and Isoleucine Degradation
Tryptophan Metabolism
Estrone Metabolism
Glycolysis
1
2
3
-log10 (p-value)
P-value
0.4
0.3
0.2
0.1
0.0
Enrichment Ratio
2
4
6
8

(D)

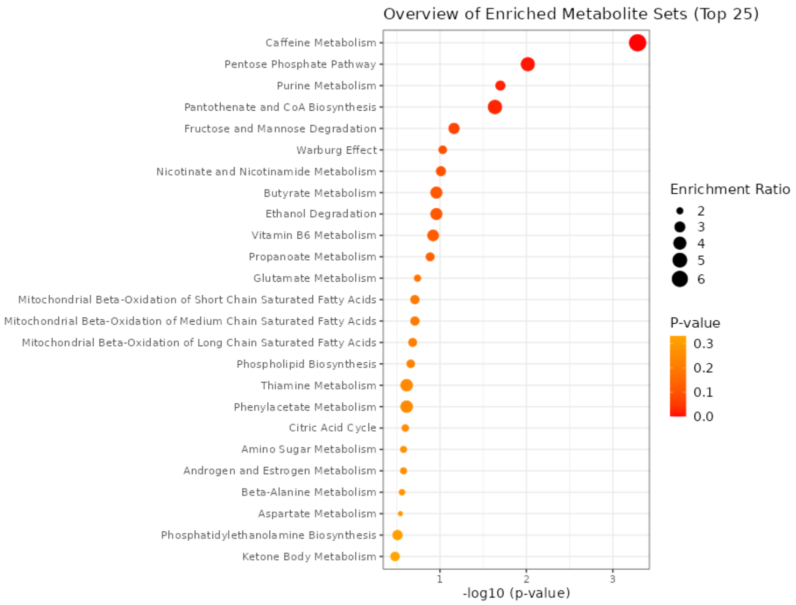

Figure S5 KEGG annotations and enrichment of differentially expressed metabolites of each pairwise comparison of *Lentinus edode*. (A) Boiling vs. Control; (B) Steaming vs. Control; (C) Air-frying vs. Control; (D) Roasting vs. Control.
