# Supplementary material for: Untargeted Metabolomic Analysis Combined with Chemometrics Revealed the Effects of Different Cooking Methods on Lentinus edodes
Source: Molecules. 2023 Aug 11;28(16):6009. doi: 10.3390/molecules28166009 (PMC10458448; doi:10.3390/molecules28166009)
Supplement: Supplementary file 1 [file molecules-28-06009-s001.zip › Table S1.pdf]

Table S1 List of identified metabolites in *Lentinus edode*

m/z	Name	SuperClass	QC	Control	Boiling	Steaming	Air-frying	Roasting
205.1334	Caulophylline	Alkaloids and derivatives	0.9275±0.0351	1.940±0.134	0.4820±0.0270	0.6058±0.0615	2.239±0.145	1.088±0.082
460.2520	Cytochalasin a	Alkaloids and derivatives	0.4555±0.1087	0.5509±0.0250	0.2502±0.0127	0.3895±0.0325	0.4309±0.0144	0.6507±0.0265
324.1899	Lsd	Alkaloids and derivatives	7.099±0.848	4.147±0.282	2.052±0.187	5.961±0.404	10.83±0.73	14.02±1.16
340.1847	Methylergonovine	Alkaloids and derivatives	0.8092±0.1868	0.5030±0.0255	0.1968±0.0117	0.7752±0.0607	0.8715±0.0374	1.595±0.056
205.1336	N-methylcytisine	Alkaloids and derivatives	1.196±0.296	2.022±0.159	0.5655±0.0470	0.8769±0.1047	3.541±0.223	0.8884±0.0530
218.1387	Pentazocine	Alkaloids and derivatives	260.5±10.0	191.9±10.5	158.4±5.2	255.3±7.5	296.8±24.6	412.8±22.3
152.0919	(-)-norephedrine	Benzenoids	6.949±0.794	10.37±0.43	4.158±0.062	7.119±0.115	7.724±0.331	7.944±0.112
190.1438	(1s,2r)-2-(diethylamino)-1-phenyl-1-propanol	Benzenoids	505.2±17.5	700.7±37.4	285.6±7.5	486.6±16.6	532.5±32.7	543.2±29.2
421.1222	(5-benzoyloxy-4,6-dihydroxy-3-methoxycyclohexen-1-yl)methyl benzoate	Benzenoids	10.60±0.47	2.321±0.109	7.915±0.092	13.51±0.31	11.91±0.84	16.87±1.09
145.0609	.beta.-naphthol	Benzenoids	2.971±0.224	11.49±0.40	2.002±0.189	3.320±0.242	2.589±0.228	2.733±0.228
293.1759	[6]-gingerol	Benzenoids	28.08±2.59	14.62±0.63	20.06±1.26	12.25±1.10	13.45±0.43	13.72±0.36
231.0184	1-(3,4-dichlorophenyl)-3,3-dimethylurea	Benzenoids	815.5±26.8	1110±35	522.4±25.0	760.6±26.0	825.3±27.7	831.9±45.3
125.0179	1,2,3-benzenetriol	Benzenoids	38.32±2.15	41.61±3.84	21.01±0.71	32.58±2.14	34.30±3.52	40.02±0.78
253.1799	1-benzhydrylpiperazine	Benzenoids	5.472±0.716	17.46±1.59	1.762±0.137	3.321±0.103	6.217±0.427	5.878±0.711
568.1854	1h-indole-5-sulfonamide, n-(3-chlorophenyl)-3-[[3,5-dimethyl-4-[(4-methyl-1-piperazinyl)carbonyl]-1h-pyrrol-2-yl]methylene]-2,3-dihydro-n-methyl-2-oxo-, (3z)-	Benzenoids	6.789±0.573	8.620±0.729	7.996±0.300	8.534±0.104	4.736±0.507	6.381±0.201
178.0719	2,2-bis(4-chlorophenyl)ethanol	Benzenoids	4.466±0.281	31.19±2.23	2.421±0.165	2.195±0.168	3.200±0.204	2.798±0.166
153.0198	2,3-dihydroxybenzoic acid	Benzenoids	8.720±0.516	8.845±0.712	5.035±0.255	10.00±0.16	9.897±1.012	10.37±0.59
188.9355	2,4-dichlorobenzoic acid	Benzenoids	45.17±5.90	14.22±0.48	7.144±0.260	22.72±1.17	104.6±2.7	66.56±1.92
123.0438	2,6-dimethoxyphenol	Benzenoids	33.14±1.19	36.23±1.23	23.10±0.42	31.41±0.87	32.79±1.69	39.88±0.92
110.0601	2-aminophenol	Benzenoids	8.410±0.327	3.251±0.268	1.991±0.141	3.433±0.140	22.85±0.74	8.579±0.180

208.0968	2-chloro-2',6'-diethylacetanilide	Benzenoids	0.6815±0.1555	0.06858±0.00637	0.6309±0.0585	1.729±0.134	0.4802±0.0339	0.4967±0.0367
245.0432	2'-chloro-2-hydroxy-5-methylbenzophenone	Benzenoids	23.13±0.38	30.48±0.90	10.16±0.46	19.49±0.37	25.71±1.13	27.91±0.32
207.1130	2-ethyl-2-phenylmalonamide	Benzenoids	7.845±0.163	17.80±1.33	4.699±0.215	6.953±0.045	7.910±0.458	9.298±0.115
229.0807	2-hydroxy-4-methoxybenzophenone	Benzenoids	48.52±3.82	64.13±1.89	33.74±0.46	47.81±1.13	45.97±4.26	48.50±2.27
151.0407	2-hydroxyphenylacetic acid	Benzenoids	6.324±0.313	10.42±0.52	3.261±0.205	6.165±0.177	6.354±0.584	7.682±0.472
168.0306	2-methoxy-5-nitrophenol	Benzenoids	0.3852±0.0348	2.312±0.039	0.7696±0.0385	0.5718±0.0144	0.6400±0.0114	0.3923±0.0068
227.1754	3,3'-dimethyl-4,4'-diaminodiphenylmethane	Benzenoids	11.01±1.12	1.819±0.024	0.6574±0.0435	0.9697±0.0190	47.17±3.58	0.5438±0.0251
227.0198	3,5-dinitrosalicylate	Benzenoids	3.043±0.236	11.13±0.74	2.323±0.165	2.265±0.265	7.746±0.281	3.532±0.333
152.0353	3-hydroxyanthranilic acid	Benzenoids	8.257±2.019	6.068±0.190	1.817±0.064	3.009±0.099	12.05±0.50	8.394±0.210
256.0827	3-hydroxymethylmefenamic acid	Benzenoids	241.7±44.1	641.1±41.8	118.8±5.2	225.4±11.4	206.7±22.7	204.3±15.6
107.0502	3-hydroxyphenylacetic acid	Benzenoids	5.618±0.518	9.105±0.324	3.081±0.131	5.366±0.139	5.508±0.392	6.001±0.151
170.0813	4-aminobiphenyl	Benzenoids	8.426±0.469	15.64±1.28	4.064±0.199	8.724±0.054	9.333±0.920	13.27±0.11
110.0714	4-aminophenol	Benzenoids	95.78±19.45	60.52±2.52	96.29±3.16	88.78±8.15	88.38±3.29	82.55±3.43
127.0037	4-chlorophenol	Benzenoids	11.43±0.88	16.48±0.65	9.972±0.998	14.36±0.69	7.541±0.380	10.58±0.77
109.0408	4-fluorophenylacetic acid	Benzenoids	8.477±0.803	8.186±0.830	7.142±0.446	7.501±0.426	8.293±0.325	8.595±0.219
201.0074	4-hydroxy-2',5'-dichlorobiphenyl	Benzenoids	80.54±3.70	175.7±3.4	41.61±1.96	80.93±2.03	76.44±0.95	88.39±0.64
105.0699	4-methylbenzyl alcohol	Benzenoids	22.98±2.21	72.25±3.97	5.706±0.335	5.835±0.166	32.93±3.25	17.35±1.05
199.1024	4-nitrosodiphenylamine	Benzenoids	89.59±7.83	63.21±2.81	104.7±3.7	102.7±7.3	92.52±4.95	94.86±2.38
289.0236	5-benzoyl-4-hydroxy-2-methoxybenzenesulfonic acid	Benzenoids	3.603±0.227	9.607±0.338	1.529±0.069	4.662±0.062	4.123±0.110	2.855±0.084
321.2105	8-gingerol	Benzenoids	34.20±0.61	41.47±1.90	32.07±1.53	37.24±0.94	28.53±1.60	32.78±0.95
299.0984	8phic8spc	Benzenoids	22.77±5.26	34.56±1.02	13.83±0.47	20.14±0.36	25.03±1.83	24.14±2.53
417.2712	Alfentanyl	Benzenoids	1.110±0.081	2.007±0.144	0.5098±0.0327	1.069±0.013	1.086±0.020	1.290±0.067
152.1184	Aminocarb	Benzenoids	3.906±0.098	3.668±0.293	3.832±0.046	3.929±0.038	3.736±0.168	3.668±0.110
245.1536	Amprenavir	Benzenoids	2.391±0.289	2.036±0.036	2.860±0.082	3.680±0.021	4.601±0.397	2.880±0.085
266.1250	Anisomycin	Benzenoids	11.84±1.77	38.84±1.62	6.566±0.412	8.469±0.065	11.27±1.39	13.08±1.09
367.1217	Atovaquone	Benzenoids	8.819±0.719	6.786±0.470	14.55±0.19	9.486±0.110	7.304±0.411	9.232±1.111

329.0686	Aurantio-obtusin	Benzenoids	3.513±0.672	3.611±0.048	1.653±0.057	2.175±0.082	2.804±0.075	3.051±0.310
404.1335	Azoxystrobin	Benzenoids	0.7342±0.0382	0.2330±0.0074	0.7842±0.0617	0.7864±0.0612	0.3172±0.0083	0.9224±0.0666
409.0794	Bensulfuron-methyl	Benzenoids	1.971±0.124	1.739±0.134	1.520±0.151	1.872±0.120	1.735±0.051	1.852±0.148
122.0729	Benzamide	Benzenoids	7.179±0.597	10.05±0.38	6.174±0.314	6.551±0.783	7.021±0.391	10.47±0.07
264.9988	Benzamide, 2-[(2-chloro-4-iodophenyl)amino]-n-(cyclopropylmethoxy)-3,4-difluoro-	Benzenoids	0.9916±0.0857	1.017±0.050	0.6948±0.0051	1.047±0.043	1.042±0.023	1.498±0.009
277.0335	Benzamide, 2-chloro-5-nitro-n-phenyl-	Benzenoids	4.166±0.357	4.132±0.075	2.846±0.177	4.124±0.384	4.584±0.205	4.936±0.205
289.1034	Benzamide, 3-[[(2e)-3-(1h-indol-3-yl)-1-oxo-2-propen-1-yl]amino]-	Benzenoids	2.606±0.369	4.400±0.337	2.949±0.124	3.024±0.195	2.796±0.270	2.700±0.080
392.1302	Benzamide, 4-[(3ar,4r,7s,7as)-1,3,3a,4,7,7a-hexahydro-1,3-dioxo-4,7-methano-2h-isoindol-2-yl]-n-8-quinolinyl-, rel-	Benzenoids	0.7544±0.0563	1.476±0.063	0.2626±0.0059	0.4905±0.0173	0.7893±0.0439	1.007±0.084
198.0760	Benzanilide	Benzenoids	206.7±8.0	251.2±11.8	127.9±3.8	276.4±8.4	199.2±15.7	177.8±9.7
377.0693	Benzenepropanamide, n-(6-chloro-2-benzothiazolyl)-3,4-dimethoxy-	Benzenoids	5.181±0.213	18.02±1.15	1.525±0.067	3.712±0.162	5.635±0.256	2.424±0.193
378.1373	Benzenesulfonamide, 4-(4,5-dihydro-3,5-diphenyl-1h-pyrazol-1-yl)-	Benzenoids	1.528±0.077	0.9585±0.0237	3.043±0.088	1.657±0.151	1.532±0.049	1.267±0.036
446.0851	Benzenesulfonamide, n-[[(1,1-dimethylethyl)amino]carbonyl]-2-(3-methoxyphenoxy)-5-nitro-	Benzenoids	0.1753±0.0231	0.1790±0.0136	0.02041±0.00057	0.2593±0.0285	0.2440±0.0027	0.2474±0.0058
354.9838	Benzenesulfonic acid, 2-[(5-bromo-2-hydroxyphenyl)methylene]hydrazide	Benzenoids	3.834±0.354	0.3494±0.0184	1.528±0.077	1.594±0.072	0.8438±0.0049	3.566±0.190
335.1316	Benzyl butyl phthalate	Benzenoids	19.09±1.23	30.63±0.96	12.84±0.31	20.03±0.73	15.50±1.13	18.01±0.35
214.0864	Benzyl nicotinate	Benzenoids	83.17±7.43	73.31±3.23	35.72±1.01	85.47±4.03	117.1±10.3	98.04±4.88
448.0857	Bicalutamide	Benzenoids	0.7614±0.0810	1.275±0.017	0.3733±0.0119	0.7716±0.0142	0.7920±0.0670	0.8361±0.0490
363.1489	Bisphenol a diglycidyl ether	Benzenoids	1.340±0.184	1.471±0.043	1.207±0.121	1.146±0.064	1.091±0.039	1.463±0.043
208.9615	Botran	Benzenoids	0.2219±0.0137	0.2218±0.0121	0.1167±0.0039	0.1982±0.0136	0.2282±0.0135	0.2479±0.0091

330.9844	Butafenacil	Benzenoids	2.440±0.184	3.953±0.046	1.153±0.057	2.400±0.014	2.585±0.183	2.561±0.079
333.1159	Butamifos	Benzenoids	0.9176±0.0646	1.147±0.052	0.7350±0.0426	1.092±0.043	0.8566±0.0080	1.011±0.081
387.1512	Cetirizine	Benzenoids	0.2328±0.0174	0.1351±0.0059	#NUM!±0.00	0.2282±0.0162	0.2455±0.0163	0.2181±0.0090
357.0561	Chlorophacinone	Benzenoids	2.172±0.100	2.922±0.161	2.077±0.051	2.470±0.014	1.914±0.197	1.725±0.059
287.0410	Ciprofibrate	Benzenoids	7.534±0.693	23.55±1.28	4.154±0.312	7.116±0.287	14.44±0.65	13.65±0.88
240.0869	Clomazon	Benzenoids	6.382±0.252	9.160±0.348	4.057±0.108	6.016±0.088	6.708±0.620	6.118±0.178
179.0810	Coniferyl aldehyde	Benzenoids	70.08±1.85	102.0±5.5	48.02±0.86	64.47±1.51	80.89±6.02	71.36±1.06
278.1423	Convolidine	Benzenoids	5.166±0.220	8.263±0.458	2.840±0.090	4.606±0.177	4.850±0.165	5.960±0.305
124.1122	Convolvamine	Benzenoids	3.268±0.192	4.956±0.210	2.083±0.037	3.460±0.121	3.646±0.268	3.417±0.140
204.1339	Crotamiton	Benzenoids	912.2±42.6	425.6±11.7	812.3±23.2	896.4±26.4	841.8±38.4	795.2±43.9
397.1157	Daunomycinone	Benzenoids	3.494±0.756	8.189±0.147	2.099±0.158	2.732±0.140	2.136±0.065	1.731±0.068
323.0951	Desmedipham	Benzenoids	9.619±0.631	8.312±0.454	12.91±0.37	12.23±0.15	8.914±0.840	7.900±0.269
441.3075	Di(2-nonyl) phthalate	Benzenoids	1.083±0.053	3.346±0.199	0.4434±0.0208	1.522±0.027	0.7215±0.0137	0.9549±0.0491
317.1112	Dibutyl phthalate	Benzenoids	1.215±0.046	0.1847±0.0059	0.5135±0.0108	1.113±0.061	1.936±0.023	2.105±0.012
333.0594	Diflufenzopyr	Benzenoids	50.78±3.09	73.91±2.94	41.36±1.87	53.09±1.92	50.29±5.69	46.90±0.83
224.0767	Dioxacarb	Benzenoids	45.02±0.46	53.23±2.93	26.37±0.35	44.91±1.48	38.86±1.90	60.08±0.70
273.1083	Dipropyl phthalate	Benzenoids	24.95±1.16	44.39±0.63	11.24±0.27	21.71±0.62	31.86±1.93	25.44±1.86
395.1001	Dl-4-hydroxy-3-methoxymandelic acid	Benzenoids	4.236±0.354	6.270±0.126	2.669±0.139	3.992±0.383	3.449±0.066	3.229±0.166
136.0758	Dl-octopamine	Benzenoids	86.18±2.42	92.09±1.09	60.13±1.06	82.67±2.55	87.87±1.24	103.4±1.8
148.0969	Ephedrine	Benzenoids	3.715±0.385	6.265±0.542	4.092±0.096	3.510±0.068	4.776±0.189	4.448±0.342
169.0584	Ethyl 2,4-dihydroxy-6-methylbenzoate	Benzenoids	9.351±0.116	6.236±0.111	22.46±1.06	10.49±0.44	7.858±0.382	8.695±0.207
639.1902	Fenamifos sulfoxide	Benzenoids	2.040±0.167	2.626±0.081	1.476±0.030	2.305±0.050	1.811±0.068	2.224±0.082
159.0279	Fenfluramine	Benzenoids	3.217±0.395	3.861±0.387	0.8188±0.0048	0.3598±0.0197	2.034±0.117	1.959±0.015
386.1065	Flamprop-isopropyl	Benzenoids	1.097±0.044	0.1580±0.0035	0.1460±0.0063	0.2642±0.0056	3.415±0.168	0.3676±0.0168
152.0562	Flufenacet	Benzenoids	21.37±1.16	5.139±0.223	4.798±0.138	8.041±0.090	39.45±2.02	46.59±0.93
297.0882	Flunixin	Benzenoids	2.268±0.188	7.740±0.172	0.8484±0.0800	2.020±0.154	2.291±0.038	2.694±0.214
231.0511	Fluometuron	Benzenoids	33.99±3.70	69.56±6.66	18.62±0.38	32.68±1.87	33.34±1.19	37.52±2.14
329.0248	Fluorodifen	Benzenoids	1.211±0.167	1.686±0.162	0.9931±0.0816	1.096±0.031	1.051±0.046	1.017±0.069
316.1006	Flusilazole	Benzenoids	13.99±1.18	#NUM!±0.00	0.1572±0.0162	0.1438±0.0091	54.76±2.71	11.05±1.20

277.0896	Flutamide	Benzenoids	12.26±1.40	9.838±0.177	12.88±0.42	10.82±0.57	8.560±0.511	11.21±0.26
343.1725	Formoterol	Benzenoids	1.360±0.115	2.410±0.194	0.9377±0.0433	1.348±0.063	1.360±0.045	1.531±0.060
415.1266	Frangulin a	Benzenoids	1.453±0.077	0.9167±0.0827	3.007±0.139	1.869±0.034	1.269±0.093	1.372±0.066
314.0055	Furosemide	Benzenoids	3.189±0.182	3.670±0.046	2.103±0.082	2.393±0.109	3.441±0.124	3.596±0.106
169.0142	Gallic acid	Benzenoids	12.96±1.14	11.04±0.97	10.52±0.45	12.72±0.48	13.45±0.75	12.47±0.08
153.0193	Gentisic acid	Benzenoids	100.1±7.5	73.00±5.72	122.6±10.1	220.7±7.8	55.50±4.78	78.53±0.72
376.0642	Haloxyfop-methyl	Benzenoids	2.142±0.348	3.883±0.102	1.590±0.109	2.771±0.272	1.779±0.188	1.108±0.047
261.1345	Heptanedioic acid, 1-[2-[(2-carboxyphenyl)methylene]hydrazide]	Benzenoids	0.8978±0.0227	1.247±0.065	0.5377±0.0261	0.8536±0.0556	1.074±0.096	0.9928±0.0507
149.0455	Homogentisic acid	Benzenoids	6.212±0.146	9.008±0.652	4.327±0.271	6.500±0.086	6.193±0.212	6.100±0.398
111.0554	Hydroquinone	Benzenoids	3.355±0.302	4.073±0.084	3.544±0.080	3.298±0.023	3.107±0.219	3.379±0.211
293.0635	Hydroxyflutamide	Benzenoids	21.50±1.47	30.13±1.71	9.205±0.286	21.20±0.60	22.64±0.76	21.52±1.29
494.2590	Imatinib	Benzenoids	1.134±0.056	#NUM!±0.00	0.3211±0.0187	0.9453±0.0325	2.200±0.047	2.018±0.151
194.1177	Isoproterenol	Benzenoids	1.033±0.181	1.208±0.119	#NUM!±0.00	1.033±0.035	1.089±0.056	1.088±0.032
429.1250	Ketoprofen .beta.-d-glucuronide	Benzenoids	6.124±0.346	8.058±0.154	5.134±0.246	5.782±0.071	6.109±0.324	5.696±0.125
251.0410	Lucidin	Benzenoids	15.04±0.79	21.42±1.32	8.980±0.410	15.50±0.35	15.78±1.18	17.46±1.46
265.1479	Magnolol	Benzenoids	664.0±12.9	798.4±24.8	1060±48	566.7±17.7	432.0±22.6	536.2±33.5
412.1104	Mandipropamid	Benzenoids	2.290±0.232	7.696±0.137	0.3599±0.0244	0.3187±0.0106	1.976±0.044	2.721±0.101
296.0992	Mebendazole	Benzenoids	1.362±0.036	2.217±0.057	1.090±0.083	1.334±0.099	1.432±0.090	1.218±0.044
343.1615	Methanone, 1-naphthalenyl(1-pentyl-1h-indazol-3-yl)-	Benzenoids	0.8443±0.0686	0.1111±0.0061	0.7521±0.0087	1.056±0.044	1.433±0.042	0.5887±0.0219
318.1559	M-hydroxycocaine	Benzenoids	0.2523±0.0072	0.1165±0.0034	0.08303±0.00776	0.1905±0.0139	0.2643±0.0058	0.2679±0.0115
399.1467	N,n'-dicarbobenzyloxy-l-ornithine	Benzenoids	3.451±0.256	6.084±0.195	1.086±0.049	3.073±0.019	2.974±0.094	3.373±0.102
199.0724	N,n-dimethyl-n'-phenylsulfamide	Benzenoids	9.493±0.532	11.03±0.25	4.421±0.054	7.855±0.084	14.75±1.01	10.28±0.22
159.0299	Naphthoresorcinol	Benzenoids	543.9±128.9	657.5±44.4	360.4±22.6	604.3±60.3	670.3±21.0	617.9±34.6
215.0562	Nepodin	Benzenoids	10.34±2.07	25.51±0.82	5.852±0.267	11.42±1.03	9.449±0.633	10.37±0.30
184.0616	N-hydroxy-4-aminobiphenyl	Benzenoids	24.77±2.35	10.01±0.82	7.097±0.416	11.90±0.66	65.27±2.37	22.85±1.97
300.2171	Nylidrin	Benzenoids	2.515±0.061	4.876±0.137	1.543±0.038	2.550±0.037	2.682±0.078	3.293±0.097

279.1188	Oxadixyl	Benzenoids	1.852±0.306	2.047±0.096	3.336±0.196	0.7306±0.0264	3.455±0.318	2.391±0.246
387.1046	Pamoic acid	Benzenoids	5.889±0.618	3.251±0.152	1.907±0.158	4.579±0.382	7.923±0.647	6.544±0.303
276.0846	Paraoxon	Benzenoids	16.08±1.08	23.54±1.17	6.817±0.367	4.032±0.194	18.35±1.69	21.46±1.45
369.0828	Parecoxib	Benzenoids	0.5462±0.0824	0.7216±0.0617	0.3395±0.0257	0.5969±0.0216	0.5610±0.0379	0.5366±0.0228
212.0686	Pendimethalin	Benzenoids	0.4747±0.0039	0.6077±0.0041	0.3949±0.0133	0.4481±0.0229	0.4556±0.0468	0.5266±0.0232
250.0355	Phaclofen	Benzenoids	12.10±0.51	16.40±1.00	11.73±0.46	12.75±0.43	12.84±0.82	12.12±0.39
180.1133	Phenacetin	Benzenoids	2.952±0.193	3.897±0.234	2.233±0.123	2.879±0.019	3.306±0.065	2.822±0.099
95.0492	Phenol	Benzenoids	4.976±0.290	5.399±0.111	3.400±0.072	4.695±0.176	5.135±0.092	5.853±0.130
122.0964	Phenylethylamine	Benzenoids	9.508±1.043	30.06±1.98	2.630±0.063	2.548±0.046	13.21±0.84	6.727±0.243
149.0244	Phenylglyoxylic acid	Benzenoids	50.51±3.16	60.91±2.50	33.91±1.73	61.66±2.03	44.16±2.44	58.23±0.72
163.0401	Phenylpyruvate	Benzenoids	6.168±0.473	5.774±0.105	3.365±0.176	7.650±0.117	7.314±0.389	8.371±0.171
201.0398	Propanedinitrile, 2-[(3,4,5-trihydroxyphenyl)methylene]-	Benzenoids	35.04±5.51	60.35±6.07	25.75±0.89	33.62±2.35	33.17±3.33	34.42±0.64
391.0882	Propanoic acid, 2-methyl-2-[4-[[[[4-methyl-2-[4-(trifluoromethyl)phenyl]-5-thiazolyl]carbonyl]amino]methyl]phenoxy]-	Benzenoids	2.686±0.270	2.786±0.213	2.543±0.230	2.832±0.108	3.651±0.163	2.432±0.088
365.1420	Pyridaben	Benzenoids	61.23±3.18	81.20±2.77	36.78±2.36	61.89±1.85	45.10±2.95	66.03±4.93
200.1025	Pyrimethanil	Benzenoids	15.75±0.94	26.68±1.00	3.538±0.115	3.611±0.021	16.99±0.53	19.28±1.32
373.1015	Quizalofop ethyl	Benzenoids	4.507±0.784	3.271±0.097	8.171±0.402	5.162±0.278	4.279±0.089	4.014±0.078
297.1172	Rac-didemethylcitalopram	Benzenoids	2.411±0.166	3.552±0.144	1.659±0.032	1.429±0.040	1.467±0.056	1.366±0.092
109.0295	Resorcinol	Benzenoids	8.975±0.685	15.42±1.53	6.126±0.421	9.382±0.466	9.618±0.676	9.814±0.636
283.0461	Rhein	Benzenoids	9.387±0.750	9.136±0.650	5.534±0.281	8.386±0.446	10.33±0.52	14.21±1.45
333.0999	Shikonin	Benzenoids	1.452±0.144	1.198±0.029	2.166±0.184	2.253±0.161	1.257±0.026	1.587±0.135
475.2152	Sildenafil	Benzenoids	1.377±0.216	4.171±0.310	0.3022±0.0036	1.065±0.066	1.546±0.148	1.732±0.048
209.0667	Sinapyl alcohol	Benzenoids	74.05±3.57	93.36±2.55	60.95±2.92	65.20±1.53	73.96±3.01	79.58±1.26
313.0299	Spirodiclofen	Benzenoids	2.971±0.069	1.531±0.093	2.386±0.036	3.693±0.036	3.166±0.283	3.785±0.137
374.2151	Spirotetramat	Benzenoids	1.213±0.225	1.357±0.033	0.7560±0.0195	1.159±0.059	1.158±0.037	1.425±0.066
387.1992	Sufentanyl	Benzenoids	1.182±0.136	4.780±0.105	0.9351±0.0490	1.439±0.116	1.740±0.047	1.883±0.079
279.1052	Sulfamethazine	Benzenoids	115.2±12.3	157.8±1.8	82.92±2.46	99.81±2.35	120.4±1.9	120.9±6.5

293.0285	Sulfamethizole	Benzenoids	8.569±0.587	11.46±1.16	6.257±0.260	8.858±0.151	8.988±0.501	8.623±0.235
405.1483	Sulfinpyrazone	Benzenoids	6.519±0.667	1.019±0.043	9.543±0.184	9.446±0.685	6.106±0.228	8.431±0.063
197.0455	Syringic acid	Benzenoids	48.88±4.59	90.78±6.13	27.35±1.57	51.19±1.39	57.17±3.27	55.56±3.97
380.9988	Triflumuron	Benzenoids	0.3105±0.0271	0.1965±0.0169	0.2730±0.0051	0.3624±0.0128	0.2187±0.0082	0.2524±0.0083
223.1443	Zectran	Benzenoids	2.626±0.107	0.04378±0.00111	0.2358±0.0199	0.2282±0.0100	10.42±1.23	0.9761±0.0407
573.1846	3'-o-desmethyletoposide	Lignans, neolignans and related compounds	6.305±0.288	7.711±0.312	3.554±0.170	5.703±0.270	5.411±0.398	3.409±0.140
399.0951	4'-demethylpodophyllotoxin	Lignans, neolignans and related compounds	22.30±0.49	32.53±1.30	6.544±0.294	21.03±0.37	19.64±1.70	16.83±1.01
297.1346	Enterolactone	Lignans, neolignans and related compounds	5.052±0.385	7.023±0.343	3.045±0.112	4.982±0.249	6.211±0.660	6.285±0.257
359.1500	Lariciresinol	Lignans, neolignans and related compounds	3.719±0.350	6.101±0.124	2.179±0.132	3.557±0.032	3.338±0.361	3.864±0.119
437.1269	Podofilox	Lignans, neolignans and related compounds	5.602±0.074	5.860±0.103	6.980±0.123	5.648±0.301	4.956±0.097	5.484±0.092
361.1868	Secoisolariciresinol	Lignans, neolignans and related compounds	1.009±0.094	1.866±0.029	0.6641±0.0698	0.8844±0.0441	0.9544±0.1046	1.143±0.034
383.0961	Teniposide	Lignans, neolignans and related compounds	2.099±0.083	2.944±0.213	1.289±0.051	2.181±0.048	1.767±0.118	2.213±0.090
245.1395	(+)-abscisic acid	Lipids and lipid-like molecules	16.64±0.82	34.72±1.18	10.89±0.14	14.59±0.22	17.02±0.87	18.04±1.18
233.1495	(+)-costunolide	Lipids and lipid-like molecules	10.67±0.70	25.13±1.80	6.910±0.518	10.26±0.30	11.87±0.46	11.20±0.30
488.2518	(2e,6e,11e,13e)-18-(2,6-dioxopiperidin-4-yl)-9-hydroxy-8-methoxy-10,12,14-trimethyl-15-oxooctadeca-2,6,11,13-tetraenoic acid	Lipids and lipid-like molecules	0.1141±0.0089	#NUM!±0.00	#NUM!±0.00	0.1793±0.0083	0.2071±0.0091	0.3405±0.0166
284.1496	(2r)-3-hydroxyisovaleroylcarnitine	Lipids and lipid-like molecules	2.114±0.106	3.569±0.191	1.797±0.046	1.907±0.107	2.136±0.061	1.966±0.153
267.1741	(5.alpha.)-androstane-3,11,17-trione	Lipids and lipid-like molecules	2.052±0.153	2.220±0.096	1.694±0.079	2.576±0.036	3.213±0.071	1.718±0.011
311.2229	(9z,12e)-15,16-dihydroxyoctadeca-9,12-dienoic acid	Lipids and lipid-like molecules	20.35±2.00	117.8±2.1	8.038±0.391	11.96±0.18	17.17±0.58	16.03±1.13
362.3265	(r)-(+)-arachidonyl-1'-hydroxy-2'-propylamide	Lipids and lipid-like molecules	5.277±0.307	3.916±0.082	1.690±0.067	9.088±0.228	1.804±0.160	6.902±0.214

173.0810	(r)-butyrylcarnitine	Lipids and lipid-like molecules	6.857±0.695	4.870±0.311	5.081±0.222	6.902±0.043	7.967±0.301	8.294±0.089
111.0805	.alpha.-cyperone	Lipids and lipid-like molecules	3.976±0.063	3.289±0.134	3.813±0.304	1.386±0.069	0.8463±0.0049	4.145±0.331
357.2497	.beta.-estradiol 17-valerate	Lipids and lipid-like molecules	66.68±2.15	110.5±5.1	30.68±0.54	63.54±3.93	77.62±4.47	80.12±1.03
359.2039	.beta.-estradiol 3-benzoate	Lipids and lipid-like molecules	2.189±0.115	2.069±0.124	1.805±0.035	2.445±0.058	2.222±0.127	2.193±0.022
195.0024	.beta.-glycerophosphate	Lipids and lipid-like molecules	5.839±0.343	5.766±0.327	6.399±0.138	5.988±0.394	5.669±0.358	5.848±0.191
697.4810	[1-hexadecanoyloxy-3-phosphonooxypropan-2-yl]octadec-9-enoate	Lipids and lipid-like molecules	19.13±3.00	11.04±0.51	7.192±0.178	6.736±0.063	7.903±0.246	8.665±0.061
655.1642	1-(1,2-dihexanoylphosphatidyl)inositol-4-phosphate	Lipids and lipid-like molecules	0.9507±0.0218	1.263±0.064	0.7364±0.0169	1.174±0.039	0.8170±0.0455	0.7481±0.0213
502.3292	1-(1z-hexadecenyl)-sn-glycero-3-phosphocholine	Lipids and lipid-like molecules	6.789±0.168	15.64±0.60	14.87±0.34	12.44±0.19	3.710±0.304	11.84±0.28
476.2782	1-(9z,12z-octadecadienoyl)-2-hydroxy-sn-glycero-3-phosphoethanolamine	Lipids and lipid-like molecules	130.8±1.6	308.4±14.1	120.0±6.2	95.36±2.50	95.35±4.19	101.0±6.2
830.5545	1,2-diarachidonoyl-sn-glycero-3-phosphocholine	Lipids and lipid-like molecules	32.04±1.94	43.91±2.14	31.40±0.67	31.54±0.74	38.55±1.89	27.50±0.59
401.2165	1,2-dihydrodesoxymetasone	Lipids and lipid-like molecules	1.960±0.123	7.729±0.370	0.8107±0.0500	1.843±0.070	2.322±0.120	2.478±0.165
688.4907	1,2-dipalmitoleoyl-sn-glycero-3-phosphoethanolamine	Lipids and lipid-like molecules	10.09±0.26	2.882±0.040	11.07±0.19	9.826±0.296	10.62±0.32	11.74±0.14
329.2336	11beta-hydroxyprogesterone	Lipids and lipid-like molecules	14.52±4.37	15.32±0.46	11.19±0.50	13.58±1.50	6.601±0.737	10.32±0.19
295.2280	12(13)-epoxy-9z-octadecenoic acid	Lipids and lipid-like molecules	11.48±1.85	41.28±1.91	7.661±0.638	9.117±0.404	8.591±0.300	8.876±0.360
317.1972	14,15-dihydroxy-5z,8z,11z,17z-eicosatetraenoic acid	Lipids and lipid-like molecules	1.181±0.065	2.056±0.043	0.6486±0.0454	1.134±0.054	1.191±0.136	1.472±0.092
371.2300	15(r),19(r)-hydroxyprostaglandin f1.alpha.	Lipids and lipid-like molecules	2.673±0.301	5.817±0.114	1.582±0.108	5.725±0.184	4.252±0.517	4.261±0.125
365.2366	15-cyclohexylpentanorprostaglandin f2.alpha.	Lipids and lipid-like molecules	9.831±0.235	11.41±0.36	9.609±0.484	10.15±0.45	8.070±0.212	9.599±0.602

339.2177	15-ketoiloprost	Lipids and lipid-like molecules	2.277±0.154	2.879±0.093	1.341±0.072	2.261±0.049	2.333±0.259	2.773±0.277
271.2279	16-hydroxyhexadecanoic acid	Lipids and lipid-like molecules	42.07±7.89	39.85±2.70	36.19±1.76	43.89±1.04	39.24±2.19	52.05±0.93
357.2135	16-phenyltetranorprostaglandin e1	Lipids and lipid-like molecules	10.82±2.98	14.18±0.61	6.021±0.175	10.13±0.98	10.15±0.36	15.95±1.07
429.3194	17.beta.-nandrolone decanoate	Lipids and lipid-like molecules	6.189±0.197	10.39±0.36	8.081±0.159	6.733±0.186	7.225±0.226	7.115±0.643
297.1675	17alpha-ethynylestradiol	Lipids and lipid-like molecules	7.072±0.545	17.11±1.51	5.932±0.411	8.816±0.537	5.977±0.194	7.913±0.671
331.2342	17alpha-hydroxyprogesterone	Lipids and lipid-like molecules	1.277±0.343	0.5421±0.0153	0.9606±0.0784	1.439±0.034	1.647±0.172	1.696±0.023
313.1760	17-oxobetamethasone	Lipids and lipid-like molecules	8.494±0.851	13.43±0.59	4.224±0.423	8.741±0.054	9.958±0.734	10.24±0.10
424.3061	17-phenyltrinorprostaglandin f2.alpha. cyclopropyl methyl amide	Lipids and lipid-like molecules	33.41±5.14	5.225±0.202	37.87±2.55	55.94±2.96	20.24±2.16	36.14±3.35
366.2367	17-phenyltrinorprostaglandin f2a methylamide	Lipids and lipid-like molecules	4.192±0.404	2.316±0.037	2.220±0.166	5.094±0.307	4.576±0.338	6.690±0.576
746.5947	1-hexadecyl-2-(9z-octadecenoyl)-sn-glycero-3-phosphocholine	Lipids and lipid-like molecules	8.856±0.583	10.07±0.30	8.665±0.268	9.031±0.207	9.511±0.653	7.807±0.324
608.4658	1-lignoceroyl-2-hydroxy-sn-glycero-3-phosphocholine	Lipids and lipid-like molecules	1.983±0.117	0.09495±0.00928	3.214±0.056	2.124±0.095	1.752±0.094	3.015±0.072
426.2619	1-myristoyl-2-hydroxy-sn-glycero-3-phosphoethanolamine	Lipids and lipid-like molecules	0.8123±0.0371	0.6684±0.0372	1.168±0.051	0.8857±0.0252	0.4738±0.0211	0.7676±0.0235
468.3087	1-myristoyl-sn-glycero-3-phosphocholine	Lipids and lipid-like molecules	7.751±0.573	3.324±0.320	13.95±0.44	9.621±0.197	3.942±0.357	8.798±0.168
784.5126	1-palmitoyl-2-arachidonoyl-sn-glycero-3-phosphoserine	Lipids and lipid-like molecules	37.77±0.39	17.28±1.20	39.43±0.70	38.79±1.11	36.43±2.22	41.67±0.87
454.2932	1-palmitoyl-2-hydroxy-sn-glycero-3-phosphoethanolamine	Lipids and lipid-like molecules	23.80±0.40	15.65±0.53	28.51±0.50	25.90±0.49	15.20±0.48	24.69±1.65
740.5224	1-palmitoyl-2-oleoyl-sn-glycero-3-phosphoethanolamine	Lipids and lipid-like molecules	440.9±8.5	152.4±2.8	467.3±10.6	427.4±13.5	450.2±14.8	478.8±25.8
496.3400	1-palmitoyl-sn-glycero-3-phosphocholine	Lipids and lipid-like molecules	101.3±6.5	64.89±4.25	116.9±2.2	111.8±2.7	83.01±5.47	128.2±3.3
482.3243	1-pentadecanoyl-sn-glycero-3-phosphocholine	Lipids and lipid-like molecules	22.15±1.47	11.57±0.37	31.78±1.08	26.46±0.50	14.01±1.61	23.91±1.58

546.3544	1-stearoyl-2-hydroxy-sn-glycero-3-phosphocholine	Lipids and lipid-like molecules	1.670±0.183	0.5798±0.0442	3.867±0.242	3.650±0.217	1.427±0.174	2.783±0.301
594.3770	2-(5-oxovaleryl)phosphatidylcholine	Lipids and lipid-like molecules	0.9106±0.0915	#NUM!±0.00	#NUM!±0.00	0.05134±0.00154	#NUM!±0.00	0.02421±0.00081
145.0506	2,2-Dimethylsuccinic acid	Lipids and lipid-like molecules	15.55±3.66	51.02±2.40	8.648±0.398	13.42±0.34	14.52±0.32	13.86±1.23
349.1835	2,3-dinor-8-isoprostaglandin-f2.alpha.	Lipids and lipid-like molecules	3.900±0.390	8.059±0.380	3.038±0.117	3.091±0.123	3.811±0.218	3.255±0.100
447.1621	21-carboxylic acid triamcinolone acetonide	Lipids and lipid-like molecules	0.3990±0.0303	0.6828±0.0468	0.2007±0.0162	0.3421±0.0100	0.3064±0.0131	0.2072±0.0171
175.0612	2-Isopropylmalic acid	Lipids and lipid-like molecules	26.13±4.87	130.5±8.3	7.735±0.750	21.75±1.88	24.46±0.80	19.46±1.23
716.5225	2-linoleoyl-1-palmitoyl-sn-glycero-3-phosphoethanolamine	Lipids and lipid-like molecules	80.05±1.42	24.45±0.72	85.79±1.55	80.76±2.85	84.12±3.12	93.15±2.08
117.0557	2-methyl-3-hydroxybutyric acid	Lipids and lipid-like molecules	10.99±0.63	11.80±0.69	2.763±0.099	5.871±0.381	7.962±0.702	7.755±0.754
268.1520	2-methylbutyryl-l-carnitine	Lipids and lipid-like molecules	7.236±0.938	7.766±0.626	5.491±0.361	6.932±0.163	7.168±0.854	7.681±0.099
762.5070	2-oleoyl-1-palmitoyl-sn-glycero-3-phosphoserine	Lipids and lipid-like molecules	7.651±0.403	6.244±0.114	7.407±0.263	10.48±0.41	9.614±0.287	8.456±0.100
431.3168	3.beta.,7.alpha.-dihydroxy-5-cholestenoic acid	Lipids and lipid-like molecules	0.4198±0.0585	0.5888±0.0094	0.6489±0.0235	0.2528±0.0110	0.9766±0.0341	0.2678±0.0204
577.2837	3-deacetylsalannin	Lipids and lipid-like molecules	0.7105±0.0829	1.647±0.066	0.5247±0.0340	0.9912±0.0114	0.5468±0.0179	0.6877±0.0797
630.3465	3-deoxyaconitine	Lipids and lipid-like molecules	0.5662±0.0711	1.019±0.072	0.1445±0.0058	0.3801±0.0097	0.7031±0.0081	0.9083±0.0643
270.1337	3-hydroxybutyrylcarnitine	Lipids and lipid-like molecules	13.69±0.62	23.01±1.34	9.674±0.224	12.14±0.19	12.62±0.62	13.75±1.26
543.2779	3-hydroxystanozolol glucuronide	Lipids and lipid-like molecules	0.4925±0.1427	4.153±0.075	0.2016±0.0191	0.5908±0.0297	0.6574±0.0185	0.7814±0.0551
145.0507	3-methylglutarylcarnitine	Lipids and lipid-like molecules	32.73±3.95	40.52±1.84	13.54±0.82	30.14±1.85	37.69±2.01	35.46±2.32
129.0557	4-acetylbutyrate	Lipids and lipid-like molecules	22.54±1.88	39.42±1.35	15.61±0.99	22.14±0.86	22.12±0.54	22.89±1.94
157.1336	4-hydroxynonenal	Lipids and lipid-like molecules	10.72±0.34	10.29±0.24	6.254±0.302	9.598±0.116	11.38±0.26	12.86±0.75
375.2244	5(s),14(r)-lipoxin b4	Lipids and lipid-like molecules	0.5487±0.0172	0.3783±0.0330	0.1612±0.0171	1.061±0.103	0.8218±0.0201	0.8095±0.0945

401.2148	5-[5-(acetyloxymethyl)-1,2,4a-trimethyl-7-oxo-3,4,8,8a-tetrahydro-2h-naphthalen-1-yl]-3-methylpentanoic acid	Lipids and lipid-like molecules	7.227±0.642	9.556±0.413	4.965±0.211	7.312±0.079	7.478±0.455	8.130±0.052
241.2038	5alpha-pregnan-3,20-dione	Lipids and lipid-like molecules	0.6723±0.1023	0.3181±0.0041	1.113±0.054	1.069±0.075	0.3524±0.0046	1.170±0.062
160.1333	5-aminovaleric acid betaine	Lipids and lipid-like molecules	22.68±1.03	31.52±1.67	17.36±0.30	26.11±2.72	21.91±1.70	24.74±1.48
251.1797	5-androsten-3.beta.,16.alpha.-diol-17-one	Lipids and lipid-like molecules	4.690±0.259	2.036±0.146	5.446±0.240	5.034±0.133	4.765±0.119	4.591±0.165
159.1130	5-androstene-3.beta.,17.beta.-diol	Lipids and lipid-like molecules	3.765±0.159	5.748±0.504	2.410±0.147	3.630±0.065	3.678±0.200	4.127±0.172
545.1580	5-heptenoic acid, 7-[(1r,2r,3r,5s)-3,5-dihydroxy-2-[(3r)-3-hydroxy-5-phenylpentyl]cyclopentyl]-, 4-(3-thioxo-3h-1,2-dithiol-5-yl)phenyl ester, (5z)-	Lipids and lipid-like molecules	3.770±0.226	3.812±0.082	2.090±0.045	3.634±0.086	3.170±0.047	3.706±0.132
431.1902	6.beta.-hydroxyeplerenone	Lipids and lipid-like molecules	0.2896±0.0592	0.1452±0.0077	#NUM!±0.00	0.1914±0.0113	0.2953±0.0034	0.6003±0.0186
297.2403	7,8-dehydropregnenolone	Lipids and lipid-like molecules	0.1617±0.0024	0.3977±0.0071	0.3371±0.0288	0.1630±0.0094	0.1241±0.0084	0.1323±0.0147
331.2280	9-deoxy-9-methyleneprostaglandin e2	Lipids and lipid-like molecules	17.73±0.86	9.116±0.160	16.95±1.35	10.10±0.14	15.90±0.54	13.34±0.95
293.2123	9-oxo-10(e),12(e)-octadecadienoic acid	Lipids and lipid-like molecules	12.35±3.67	29.08±1.64	5.996±0.597	7.539±0.104	8.290±0.683	8.770±0.757
204.1229	Acetylcarnitine	Lipids and lipid-like molecules	220.8±11.2	351.1±8.8	159.0±4.9	129.8±10.0	243.4±17.8	304.2±16.4
810.1338	Acetyl-coa	Lipids and lipid-like molecules	0.6955±0.0554	1.311±0.039	0.6231±0.0149	1.518±0.052	0.2102±0.0133	0.2860±0.0042
327.1781	Acitretin	Lipids and lipid-like molecules	5.887±0.493	17.95±0.61	2.593±0.025	6.806±0.148	5.360±0.228	6.891±0.644
559.1490	Acrinathrin	Lipids and lipid-like molecules	0.3110±0.0192	0.5402±0.0236	0.2667±0.0073	0.3063±0.0096	0.3182±0.0371	0.2743±0.0108
301.1760	Adrenosterone	Lipids and lipid-like molecules	7.414±0.307	12.54±0.54	2.761±0.048	5.867±0.123	7.757±0.544	7.429±0.086
371.1226	Ajugol	Lipids and lipid-like molecules	1.512±0.194	1.115±0.105	2.377±0.245	1.608±0.106	1.228±0.039	1.405±0.055

303.2001	Aleuritic acid	Lipids and lipid-like molecules	24.50±1.48	10.88±0.36	34.51±1.89	35.26±0.99	20.20±0.65	18.56±1.52
243.1342	All-trans-4-hydroxyretinoic acid	Lipids and lipid-like molecules	9.194±0.325	14.42±0.80	4.668±0.082	7.421±0.098	11.96±0.66	13.05±0.48
315.1879	All-trans-4-ketoretinoic acid	Lipids and lipid-like molecules	0.6458±0.1135	1.026±0.099	0.3621±0.0115	0.5726±0.0344	0.6282±0.0190	0.9323±0.0884
427.2560	Andrastin d	Lipids and lipid-like molecules	1.994±0.360	1.867±0.126	0.3958±0.0219	1.894±0.209	2.408±0.028	4.402±0.362
351.1263	Anisatin	Lipids and lipid-like molecules	51.53±2.54	60.79±2.48	35.83±1.75	50.79±2.08	47.85±3.20	64.35±5.10
305.1457	Artemisinin	Lipids and lipid-like molecules	6.383±0.660	8.058±0.517	2.639±0.180	5.734±0.307	7.917±0.246	9.881±0.823
255.1705	Beta-estradiol	Lipids and lipid-like molecules	1.077±0.051	0.3517±0.0240	0.4788±0.0245	1.841±0.030	1.887±0.108	0.8631±0.0279
411.2217	Betamethasone 9,11-epoxide 21-propionate	Lipids and lipid-like molecules	0.3132±0.0850	0.09691±0.002 78	0.05632±0.002 37	0.3039±0.0027	0.4348±0.0414	1.402±0.047
457.3514	Betulinic acid	Lipids and lipid-like molecules	6.505±0.871	10.08±0.18	6.103±0.428	7.109±0.042	5.127±0.147	5.697±0.322
393.3154	Bis(2-ethylhexyl) adipate	Lipids and lipid-like molecules	42.08±0.63	23.94±0.90	45.21±2.14	55.80±1.55	44.44±3.06	36.70±1.80
628.3626	Bulleyaconi cine a	Lipids and lipid-like molecules	0.3475±0.0674	1.589±0.022	0.1889±0.0050	0.1860±0.0053	0.3468±0.0040	0.3460±0.0189
405.1695	Chlormadinone acetate	Lipids and lipid-like molecules	1.079±0.043	4.894±0.230	#NUM!±0.00	#NUM!±0.00	1.438±0.115	0.7977±0.0289
465.3043	Cholesteryl sulfate	Lipids and lipid-like molecules	2.871±0.247	0.4531±0.0143	2.621±0.164	2.115±0.025	1.340±0.030	2.473±0.150
407.2955	Cholic acid	Lipids and lipid-like molecules	29.70±1.42	2.081±0.074	47.78±3.76	26.93±0.47	12.33±0.90	12.37±0.97
443.2251	Cinobufagin	Lipids and lipid-like molecules	132.7±14.4	398.0±5.5	78.33±2.72	120.7±5.1	156.7±10.2	163.8±1.5
327.2179	Cis-4,7,10,13,16,19-docosahexaenoic acid	Lipids and lipid-like molecules	21.39±1.05	32.88±2.74	5.179±0.262	12.71±0.73	17.47±0.86	40.74±0.55
129.0194	Citraconic acid	Lipids and lipid-like molecules	189.8±9.4	234.0±15.9	145.6±6.7	184.2±6.0	202.3±20.9	205.0±11.7
147.0299	Citramalate	Lipids and lipid-like molecules	18.80±0.42	17.77±0.81	15.19±0.77	18.56±1.13	20.63±1.35	83.39±6.66
501.1598	Clobetasone butyrate	Lipids and lipid-like molecules	1.549±0.203	2.474±0.055	0.9132±0.0687	1.221±0.072	1.387±0.071	1.435±0.066

541.1585	Cornuside	Lipids and lipid-like molecules	0.8318±0.1141	0.9113±0.0704	0.2691±0.0249	0.6645±0.0300	1.232±0.112	0.9431±0.0756
187.1437	Costunolide	Lipids and lipid-like molecules	20.71±1.60	31.36±2.22	6.107±0.130	10.20±0.65	27.02±2.94	31.68±0.84
149.0961	Cuminaldehyde	Lipids and lipid-like molecules	20.81±0.86	18.89±0.61	22.48±0.78	2.540±0.049	2.637±0.031	21.60±1.38
435.1110	Cypermethrin	Lipids and lipid-like molecules	0.7555±0.1187	1.134±0.053	0.6094±0.0468	0.6381±0.0512	0.7003±0.0130	0.8854±0.0309
531.4049	D-.alpha.-tocopherol succinate	Lipids and lipid-like molecules	2.960±0.125	3.504±0.187	2.254±0.069	1.861±0.099	2.523±0.031	2.712±0.130
413.2911	Delta4-dafachronic acid	Lipids and lipid-like molecules	0.4462±0.0273	0.6428±0.0312	0.2365±0.0151	0.3730±0.0308	0.3183±0.0265	0.3539±0.0115
395.2269	Deoxycorticosterone acetate	Lipids and lipid-like molecules	1.390±0.249	1.106±0.098	1.263±0.061	2.139±0.154	2.457±0.067	3.783±0.260
471.1355	Deoxynivalenol 3-glucuronide	Lipids and lipid-like molecules	2.296±0.048	2.839±0.093	1.701±0.043	2.184±0.045	2.347±0.035	2.269±0.039
293.2113	Desogestrel	Lipids and lipid-like molecules	1.162±0.169	1.419±0.014	0.4590±0.0340	1.285±0.051	0.3998±0.0352	0.4442±0.0151
206.1387	Dexpanthenol	Lipids and lipid-like molecules	3.296±0.247	3.317±0.229	1.368±0.118	0.3697±0.0123	15.78±0.70	0.5103±0.0145
312.1920	Dienogest	Lipids and lipid-like molecules	2.288±0.373	2.862±0.092	1.366±0.028	2.020±0.215	3.388±0.348	2.740±0.113
247.1653	Dinor-12-oxophytodienoic acid	Lipids and lipid-like molecules	60.13±1.91	77.91±3.42	38.45±0.78	56.56±1.67	62.25±3.97	63.17±0.87
177.1387	Dodecanedioic acid	Lipids and lipid-like molecules	2.090±0.184	4.252±0.084	1.384±0.120	2.112±0.083	3.467±0.083	2.245±0.058
198.1853	Dodecanoic acid, 12-[[(cyclohexylamino)carbonyl]amino]-	Lipids and lipid-like molecules	2.353±0.103	1.165±0.045	1.095±0.059	2.302±0.024	1.606±0.090	1.008±0.070
455.3274	Echinocystic acid	Lipids and lipid-like molecules	3.107±0.174	3.691±0.170	3.948±0.098	3.367±0.056	2.983±0.060	2.974±0.115
219.1341	Empenthrin	Lipids and lipid-like molecules	12.93±2.94	13.93±0.61	8.210±0.366	11.96±0.27	18.13±1.06	13.99±0.90
349.0946	Estrone sulfate	Lipids and lipid-like molecules	5.042±0.247	3.052±0.042	3.729±0.079	6.187±0.504	4.418±0.173	4.434±0.178
297.1675	Exemestane	Lipids and lipid-like molecules	2.026±0.343	3.977±0.077	0.9062±0.0657	1.888±0.131	1.591±0.060	2.647±0.277
423.1979	Fludrocortisone	Lipids and lipid-like molecules	2.614±0.416	0.4449±0.0044	0.8228±0.0211	1.382±0.065	3.240±0.065	5.716±0.076

417.1921	Flunisolide	Lipids and lipid-like molecules	0.1792±0.0234	0.2050±0.0116	0.07796±0.00221	0.1794±0.0094	0.2108±0.0119	0.3840±0.0067
403.2556	Gamabufotalin	Lipids and lipid-like molecules	1.798±0.103	2.659±0.089	0.7666±0.0744	1.552±0.026	2.327±0.092	2.090±0.206
601.3629	Garcinol	Lipids and lipid-like molecules	7.928±0.870	9.226±0.677	9.425±0.446	7.626±0.286	6.518±0.190	7.478±0.676
427.1329	Gardenoside	Lipids and lipid-like molecules	2.045±0.092	0.3402±0.0176	2.996±0.126	3.936±0.105	1.306±0.053	2.642±0.104
249.0945	Genipin	Lipids and lipid-like molecules	39.48±2.09	37.80±2.40	33.87±0.61	41.33±1.02	28.73±1.71	32.64±2.46
411.1073	Geniposide	Lipids and lipid-like molecules	7.449±0.276	6.695±0.150	8.894±0.327	4.985±0.081	7.656±0.404	7.722±0.134
219.1744	Germacrone	Lipids and lipid-like molecules	3.114±0.230	9.224±0.502	1.511±0.097	2.892±0.143	3.315±0.342	3.360±0.106
309.1675	Gestrinone	Lipids and lipid-like molecules	5.057±0.636	25.46±2.58	1.380±0.135	6.107±0.047	5.133±0.163	7.125±0.840
283.1267	Gibberellic acid	Lipids and lipid-like molecules	1.383±0.250	3.917±0.106	0.7548±0.0248	1.444±0.169	1.377±0.144	1.067±0.094
333.1772	Gibberellin a4	Lipids and lipid-like molecules	4.090±0.361	6.009±0.376	2.442±0.101	4.199±0.190	4.376±0.490	4.630±0.054
315.1675	Gibberellin a9	Lipids and lipid-like molecules	2.348±0.241	0.8112±0.0429	0.2130±0.0130	1.252±0.042	3.660±0.141	3.079±0.089
258.1101	Glycerophosphocholine	Lipids and lipid-like molecules	5455±204	5988±188	4589±89	5335±169	5628±377	5577±301
466.3028	Glycocholic acid	Lipids and lipid-like molecules	0.7265±0.0627	1.224±0.018	0.3277±0.0213	0.7211±0.0484	0.9722±0.0863	0.9999±0.0893
351.0545	Glycyrrhizic acid	Lipids and lipid-like molecules	0.5315±0.1146	0.4173±0.0061	0.4435±0.0294	0.5321±0.0245	0.5132±0.0064	0.5338±0.0349
362.0536	Guanosine 5'-monophosphate (GMP)	Lipids and lipid-like molecules	0.4675±0.0463	0.5418±0.0098	0.2872±0.0220	0.3435±0.0260	0.4741±0.0319	0.4839±0.0360
269.2486	Heptadecanoic acid	Lipids and lipid-like molecules	1.649±0.178	1.778±0.080	1.997±0.044	2.108±0.207	1.393±0.042	1.862±0.138
578.4142	Hexanamide, n-[(1s,2r)-2-hydroxy-1-(hydroxymethyl)heptadecyl]-6-[(7-nitro-2,1,3-benzoxadiazol-4-yl)amino]-	Lipids and lipid-like molecules	1.070±0.111	1.520±0.022	0.6828±0.0220	0.7049±0.0137	1.162±0.121	1.379±0.090
405.2097	hydrocortisone 21-acetate	Lipids and lipid-like molecules	1.277±0.070	1.330±0.021	1.047±0.023	1.331±0.018	1.253±0.090	1.523±0.048

221.0668	Isomaltose	Lipids and lipid-like molecules	28.66±1.36	45.53±2.25	15.90±0.89	23.97±0.42	30.73±2.07	32.77±0.80
101.0608	Isovaleric acid	Lipids and lipid-like molecules	8.327±0.210	11.10±0.49	5.826±0.264	8.715±0.088	8.488±0.720	8.230±0.407
187.0967	Isovaleryl-l-carnitine	Lipids and lipid-like molecules	12.32±0.35	16.48±1.02	8.235±0.156	11.80±0.14	13.03±0.69	13.87±0.28
129.0194	Itaconic acid	Lipids and lipid-like molecules	86.23±7.45	121.1±6.4	52.30±2.86	81.03±1.92	118.6±5.9	84.03±5.16
363.1490	Janerin	Lipids and lipid-like molecules	1.543±0.456	1.608±0.065	1.217±0.105	1.232±0.132	1.439±0.060	2.416±0.299
325.1312	Lactitol	Lipids and lipid-like molecules	27.21±1.44	26.00±0.97	35.31±1.75	28.17±0.85	24.75±2.08	27.56±0.92
339.1103	Lactobionic acid	Lipids and lipid-like molecules	4.051±0.240	2.389±0.069	5.189±0.368	4.550±0.113	3.174±0.039	4.358±0.053
495.2601	Leukotriene d4	Lipids and lipid-like molecules	1.079±0.087	7.080±0.187	0.7616±0.0450	1.559±0.024	1.137±0.123	1.167±0.111
513.6652	Linoleoyl coenzyme a	Lipids and lipid-like molecules	2.120±0.179	3.285±0.179	0.8961±0.0435	1.832±0.045	1.696±0.039	1.877±0.176
1028.3369	Linoleoyl-CoA	Lipids and lipid-like molecules	0.2646±0.0208	0.4858±0.0376	#NUM!±0.00	0.2090±0.0174	0.1925±0.0204	0.2426±0.0132
520.3399	Lpc 18:2	Lipids and lipid-like molecules	1300±84	3505±120	2855±258	821.7±40.3	710.1±68.9	2327±127
365.1056	Maltose	Lipids and lipid-like molecules	58.00±4.06	22.27±0.81	84.51±1.59	56.14±1.44	58.95±5.72	63.65±1.18
259.0459	Mesaconic acid	Lipids and lipid-like molecules	5.120±0.456	7.738±0.241	2.825±0.079	4.347±0.063	3.724±0.321	6.103±0.567
209.1536	Methyl dihydrojasmonate	Lipids and lipid-like molecules	1.946±0.095	1.368±0.060	1.248±0.109	3.830±0.066	1.472±0.094	1.115±0.115
225.1346	Methyl jasmonate	Lipids and lipid-like molecules	2.744±0.200	3.725±0.343	1.953±0.038	3.144±0.069	3.628±0.353	3.077±0.097
147.0663	Mevalonic acid	Lipids and lipid-like molecules	109.0±10.3	146.4±7.3	54.88±3.47	116.3±8.6	134.6±12.0	132.3±10.6
353.2689	Monolinolenin (9c,12c,15c)	Lipids and lipid-like molecules	2.337±0.291	2.621±0.034	3.004±0.039	4.772±0.178	3.204±0.156	2.072±0.156
406.1323	N-acetyl-d-lactosamine	Lipids and lipid-like molecules	3.163±0.076	1.737±0.026	5.802±0.134	2.960±0.054	2.756±0.133	3.394±0.198
307.0842	Neohesperidose	Lipids and lipid-like molecules	38.14±1.51	32.30±2.19	41.71±2.21	37.03±1.17	31.73±1.71	35.62±0.93

335.0952	Nivalenol	Lipids and lipid-like molecules	2.498±0.127	2.127±0.077	3.098±0.069	2.598±0.185	2.053±0.057	2.406±0.064
474.3789	N-nervonoyltaurine	Lipids and lipid-like molecules	71.45±2.80	94.30±6.31	91.37±1.60	87.14±3.54	50.00±2.42	58.99±1.10
477.1693	Obacunone	Lipids and lipid-like molecules	1.641±0.114	1.008±0.061	4.187±0.074	3.737±0.248	1.226±0.077	1.361±0.047
143.1077	Octanoic acid	Lipids and lipid-like molecules	2.490±0.349	6.343±0.254	1.618±0.122	1.979±0.149	1.633±0.129	2.065±0.182
288.2171	Octanoylcarnitine	Lipids and lipid-like molecules	528.1±20.5	692.0±43.8	354.3±11.7	520.3±15.4	554.3±43.5	589.5±33.1
297.2789	Oleic acid methyl ester	Lipids and lipid-like molecules	5.342±0.238	7.426±0.173	5.198±0.423	6.517±0.174	5.535±0.271	5.699±0.068
423.2583	Ophiobolin a	Lipids and lipid-like molecules	1.228±0.204	0.1194±0.0064	0.1702±0.0077	0.9042±0.0928	1.586±0.154	3.273±0.270
297.1530	Ostruthin	Lipids and lipid-like molecules	119.1±2.5	43.26±2.38	456.7±8.0	31.07±0.58	23.25±1.18	23.23±1.59
309.1295	Paclitaxel	Lipids and lipid-like molecules	12.22±0.90	2.999±0.285	2.174±0.090	2.486±0.064	29.49±1.50	11.96±0.83
703.5756	Palmitoyl sphingomyelin	Lipids and lipid-like molecules	18.86±2.13	1.920±0.112	1.259±0.102	1.783±0.114	1.096±0.119	1.372±0.128
206.1388	Pantothenol	Lipids and lipid-like molecules	118.7±6.7	194.0±11.0	75.61±1.93	104.8±4.4	125.5±6.0	136.1±2.6
249.1447	Parthenolide	Lipids and lipid-like molecules	14.11±1.11	18.00±1.09	9.974±0.268	14.74±0.34	14.17±1.30	15.04±0.90
788.5446	Pc 32:2	Lipids and lipid-like molecules	1.647±0.181	1.018±0.012	1.818±0.082	1.593±0.039	1.022±0.051	1.462±0.014
802.5600	Pc 33:2	Lipids and lipid-like molecules	3.881±0.209	1.968±0.157	4.430±0.214	4.066±0.045	3.516±0.353	3.757±0.171
816.5757	Pc 34:2	Lipids and lipid-like molecules	13.24±1.43	6.343±0.204	14.77±0.71	12.89±0.22	12.80±0.38	13.85±0.92
840.5758	Pc 36:4	Lipids and lipid-like molecules	172.1±23.6	128.3±3.6	253.0±15.1	192.2±13.5	156.3±13.8	156.1±4.8
856.5706	Pc(18:2/13-hode)	Lipids and lipid-like molecules	2.442±0.213	3.630±0.220	3.786±0.172	3.168±0.095	1.527±0.105	1.619±0.154
714.5078	Pe 34:2	Lipids and lipid-like molecules	105.8±4.7	23.99±1.61	123.5±5.7	105.7±4.6	107.6±7.6	122.9±1.8
742.5357	Pe 36:2	Lipids and lipid-like molecules	6.958±0.378	2.688±0.094	7.779±0.351	6.941±0.057	7.181±0.677	7.926±0.664

738.5080	Pe 36:4	Lipids and lipid-like molecules	747.6±58.3	179.7±9.2	818.1±43.6	714.1±28.3	684.5±27.7	755.9±45.7
730.5030	Pe(16:0/9-hode)	Lipids and lipid-like molecules	3.117±0.082	2.606±0.189	1.868±0.085	1.585±0.179	2.215±0.230	2.099±0.022
241.2174	Pentadecanoic acid	Lipids and lipid-like molecules	9.388±0.514	17.57±1.22	7.004±0.467	8.445±0.576	8.209±0.596	8.622±0.912
639.4085	Phorbol 12-myristate 13-acetate	Lipids and lipid-like molecules	1.236±0.197	4.310±0.409	0.9708±0.0940	1.563±0.019	1.057±0.107	1.232±0.036
833.5185	Pi 34:2	Lipids and lipid-like molecules	34.32±2.03	9.720±0.335	67.95±3.48	33.47±0.99	27.29±0.80	27.33±0.43
553.2965	Proscillaridin a	Lipids and lipid-like molecules	2.395±0.243	0.2954±0.0102	0.4662±0.0103	1.263±0.062	3.507±0.078	5.458±0.291
235.1188	Prostaglandin d1	Lipids and lipid-like molecules	7.818±0.395	3.705±0.084	7.338±0.473	8.400±0.274	7.272±0.215	6.237±0.142
373.1824	Prostaglandin d3	Lipids and lipid-like molecules	1.758±0.188	2.040±0.047	0.9854±0.0173	1.378±0.019	1.950±0.118	2.542±0.140
323.2583	Prostaglandin e1 alcohol	Lipids and lipid-like molecules	14.99±0.74	28.93±0.85	13.87±0.37	17.86±0.32	15.53±1.01	14.88±0.96
359.2405	Prostaglandin f2.alpha. 1,15-lactone	Lipids and lipid-like molecules	2.819±0.205	1.228±0.118	1.731±0.050	2.980±0.046	3.345±0.060	3.948±0.171
392.3312	Prostaglandin f2.alpha. diethylamide	Lipids and lipid-like molecules	34.75±2.50	7.903±0.704	23.58±1.19	43.35±1.83	44.69±3.51	39.69±2.16
351.2176	Prostaglandin i2	Lipids and lipid-like molecules	1.285±0.058	1.407±0.014	1.070±0.056	0.9436±0.0414	1.314±0.069	1.466±0.045
371.1195	Rutinose	Lipids and lipid-like molecules	7.414±0.987	8.594±0.162	4.718±0.496	6.891±0.092	7.698±0.224	6.644±0.094
201.1133	Sebacic acid	Lipids and lipid-like molecules	10.94±0.33	4.789±0.092	3.098±0.180	6.349±0.091	16.18±0.59	19.21±1.27
238.0452	Sn-glycerol-3-phosphoethanolamine	Lipids and lipid-like molecules	21.41±0.99	21.24±1.05	23.18±0.44	22.08±0.51	20.46±1.80	22.12±1.32
263.1240	Solstitialin a	Lipids and lipid-like molecules	18.64±1.96	25.58±0.27	11.26±0.49	16.90±0.42	20.23±1.89	19.80±0.82
143.0817	Succinic acid n,n-dimethylhydrazide	Lipids and lipid-like molecules	7.716±0.387	0.1924±0.0130	0.3419±0.0163	0.3738±0.0457	26.10±0.67	2.153±0.015
413.3053	Testosterone cypionate	Lipids and lipid-like molecules	6.937±0.441	4.935±0.460	9.030±0.301	6.425±0.058	10.73±0.62	8.266±0.185
349.2418	Tetrahydrocorticosterone	Lipids and lipid-like molecules	4.126±0.102	2.750±0.041	4.331±0.284	4.400±0.106	3.496±0.192	4.400±0.074

173.0922	Thymol	Lipids and lipid-like molecules	1.247±0.070	4.775±0.488	0.7496±0.0421	1.054±0.113	1.763±0.085	1.652±0.073
494.3090	Tiamulin	Lipids and lipid-like molecules	0.1716±0.0260	0.3013±0.0018	0.05452±0.00415	0.1905±0.0178	0.2366±0.0082	0.2428±0.0091
379.1502	Tiaprost	Lipids and lipid-like molecules	0.5708±0.0718	0.8204±0.0234	0.3462±0.0091	0.6059±0.0144	0.5752±0.0609	0.6921±0.0226
159.0299	Trans,trans-muconic acid	Lipids and lipid-like molecules	108.6±11.0	163.5±11.1	83.18±7.69	112.0±8.3	123.5±1.4	129.6±2.7
99.0087	Trans-2,3-dimethylacrylic acid	Lipids and lipid-like molecules	26.68±2.11	30.16±1.46	13.76±0.80	22.80±2.11	26.13±0.81	29.35±0.68
143.0350	Trans-2-butene-1,4-dicarboxylic acid	Lipids and lipid-like molecules	29.34±1.05	44.35±1.92	14.24±0.65	22.02±0.40	35.93±3.61	38.29±0.96
311.1468	Trans-crocetin	Lipids and lipid-like molecules	1.341±0.297	4.704±0.393	2.661±0.147	2.783±0.286	0.8295±0.0390	3.238±0.353
287.1866	Trans-dehydroandrosterone	Lipids and lipid-like molecules	65.66±4.26	80.78±4.00	39.82±1.40	62.30±2.49	58.97±5.06	69.02±1.44
227.1407	Trans-traumatic acid	Lipids and lipid-like molecules	41.39±1.70	54.68±1.92	22.23±1.03	30.35±0.56	37.95±2.39	54.13±1.99
195.0763	Verbenalin	Lipids and lipid-like molecules	49.49±3.06	84.87±4.87	24.09±1.06	45.95±1.21	61.19±4.22	61.57±1.82
369.0073	1-.beta.-d-arabinofuranosyluracil 5'-monophosphate	Nucleosides, nucleotides, and analogues	0.5824±0.0711	0.3877±0.0214	0.6204±0.0149	0.4027±0.0198	0.5947±0.0068	0.5724±0.0233
314.0640	2'-Deoxyadenosine 5'-monophosphate (dAMP)	Nucleosides, nucleotides, and analogues	0.1285±0.0223	#NUM!±0.00	#NUM!±0.00	0.3622±0.0433	0.5566±0.0351	0.3858±0.0337
505.1645	2'-deoxyinosine	Nucleosides, nucleotides, and analogues	2.762±0.196	3.123±0.146	3.175±0.029	3.226±0.104	2.060±0.101	2.738±0.082
426.0223	Adenosine 5'-diphosphate	Nucleosides, nucleotides, and analogues	4.771±0.222	7.482±0.558	2.231±0.100	5.226±0.128	5.444±0.580	4.719±0.084
560.0795	Adenosine 5'-diphosphoribose	Nucleosides, nucleotides, and analogues	2.166±0.042	0.1029±0.0001	3.697±0.079	6.061±0.114	0.5506±0.0068	1.431±0.045
370.0527	Adenosine 5'-monophosphate	Nucleosides, nucleotides, and analogues	4.370±0.279	2.821±0.231	3.570±0.158	4.551±0.200	4.412±0.417	5.144±0.176
346.0557	Adenosine 5'-phosphosulfate	Nucleosides, nucleotides, and analogues	53.57±0.90	28.60±0.89	21.90±1.12	47.92±1.18	69.60±3.87	82.39±0.81
462.0669	Adenylosuccinate	Nucleosides, nucleotides, and analogues	0.2171±0.0277	0.8736±0.0803	#NUM!±0.00	0.02845±0.00273	0.1595±0.0050	0.1944±0.0072
464.0819	Adenylosuccinic acid	Nucleosides, nucleotides, and analogues	0.2090±0.0252	0.9471±0.0109	#NUM!±0.00	0.05275±0.00154	0.1741±0.0113	0.1795±0.0039

558.0644	Adp-ribose	Nucleosides, nucleotides, and analogues	3.456±0.167	0.2528±0.0033	5.458±0.251	9.550±0.119	0.7768±0.0613	2.261±0.071
328.0531	Camp	Nucleosides, nucleotides, and analogues	0.8479±0.0795	1.108±0.052	0.4027±0.0424	0.7740±0.0608	0.9436±0.1034	0.9098±0.0274
304.0341	Cytidine 2',3'-cyclic phosphate	Nucleosides, nucleotides, and analogues	0.9398±0.0459	0.2886±0.0021	0.4064±0.0191	0.8441±0.0373	1.029±0.069	1.275±0.079
252.1092	Deoxyadenosine	Nucleosides, nucleotides, and analogues	13.24±1.27	13.14±0.76	2.704±0.092	7.292±0.117	23.04±2.66	30.82±0.87
321.0635	Deoxythymidine 5'-phosphate (dTMP)	Nucleosides, nucleotides, and analogues	10.22±0.08	10.17±0.23	10.78±0.13	10.23±0.24	10.04±0.48	9.450±0.058
588.0753	Gdp-l-fucose	Nucleosides, nucleotides, and analogues	0.7777±0.0269	0.4848±0.0165	0.1876±0.0093	0.3620±0.0116	1.287±0.058	1.529±0.054
264.0820	Gemcitabine	Nucleosides, nucleotides, and analogues	0.8899±0.0329	0.7347±0.0158	0.5496±0.0294	0.5304±0.0173	0.6908±0.0090	0.8948±0.0587
344.0401	Guanosine 3',5'-cyclic monophosphate	Nucleosides, nucleotides, and analogues	2.096±0.243	0.4630±0.0405	0.9144±0.0475	1.578±0.168	2.697±0.074	3.120±0.110
347.0444	Inosine 5'-monophosphate	Nucleosides, nucleotides, and analogues	0.8206±0.0295	1.446±0.017	0.4997±0.0281	0.8318±0.0954	0.9360±0.0342	0.9242±0.0278
290.0299	Nicotinate d-ribonucleotide	Nucleosides, nucleotides, and analogues	1.693±0.359	1.818±0.139	2.220±0.115	1.415±0.105	1.606±0.034	1.767±0.040
397.1159	S-Adenosylmethionine	Nucleosides, nucleotides, and analogues	3.475±0.228	4.075±0.199	2.617±0.169	3.319±0.097	2.576±0.121	2.402±0.258
298.0969	S-methyl-5'-thioadenosine	Nucleosides, nucleotides, and analogues	911.0±92.8	1259±79	629.7±34.8	1191±39	1061±35	1040±58
306.0332	Trp-Cys	Nucleosides, nucleotides, and analogues	2.383±0.339	2.350±0.076	1.544±0.032	2.309±0.027	2.610±0.080	3.147±0.093
535.0373	Udp-xylose	Nucleosides, nucleotides, and analogues	7.658±0.260	7.186±0.207	4.504±0.052	8.646±0.331	9.357±0.847	9.797±0.106
405.0096	Uridine 5'-diphosphate	Nucleosides, nucleotides, and analogues	32.38±4.01	51.65±3.31	22.24±0.46	30.20±0.57	31.88±1.51	28.78±0.34
809.0125	Uridine 5'-diphosphate (UDP)	Nucleosides, nucleotides, and analogues	4.870±0.825	11.87±0.42	2.765±0.039	6.981±0.534	4.021±0.197	4.828±0.061
589.0454	Uridine 5'-diphosphogalactose	Nucleosides, nucleotides, and analogues	4.078±0.291	4.789±0.323	4.202±0.079	4.406±0.068	3.336±0.025	3.579±0.109
606.0745	Uridine diphosphate-n-acetylglucosamine	Nucleosides, nucleotides, and analogues	1.884±0.293	1.979±0.106	1.088±0.095	5.488±0.320	2.011±0.165	2.103±0.177
211.0014	Xanthosine 5'-monophosphate	Nucleosides, nucleotides, and analogues	3.947±1.160	4.309±0.078	1.451±0.083	4.325±0.267	4.673±0.088	4.655±0.170

290.0859	Zidovudine	Nucleosides, nucleotides, and analogues	6.582±0.559	0.8287±0.0358	0.4186±0.0044	0.8382±0.0158	10.22±0.48	14.55±0.87
481.3502	(r)-aminocarnitine	Organic acids and derivatives	14.18±1.21	17.54±0.94	12.77±0.77	17.72±0.31	15.54±0.94	14.09±1.43
423.0433	.alpha.-carboxybenzylpenicillin	Organic acids and derivatives	3.656±0.189	4.275±0.282	2.933±0.071	3.300±0.103	3.804±0.383	3.566±0.132
408.1271	.alpha.-d-glucose pentaacetate	Organic acids and derivatives	2.395±0.176	3.022±0.164	1.524±0.056	2.091±0.037	2.176±0.079	2.685±0.129
102.0550	.alpha.-guanidinoglutaric acid	Organic acids and derivatives	26.13±1.20	25.71±1.03	18.84±0.30	29.92±1.74	29.07±1.90	30.65±0.66
203.0561	.alpha.-L-Glu-Gly	Organic acids and derivatives	51.92±2.58	52.72±2.38	51.82±3.87	64.03±1.60	61.50±2.97	55.12±1.59
263.0877	.alpha.-L-Glu-L-Asp	Organic acids and derivatives	6.107±0.497	7.437±0.260	2.041±0.089	5.249±0.397	9.597±0.555	7.003±0.149
86.0603	.gamma.-aminobutyric acid	Organic acids and derivatives	360.1±22.5	753.1±36.7	140.4±5.5	186.2±6.1	241.6±16.0	255.0±15.6
271.0825	.gamma.-glutamyl-(s)-allyl-l-cysteine	Organic acids and derivatives	20.53±0.62	29.98±2.02	15.09±1.10	18.29±0.32	22.39±1.75	20.45±1.56
209.0125	1,2,4-benzenetricarboxylic acid	Organic acids and derivatives	0.8204±0.0753	1.504±0.018	0.2585±0.0075	0.6613±0.0377	1.093±0.018	1.047±0.112
239.1756	12-hydroxydodecanoic acid	Organic acids and derivatives	1.328±0.054	2.047±0.120	0.8113±0.0048	1.138±0.020	1.862±0.161	1.381±0.066
170.0926	1-methyl-l-histidine	Organic acids and derivatives	32.52±1.61	41.65±2.41	16.20±0.40	30.83±0.57	29.56±3.11	34.68±1.08
131.0815	2-ketohexanoic acid	Organic acids and derivatives	49.66±5.45	62.14±4.84	35.73±0.85	68.69±7.80	50.12±4.54	50.08±0.65
194.1020	2-methyl-n-(4-methylphenyl)alanine	Organic acids and derivatives	28.52±0.69	28.67±1.05	29.02±0.56	30.98±0.72	26.63±0.78	25.76±1.55
365.0531	2s-amino-4-phosphonobutyric acid	Organic acids and derivatives	3.293±0.033	5.769±0.184	1.096±0.061	3.357±0.103	3.465±0.068	3.126±0.120
381.9605	3,3'-diiodo-l-thyronine	Organic acids and derivatives	0.4113±0.0355	0.2255±0.0104	0.1680±0.0086	0.3576±0.0343	0.5628±0.0101	0.6984±0.0294
264.1089	3,4-dihydroxycinnamic acid (l-alanine methyl ester) amide	Organic acids and derivatives	3.991±0.157	5.897±0.226	2.348±0.084	3.575±0.022	4.373±0.141	4.375±0.081
303.1814	3.beta.-hydroxymevastatin	Organic acids and derivatives	7.797±0.709	9.677±0.739	4.616±0.375	7.454±0.656	9.767±0.186	9.547±0.647
140.0707	3-amino-2,3-dihydrobenzoic acid	Organic acids and derivatives	63.70±4.92	119.1±7.3	29.41±1.53	61.67±1.59	65.96±3.32	80.59±1.97

116.1070	3-dehydrocarnitine	Organic acids and derivatives	1299±56	1855±127	1012±33	1314±39	1336±101	1382±74
189.0405	3-dehydroquinic acid	Organic acids and derivatives	495.5±49.8	907.1±59.6	267.4±9.2	502.8±31.4	578.1±17.8	543.0±30.2
117.0548	3-oxopentanoic acid	Organic acids and derivatives	6.083±0.379	7.801±0.367	4.230±0.042	6.193±0.084	6.787±0.427	5.913±0.259
339.0220	3-phosphonoalanine	Organic acids and derivatives	2.388±0.203	3.371±0.137	1.254±0.020	2.579±0.143	2.615±0.138	2.888±0.109
168.0657	4-acetamidobutanoate	Organic acids and derivatives	105.6±7.0	164.0±13.4	49.79±1.06	83.12±1.95	111.4±4.0	109.2±0.6
164.0705	4-hydroxy-l-glutamic acid	Organic acids and derivatives	145.7±5.4	156.3±9.2	122.1±3.2	169.0±5.0	170.1±11.1	164.9±2.1
148.0970	4-hydroxy-l-isoleucine	Organic acids and derivatives	53.16±3.26	81.05±7.05	57.56±0.59	52.53±2.64	50.78±4.22	57.65±5.53
348.0394	4-hydroxytriamterene sulfate	Organic acids and derivatives	1.576±0.144	#NUM!±0.00	0.2313±0.0211	2.557±0.128	3.044±0.304	3.205±0.211
114.0664	5-aminolevulinic acid	Organic acids and derivatives	30.41±3.17	53.38±3.92	13.18±1.10	24.92±1.12	31.75±0.81	29.35±0.42
116.0353	Acetylglycine	Organic acids and derivatives	9.669±0.802	11.01±0.69	5.015±0.280	9.014±0.295	13.03±0.70	11.11±0.60
244.1545	Agomelatine	Organic acids and derivatives	125.4±14.3	160.7±11.4	86.88±3.45	104.8±2.6	153.3±9.7	154.1±10.8
361.1648	Ala-Asp-Arg	Organic acids and derivatives	0.4052±0.0164	0.4906±0.0054	0.2060±0.0112	0.3433±0.0329	0.3459±0.0114	0.5901±0.0315
217.0718	Ala-Glu	Organic acids and derivatives	33.74±0.99	39.75±2.00	27.70±0.95	37.04±1.58	36.01±3.13	34.89±0.96
237.1237	Ala-phe	Organic acids and derivatives	5.522±0.441	4.110±0.048	1.644±0.060	4.090±0.322	7.965±0.233	7.817±0.280
276.1345	Ala-Trp	Organic acids and derivatives	4.824±0.369	4.362±0.100	2.351±0.123	4.048±0.193	5.752±0.163	7.458±0.385
148.0760	Albizziin	Organic acids and derivatives	4.278±0.415	7.013±0.429	1.966±0.164	4.552±0.144	4.379±0.455	5.085±0.067
221.0601	Allocystathionine	Organic acids and derivatives	1.397±0.126	1.702±0.030	1.437±0.132	1.658±0.141	1.439±0.176	1.563±0.142
145.0143	Alpha-ketoglutarate	Organic acids and derivatives	112.2±6.2	96.21±1.54	87.64±4.05	117.7±7.0	141.4±11.7	158.9±7.8
115.0401	Alpha-ketoisovaleric acid	Organic acids and derivatives	2.969±0.169	3.668±0.265	23.21±0.66	3.329±0.204	3.100±0.045	2.673±0.114

117.9968	Aminomalonic acid	Organic acids and derivatives	1.920±0.203	0.06721±0.00253	0.6596±0.0349	0.4411±0.0292	7.760±0.509	1.770±0.105
221.0305	Aminomethylphosphonic acid	Organic acids and derivatives	14.20±3.50	9.463±0.650	10.37±0.35	18.93±0.56	11.43±0.84	11.69±0.52
291.1300	Argininosuccinic acid	Organic acids and derivatives	55.94±4.74	59.65±3.06	31.14±1.40	49.51±1.35	71.96±7.85	64.84±0.76
286.1773	Arg-Leu	Organic acids and derivatives	8.718±2.222	4.177±0.326	2.440±0.092	6.454±0.272	9.260±0.260	13.31±0.26
302.1347	Asn-Gly-Asn	Organic acids and derivatives	0.2864±0.0377	0.2313±0.0165	0.1968±0.0187	0.3179±0.0280	0.3134±0.0146	0.3492±0.0278
293.1056	Aspartame	Organic acids and derivatives	2.159±0.363	2.494±0.247	1.516±0.137	1.467±0.032	1.690±0.091	4.037±0.340
271.1268	Asp-His	Organic acids and derivatives	1.040±0.296	0.7948±0.0142	0.5484±0.0040	1.084±0.044	1.145±0.106	1.334±0.039
393.1945	Asp-Met-Lys	Organic acids and derivatives	3.669±0.628	5.152±0.278	1.138±0.114	3.103±0.112	4.110±0.140	4.520±0.085
231.0977	Asp-Pro	Organic acids and derivatives	2.431±0.445	8.158±0.549	2.155±0.021	3.082±0.065	1.289±0.081	1.428±0.074
425.2148	Benazepril	Organic acids and derivatives	5.468±0.548	10.37±0.83	1.498±0.102	4.466±0.131	4.770±0.297	6.237±0.256
160.1081	Betonicine	Organic acids and derivatives	406.6±29.5	516.4±33.2	330.3±4.8	388.6±11.5	440.5±39.1	404.2±21.8
424.2171	Calpain inhibitor ii	Organic acids and derivatives	0.5314±0.0029	0.1329±0.0038	0.1331±0.0023	0.3702±0.0333	0.8154±0.0307	1.397±0.063
240.0656	Captopril	Organic acids and derivatives	0.2363±0.0102	0.01295±0.00072	0.3406±0.0231	0.5494±0.0286	0.1400±0.0009	0.2288±0.0058
225.0617	Chorismic acid	Organic acids and derivatives	35.50±1.85	47.63±2.21	26.16±1.22	37.38±1.39	34.93±3.92	37.52±0.43
339.1466	Cilastatin	Organic acids and derivatives	5.773±0.417	7.063±0.296	16.85±0.88	10.62±0.16	8.275±0.726	9.046±0.058
173.0092	Cis-aconitate	Organic acids and derivatives	230.3±12.9	286.9±21.2	174.6±2.4	225.8±7.9	246.8±27.4	252.3±14.3
191.0197	Citrate	Organic acids and derivatives	791.6±10.1	1095±75	716.3±74.2	791.4±23.2	754.2±33.7	801.1±42.9
357.1039	Cyclohexanesulfamic acid	Organic acids and derivatives	17.84±0.91	13.54±0.69	7.235±0.348	8.543±0.222	9.112±0.955	9.286±0.537
187.1079	Cyclopropanecarboxamide, n-[3-(2-methoxyethyl)-4,5-dimethyl-2(3h)-thiazolylidene]-	Organic acids and derivatives	1.679±0.196	2.217±0.190	1.235±0.064	2.067±0.143	2.664±0.223	2.265±0.020

	2,2,3,3-tetramethyl-, [n(z)]-							
248.0930	Cys-Gln	Organic acids and derivatives	8.828±0.557	1.197±0.014	7.525±0.267	11.49±0.57	9.038±0.410	10.70±0.15
441.2095	Cys-Tyr-Arg	Organic acids and derivatives	7.288±0.724	33.66±0.53	2.683±0.193	6.208±0.466	9.283±0.527	8.447±0.595
133.0609	D-asparagine	Organic acids and derivatives	41.64±0.80	42.70±2.65	25.69±0.43	38.71±1.15	41.08±2.30	44.99±0.62
178.0724	D-glucosaminic acid	Organic acids and derivatives	26.86±2.41	48.70±3.16	34.75±2.97	33.30±2.70	27.26±2.45	23.66±1.49
147.0763	D-glutamine	Organic acids and derivatives	1002±14	1068±37	872.0±6.1	988.4±30.5	971.6±31.3	986.8±52.9
305.1209	Diazinon	Organic acids and derivatives	39.49±5.99	55.85±3.85	34.42±2.69	67.09±1.93	53.09±3.08	65.55±2.42
312.9849	Dicloxacillin	Organic acids and derivatives	1.545±0.260	5.134±0.174	0.5645±0.0138	1.670±0.082	1.736±0.092	1.852±0.068
466.1539	Dihydrofolic acid	Organic acids and derivatives	0.2604±0.0282	0.3555±0.0030	0.3407±0.0052	0.3797±0.0225	0.2283±0.0158	0.2282±0.0100
175.1190	DL-arginine	Organic acids and derivatives	1163±82	1307±110	733.4±4.6	1109±44	913.1±41.9	1104±59
131.0462	DL-asparagine	Organic acids and derivatives	38.26±0.37	54.39±3.37	24.32±0.87	36.68±0.90	38.41±2.18	42.99±0.80
223.0747	Dl-cystathionine	Organic acids and derivatives	40.24±2.06	69.51±4.43	18.33±0.48	38.99±1.07	41.92±3.33	44.24±0.52
148.0605	DL-Glutamic acid	Organic acids and derivatives	300.9±9.9	221.8±3.0	216.2±3.2	312.7±13.2	349.8±17.1	363.5±19.9
132.1021	DL-isoleucine	Organic acids and derivatives	294.1±18.5	397.5±12.9	141.4±5.5	243.9±8.0	303.3±14.8	350.7±20.5
166.0865	DL-phenylalanine	Organic acids and derivatives	541.8±21.0	673.2±21.7	#NUM!±0.00	485.5±20.2	552.6±25.7	650.5±35.1
104.0357	DL-serine	Organic acids and derivatives	28.45±0.62	36.69±1.11	16.59±0.56	27.15±0.54	29.60±1.71	32.29±0.37
120.0656	DL-threonine	Organic acids and derivatives	47.48±1.75	65.69±5.18	35.13±0.75	50.52±2.85	45.11±1.09	47.87±0.55
182.0811	DL-tyrosine	Organic acids and derivatives	918.6±38.2	1229±70	600.9±15.4	873.7±32.7	988.1±57.0	991.8±53.3
118.0864	DL-valine	Organic acids and derivatives	144.0±2.4	178.8±2.7	103.2±4.8	125.7±4.2	151.0±1.9	167.8±3.5
130.0496	D-pyroglutamic acid	Organic acids and	19.98±1.92	4.048±0.102	32.70±1.00	24.01±1.45	11.80±0.37	11.39±0.48

		derivatives						
143.0818	Ectoine	Organic acids and derivatives	139.8±31.2	125.4±9.3	95.08±3.69	115.9±3.1	185.2±15.1	107.5±1.5
230.0957	Ergothioneine	Organic acids and derivatives	482.1±9.2	544.6±16.4	280.1±6.1	425.1±12.6	479.6±14.1	495.1±26.8
311.1155	Folate	Organic acids and derivatives	478.8±27.2	501.4±5.9	498.4±19.4	523.2±19.7	416.1±13.3	416.2±32.8
231.0147	Fumarate	Organic acids and derivatives	13.95±0.94	16.62±0.55	7.692±0.346	13.90±0.52	16.29±0.98	16.21±0.98
276.1191	Gamma-glu-glu	Organic acids and derivatives	83.57±5.33	37.01±2.10	36.54±0.91	68.71±3.11	124.0±12.1	129.6±1.0
279.1189	gamma-Glutamyl-L-methionine	Organic acids and derivatives	4.123±0.304	6.462±0.476	1.840±0.048	3.665±0.137	4.978±0.332	4.778±0.134
277.0896	Gamma-l-glutamyl-l-glutamic acid	Organic acids and derivatives	54.60±3.38	50.71±1.50	43.09±1.36	50.64±2.45	44.56±3.53	58.17±0.67
295.1291	gamma-L-Glutamyl-L-phenylalanine	Organic acids and derivatives	9.807±0.284	3.132±0.130	4.537±0.175	9.324±0.525	16.22±0.46	15.53±0.93
275.1352	Gln-gln	Organic acids and derivatives	6.473±0.046	14.97±0.47	2.611±0.042	4.603±0.137	4.944±0.212	4.480±0.062
304.1619	Glu-Arg	Organic acids and derivatives	6.585±0.862	1.076±0.047	1.527±0.017	5.041±0.375	13.39±1.55	13.16±0.22
182.0440	Glufosinate	Organic acids and derivatives	36.73±1.95	42.75±1.63	25.05±0.25	43.24±1.04	35.31±2.43	37.50±0.61
407.1891	Glu-Met-Lys	Organic acids and derivatives	0.2390±0.0151	0.06948±0.00497	0.1977±0.0092	0.5139±0.0143	0.4282±0.0090	0.3455±0.0278
391.1942	Glu-Ser-Arg	Organic acids and derivatives	1.511±0.158	1.860±0.055	0.9712±0.0447	1.591±0.061	1.621±0.097	1.715±0.062
613.1598	Glutathione, oxidized	Organic acids and derivatives	5.070±0.079	22.74±1.43	0.8162±0.0504	2.788±0.047	4.168±0.449	4.042±0.053
334.1401	Glu-Trp	Organic acids and derivatives	2.444±0.134	0.2474±0.0251	0.9790±0.0079	2.505±0.035	4.103±0.190	4.057±0.107
339.1998	Gly-His-Lys	Organic acids and derivatives	158.0±4.6	153.5±2.9	292.3±9.9	137.0±4.1	102.9±2.8	102.9±2.3
187.1089	Gly-Leu	Organic acids and derivatives	6.886±0.863	6.338±0.143	3.442±0.124	5.582±0.420	7.685±0.884	9.930±0.382
459.2199	Hc toxin	Organic acids and derivatives	1.500±0.349	9.466±0.938	1.035±0.070	1.103±0.056	1.886±0.178	1.799±0.112
293.1346	His-His	Organic acids and	1.773±0.230	1.984±0.050	0.5534±0.0204	1.144±0.135	4.246±0.494	1.774±0.104

		derivatives						
269.1610	His-Leu	Organic acids and derivatives	12.18±0.70	14.45±0.70	3.538±0.056	8.331±0.052	13.82±0.65	18.38±1.18
156.0769	Histidine	Organic acids and derivatives	419.6±16.0	427.4±19.9	385.5±5.3	412.2±12.9	375.5±25.7	462.0±54.2
205.0354	Homocitrate	Organic acids and derivatives	283.0±58.1	531.8±40.6	164.4±8.6	312.1±13.0	318.0±22.7	318.1±17.1
288.2032	Ile-Arg	Organic acids and derivatives	11.15±0.87	7.176±0.229	6.634±0.063	10.06±0.17	13.52±0.77	15.64±0.94
229.1546	Ile-Pro	Organic acids and derivatives	18.44±0.75	51.41±2.83	8.075±0.490	15.34±0.90	17.74±0.67	23.14±1.77
318.1815	Ile-Trp	Organic acids and derivatives	6.730±0.460	2.945±0.165	3.409±0.161	7.596±0.122	10.03±1.05	12.47±0.17
306.1298	Imazamox	Organic acids and derivatives	26.47±2.39	48.45±3.04	13.31±0.41	20.91±0.90	26.88±0.87	28.58±0.53
234.1086	Imazapyr	Organic acids and derivatives	20.79±0.83	27.52±0.86	16.45±0.35	20.35±0.70	19.91±1.11	19.73±1.21
212.0058	Indoxyl sulfate	Organic acids and derivatives	1.485±0.124	#NUM!±0.00	0.3981±0.0144	0.4371±0.0117	0.5355±0.0402	0.4881±0.0398
173.0092	Isocitrate	Organic acids and derivatives	20.38±3.60	43.81±3.11	14.69±0.43	17.09±0.47	38.07±2.79	18.24±1.12
173.0092	Isocitric acid	Organic acids and derivatives	37.14±0.73	37.97±2.46	30.14±0.65	43.98±1.10	43.11±2.38	36.63±0.98
707.2262	Jasplakinolide	Organic acids and derivatives	0.4682±0.0392	0.5355±0.0108	0.3231±0.0290	0.4767±0.0168	0.4045±0.0060	0.3957±0.0291
147.0299	L-2-hydroxyglutaric acid	Organic acids and derivatives	880.2±28.5	1231±44	595.1±15.9	868.7±29.2	970.8±87.3	934.1±50.3
188.0918	L-abrine	Organic acids and derivatives	9.964±0.654	23.98±2.30	6.196±0.249	10.17±0.24	13.13±0.90	10.42±0.06
177.1125	L-canavanine	Organic acids and derivatives	1.034±0.139	3.280±0.171	1.159±0.059	1.025±0.082	4.949±0.115	1.240±0.041
154.0510	L-carnosine	Organic acids and derivatives	4.995±0.276	11.36±0.34	3.171±0.144	4.681±0.077	4.532±0.389	4.998±0.359
176.1031	L-citrulline	Organic acids and derivatives	51.97±10.12	36.02±1.52	60.71±0.36	74.10±1.66	47.28±5.39	50.19±0.78
427.0956	L-cysteine-glutathione disulfide	Organic acids and derivatives	0.3313±0.0355	1.321±0.053	0.03398±0.00061	0.1265±0.0055	0.2551±0.0228	0.2409±0.0028
239.0196	L-cystine	Organic acids and	5.229±0.382	13.96±0.49	3.608±0.130	5.959±0.120	4.052±0.294	4.213±0.083

		derivatives						
203.1391	Leu-Ala	Organic acids and derivatives	18.39±2.64	15.01±0.84	4.700±0.430	12.96±0.49	25.30±2.38	37.10±2.97
401.2762	Leu-Ile-Arg	Organic acids and derivatives	1.516±0.076	2.187±0.141	0.5546±0.0435	1.958±0.203	1.512±0.077	2.326±0.014
401.2873	Leu-Leu-Arg	Organic acids and derivatives	3.292±0.476	3.227±0.057	1.321±0.117	3.713±0.128	3.215±0.066	3.086±0.228
409.3103	Leupeptin	Organic acids and derivatives	20.95±0.61	13.60±0.42	19.02±0.32	28.70±0.53	35.09±1.72	16.95±1.07
279.1705	Leu-Phe	Organic acids and derivatives	24.45±2.00	16.90±0.72	13.07±0.29	28.84±1.53	25.17±1.84	37.77±1.30
231.1705	Leu-Val	Organic acids and derivatives	72.07±7.09	44.83±3.07	30.72±2.15	66.58±4.15	78.27±0.92	140.6±3.7
272.0777	L-glutathione, reduced	Organic acids and derivatives	19.15±3.37	30.60±2.08	10.60±0.85	20.63±1.16	26.44±3.24	18.22±1.10
189.1346	L-homoarginine	Organic acids and derivatives	210.6±9.1	311.2±16.9	112.6±4.0	201.3±8.1	232.6±16.6	240.7±13.1
132.0125	L-homocystine	Organic acids and derivatives	14.66±1.31	0.4513±0.0116	0.1513±0.0123	0.5364±0.0320	53.15±3.23	6.707±0.187
405.2141	Lincomycin	Organic acids and derivatives	5.502±0.106	3.709±0.294	2.564±0.089	3.849±0.105	7.932±0.343	9.564±0.446
342.2398	Lisinopril (8r,s)-diketopiperazine	Organic acids and derivatives	7.607±0.456	5.829±0.244	5.061±0.454	8.224±0.069	7.580±0.640	8.879±0.115
229.1543	L-leucyl-l-proline	Organic acids and derivatives	137.6±3.1	201.5±7.1	94.56±1.70	133.8±4.1	149.8±6.3	149.2±3.7
319.1948	Lovastatin hydroxy acid	Organic acids and derivatives	0.9813±0.2394	0.6402±0.0191	1.434±0.070	0.9336±0.0808	0.6775±0.0287	1.010±0.036
128.0353	L-pyroglutamic acid	Organic acids and derivatives	1174±3	503.0±9.0	1194±41	1351±40	1229±36	1141±63
277.1395	L-saccharopine	Organic acids and derivatives	65.16±4.56	64.78±4.37	33.00±0.49	65.52±2.58	74.65±8.53	85.90±1.49
446.2362	L-serine, n-(1-oxohexadecyl)-o-phosphono-	Organic acids and derivatives	2.455±0.068	3.273±0.189	1.604±0.041	2.340±0.122	2.510±0.060	2.799±0.097
250.1286	Lys-Cys	Organic acids and derivatives	70.26±2.73	74.62±4.20	65.92±1.97	65.25±1.62	59.34±5.30	78.62±0.72
147.1129	Lysine	Organic acids and derivatives	52.87±2.26	82.93±5.66	26.62±1.09	49.89±1.18	58.92±6.21	65.99±0.76
260.1971	Lys-Leu	Organic acids and	5.060±0.221	9.660±0.332	2.566±0.017	4.525±0.044	4.950±0.480	5.895±0.167

		derivatives						
133.0142	Malate	Organic acids and derivatives	2583±167	3484±150	2163±98	2444±78	3023±349	2849±188
248.1494	Mefenoxam	Organic acids and derivatives	20.01±0.82	29.54±1.68	#NUM!±0.00	18.30±0.65	22.66±1.58	27.29±1.42
303.0529	Melphalan	Organic acids and derivatives	110.3±1.5	147.1±2.1	89.57±3.84	104.9±3.9	115.6±3.1	117.8±0.9
302.1348	Metalaxyl	Organic acids and derivatives	2.264±0.208	2.586±0.157	2.083±0.015	2.687±0.318	2.192±0.080	2.424±0.131
371.2278	Methyl (1-(cyclohexylmethyl)-1h-indole-3-carbonyl)-l-valinate	Organic acids and derivatives	5.502±0.430	6.823±0.505	4.731±0.231	4.295±0.097	4.033±0.245	4.850±0.063
469.2395	Met-Tyr-Arg	Organic acids and derivatives	0.1894±0.0095	0.08579±0.003 06	0.04005±0.002 23	0.05735±0.005 30	0.2873±0.0217	0.4565±0.0200
102.0550	N-(.beta.-ketocaproyl)-dl-homoserine lactone	Organic acids and derivatives	1.828±0.171	5.447±0.417	0.3729±0.0108	1.642±0.079	2.347±0.177	2.037±0.044
327.2015	N-(1-amino-3,3-dimethyl-1-oxobutan-2-yl)-1-pentyl-1h-indole-3-carboxamide	Organic acids and derivatives	13.77±1.02	17.17±1.62	10.67±0.47	10.63±0.24	11.03±0.87	12.26±0.58
189.1592	N,n,n-trimethyllysine	Organic acids and derivatives	1603±73	2338±171	868.0±7.2	1513±58	1762±196	1805±105
238.1076	N-.alpha.-(tert-butoxycarbonyl)-l-proline	Organic acids and derivatives	18.53±0.52	30.50±1.44	10.40±0.16	16.63±0.35	20.05±1.26	18.23±1.10
244.1545	N-3-hydroxyoctanoyl-l-homoserine lactone	Organic acids and derivatives	10.59±0.60	20.21±1.40	5.551±0.235	9.597±0.363	11.63±0.64	10.75±0.37
210.0986	N6-(1-iminoethyl)-l-lysine	Organic acids and derivatives	5.665±0.759	6.158±0.198	3.016±0.249	3.115±0.221	4.163±0.405	9.748±0.369
154.0976	N-acetylhistamine	Organic acids and derivatives	34.42±2.48	18.18±1.11	22.97±0.64	45.63±1.14	62.71±6.76	45.40±0.53
198.0891	N-acetylhistidine	Organic acids and derivatives	40.06±4.65	60.94±5.23	16.00±0.83	39.52±3.26	54.54±3.11	51.09±0.68
174.0408	N-acetyl-l-aspartic acid	Organic acids and derivatives	115.5±4.6	153.8±2.8	71.65±2.66	120.8±2.8	117.5±5.9	110.0±0.9
164.0717	N-acetyl-l-phenylalanine	Organic acids and derivatives	336.7±10.7	497.5±7.4	159.1±7.9	287.7±12.0	365.1±18.0	430.6±26.4
226.0936	N-acetyl-p-fluoro-dl-phenylalanine	Organic acids and derivatives	190.1±8.6	187.5±15.9	173.7±10.9	192.0±9.7	76.29±3.54	79.35±2.82
505.1416	N-acetyl-s-benzyl-l-cysteine	Organic acids and derivatives	0.8287±0.0633	1.397±0.074	0.4611±0.0184	0.8870±0.0237	0.9909±0.0578	0.9575±0.0694

316.1880	Nateglinide	Organic acids and derivatives	16.07±0.37	7.665±0.174	7.636±0.420	13.51±0.41	19.64±0.70	25.39±1.81
116.0706	N-benzyl-d-methionine methyl ester	Organic acids and derivatives	1.650±0.323	3.795±0.168	0.8230±0.0126	1.656±0.023	2.302±0.094	2.334±0.035
290.0883	N-fructosyl pyroglutamate	Organic acids and derivatives	8.765±0.067	1.841±0.007	1.241±0.098	1.729±0.073	27.16±1.93	11.17±0.10
203.1503	Ng,ng-dimethyl-l-arginine	Organic acids and derivatives	732.4±36.4	1134±77	379.5±10.5	681.6±28.1	792.2±63.2	819.0±44.3
200.1520	N-hexanoyl-l-homoserine lactone	Organic acids and derivatives	189.6±6.9	303.2±16.2	90.39±1.02	164.8±5.7	228.2±18.4	213.2±11.5
210.1338	N-octanoyl-l-homoserine lactone	Organic acids and derivatives	5.315±0.548	5.907±0.584	0.8554±0.0484	0.9999±0.0748	2.239±0.217	2.096±0.090
130.0146	Norleucine	Organic acids and derivatives	43.36±1.58	69.05±3.75	24.84±1.29	45.55±1.41	46.46±3.96	45.53±1.62
118.0863	Norvaline	Organic acids and derivatives	9.435±0.522	80.96±8.27	2.036±0.207	3.223±0.119	4.684±0.408	3.726±0.302
202.0585	O-succinyl-l-homoserine	Organic acids and derivatives	63.11±3.52	41.31±1.37	122.1±1.6	74.41±3.51	47.72±2.28	50.95±1.47
277.1228	Pantetheine	Organic acids and derivatives	33.44±2.19	120.2±8.3	0.5456±0.0397	0.5447±0.0095	41.98±2.00	24.23±1.84
577.2337	Pantethine	Organic acids and derivatives	0.8879±0.1219	0.3166±0.0087	0.9810±0.0755	0.8696±0.0179	0.7660±0.0780	0.9799±0.0320
202.1063	Pantothenic acid	Organic acids and derivatives	55.94±2.19	57.04±2.26	56.36±0.67	65.51±1.76	47.14±3.26	57.74±1.03
210.1126	Perifosine	Organic acids and derivatives	32.64±1.04	39.68±2.49	17.34±0.17	30.73±0.59	43.15±3.50	31.92±0.37
278.1148	Phe-asn	Organic acids and derivatives	1.097±0.004	0.6026±0.0043	0.4072±0.0143	0.7727±0.0148	1.646±0.092	2.148±0.068
294.1451	Phe-gln	Organic acids and derivatives	1.275±0.095	1.593±0.095	0.4228±0.0206	1.060±0.058	2.421±0.104	2.522±0.088
297.1270	Phe-met	Organic acids and derivatives	1.465±0.105	0.8858±0.0198	0.8149±0.0048	2.120±0.143	1.480±0.074	2.342±0.153
192.0667	Phenaceturic acid	Organic acids and derivatives	4.200±1.140	1.903±0.072	0.8871±0.0309	1.218±0.054	8.547±0.364	3.768±0.119
166.0864	Phenylalanine	Organic acids and derivatives	11.34±0.46	16.39±1.11	5.841±0.615	10.31±0.48	12.61±1.04	8.539±0.079
263.1604	Phe-pro	Organic acids and derivatives	4.126±0.220	6.324±0.501	2.676±0.038	4.109±0.041	5.478±0.531	4.489±0.054

381.2111	Phe-Ser-Lys	Organic acids and derivatives	1.767±0.377	0.4416±0.0303	0.4342±0.0084	1.385±0.045	2.074±0.024	3.110±0.212
267.1341	Phe-thr	Organic acids and derivatives	1.746±0.111	1.234±0.114	1.012±0.106	1.503±0.042	2.945±0.099	3.370±0.197
352.1657	Phe-trp	Organic acids and derivatives	1.690±0.049	0.3841±0.0288	0.7463±0.0270	1.836±0.034	1.832±0.056	2.448±0.138
228.0879	Pro-asn	Organic acids and derivatives	15.91±1.22	24.20±2.46	9.101±0.708	14.90±0.97	16.96±0.52	17.05±1.62
231.0978	Pro-Asp	Organic acids and derivatives	10.10±0.54	13.81±0.76	6.931±0.190	10.15±0.14	10.14±0.59	10.01±0.08
375.1992	Pro-Cys-Arg	Organic acids and derivatives	1.783±0.082	2.237±0.035	1.422±0.060	1.820±0.035	1.856±0.094	2.030±0.052
244.1296	Pro-gln	Organic acids and derivatives	11.46±0.18	9.648±0.232	8.390±0.059	12.36±0.46	11.09±0.95	13.71±0.18
208.1180	Propachlor oa	Organic acids and derivatives	349.6±15.6	378.1±13.6	301.9±10.7	398.7±12.3	355.9±26.8	322.2±18.4
263.1393	Pro-phe	Organic acids and derivatives	13.52±1.81	13.97±0.36	5.277±0.172	10.52±0.60	15.16±1.17	19.74±1.22
73.0295	Propionic acid	Organic acids and derivatives	85.89±3.18	132.1±3.2	47.05±2.26	76.86±2.72	89.16±7.32	96.29±1.21
213.1235	Pro-pro	Organic acids and derivatives	18.56±5.04	14.14±0.78	7.291±0.140	17.43±0.34	19.14±1.38	24.00±1.46
383.2042	Pyroglu-pro-arg	Organic acids and derivatives	2.926±0.125	2.683±0.044	2.013±0.081	3.076±0.019	3.458±0.301	3.768±0.193
359.1829	Pyroglu-thr-lys	Organic acids and derivatives	0.4282±0.0278	0.1030±0.0050	0.6351±0.0196	0.8164±0.0487	0.3375±0.0333	0.4003±0.0055
461.1982	Quinapril	Organic acids and derivatives	1.743±0.441	2.244±0.094	0.8749±0.0367	0.8360±0.0240	2.132±0.094	2.879±0.275
439.1095	Raltitrexed	Organic acids and derivatives	2.526±0.079	3.698±0.229	2.162±0.080	2.473±0.046	2.697±0.127	2.561±0.076
88.0404	Sarcosine	Organic acids and derivatives	149.5±6.5	315.2±3.7	43.28±1.65	85.67±4.17	148.3±8.3	182.1±9.9
177.0649	Ser-Ala	Organic acids and derivatives	12.88±0.48	14.90±0.94	9.026±0.400	10.65±0.23	7.920±0.237	9.664±0.752
251.1038	Ser-Phe	Organic acids and derivatives	14.46±0.27	9.256±0.176	6.240±0.456	10.15±0.13	18.04±1.04	24.76±1.90
378.0931	S-lactoylglutathione	Organic acids and derivatives	2.712±0.073	3.030±0.042	1.812±0.072	2.794±0.028	2.768±0.032	2.848±0.145

117.0193	Succinate	Organic acids and derivatives	416.7±15.6	635.7±22.1	223.4±11.4	363.8±13.2	431.3±39.4	444.7±23.9
124.0069	Taurine	Organic acids and derivatives	9.693±0.414	9.907±0.226	6.586±0.298	9.136±1.090	6.322±0.415	6.561±0.077
377.2034	Thr-Glu-Lys	Organic acids and derivatives	1.947±0.197	2.862±0.043	1.166±0.061	1.900±0.088	1.949±0.148	2.176±0.149
231.1351	Thr-Leu	Organic acids and derivatives	38.39±2.21	30.01±1.67	18.06±0.73	36.36±2.48	46.87±2.32	66.39±2.99
267.1108	Thr-Phe	Organic acids and derivatives	1.686±0.139	1.914±0.052	1.426±0.009	1.485±0.068	1.834±0.060	1.970±0.094
330.2037	Thr-Val-Leu	Organic acids and derivatives	6.945±0.701	4.144±0.182	3.239±0.121	6.165±0.128	10.12±0.18	11.46±0.53
346.1637	Thyrotropin-releasing hormone	Organic acids and derivatives	0.5205±0.0439	0.7843±0.0092	0.4370±0.0386	0.4746±0.0307	0.6079±0.0302	0.5701±0.0162
429.2507	Trandolapril	Organic acids and derivatives	0.5286±0.0036	0.4449±0.0171	0.5686±0.0228	1.282±0.020	0.3177±0.0058	0.5621±0.0473
129.0198	Trans-aconitic acid	Organic acids and derivatives	18.13±0.75	23.15±1.35	15.01±0.17	14.45±1.19	22.33±0.13	21.31±2.01
421.2426	Tris(2-butoxyethyl) phosphate	Organic acids and derivatives	0.6863±0.0271	#NUM!±0.00	0.1974±0.0145	0.5130±0.0641	1.249±0.063	1.522±0.098
302.1502	Trp-Pro	Organic acids and derivatives	3.665±0.168	15.80±1.59	1.091±0.081	3.036±0.306	3.886±0.234	4.830±0.123
247.1655	Tryptophan betaine	Organic acids and derivatives	16.57±0.83	23.59±0.87	10.89±0.11	15.31±0.27	17.35±1.07	17.33±1.04
295.1655	Tyr-Ile	Organic acids and derivatives	9.756±1.009	6.300±0.338	4.637±0.425	11.95±0.40	11.19±0.98	16.17±1.20
313.1009	Tyr-Met	Organic acids and derivatives	13.92±1.14	25.83±1.12	12.38±0.12	15.78±0.38	11.90±0.63	16.92±1.72
329.1499	Tyr-Phe	Organic acids and derivatives	1.070±0.168	0.4980±0.0095	0.3791±0.0101	1.105±0.081	1.555±0.023	1.929±0.100
231.0987	Val-Asp	Organic acids and derivatives	1.337±0.063	0.7354±0.0368	0.7099±0.0421	0.9652±0.0229	1.740±0.059	2.268±0.149
249.1269	Val-met	Organic acids and derivatives	4.518±0.628	3.384±0.281	1.813±0.130	4.556±0.251	5.758±0.192	8.469±0.660
181.0973	Val-Ser-Arg	Organic acids and derivatives	23.36±1.67	31.97±0.69	12.37±0.15	23.78±0.84	26.23±0.87	19.06±1.33
217.1548	Val-Val	Organic acids and derivatives	7.258±0.892	4.749±0.293	2.512±0.196	6.223±0.489	10.33±0.49	15.63±1.79

72.0810	1,2-diamino-2-methylpropane	Organic nitrogen compounds	89.34±1.29	112.3±2.0	54.16±0.36	78.28±1.98	94.53±1.30	106.2±3.0
116.1434	1-heptanamine	Organic nitrogen compounds	6.119±0.275	9.265±0.718	3.467±0.127	6.670±0.058	6.352±0.510	6.322±0.155
750.5494	1-palmitoyl-2-thiopalmitoyl phosphatidylcholine	Organic nitrogen compounds	30.43±0.71	29.69±2.54	26.59±1.42	28.85±1.81	26.70±1.23	21.93±1.59
154.1340	2,2'-azobis-2-methylpropanimidamide	Organic nitrogen compounds	62.79±0.95	57.99±2.67	62.07±0.48	71.98±2.31	61.14±3.84	69.15±0.98
210.1126	2,2-bis[hydroxymethyl]-2,2',2''-nitrilotriethanol	Organic nitrogen compounds	2.060±0.132	3.339±0.208	0.9285±0.0505	1.783±0.030	2.263±0.035	2.314±0.064
120.0808	2-amino-1-phenylethanol	Organic nitrogen compounds	282.8±10.7	360.2±6.4	156.3±3.1	251.2±8.8	290.7±12.5	345.4±18.8
88.0758	2-amino-2-methyl-1,3-propanediol	Organic nitrogen compounds	17.06±1.68	4.453±0.157	4.091±0.195	4.871±0.225	54.74±3.26	13.86±0.83
238.1286	3-(cyclohexylamino)-2-hydroxy-1-propanesulfonic acid	Organic nitrogen compounds	540.6±12.6	730.5±22.8	243.9±5.9	129.5±4.3	618.7±27.5	707.8±40.1
221.0188	4-hydroxycyclophosphamide	Organic nitrogen compounds	12.60±0.83	12.50±0.75	15.36±0.57	13.32±0.41	12.06±0.37	12.65±0.13
146.1176	Acetylcholine	Organic nitrogen compounds	2123±61	2150±105	1632±28	2035±78	2167±130	2086±118
587.0299	Carboxyphosphamide	Organic nitrogen compounds	10.69±0.96	12.75±0.91	11.07±0.51	11.30±0.43	9.457±0.404	9.758±0.223
162.1121	Carnitine	Organic nitrogen compounds	44.84±2.85	69.29±2.38	34.18±0.21	33.51±3.56	51.21±2.73	55.96±1.08
104.1071	Choline	Organic nitrogen compounds	2431±116	4586±364	1302±24	1877±66	1928±169	1887±102
172.0492	Crimidine	Organic nitrogen compounds	66.53±13.94	106.5±6.3	169.0±8.1	100.1±4.6	32.34±1.54	37.38±3.87
100.1121	Cyclohexylamine	Organic nitrogen compounds	8.112±0.662	7.277±0.459	10.27±0.85	11.53±0.57	6.557±0.757	7.616±0.484
253.1798	Dipyridamole	Organic nitrogen compounds	3.669±0.449	7.079±0.385	2.877±0.150	5.163±0.379	2.470±0.114	3.384±0.282
134.1176	Ethyldiethanolamine	Organic nitrogen compounds	3.057±0.076	4.197±0.188	0.9781±0.0440	1.089±0.136	3.938±0.279	4.206±0.081
162.1121	L-carnitine	Organic nitrogen compounds	2572±128	2760±146	2273±22	2514±75	2673±201	2550±139
262.1288	Methapyrilene	Organic nitrogen compounds	10.34±0.30	38.11±1.16	2.569±0.061	4.623±0.095	8.548±0.703	4.501±0.053

184.0733	Miltefosine	Organic nitrogen compounds	57.91±2.58	143.1±1.7	36.61±0.27	25.51±0.72	30.31±2.77	21.03±1.32
118.1227	N,n-diethyl-2-aminoethanol	Organic nitrogen compounds	3029±114	3352±223	2123±53	3020±89	3103±209	3270±190
206.1653	N1-(1-methyl-4-piperidinyl)-1,4-benzenediamine	Organic nitrogen compounds	2.054±0.165	#NUM!±0.00	0.1341±0.0136	0.2225±0.0080	7.919±0.466	0.01949±0.00181
516.3030	Oleyloxyethylphosphorylcholine	Organic nitrogen compounds	0.2280±0.0122	#NUM!±0.00	0.07411±0.00164	0.1722±0.0067	0.4075±0.0356	0.3899±0.0061
184.0728	Phosphorylcholine	Organic nitrogen compounds	62.34±1.98	67.78±2.14	51.28±0.63	58.05±1.50	63.06±3.72	66.31±1.62
318.3004	Phytosphingosine	Organic nitrogen compounds	20.52±0.73	11.90±0.35	10.26±0.54	17.11±0.32	29.41±1.52	38.59±2.48
210.0875	Porphobilinogen	Organic nitrogen compounds	1.613±0.204	1.349±0.054	0.5531±0.0217	2.282±0.048	1.674±0.074	2.256±0.070
282.2791	Sphingosine	Organic nitrogen compounds	2.234±0.019	1.132±0.099	1.375±0.052	1.514±0.105	1.804±0.053	2.068±0.019
104.0707	Tris(hydroxymethyl)aminomethane	Organic nitrogen compounds	39.75±2.17	46.64±3.57	29.15±1.17	41.34±1.31	46.51±2.29	45.33±0.53
212.1030	(-)-quebrachitol	Organic oxygen compounds	1.121±0.314	1.588±0.024	1.446±0.064	1.294±0.050	1.732±0.080	1.147±0.033
421.0754	.alpha.,.alpha.'-trehalose 6-phosphate	Organic oxygen compounds	4.651±0.182	9.040±0.199	1.479±0.079	2.950±0.049	5.460±0.581	5.936±0.090
380.1006	1-(1-2r-phosphothiotidyl)inositol-3,4-bisphosphate	Organic oxygen compounds	1.184±0.152	1.002±0.016	0.9792±0.0575	1.184±0.122	1.046±0.029	1.008±0.051
341.1088	1,4-d-xylobiose	Organic oxygen compounds	679.3±33.2	604.8±40.4	898.7±40.7	668.4±34.3	547.1±60.4	583.0±34.3
163.0612	1,5-anhydro-d-sorbitol	Organic oxygen compounds	39.40±2.80	71.07±2.43	25.39±1.89	33.08±1.34	45.90±3.33	44.79±0.53
196.0962	1-deoxy-1-(methylamino)-d-galactitol	Organic oxygen compounds	29.97±0.93	32.64±1.44	20.58±0.27	33.12±0.89	31.26±2.93	28.17±0.73
290.2076	1-heptanone, 1-(4-methoxyphenyl)-2-(1-pyrrolidinyl)-	Organic oxygen compounds	5.018±0.206	1.449±0.049	4.012±0.067	8.132±0.078	4.559±0.364	6.327±0.138
153.0759	2',4'-dihydroxyacetophenone	Organic oxygen compounds	3.012±0.164	6.727±0.299	2.739±0.079	4.126±0.195	2.960±0.039	2.876±0.103
117.0660	2-deoxy-d-ribose	Organic oxygen compounds	123.6±5.0	138.0±9.6	112.3±2.9	141.9±4.6	112.2±10.7	123.2±1.0

493.1671	2'-fucosyllactose	Organic oxygen compounds	0.4500±0.0151	0.6508±0.0275	0.3545±0.0276	0.5332±0.0130	0.3610±0.0244	0.4451±0.0088
99.0918	2-hexenal	Organic oxygen compounds	16.37±2.18	2.544±0.033	3.575±0.356	4.490±0.389	55.76±3.45	5.341±0.242
167.0928	2'-hydroxy-4'-methoxyacetophenone	Organic oxygen compounds	11.28±1.21	18.93±1.29	7.399±0.715	15.13±0.60	10.98±0.79	11.53±0.24
151.0519	3,4-dihydroxyacetophenone	Organic oxygen compounds	137.0±3.2	#NUM!±0.00	#NUM!±0.00	2.328±0.280	130.4±6.0	160.7±6.5
363.3106	3,6,9,12-tetraoxatetracosan-1-ol	Organic oxygen compounds	10.75±2.18	11.80±0.83	11.84±0.48	12.54±0.99	8.143±0.729	8.657±0.414
617.1323	3.alpha.,4.beta.-galactotriose	Organic oxygen compounds	0.2402±0.0081	0.5642±0.0388	0.2048±0.0092	0.3003±0.0211	0.2470±0.0112	0.2314±0.0286
535.1518	3-deoxy-d-glycero-d-galacto-2-nonulosonic acid	Organic oxygen compounds	2.343±0.138	6.737±0.596	0.7833±0.0353	1.806±0.109	2.578±0.248	1.659±0.025
208.0829	3-hydroxykynurenine	Organic oxygen compounds	88.43±10.13	164.9±2.1	52.20±5.42	148.9±4.4	83.55±1.00	84.36±2.79
195.0875	4',6'-dimethoxy-2'-hydroxyacetophenone	Organic oxygen compounds	19.88±5.81	35.66±2.68	9.563±0.557	19.41±0.99	14.92±0.29	20.06±2.02
377.0895	4.alpha.-mannobiose	Organic oxygen compounds	22.26±1.22	22.48±0.89	13.46±1.39	20.25±0.49	19.89±0.76	23.44±1.63
192.0504	4-aminophenyl-1-thio-.beta.-d-galactopyranoside	Organic oxygen compounds	10.97±0.51	14.66±0.81	6.333±0.196	11.25±0.17	9.923±0.236	11.69±0.42
117.1025	4-hydroxy-4-methyl-2-pentanone	Organic oxygen compounds	14.95±4.44	21.56±1.61	6.452±0.191	16.62±0.66	10.95±0.79	11.07±1.10
489.1695	5-azacytidine	Organic oxygen compounds	11.13±0.77	6.862±0.231	18.76±0.98	19.20±1.47	10.94±0.20	16.31±1.12
341.1791	9-phenyl-1-(2,4,6-trihydroxyphenyl)nonan-1-one	Organic oxygen compounds	14.50±0.74	11.34±0.57	35.40±1.60	11.88±0.31	13.01±0.90	9.498±0.079
347.0952	Acetohexamide	Organic oxygen compounds	2.336±0.147	2.665±0.206	2.286±0.080	2.503±0.021	2.112±0.025	6.518±0.539
161.0455	Alpha-D-Glucose	Organic oxygen compounds	69.03±2.75	128.0±6.7	42.32±3.17	57.57±1.45	73.57±1.87	71.44±3.48
410.1111	Altanserin	Organic oxygen compounds	18.38±1.03	20.57±1.05	10.16±0.48	19.03±0.60	17.29±1.09	18.18±1.12
453.1009	Bispyribac	Organic oxygen compounds	4.474±0.107	7.205±0.459	3.296±0.066	4.154±0.183	4.172±0.221	5.489±0.666
533.1724	Blood group b trisaccharide	Organic oxygen compounds	1.445±0.096	1.673±0.019	1.052±0.048	1.391±0.025	1.470±0.113	1.531±0.043

835.2689	B-pentasaccharide	Organic oxygen compounds	0.9225±0.0459	1.293±0.059	0.6795±0.0083	1.052±0.012	0.9081±0.0339	0.9001±0.0258
385.1313	Catalpol	Organic oxygen compounds	0.3344±0.0824	0.1245±0.0096	0.3639±0.0055	0.3144±0.0101	0.2403±0.0190	0.4332±0.0076
365.1062	Coniferin	Organic oxygen compounds	10.76±0.40	10.07±0.18	3.507±0.236	3.162±0.160	12.33±0.46	16.44±1.28
540.0540	Cyclic adenosine diphosphate ribose	Organic oxygen compounds	10.93±0.45	18.95±1.12	3.711±0.189	6.701±0.334	11.32±0.90	11.42±0.16
422.1062	Cyclosulfamuron	Organic oxygen compounds	3.013±0.298	2.042±0.029	2.924±0.139	2.823±0.230	3.048±0.125	3.507±0.206
101.0242	D-(-)-erythrose	Organic oxygen compounds	34.50±3.26	26.04±2.02	24.63±1.12	26.66±1.18	44.63±3.09	43.79±1.52
236.1495	D,l-n,n-didesmethyl-o-desmethylvenlafaxine	Organic oxygen compounds	5927±275	5772±286	5526±61	6116±182	6006±302	5707±306
165.0409	D-arabinonic acid	Organic oxygen compounds	2742±35	3498±151	1846±95	2562±81	2788±163	2943±159
133.0507	D-arabitol	Organic oxygen compounds	175.0±15.6	285.2±20.7	81.91±7.84	150.3±9.0	204.9±10.6	206.2±11.1
193.0352	D-galacturonic acid	Organic oxygen compounds	203.2±3.5	274.1±4.3	127.9±6.1	203.8±6.0	220.6±6.6	197.3±10.6
195.0510	D-gluconate	Organic oxygen compounds	370.5±74.5	529.0±22.3	353.7±18.1	335.3±17.1	289.5±25.8	394.4±21.2
177.0404	D-glucono-1,5-lactone	Organic oxygen compounds	106.4±8.6	189.1±11.9	101.1±6.7	119.2±3.1	111.3±10.3	91.00±0.52
283.0193	D-glucose 6-phosphate	Organic oxygen compounds	3.741±1.066	2.649±0.185	3.910±0.023	4.560±0.129	3.136±0.043	3.815±0.160
175.0248	D-glucuronic acid	Organic oxygen compounds	440.2±25.0	746.8±24.0	276.9±27.5	498.0±41.8	442.8±29.6	464.8±41.9
89.0241	Dihydroxyacetone	Organic oxygen compounds	147.2±33.3	175.5±5.6	118.0±5.3	93.42±2.81	145.4±11.9	111.4±0.7
181.0717	D-mannitol	Organic oxygen compounds	7710±247	8066±359	7431±342	7067±798	7309±448	6632±364
261.0286	D-mannitol 1-phosphate	Organic oxygen compounds	314.9±28.7	536.8±29.3	187.8±9.9	292.0±15.6	330.4±26.9	345.7±22.4
180.0688	D-mannosamine	Organic oxygen compounds	9.090±0.989	0.4765±0.0280	2.502±0.018	0.9139±0.0786	33.46±0.82	6.731±0.144
259.0130	D-mannose 6-phosphate	Organic oxygen compounds	21.62±0.69	65.47±2.22	10.45±0.48	20.78±1.45	19.31±1.19	24.11±2.22

161.0452	D-psicose	Organic oxygen compounds	21.56±2.08	24.84±0.87	15.50±0.72	21.01±0.79	19.51±0.77	20.71±2.48
229.0354	D-ribose 1-phosphate	Organic oxygen compounds	4.196±0.391	27.59±2.41	16.94±1.15	4.677±0.422	8.094±0.111	6.629±0.236
289.0331	D-Ribose 5-phosphate	Organic oxygen compounds	24.28±1.45	13.66±0.85	15.86±0.72	29.18±1.33	28.85±2.82	35.65±0.49
229.0340	D-ribulose 5-phosphate	Organic oxygen compounds	6.850±0.458	9.408±0.202	5.418±0.246	9.077±0.432	7.741±0.694	6.588±0.422
163.0612	D-sorbitol	Organic oxygen compounds	182.8±15.6	303.4±17.6	92.59±6.64	169.0±9.5	193.0±22.0	220.5±15.0
143.0350	D-Threitol	Organic oxygen compounds	5.515±0.738	8.241±0.740	6.150±0.277	5.796±0.038	5.956±0.734	5.706±0.069
113.0245	D-xylose	Organic oxygen compounds	29.98±3.00	37.81±1.52	34.37±1.56	38.08±0.94	27.18±1.75	28.95±0.49
293.1792	Embelin	Organic oxygen compounds	200.2±3.1	239.1±2.8	291.6±13.3	192.8±6.1	138.7±4.7	171.9±4.1
125.0711	Ethanone, 1-[2-hydroxy-3-propyl-4-[4-(2h-tetrazol-5-yl)butoxy]phenyl]-	Organic oxygen compounds	4.120±0.467	8.433±0.358	3.083±0.037	3.517±0.075	4.044±0.241	3.586±0.274
461.1512	Forsythoside e	Organic oxygen compounds	0.4882±0.0537	0.8042±0.0390	0.2213±0.0156	0.5863±0.0115	0.5649±0.0554	0.5518±0.0245
339.0042	Fructose 1,6-diphosphate	Organic oxygen compounds	0.8376±0.0760	0.3248±0.0053	0.9551±0.0432	1.110±0.043	1.085±0.082	0.7763±0.0506
401.1300	Galactinol	Organic oxygen compounds	706.6±40.1	625.8±39.4	895.8±40.3	695.2±35.2	571.3±55.8	607.1±35.1
338.1816	Geneticin	Organic oxygen compounds	0.3392±0.0258	0.4953±0.0483	0.1774±0.0015	0.3252±0.0092	0.3541±0.0097	0.3015±0.0123
105.0194	Glyceric acid	Organic oxygen compounds	132.8±17.3	257.1±5.8	97.34±4.71	120.7±8.1	133.2±2.5	148.2±5.6
432.1320	Hdmboa + o-hex	Organic oxygen compounds	0.9318±0.0734	1.276±0.044	0.6171±0.0301	0.7915±0.0159	0.7124±0.0833	1.018±0.076
309.1813	Heptaethylene glycol	Organic oxygen compounds	1.132±0.042	1.334±0.069	0.7459±0.0479	1.146±0.074	1.535±0.113	1.358±0.046
283.1752	Hexaethylene glycol	Organic oxygen compounds	26.87±1.09	30.10±1.08	19.81±1.28	21.47±0.44	20.47±1.79	22.52±1.43
296.1220	Indican	Organic oxygen compounds	5.746±0.407	4.939±0.192	5.796±0.057	6.529±0.038	4.585±0.112	6.506±0.509
101.0244	L-(+)-erythrulose	Organic oxygen compounds	209.2±5.5	221.2±9.2	199.9±9.8	161.9±5.9	189.5±9.3	181.1±9.7

647.1854	Laminaritetraose	Organic oxygen compounds	3.640±0.623	4.410±0.169	1.635±0.157	4.603±0.289	2.396±0.053	1.720±0.062
151.0613	L-arabinitol	Organic oxygen compounds	40.81±3.81	66.11±3.11	28.52±2.46	38.55±2.13	48.44±2.42	49.02±1.81
637.1533	Leiocarposide	Organic oxygen compounds	0.3112±0.0458	0.09020±0.007 12	0.8274±0.0156	0.2544±0.0287	0.1864±0.0140	0.2107±0.0158
165.0761	L-iditol	Organic oxygen compounds	1.278±0.094	1.579±0.049	1.507±0.025	1.319±0.018	1.598±0.048	1.104±0.129
892.2910	Lnfp iii	Organic oxygen compounds	0.9019±0.0200	0.8908±0.0481	1.305±0.046	1.082±0.021	0.7567±0.0770	0.8352±0.0569
746.2126	Lnnt	Organic oxygen compounds	0.4491±0.0368	0.8881±0.0811	0.2232±0.0014	0.5164±0.0196	0.4797±0.0423	0.5241±0.0128
730.2385	Lnt	Organic oxygen compounds	4.918±0.410	6.868±0.437	4.199±0.064	5.783±0.240	4.459±0.543	4.977±0.122
135.0299	L-threonate	Organic oxygen compounds	1473±91	1963±104	962.9±53.7	1396±61	1653±112	1519±82
527.1586	Maltotriose	Organic oxygen compounds	10.19±0.65	12.33±0.66	10.71±0.24	11.88±0.21	9.865±0.751	9.393±0.195
402.1673	Methanesulfonamide, n-[4-[[1-[2-(6-methyl-2-pyridinyl)ethyl]-4-piperidinyl]carbonyl]phenyl]-	Organic oxygen compounds	2.482±0.114	1.580±0.089	3.773±0.058	2.849±0.146	2.056±0.063	2.420±0.116
367.1051	Methyl chlorogenate	Organic oxygen compounds	12.89±0.33	20.12±1.49	10.73±0.42	11.80±0.26	10.57±0.28	11.81±0.80
117.0557	Methyl-2-hydroxyisobutyric acid	Organic oxygen compounds	9.472±0.665	18.79±1.86	5.781±0.259	9.524±1.168	12.10±0.41	10.28±0.10
252.1234	Muramic acid	Organic oxygen compounds	10.56±2.30	13.88±0.75	6.447±0.184	10.53±0.13	13.13±1.26	11.85±0.46
179.0563	Myo-inositol	Organic oxygen compounds	1071±33	1693±81	514.6±24.8	890.4±96.1	1163±80	1358±73
447.1588	N,n'-diacetylchitobiose	Organic oxygen compounds	10.56±0.21	0.9065±0.0434	6.471±0.048	8.411±0.125	13.73±0.62	20.01±1.22
186.0762	N-acetyl-.beta.-d-mannosamine	Organic oxygen compounds	25.56±0.12	31.78±0.93	20.01±0.15	28.31±0.51	24.02±0.77	25.60±1.55
222.0984	N-acetyl-d-galactosaminitol	Organic oxygen compounds	53.70±3.95	109.3±6.3	33.84±3.22	52.24±2.80	54.77±2.80	56.39±2.76
204.0980	N-acetyl-d-glucosamine	Organic oxygen compounds	110.3±22.0	100.3±1.8	73.65±1.76	99.70±2.68	89.74±3.32	88.82±1.71
300.0395	N-acetyl-d-glucosamine 6-phosphate	Organic oxygen compounds	5.789±0.204	6.714±0.118	5.089±0.313	6.017±0.110	5.890±0.278	5.862±0.104

465.1696	N-acetylglucosamine	Organic oxygen compounds	2.336±0.131	0.4625±0.0143	1.947±0.050	1.873±0.137	2.070±0.069	5.690±0.285
310.1149	N-acetylneuraminic acid	Organic oxygen compounds	10.89±0.79	15.15±0.53	7.831±0.186	10.63±0.14	9.576±0.610	10.45±0.06
191.0560	Neochlorogenic acid	Organic oxygen compounds	27.86±2.37	30.87±0.91	17.95±0.80	25.61±1.48	46.64±3.02	27.69±0.65
638.2390	Neohesperidose heptaacetate	Organic oxygen compounds	0.1756±0.0244	0.2140±0.0107	0.2217±0.0024	0.3020±0.0110	0.1560±0.0138	0.1453±0.0089
251.0776	Orcinol .beta.-d-glucoside	Organic oxygen compounds	6.785±0.313	24.30±2.37	3.051±0.109	6.833±0.192	6.889±0.423	7.751±0.046
218.1035	Pantothenate	Organic oxygen compounds	1199±79	1023±72	587.7±42.5	593.4±34.4	1294±39	1398±111
239.1484	Pentaethylene glycol	Organic oxygen compounds	35.95±1.25	58.39±5.55	26.95±0.60	31.01±0.93	29.82±1.70	35.02±1.46
235.0717	Perseitol	Organic oxygen compounds	0.8965±0.0138	1.186±0.120	0.7076±0.0275	1.389±0.017	0.9479±0.0469	1.231±0.142
499.1645	Primeverin	Organic oxygen compounds	0.4196±0.0915	0.1147±0.0064	0.6137±0.0264	0.3728±0.0228	0.4877±0.0111	0.5588±0.0258
318.1164	Prunasin	Organic oxygen compounds	1.220±0.130	0.07341±0.00245	0.08803±0.00051	0.1469±0.0049	4.373±0.476	0.5849±0.0612
454.1600	Pseurotin a	Organic oxygen compounds	0.5198±0.0356	0.7465±0.0214	0.2739±0.0104	0.6053±0.0240	0.5468±0.0085	0.6815±0.0251
527.1592	Raffinose	Organic oxygen compounds	3.322±0.276	4.003±0.150	4.116±0.239	3.022±0.054	2.418±0.257	2.597±0.184
483.0694	Regorafenib (bay 73-4506)	Organic oxygen compounds	0.3884±0.0066	0.2145±0.0061	0.4593±0.0054	0.4917±0.0103	0.3585±0.0259	0.3238±0.0184
309.1159	Salicin	Organic oxygen compounds	16.30±0.93	14.90±0.66	12.91±0.16	11.10±0.39	15.59±0.69	19.31±1.15
111.0451	Shikimate	Organic oxygen compounds	31.19±0.70	45.72±1.63	19.00±0.83	34.28±1.08	28.92±1.94	33.02±0.82
261.0381	Sorbitol 6-phosphate	Organic oxygen compounds	9.563±2.768	8.699±0.467	4.521±0.107	6.180±0.168	7.771±0.231	7.132±0.083
665.2149	Stachyose	Organic oxygen compounds	0.8772±0.1221	1.059±0.039	0.7689±0.0427	0.8833±0.0246	0.6070±0.0133	0.7874±0.0306
195.1227	Tetraethylene glycol	Organic oxygen compounds	20.56±0.70	65.47±5.85	15.26±0.54	18.28±0.95	16.85±1.40	17.63±1.22
258.1313	Tolmetin	Organic oxygen compounds	16.58±0.59	12.66±0.44	14.03±0.21	20.38±0.84	15.63±0.48	16.17±0.97

367.1050	Trans-3'-hydroxycotinine o-.beta.-d-glucuronide	Organic oxygen compounds	29.76±2.54	35.85±1.06	12.23±0.57	19.68±1.01	32.80±2.82	42.73±4.92
683.2250	Trehalose	Organic oxygen compounds	127.8±7.1	114.9±11.2	174.4±8.8	127.1±7.1	103.1±9.9	108.3±4.1
151.0965	Triethylene glycol monobutyl ether	Organic oxygen compounds	2.645±0.118	5.721±0.159	2.138±0.128	2.025±0.219	2.758±0.042	2.214±0.013
392.1530	Trifluperidol	Organic oxygen compounds	0.4420±0.0598	0.3030±0.0071	0.6341±0.0100	0.3594±0.0162	0.2235±0.0095	0.3913±0.0209
521.1385	Verproside	Organic oxygen compounds	1.936±0.167	2.984±0.066	1.074±0.014	2.284±0.027	1.600±0.050	1.987±0.169
211.0825	Volemitol	Organic oxygen compounds	24.44±2.76	22.93±1.20	22.24±1.12	26.32±2.01	19.55±0.73	19.49±1.19
151.0612	Xylitol	Organic oxygen compounds	3582±57	3632±125	3364±156	3825±117	3200±111	3192±171
411.1315	(-)-riboflavin	Organoheterocyclic compounds	1.689±0.185	1.763±0.078	1.453±0.051	1.599±0.089	1.701±0.185	1.419±0.107
275.1234	(+)-chlorpheniramine	Organoheterocyclic compounds	6.333±0.292	6.605±0.485	5.298±0.172	6.360±0.250	6.858±0.605	6.325±0.094
235.0967	(2r)-5-methoxy-2-methyl-2,3,8,9-tetrahydrofuro[2,3-h]chromen-4-one	Organoheterocyclic compounds	1.133±0.160	1.247±0.019	1.042±0.036	1.036±0.094	1.352±0.166	1.474±0.056
189.1236	.alpha.-ethyltryptamine	Organoheterocyclic compounds	104.9±11.0	5.798±0.238	172.9±6.2	126.5±8.3	58.62±3.51	58.76±1.34
204.0689	1-(2,8-dihydroxyquinolin-5-yl)ethan-1-one	Organoheterocyclic compounds	6.077±0.741	29.36±0.75	1.975±0.130	5.783±0.451	9.150±0.311	6.950±0.461
197.0913	1-(3-chlorophenyl)piperazine	Organoheterocyclic compounds	18.84±0.69	26.37±0.94	13.15±0.17	18.47±0.53	18.31±0.77	20.73±1.24
217.1547	1-(4-piperidinyl)-1,3-dihydro-2h-indol-2-one	Organoheterocyclic compounds	121.6±3.0	157.9±3.1	79.89±1.87	122.3±3.8	138.8±4.6	129.6±2.9
195.0675	1,7-dimethyluric acid	Organoheterocyclic compounds	4.432±0.324	4.823±0.085	3.559±0.261	3.805±0.117	3.372±0.109	4.372±0.231
284.3313	15-deoxy-goyazensolide	Organoheterocyclic compounds	13.34±2.22	7.680±0.494	16.92±0.46	17.77±0.36	13.73±1.15	9.139±0.292
143.1181	1-acetyl-3-piperidinamine	Organoheterocyclic compounds	34.71±1.63	48.11±3.29	25.79±0.42	36.83±0.92	33.85±3.52	35.19±0.41
111.0554	1-acetylimidazole	Organoheterocyclic compounds	2.018±0.278	1.594±0.133	3.593±0.073	1.536±0.018	2.561±0.040	2.284±0.135
297.1069	1h-indol-6-ol, 1-(4-methoxyphenyl)-2-methyl-3-	Organoheterocyclic compounds	3.780±0.417	3.170±0.240	2.842±0.043	4.549±0.318	3.949±0.080	4.867±0.179

	nitro-							
406.1160	1h-indole-1-pentanoic acid, 3-(1-naphthalenylcarbonyl)-	Organoheterocyclic compounds	1.483±0.053	1.770±0.028	0.6427±0.0374	1.532±0.022	1.793±0.075	1.643±0.036
189.0881	1h-indole-6-carboxamide, n-hydroxy-1-[(4-methoxyphenyl)methyl]-	Organoheterocyclic compounds	43.96±0.87	55.36±1.96	36.30±1.66	43.46±1.08	41.33±2.08	46.98±1.01
101.1074	1-methylpiperazine	Organoheterocyclic compounds	17.11±0.79	44.71±3.82	15.38±0.23	19.26±0.65	16.89±1.08	15.57±1.31
363.0928	1-methyluric acid	Organoheterocyclic compounds	0.8324±0.0865	0.2318±0.0155	0.8067±0.0376	1.079±0.023	0.8486±0.0744	0.9028±0.0499
372.1897	1-pentyl-3-(4-methoxynaphthoyl)indole	Organoheterocyclic compounds	0.4303±0.0323	0.1980±0.0081	#NUM!±0.00	0.3165±0.0129	0.6116±0.0702	0.8308±0.0790
217.1046	2-(2',3',4'-trihydroxybutyl)quinoxaline	Organoheterocyclic compounds	4.198±0.481	29.76±0.62	3.527±0.058	4.514±0.301	3.448±0.263	3.934±0.141
258.1461	2-heptyl-4-hydroxyquinoline n-oxide	Organoheterocyclic compounds	7.525±0.095	9.172±0.424	3.651±0.112	5.429±0.299	9.644±0.859	11.91±0.47
395.2402	2-hydroxyatrazine	Organoheterocyclic compounds	0.6356±0.0750	0.6193±0.0095	0.3005±0.0125	0.3526±0.0208	0.7267±0.0724	0.8464±0.0312
224.0691	2-methoxyacridin-9-ol	Organoheterocyclic compounds	0.3869±0.0204	0.2789±0.0235	0.4130±0.0422	0.3770±0.0457	0.3893±0.0239	0.3821±0.0271
94.0298	3-hydroxypyridine	Organoheterocyclic compounds	5.768±0.469	7.486±0.421	4.562±0.260	6.102±0.198	5.480±0.359	6.806±0.211
178.1077	3'-hydroxyrepaglinide	Organoheterocyclic compounds	3.632±0.640	4.009±0.304	#NUM!±0.00	3.086±0.054	4.720±0.390	3.329±0.315
148.0972	3-methyloxyindole	Organoheterocyclic compounds	98.55±5.73	112.2±6.1	88.00±2.22	135.3±5.1	99.20±7.29	91.25±0.80
108.0445	3-pyridinecarboxaldehyde	Organoheterocyclic compounds	3.121±0.402	5.235±0.417	1.693±0.082	3.498±0.216	4.232±0.117	4.572±0.237
241.0353	4,5-dihydro-4,5-dioxo-1h-pyrrolo[2,3-f]quinoline-2,7,9-tricarboxylic acid	Organoheterocyclic compounds	169.1±17.2	309.2±20.2	94.21±3.14	177.2±6.4	201.0±6.6	183.6±17.9
204.1343	4-aminoantipyrine	Organoheterocyclic compounds	1.562±0.445	1.593±0.036	0.5435±0.0358	0.8610±0.0792	2.320±0.148	2.370±0.046
557.2573	4-hydroxyatorvastatin lactone	Organoheterocyclic compounds	6.832±0.849	31.03±1.89	3.115±0.320	6.540±0.554	10.27±0.83	7.769±0.542
144.0302	4-hydroxyquinoline	Organoheterocyclic compounds	12.67±0.72	35.56±1.21	5.675±0.388	11.38±0.36	13.18±1.00	12.92±0.38
208.0619	4-morpholinopropanesulfonic	Organoheterocyclic	1.085±0.085	0.3112±0.0026	0.4563±0.0321	0.7607±0.0623	2.486±0.294	1.365±0.075

	acid	compounds						
129.1024	4-piperidinecarboxamide	Organoheterocyclic compounds	2.720±0.317	1.070±0.015	1.777±0.130	2.401±0.068	6.399±0.202	2.418±0.174
218.1500	5-fluoro-2-indolyl deschlorohalopemide	Organoheterocyclic compounds	1.614±0.100	2.520±0.137	1.004±0.043	1.728±0.036	1.859±0.082	1.737±0.018
291.0725	5-hydroxy-7-(hydroxymethyl)-2-methyl-2-(5-oxooxolan-2-yl)-3h-chromen-4-one	Organoheterocyclic compounds	9.509±0.416	7.789±0.200	2.913±0.126	4.632±0.256	6.314±0.206	5.486±0.064
190.0358	5-hydroxyindoleacetate	Organoheterocyclic compounds	11.02±2.72	13.34±0.40	6.421±0.425	6.895±0.591	9.708±0.948	7.653±0.742
141.0194	5-hydroxymethyl-2-furancarboxylic acid	Organoheterocyclic compounds	86.23±5.00	106.8±6.2	69.96±3.17	96.03±2.70	92.98±11.38	90.34±2.23
219.0663	5-Hydroxytryptophan	Organoheterocyclic compounds	5.101±1.340	6.945±0.147	3.028±0.052	7.123±0.598	5.279±0.126	6.491±0.109
129.0659	5-methyl-5,6-dihydrouracil	Organoheterocyclic compounds	180.7±8.1	224.9±10.1	99.73±1.48	190.5±6.3	203.0±20.1	238.8±12.8
482.1644	5-methyltetrahydrofolic acid	Organoheterocyclic compounds	1.907±0.275	1.777±0.042	0.7534±0.0209	1.912±0.133	3.819±0.219	2.272±0.178
397.0647	5-nitro-2-furaldehyde semicarbazone	Organoheterocyclic compounds	2.728±0.242	2.536±0.166	4.530±0.197	3.739±0.178	1.841±0.080	2.168±0.037
173.0455	5-nitroso-8-quinolinol	Organoheterocyclic compounds	27.37±2.53	46.78±4.36	15.59±1.44	26.74±2.39	30.72±2.54	29.16±1.74
449.0632	5-pyrimidinecarboxamide, 2-[(2,4-dichlorophenyl)amino]-n-[(tetrahydro-2h-pyran-4-yl)methyl]-4-(trifluoromethyl)-	Organoheterocyclic compounds	4.418±0.049	4.611±0.082	3.639±0.060	4.225±0.376	4.082±0.335	4.184±0.055
441.2526	6,8-dihydroxy-2,2,4,4-tetramethyl-7-(3-methylbutanoyl)-9-(2-methylpropyl)-9h-xanthene-1,3-dione	Organoheterocyclic compounds	85.04±0.66	103.3±4.2	119.2±4.1	80.53±2.42	60.47±3.09	71.39±0.59
226.1075	6-benzylaminopurine	Organoheterocyclic compounds	294.0±11.7	347.8±9.0	179.7±3.5	356.8±10.5	303.3±17.0	266.7±14.6
232.0793	6-hydroxymelatonin	Organoheterocyclic compounds	11.90±0.68	15.79±0.61	12.53±0.55	15.68±0.28	5.893±0.178	11.09±0.73
296.0646	6-methoxyflindersine	Organoheterocyclic compounds	1.447±0.108	1.341±0.134	0.7750±0.0743	1.492±0.022	1.834±0.107	1.598±0.095
240.0868	7,8-dihydrobiopterin	Organoheterocyclic	2.485±0.182	3.368±0.048	1.146±0.066	1.806±0.039	2.447±0.050	2.498±0.179

		compounds						
213.0172	8-chlorotheophylline	Organoheterocyclic compounds	5.575±0.436	33.76±0.71	4.410±0.117	5.087±0.032	3.844±0.098	6.149±0.719
379.1545	8-desoxygartanin	Organoheterocyclic compounds	1.730±0.187	1.470±0.065	1.757±0.134	2.141±0.025	1.627±0.113	1.580±0.010
190.0476	8-hydroxyquinoline-5-carboxylic acid	Organoheterocyclic compounds	3.704±0.343	1.709±0.089	5.983±0.579	3.579±0.169	2.596±0.289	3.120±0.103
152.0820	8-methylcaffeine	Organoheterocyclic compounds	38.00±3.71	110.2±8.4	16.84±0.20	31.92±2.40	52.58±5.55	55.74±1.24
266.1114	Albendazole	Organoheterocyclic compounds	0.3628±0.1089	0.07488±0.00235	0.9412±0.0672	0.4846±0.0083	0.3520±0.0428	0.3207±0.0183
298.1013	Albendazole sulfone	Organoheterocyclic compounds	3.039±0.287	1.179±0.043	2.238±0.064	2.466±0.022	3.689±0.391	3.387±0.115
228.1343	Ametryne	Organoheterocyclic compounds	2.206±0.176	7.977±0.209	1.386±0.113	2.085±0.197	2.674±0.291	2.372±0.055
232.1546	Aminophenazone	Organoheterocyclic compounds	1120±92	804.9±39.9	847.5±10.2	1093±33	1244±67	1321±72
297.0829	Amlexanox	Organoheterocyclic compounds	6.330±0.280	9.381±0.322	4.186±0.167	6.052±0.153	7.289±0.366	5.598±0.201
129.0408	Ammelide	Organoheterocyclic compounds	2.908±0.441	#NUM!±0.00	1.223±0.080	2.682±0.127	2.909±0.087	2.959±0.124
320.0989	Amoxicillin	Organoheterocyclic compounds	0.2910±0.0160	0.5811±0.0047	0.08936±0.00792	0.2217±0.0068	0.2765±0.0307	0.3010±0.0037
350.1060	Ampicillin	Organoheterocyclic compounds	3.936±0.218	5.149±0.105	3.709±0.126	4.258±0.068	3.220±0.229	4.337±0.193
394.1109	Amsacrine	Organoheterocyclic compounds	0.8469±0.1933	0.9172±0.0330	0.8141±0.0358	0.8190±0.0142	0.8842±0.0627	0.7095±0.0848
557.1333	Aprepitant	Organoheterocyclic compounds	0.3692±0.0312	0.6193±0.0172	0.2501±0.0041	0.3797±0.0112	0.3758±0.0461	0.3683±0.0193
378.1704	Atalaphylline	Organoheterocyclic compounds	7.136±0.189	8.207±0.218	5.032±0.312	6.626±0.099	7.672±0.370	8.213±0.048
559.2728	Atorvastatin	Organoheterocyclic compounds	8.363±1.309	17.05±0.50	3.205±0.255	8.090±0.070	10.14±0.38	12.47±0.55
382.1830	Azelastine	Organoheterocyclic compounds	0.8252±0.1391	1.069±0.054	0.4894±0.0215	0.8299±0.0563	0.9261±0.0368	0.9206±0.0573
230.1004	Benz[c]acridine	Organoheterocyclic compounds	3.185±0.368	4.081±0.081	2.807±0.151	3.142±0.067	2.645±0.066	3.674±0.278
219.0916	Benzamide, n-1h-indol-5-yl-	Organoheterocyclic	3.222±0.242	1.714±0.119	0.9310±0.0699	0.8467±0.0554	10.23±0.32	3.535±0.152

		compounds						
567.1683	Benzeneacetamide, 4-(4,9-diethoxy-1,3-dihydro-1-oxo-2h-benz[f]isoindol-2-yl)-n-(phenylsulfonyl)-	Organoheterocyclic compounds	0.3560±0.0627	0.1589±0.0153	0.2732±0.0158	0.4641±0.0473	0.3543±0.0058	0.3324±0.0406
238.0358	Benzo[h]quinoline-3-carboxylic acid, 1,4-dihydro-4-oxo-	Organoheterocyclic compounds	14.57±1.08	17.52±0.86	10.64±0.12	18.91±0.41	15.44±0.54	14.32±0.93
413.1971	Bisindolylmaleimide i	Organoheterocyclic compounds	23.85±1.70	54.09±5.12	9.275±0.689	25.69±0.83	23.53±0.99	30.58±2.19
326.1346	Bromosporine	Organoheterocyclic compounds	0.3851±0.0665	0.3118±0.0020	0.5051±0.0321	0.6688±0.0136	0.7590±0.0772	0.6642±0.0253
195.0877	Caffeine	Organoheterocyclic compounds	6.835±0.808	28.88±1.48	3.326±0.088	6.057±0.753	8.013±0.727	9.083±0.516
168.0657	Carbazole	Organoheterocyclic compounds	23.46±2.38	28.21±2.57	17.52±0.55	24.30±0.54	23.45±2.10	21.68±1.30
214.0687	Carbendazim	Organoheterocyclic compounds	2.347±0.254	6.551±0.144	0.9469±0.0719	2.374±0.036	2.282±0.270	2.916±0.196
217.0973	Carboline base + 4h, carboxylic acid	Organoheterocyclic compounds	3.404±0.218	0.5937±0.0057	0.3798±0.0057	1.868±0.041	7.274±0.445	6.827±0.526
382.1025	Celecoxib	Organoheterocyclic compounds	4.098±0.335	6.362±0.558	2.727±0.136	4.663±0.095	3.200±0.317	3.750±0.149
233.0304	Cephalexin	Organoheterocyclic compounds	9.306±0.983	20.04±0.83	3.180±0.220	16.63±0.62	7.761±0.501	9.020±0.120
307.1001	Cimifugin	Organoheterocyclic compounds	72.97±5.19	54.65±2.54	141.3±5.9	81.72±1.98	57.85±4.54	58.09±1.39
317.0309	Cinnavalininate	Organoheterocyclic compounds	1.485±0.032	1.956±0.060	0.6491±0.0326	1.648±0.137	1.743±0.166	2.027±0.012
332.1452	Ciprofloxacin	Organoheterocyclic compounds	2.585±0.275	3.452±0.251	2.028±0.164	2.072±0.134	2.913±0.197	3.487±0.186
469.0867	Cromolyn	Organoheterocyclic compounds	1.848±0.169	2.355±0.152	1.879±0.046	2.087±0.044	1.424±0.046	1.752±0.084
246.1700	Cycloheximide	Organoheterocyclic compounds	1615±65	2231±135	1042±38	1511±49	1689±132	1820±114
112.0506	Cytosine	Organoheterocyclic compounds	233.5±8.7	159.3±1.9	63.92±1.38	156.6±13.1	324.5±10.1	374.8±34.5
358.1741	Danofloxacin	Organoheterocyclic compounds	0.6074±0.0597	0.4123±0.0365	0.2076±0.0075	0.6234±0.0480	0.9127±0.0367	2.067±0.123
435.2235	Defluoroatorvastatin	Organoheterocyclic	1.155±0.087	1.902±0.094	0.8782±0.0101	1.303±0.016	1.092±0.027	1.200±0.055

		compounds						
173.0922	Deoxypeganine	Organoheterocyclic compounds	28.18±4.54	26.12±1.71	23.30±1.78	24.78±0.74	30.90±2.68	23.10±1.43
187.1078	Deoxyvasicinone	Organoheterocyclic compounds	39.37±1.27	44.01±1.96	35.56±0.78	38.90±0.96	37.95±2.47	36.97±0.45
280.1042	Dibenz(a,h)acridine	Organoheterocyclic compounds	13.68±0.56	22.55±0.75	8.109±0.095	12.67±0.32	12.85±0.73	14.14±0.51
200.0684	Dictamine	Organoheterocyclic compounds	28.98±3.20	33.09±1.26	20.90±0.77	18.05±1.25	31.87±2.69	36.65±1.12
356.1559	Difloxacin	Organoheterocyclic compounds	0.3120±0.0681	0.7872±0.0790	#NUM!±0.00	0.01899±0.00138	0.2090±0.0080	0.3732±0.0075
127.0513	Dihydrothymine	Organoheterocyclic compounds	77.02±2.99	89.36±5.43	49.02±0.84	69.22±2.94	75.86±5.84	83.93±0.65
188.0707	DL-tryptophan	Organoheterocyclic compounds	309.2±18.9	459.7±20.0	149.4±3.5	287.9±9.1	307.3±13.7	387.6±25.9
296.1496	Dosulepin	Organoheterocyclic compounds	5.476±0.245	8.246±0.548	3.155±0.099	5.563±0.063	6.190±0.601	5.493±0.157
175.0710	Edaravone	Organoheterocyclic compounds	9.459±1.681	14.07±1.15	6.251±0.197	10.08±0.73	10.13±0.55	13.20±1.55
247.1289	Ellipticine	Organoheterocyclic compounds	45.67±4.47	55.89±2.59	25.25±0.61	41.50±1.09	45.85±3.42	52.73±5.29
233.1174	Encecalin	Organoheterocyclic compounds	0.2910±0.0588	0.4869±0.0101	0.1043±0.0070	0.2951±0.0146	0.3211±0.0394	0.3497±0.0354
382.1432	Enrofloxacin	Organoheterocyclic compounds	1.989±0.101	0.8103±0.0288	1.267±0.066	1.685±0.085	2.655±0.324	3.081±0.104
399.0093	Ethiprole	Organoheterocyclic compounds	0.2951±0.0302	2.367±0.072	0.1290±0.0045	0.2405±0.0073	0.2962±0.0241	0.3561±0.0076
226.0715	Ethyl 3-indoleacetate	Organoheterocyclic compounds	293.1±14.9	330.8±12.8	187.4±3.9	284.6±8.5	299.4±17.6	279.2±15.3
236.0587	Ethyl 8-fluoro-4-hydroxyquinoline-3-carboxylate	Organoheterocyclic compounds	2.236±0.245	0.1845±0.0032	0.3797±0.0190	0.9891±0.0144	7.343±0.175	1.847±0.075
226.9721	Fenclorim	Organoheterocyclic compounds	3.553±0.214	4.504±0.179	1.797±0.094	2.906±0.115	3.829±0.068	4.003±0.051
424.1220	Flumiclorac-pentyl	Organoheterocyclic compounds	2.151±0.071	1.852±0.070	1.965±0.051	1.973±0.155	2.290±0.036	2.185±0.084
248.0531	Forchlorfenuron	Organoheterocyclic compounds	4.765±0.490	5.074±0.266	4.792±0.117	4.876±0.038	4.492±0.474	4.579±0.224
358.1348	Gatifloxacin	Organoheterocyclic	0.8744±0.0285	1.076±0.046	0.8816±0.0415	0.8432±0.0227	0.6766±0.0228	0.7711±0.0864

		compounds						
303.1054	Hematoxylin	Organoheterocyclic compounds	3.329±0.446	8.928±0.746	1.042±0.073	2.612±0.121	3.696±0.336	3.178±0.095
319.1001	His-Tyr	Organoheterocyclic compounds	3.144±0.446	6.180±0.197	2.721±0.189	3.976±0.046	3.011±0.236	2.844±0.169
312.1538	Imazaquin	Organoheterocyclic compounds	1.559±0.065	0.9720±0.0260	0.3642±0.0139	0.8677±0.0804	2.415±0.035	2.955±0.236
186.1126	Indole-3-butyric acid	Organoheterocyclic compounds	30.40±0.30	29.70±1.48	19.82±1.02	25.68±0.56	28.65±2.21	35.81±1.70
146.0602	Indole-3-carboxaldehyde	Organoheterocyclic compounds	18.28±1.28	27.57±1.53	8.604±0.262	17.04±0.30	19.12±0.93	23.75±1.69
204.0878	Indolelactic acid	Organoheterocyclic compounds	71.15±6.83	100.4±1.2	42.47±2.31	53.51±3.45	65.56±4.21	72.69±1.39
254.1501	Irgarol	Organoheterocyclic compounds	5.179±0.210	3.333±0.250	0.8986±0.0305	0.9151±0.0204	16.74±1.30	1.712±0.018
186.1238	Isocarbamid	Organoheterocyclic compounds	9.299±0.415	8.467±0.714	5.755±0.094	9.986±0.354	14.54±1.43	9.043±0.232
121.0397	Isoniazid	Organoheterocyclic compounds	13.17±1.49	8.446±0.566	30.32±2.94	10.64±0.75	6.450±0.232	10.49±0.60
214.0486	Kinetin	Organoheterocyclic compounds	225.1±8.1	267.3±15.2	149.2±6.9	208.3±6.6	221.7±12.4	241.2±13.0
268.0517	Lansoprazole	Organoheterocyclic compounds	150.9±6.2	285.1±14.0	88.16±1.61	132.4±4.8	169.2±6.0	176.2±1.2
260.1243	Lenalidomide	Organoheterocyclic compounds	67.53±2.06	97.74±4.97	49.74±2.45	59.56±1.87	64.94±4.23	70.30±1.41
362.1562	Levofloxacin	Organoheterocyclic compounds	0.7874±0.1780	1.344±0.052	0.3694±0.0316	0.5786±0.0403	0.7694±0.0232	0.8015±0.0231
177.0405	L-Gulonic gamma-lactone	Organoheterocyclic compounds	9.978±0.611	15.66±0.19	6.256±0.621	9.892±1.167	14.08±0.41	10.37±0.15
195.0510	L-gulono-1,4-lactone	Organoheterocyclic compounds	1583±59	2063±88	1258±58	1646±52	1563±88	1439±77
427.2302	Lovatatin	Organoheterocyclic compounds	24.76±3.63	264.0±5.4	10.00±0.85	21.32±1.43	32.73±2.35	28.25±1.90
266.0886	Lycobetaine	Organoheterocyclic compounds	6.101±0.276	9.896±0.624	4.689±0.160	6.220±0.136	5.744±0.585	5.535±0.135
248.1494	Meperidine	Organoheterocyclic compounds	121.9±21.3	43.84±3.25	297.3±8.5	153.0±17.8	55.42±4.47	55.70±1.55
186.0772	Metamitron-desamino	Organoheterocyclic	63.85±1.45	35.67±1.40	52.29±1.90	79.72±7.32	69.33±6.15	56.41±2.80

		compounds						
350.2691	Methanone, (1-pentyl-1h-indol-3-yl)tricyclo[3.3.1.13,7]dec-1-yl-	Organoheterocyclic compounds	1.485±0.180	0.4134±0.0140	1.718±0.022	1.345±0.035	1.344±0.108	1.515±0.083
388.1847	Methanone, [1-(5-hydroxypentyl)-1h-indol-3-yl](4-methoxy-1-naphthalenyl)-	Organoheterocyclic compounds	0.9538±0.0882	0.7107±0.0560	0.4282±0.0073	1.182±0.049	1.738±0.079	2.225±0.160
350.1812	Methanone, [1-[(4-fluorophenyl)methyl]-1h-indol-3-yl](2,2,3,3-tetramethylcyclopropyl)-	Organoheterocyclic compounds	4.025±0.077	4.303±0.205	3.124±0.036	4.388±0.044	4.009±0.191	4.166±0.103
251.1240	Methaqualone	Organoheterocyclic compounds	40.67±2.27	64.44±2.78	24.49±0.47	38.13±1.58	39.85±3.98	46.62±2.74
325.1077	Methopterine	Organoheterocyclic compounds	5.978±0.450	7.912±0.549	4.452±0.289	6.165±0.200	5.998±0.639	7.518±0.899
435.1446	Methotrexate	Organoheterocyclic compounds	0.8088±0.0728	0.4918±0.0254	1.024±0.078	0.8461±0.0476	0.9853±0.0657	0.9521±0.0962
234.1338	Metolachlor-morpholinone	Organoheterocyclic compounds	4.130±0.183	3.035±0.056	3.028±0.054	2.903±0.137	5.079±0.179	5.414±0.382
212.0911	Milrinone	Organoheterocyclic compounds	14.07±0.32	18.97±1.20	10.41±0.37	15.62±1.09	14.31±1.13	24.63±2.03
452.2485	NCGC00381123-01	Organoheterocyclic compounds	1.215±0.093	0.4094±0.0303	0.2134±0.0203	0.7137±0.0323	1.825±0.089	2.619±0.212
326.1236	N-desmethyldanofloxacin	Organoheterocyclic compounds	0.5778±0.0657	2.233±0.148	0.2930±0.0176	0.4777±0.0099	0.7293±0.0307	0.4858±0.0108
144.0656	N-ethylmaleimide	Organoheterocyclic compounds	11.49±1.54	19.95±0.64	2.983±0.120	8.459±0.188	21.81±2.32	13.58±0.08
411.0903	Nicosulfuron	Organoheterocyclic compounds	1.260±0.013	1.788±0.058	0.8199±0.0546	1.347±0.144	1.235±0.097	1.427±0.069
163.1231	Nicotine	Organoheterocyclic compounds	67.71±7.36	117.9±7.2	33.19±0.78	77.34±5.73	69.07±3.31	82.61±0.79
282.0951	Nitrazepam	Organoheterocyclic compounds	5.495±0.739	11.93±0.42	2.983±0.279	6.050±0.270	5.002±0.607	5.656±0.416
175.1442	N-methyltryptamine	Organoheterocyclic compounds	158.7±8.4	257.2±18.4	86.64±1.14	150.8±11.9	204.7±16.0	194.0±11.6
153.0660	Nudifloramide	Organoheterocyclic compounds	1.601±0.181	0.3175±0.0224	2.012±0.036	1.922±0.022	1.298±0.079	1.337±0.088
431.1483	O-desmethylgefitinib	Organoheterocyclic	0.3607±0.0355	0.2885±0.0158	0.2301±0.0112	0.3441±0.0098	0.3590±0.0210	0.3977±0.0180

		compounds						
299.1716	Olomoucine	Organoheterocyclic compounds	1.823±0.013	2.394±0.241	1.120±0.047	1.802±0.059	1.990±0.089	2.009±0.102
338.1560	Olopatadine	Organoheterocyclic compounds	3.678±0.239	5.210±0.100	2.557±0.146	3.566±0.162	3.416±0.233	3.371±0.099
149.0929	Omeprazole sulfone n-oxide	Organoheterocyclic compounds	1.652±0.144	3.772±0.265	0.7119±0.0683	1.849±0.087	1.732±0.070	2.366±0.170
262.0509	Oxolinic acid	Organoheterocyclic compounds	3.523±0.746	6.549±0.265	1.410±0.092	3.777±0.238	3.011±0.155	3.574±0.441
181.0720	Paraxanthine	Organoheterocyclic compounds	8.636±0.465	39.01±2.55	4.484±0.191	8.093±0.159	9.846±0.969	9.773±0.529
180.0654	Phenanthridine	Organoheterocyclic compounds	69.62±5.24	109.6±9.2	45.06±1.05	68.49±2.01	75.99±5.89	76.17±1.40
249.1559	Pindolol	Organoheterocyclic compounds	1.273±0.072	2.044±0.117	0.8562±0.0139	1.506±0.017	1.176±0.007	1.261±0.052
363.0695	Piperazine-n,n'-bis(2-hydroxypropanesulfonic acid)	Organoheterocyclic compounds	0.8078±0.1140	1.261±0.020	0.6643±0.0280	0.7447±0.0161	0.8342±0.0383	1.075±0.095
358.1775	Pitavastatin	Organoheterocyclic compounds	0.7422±0.0768	1.277±0.016	0.1498±0.0087	0.6125±0.0114	0.5487±0.0209	0.5798±0.0637
325.0937	Prazepam	Organoheterocyclic compounds	0.7286±0.1019	0.7423±0.0450	1.096±0.028	1.005±0.042	0.3594±0.0367	0.5401±0.0338
382.1525	Prazosin	Organoheterocyclic compounds	2.142±0.074	2.181±0.028	3.013±0.138	3.103±0.024	1.676±0.064	1.690±0.139
230.1386	Propazine	Organoheterocyclic compounds	441.3±13.5	588.7±34.1	366.3±14.9	442.3±14.0	463.1±30.8	455.8±27.4
211.0827	Pyocyanin	Organoheterocyclic compounds	5.919±0.227	12.00±1.11	3.794±0.044	6.687±0.039	6.349±0.468	6.274±0.270
379.1215	Pyridate	Organoheterocyclic compounds	4.098±0.786	4.537±0.306	4.035±0.220	3.567±0.227	3.269±0.090	5.405±0.073
378.1163	Pyridine, 4-[4-(4-fluorophenyl)-2-[4-(methylsulfinyl)phenyl]-1h-imidazol-5-yl]-	Organoheterocyclic compounds	0.8518±0.1174	0.5544±0.0260	0.4025±0.0194	0.9576±0.0260	0.9102±0.0678	1.111±0.064
168.0656	Pyridoxal	Organoheterocyclic compounds	11.75±1.32	30.00±0.74	7.601±0.559	11.37±1.17	10.34±1.00	12.30±1.00
169.0973	Pyridoxamine	Organoheterocyclic compounds	8.311±0.291	10.73±0.98	6.239±0.428	7.276±0.312	8.895±0.249	7.944±0.100
247.0460	Pyridoxamine 5-phosphate	Organoheterocyclic compounds	7.056±0.465	11.97±0.59	3.698±0.251	8.219±0.269	7.995±0.210	8.138±0.514

170.0812	Pyridoxine	Organoheterocyclic compounds	164.3±12.6	461.3±23.7	62.04±4.41	138.1±5.7	210.6±10.0	185.7±18.4
110.0247	Pyrrole-2-carboxylic acid	Organoheterocyclic compounds	19.75±0.26	19.13±0.67	5.751±0.246	9.716±0.127	26.31±0.78	25.29±1.54
196.0604	Quinaldic acid	Organoheterocyclic compounds	187.2±10.8	208.4±10.6	172.5±9.6	245.1±9.1	179.8±15.5	164.8±1.6
144.0667	Quinolin-2-ol	Organoheterocyclic compounds	2.258±0.216	#NUM!±0.00	0.9598±0.0703	0.9948±0.0478	7.550±0.713	1.769±0.133
131.0454	Quinoxaline	Organoheterocyclic compounds	3.069±0.100	8.722±0.827	1.563±0.022	3.097±0.024	3.283±0.285	3.400±0.101
288.1056	Rutaecarpine	Organoheterocyclic compounds	44.57±2.52	1.853±0.025	1.477±0.046	3.098±0.018	93.34±4.87	28.36±1.61
273.1446	Sempervirine	Organoheterocyclic compounds	27.64±1.07	31.24±1.27	20.00±0.41	25.96±1.02	28.97±1.71	32.44±2.28
238.0935	Sepiapterin	Organoheterocyclic compounds	161.6±35.7	322.0±4.6	80.12±1.84	149.0±9.5	163.2±8.0	180.9±14.7
323.0740	Temazepam	Organoheterocyclic compounds	367.1±21.0	467.7±16.7	284.4±5.7	369.9±11.9	362.0±25.0	365.2±19.7
176.0919	Tenofovir	Organoheterocyclic compounds	33.56±1.94	37.23±2.25	22.19±0.29	49.68±5.20	35.62±2.69	350.9±37.6
163.0428	Theobromine	Organoheterocyclic compounds	1.752±0.035	3.007±0.056	2.678±0.182	2.309±0.121	1.564±0.048	1.599±0.024
181.0530	Theophylline	Organoheterocyclic compounds	15.04±0.94	51.26±2.09	13.82±1.10	29.38±0.73	9.403±0.660	10.97±0.09
358.1974	Trans-epoxysuccinyl-l-leucylamido(4-guanidino)butane	Organoheterocyclic compounds	7.328±0.553	10.64±0.34	2.645±0.063	6.542±0.118	8.969±0.453	8.506±0.550
408.1480	Trifluoroperazine	Organoheterocyclic compounds	4.945±0.316	3.338±0.290	8.513±0.101	5.012±0.134	4.746±0.162	4.424±0.081
209.0091	Trimellitic acid anhydride	Organoheterocyclic compounds	18.45±3.36	18.94±1.49	15.59±1.26	35.09±0.90	12.29±0.38	5.431±0.064
283.1298	Tropisetron	Organoheterocyclic compounds	1.111±0.201	2.595±0.203	4.989±0.234	5.430±0.139	5.032±0.230	2.952±0.091
111.0200	Uracil	Organoheterocyclic compounds	56.58±2.74	185.4±16.1	8.740±0.394	15.91±0.85	42.08±2.13	66.30±6.05
309.0585	Urea, n-[4-(2-chlorophenyl)-6,7-dimethyl-3-quinolinyl]-n'-(2,4-difluorophenyl)-	Organoheterocyclic compounds	9.687±0.383	12.66±0.66	5.893±0.264	11.23±0.30	8.957±0.214	10.19±0.21

432.1117	Urea, n-[4-[4-amino-1-(1-methylethyl)-1h-pyrazolo[3,4-d]pyrimidin-3-yl]phenyl]-n'-[2-fluoro-5-(trifluoromethyl)phenyl]-	Organoheterocyclic compounds	4.142±0.196	4.346±0.190	4.015±0.052	3.853±0.036	3.494±0.193	4.177±0.085
264.1266	Zaleplon	Organoheterocyclic compounds	6.306±0.789	4.125±0.108	4.167±0.117	5.679±0.042	7.142±0.218	7.388±0.369
235.1190	Zolpidem	Organoheterocyclic compounds	21.90±2.30	7.011±0.342	19.41±1.05	35.89±0.85	22.84±2.60	25.95±1.62
217.0973	4,4'-thiodianiline	Organosulfur compounds	14.15±0.58	3.238±0.091	10.77±0.38	38.57±1.43	3.327±0.204	22.37±1.36
198.0538	6-tert-butyl-3-methylsulfanyl-2h-1,2,4-triazin-5-one	Organosulfur compounds	3.746±0.532	9.308±0.611	3.133±0.178	4.009±0.471	4.246±0.134	3.948±0.143
187.1078	Metribuzin	Organosulfur compounds	34.27±0.41	33.88±2.29	25.02±0.90	27.69±0.95	36.72±2.84	42.29±1.48
415.2562	Propanoic acid, 2-[[4-[2-[[(cyclohexylamino)carbonyl](4-cyclohexylbutyl)amino]ethyl]phenyl]thio]-2-methyl-	Organosulfur compounds	1.722±0.164	0.7379±0.0110	0.7861±0.0306	1.332±0.150	2.199±0.133	2.837±0.141
137.0357	(-)-catechin	Phenylpropanoids and polyketides	1.384±0.201	1.244±0.060	1.545±0.078	0.4864±0.0239	1.439±0.080	1.572±0.163
291.1053	(+)-catechin	Phenylpropanoids and polyketides	45.95±6.78	21.55±0.94	41.20±2.59	41.57±1.37	42.91±2.25	55.57±1.08
485.2356	(2r,3r,4s,5s,6r)-2-[1,7-bis(4-hydroxyphenyl)heptan-3-yloxy]-6-(hydroxymethyl)oxane-3,4,5-triol	Phenylpropanoids and polyketides	1.853±0.260	19.66±0.50	0.8992±0.0831	1.383±0.097	2.495±0.295	2.095±0.189
301.1009	2',4'-dihydroxy-2,3-dimethoxychalcone	Phenylpropanoids and polyketides	3.438±0.254	5.409±0.153	2.439±0.083	3.148±0.023	2.813±0.098	3.713±0.429
283.1106	2,6'-dimethoxy-2'-hydroxychalcone	Phenylpropanoids and polyketides	0.3919±0.0276	0.3897±0.0153	0.2919±0.0264	0.1955±0.0059	0.1620±0.0017	0.2833±0.0108
315.1166	2-hydroxy-2',4',6'-trimethoxychalcone	Phenylpropanoids and polyketides	7.320±0.441	8.943±0.184	6.425±0.312	7.311±0.251	6.064±0.362	7.687±0.325
255.0912	2-hydroxy-3-methoxychalcone	Phenylpropanoids and polyketides	12.71±0.84	12.13±1.03	10.28±0.36	13.55±1.00	14.70±0.81	14.70±0.91
431.1642	3-(2,4-dihydroxyphenyl)-7-hydroxy-6,8-bis(3-methylbut-2-enyl)-2,3-dihydrochromen-4-	Phenylpropanoids and polyketides	1.650±0.167	2.455±0.101	1.484±0.072	1.438±0.051	1.085±0.117	1.583±0.168

	one							
365.0847	3,2',4',5'-tetramethoxyflavone	Phenylpropanoids and polyketides	5.899±0.370	6.767±0.320	5.343±0.283	6.353±0.149	6.362±0.715	6.114±0.239
377.0757	3',4',5,7-tetrahydroxy-3,6,8-trimethoxyflavone	Phenylpropanoids and polyketides	1.287±0.130	0.6035±0.0505	1.427±0.039	1.775±0.210	1.729±0.155	2.220±0.264
269.0304	3,4',7-trihydroxyflavone	Phenylpropanoids and polyketides	10.41±0.87	18.19±0.54	7.367±0.344	11.08±0.21	11.45±0.33	11.18±0.33
285.1311	3',4'-dimethoxy-2'-hydroxychalcone	Phenylpropanoids and polyketides	3.695±0.101	5.307±0.197	1.819±0.067	3.550±0.212	3.072±0.230	4.725±0.377
247.0784	3,5-dimethoxy-4-hydroxycinnamic acid	Phenylpropanoids and polyketides	105.8±2.5	195.4±10.5	101.1±4.5	94.28±3.07	100.5±2.2	101.7±1.9
303.0689	3,7,3',4',5'-pentahydroxyflavone	Phenylpropanoids and polyketides	1.320±0.078	1.090±0.105	0.5665±0.0387	1.468±0.044	1.990±0.060	2.075±0.047
313.0911	3,7,3'-trimethoxyflavone	Phenylpropanoids and polyketides	2.836±0.110	5.687±0.199	1.107±0.064	1.883±0.066	2.215±0.070	2.197±0.028
299.0853	4-hydroxy-7-methoxy-3-(4-methoxyphenyl)coumarin	Phenylpropanoids and polyketides	0.7847±0.0479	1.103±0.088	0.7766±0.0404	0.8761±0.0649	0.8504±0.0447	0.9489±0.0291
223.0823	4'-hydroxychalcone	Phenylpropanoids and polyketides	24.12±1.73	35.34±2.52	17.07±1.68	21.79±0.81	25.85±0.69	29.77±1.27
161.0559	4-methoxycinnamic acid	Phenylpropanoids and polyketides	3.136±0.314	3.794±0.080	2.205±0.081	3.187±0.118	3.170±0.192	3.392±0.106
353.0847	4-methylumbelliferyl .beta.-d-glucuronide	Phenylpropanoids and polyketides	10.68±0.86	13.40±0.65	11.47±0.50	12.11±0.16	9.210±0.652	11.41±0.15
253.0580	5,3'-dihydroxyflavone	Phenylpropanoids and polyketides	51.80±2.12	70.08±2.45	38.61±1.74	50.91±1.32	51.32±4.10	55.07±0.85
239.1140	5-hydroxyflavone	Phenylpropanoids and polyketides	1.519±0.089	2.370±0.090	1.318±0.116	1.733±0.021	1.350±0.154	1.841±0.030
355.1452	6',7'-dihydroxybergamottin	Phenylpropanoids and polyketides	2.316±0.218	1.941±0.079	3.179±0.155	2.754±0.132	2.203±0.132	2.514±0.077
407.2107	6,8-diprenylnaringenin	Phenylpropanoids and polyketides	4.003±0.289	4.131±0.180	2.097±0.140	4.374±0.054	5.452±0.305	5.386±0.418
237.0769	6-hydroxyflavone	Phenylpropanoids and polyketides	29.81±0.83	53.24±3.29	16.93±0.90	33.13±2.68	29.71±2.16	32.53±0.44
313.1144	7,2',3'-trimethoxyflavone	Phenylpropanoids and polyketides	0.4390±0.0742	0.4822±0.0476	0.3522±0.0175	0.4244±0.0141	0.5352±0.0052	0.5269±0.0407
249.0405	7,8-dihydroxy-4-methylcoumarin-3-acetic acid	Phenylpropanoids and polyketides	0.8862±0.0648	2.002±0.025	#NUM!±0.00	0.6745±0.0167	1.047±0.087	1.571±0.098
161.0456	7-hydroxycoumarin	Phenylpropanoids and	5.855±0.781	5.381±0.218	5.675±0.264	5.987±0.398	6.106±0.547	6.931±0.706

		polyketides						
221.0455	7-hydroxycoumarin-4-acetic acid	Phenylpropanoids and polyketides	210.6±9.4	357.7±17.0	206.1±11.6	195.5±5.8	191.1±2.8	193.6±13.7
239.0776	7-hydroxyflavone	Phenylpropanoids and polyketides	13.60±0.19	6.258±0.559	14.64±0.58	13.53±0.32	11.66±0.45	12.15±0.41
279.0529	8-carboxy-3-methylflavone	Phenylpropanoids and polyketides	180.8±10.3	127.6±4.8	221.3±10.1	173.2±5.3	165.7±3.5	159.7±1.4
269.0702	Apigenin	Phenylpropanoids and polyketides	2.298±0.110	2.802±0.164	1.362±0.062	2.201±0.136	2.633±0.112	2.491±0.096
455.1165	Apigenin 7-glucoside	Phenylpropanoids and polyketides	4.660±0.326	5.411±0.374	4.436±0.244	4.598±0.266	4.217±0.353	4.671±0.253
342.1071	Benzoic acid, 2-[[(2z)-3-(3,4-dimethoxyphenyl)-1-oxo-2-propen-1-yl]amino]-3-hydroxy-	Phenylpropanoids and polyketides	1.186±0.131	0.7839±0.0502	0.8078±0.0752	1.047±0.069	1.261±0.114	1.345±0.125
221.0956	Benzyl cinnamate	Phenylpropanoids and polyketides	2.306±0.200	0.1980±0.0077	0.03193±0.00071	0.3494±0.0272	10.57±0.91	0.9531±0.0403
181.0530	Caffeic acid	Phenylpropanoids and polyketides	29.93±0.13	53.09±1.56	18.06±0.62	31.12±0.73	28.42±1.96	27.59±1.37
358.0909	Casticin	Phenylpropanoids and polyketides	0.2247±0.0145	0.08054±0.00498	0.1482±0.0073	0.3412±0.0116	0.2518±0.0237	0.2511±0.0043
320.0796	Cinnamyl 3,4-dihydroxy-.alpha.-cyanocinnamate	Phenylpropanoids and polyketides	3.034±0.047	2.420±0.120	1.574±0.077	3.149±0.130	3.018±0.078	3.275±0.123
217.0715	Demethoxycurcumin	Phenylpropanoids and polyketides	8.032±0.176	11.35±0.21	6.530±0.306	8.339±0.099	8.719±0.258	9.097±0.081
530.2593	Epothilone b	Phenylpropanoids and polyketides	1.225±0.046	#NUM!±0.00	0.6667±0.0537	2.023±0.197	1.282±0.045	1.395±0.087
289.0897	Eriodictyol	Phenylpropanoids and polyketides	5.791±0.500	8.028±0.194	6.430±0.219	5.926±0.270	4.352±0.310	6.383±0.591
301.1008	Farrerol	Phenylpropanoids and polyketides	4.414±0.268	5.686±0.314	3.290±0.246	4.368±0.129	3.981±0.133	5.318±0.335
269.0748	Formononetin	Phenylpropanoids and polyketides	2.210±0.203	1.254±0.033	2.214±0.079	2.588±0.262	2.230±0.173	2.091±0.017
615.1714	Fortunellin	Phenylpropanoids and polyketides	0.8007±0.0571	0.4049±0.0053	1.330±0.127	0.9791±0.0651	0.6699±0.0095	0.6348±0.0513
461.0936	Homoplantaginin	Phenylpropanoids and polyketides	0.4913±0.0632	0.4469±0.0149	0.4805±0.0321	0.4601±0.0224	0.3687±0.0255	0.4925±0.0290
361.1107	Irigenin	Phenylpropanoids and polyketides	1.016±0.187	1.237±0.074	0.8242±0.0560	1.245±0.117	0.7319±0.0795	1.036±0.092

387.1175	Irisflorentin	Phenylpropanoids and polyketides	1.298±0.157	1.867±0.173	1.057±0.041	1.384±0.046	1.333±0.071	1.588±0.018
269.0879	Isoimperatorin	Phenylpropanoids and polyketides	12.60±0.39	16.46±0.69	7.926±0.383	13.75±0.19	13.39±0.87	23.39±1.60
255.0887	Isoliquiritin	Phenylpropanoids and polyketides	1.425±0.077	1.388±0.063	0.9670±0.0556	0.9573±0.0467	2.744±0.171	2.292±0.102
269.0399	Isopimpinellin	Phenylpropanoids and polyketides	2.073±0.093	2.535±0.085	1.056±0.047	2.021±0.032	2.424±0.086	2.425±0.080
287.0876	Isosakuranetin	Phenylpropanoids and polyketides	1.788±0.131	3.234±0.169	0.7447±0.0511	1.796±0.051	2.040±0.107	1.877±0.050
285.1346	Loureirin a	Phenylpropanoids and polyketides	2.340±0.204	3.832±0.338	1.258±0.079	2.440±0.181	2.580±0.094	3.027±0.125
471.1117	Luteolin 7-glucoside	Phenylpropanoids and polyketides	0.9916±0.0669	1.396±0.082	0.5605±0.0304	1.006±0.021	1.076±0.062	1.077±0.039
281.0547	Luvangetin	Phenylpropanoids and polyketides	2.615±0.169	2.475±0.131	3.006±0.102	2.718±0.157	2.507±0.255	2.380±0.154
285.0696	Maackiaine	Phenylpropanoids and polyketides	6.709±0.442	2.729±0.158	10.20±0.39	7.062±0.113	5.972±0.170	5.676±0.193
449.0936	Marein	Phenylpropanoids and polyketides	1.268±0.204	0.2838±0.0091	0.3307±0.0148	0.3145±0.0312	2.652±0.198	2.385±0.014
303.0510	Morin	Phenylpropanoids and polyketides	0.4536±0.0473	0.5632±0.0385	#NUM!±0.00	0.3619±0.0246	0.4751±0.0252	0.4834±0.0365
499.1577	Mundulone acetate	Phenylpropanoids and polyketides	0.8072±0.0729	0.9221±0.0291	0.3192±0.0155	0.5952±0.0197	0.6796±0.0454	0.9756±0.0376
147.0651	Naringenin	Phenylpropanoids and polyketides	8.355±0.404	8.301±0.412	9.964±0.360	10.18±0.23	7.497±0.456	6.844±0.280
419.1165	Narirutin	Phenylpropanoids and polyketides	0.8283±0.0955	0.9553±0.0721	0.3387±0.0183	0.8341±0.0308	1.022±0.019	1.187±0.034
345.1162	Nevadensin	Phenylpropanoids and polyketides	0.4885±0.0526	0.6427±0.0169	0.4819±0.0206	0.5468±0.0442	0.4150±0.0249	0.5052±0.0344
403.1125	Nobiletin	Phenylpropanoids and polyketides	3.131±0.325	2.225±0.111	5.108±0.174	3.340±0.027	2.667±0.080	2.933±0.176
409.1313	Nodakenin	Phenylpropanoids and polyketides	1.188±0.078	1.300±0.032	2.766±0.107	1.372±0.149	1.599±0.047	1.402±0.148
357.0633	Orientin	Phenylpropanoids and polyketides	0.7572±0.0057	1.283±0.126	#NUM!±0.00	0.8870±0.0566	0.7190±0.0260	0.9703±0.1161
147.0441	P-coumaric acid	Phenylpropanoids and polyketides	16.39±0.40	17.59±0.57	11.62±0.40	15.64±0.69	16.51±0.49	18.77±1.13

495.1328	Picroside ii	Phenylpropanoids and polyketides	1.139±0.198	1.591±0.035	0.9643±0.0569	1.014±0.121	1.116±0.101	1.385±0.097
389.1202	Polydatin	Phenylpropanoids and polyketides	0.7743±0.0444	1.302±0.032	0.6485±0.0461	0.9848±0.0132	0.7464±0.0311	0.5385±0.0649
335.0985	Psoralidin	Phenylpropanoids and polyketides	13.42±0.93	20.95±0.70	7.138±0.493	10.23±0.96	16.13±0.47	17.77±1.09
395.1663	Rotenone	Phenylpropanoids and polyketides	31.09±8.84	2.952±0.128	11.38±0.78	17.59±0.73	26.64±2.53	60.05±3.63
681.1296	Rutarensin	Phenylpropanoids and polyketides	0.2531±0.0036	#NUM!±0.00	0.08041±0.00278	0.2414±0.0081	0.4287±0.0159	0.4337±0.0213
409.1321	Samidin	Phenylpropanoids and polyketides	2.428±0.150	3.261±0.206	2.571±0.183	2.585±0.093	1.712±0.083	2.424±0.128
377.0692	Scopolin	Phenylpropanoids and polyketides	1.023±0.077	2.336±0.035	0.6087±0.0406	1.086±0.053	1.216±0.038	1.026±0.068
427.1265	Spinosine	Phenylpropanoids and polyketides	1.668±0.060	1.438±0.027	3.020±0.136	2.371±0.019	1.594±0.050	1.873±0.053
393.1007	Swertisin	Phenylpropanoids and polyketides	3.396±0.229	4.369±0.077	2.578±0.157	4.104±0.208	2.695±0.144	2.475±0.161
395.1198	Tangeritin	Phenylpropanoids and polyketides	0.8022±0.0949	0.6491±0.0145	2.631±0.099	0.3806±0.0248	0.8330±0.0825	1.084±0.063
463.1327	Tectoridin	Phenylpropanoids and polyketides	5.799±0.120	0.8737±0.0223	1.961±0.076	4.379±0.397	9.306±0.986	13.56±0.33
309.1312	Warfarin	Phenylpropanoids and polyketides	3.204±0.354	4.827±0.100	1.568±0.109	3.600±0.250	2.960±0.250	4.481±0.438
337.0965	Wighteone	Phenylpropanoids and polyketides	3.717±0.300	4.880±0.120	1.424±0.105	3.339±0.378	4.462±0.116	5.041±0.076
