# Supplementary material for: Untargeted Metabolomic Analysis Combined with Chemometrics Revealed the Effects of Different Cooking Methods on Lentinus edodes
Source: Molecules. 2023 Aug 11;28(16):6009. doi: 10.3390/molecules28166009 (PMC10458448; doi:10.3390/molecules28166009)
Supplement: Supplementary file 1 [file molecules-28-06009-s001.zip › Table S2.pdf]

Table S2 Classification of differential metabolites in four pairwise comparisons.

Class name	Boiling vs Control		Steaming vs Control		Air-frying vs Control		Roasting vs Control	
	**Down**	**Up**	**Down**	**Up**	**Down**	**Up**	**Down**	**Up**
Benzenoids	31	8	21	12	14	17	21	19
Lipids and lipid-like molecules	35	14	27	32	31	28	26	48
Nucleosides, nucleotides, and analogues	2	4	2	6	3	8	3	10
Organic acids and derivatives	38	10	18	21	16	32	16	46
Organic nitrogen compounds	5	2	4	2	4	4	4	5
Organic oxygen compounds	9	5	9	9	5	9	8	15
Organoheterocyclic compounds	37	8	21	13	20	22	19	30
Phenylpropanoids and polyketides	8	5	3	8	2	8	3	13
Others	3	0	1	1	0	2	0	5
Total	168	56	106	104	95	130	100	191
