# Supplementary material for: Untargeted Metabolomic Analysis Combined with Chemometrics Revealed the Effects of Different Cooking Methods on Lentinus edodes
Source: Molecules. 2023 Aug 11;28(16):6009. doi: 10.3390/molecules28166009 (PMC10458448; doi:10.3390/molecules28166009)
Supplement: Supplementary file 1 [file molecules-28-06009-s001.zip › Table S3.pdf]

Table S3 List of differential metabolites between Boiling and Control

m/z	Name	SuperClass	Control	Boiling	Fold Change (FC)	log2(FC)	P value (10^-6)	VIP	Type
205.1334	Caulophylline	Alkaloids and derivatives	1.940±0.134	0.4820±0.0270	0.37513	-1.4145	77.54	1.053	Down
205.1336	N-methylcytisine	Alkaloids and derivatives	2.022±0.159	0.5655±0.0470	0.4227	-1.2423	158.38	1.006	Down
145.0609	.beta.-naphthol	Benzenoids	11.49±0.40	2.002±0.189	0.26275	-1.9282	29.02	1.180	Down
253.1799	1-benzhydrylpiperazine	Benzenoids	17.46±1.59	1.762±0.137	0.1525	-2.7131	6.38	1.351	Down
178.0719	2,2-bis(4-chlorophenyl)ethanol	Benzenoids	31.19±2.23	2.421±0.165	0.11721	-3.0928	0.48	1.427	Down
208.0968	2-chloro-2',6'-diethylacetanilide	Benzenoids	0.06858±0.00637	0.6309±0.0585	13.912	3.7983	1.24	1.329	Up
207.1130	2-ethyl-2-phenylmalonamide	Benzenoids	17.80±1.33	4.699±0.215	0.39949	-1.3238	25.06	1.029	Down
227.0198	3,5-dinitrosalicylate	Benzenoids	11.13±0.74	2.323±0.165	0.31488	-1.6671	5.84	1.117	Down
256.0827	3-hydroxymethylmefenamic acid	Benzenoids	641.1±41.8	118.8±5.2	0.28002	-1.8364	4.06	1.158	Down
170.0813	4-aminobiphenyl	Benzenoids	15.64±1.28	4.064±0.199	0.39298	-1.3475	12.91	1.036	Down
201.0074	4-hydroxy-2',5'-dichlorobiphenyl	Benzenoids	175.7±3.4	41.61±1.96	0.35766	-1.4833	2.45	1.071	Down
105.0699	4-methylbenzyl alcohol	Benzenoids	72.25±3.97	5.706±0.335	0.11931	-3.0673	0.21	1.422	Down
289.0236	5-benzoyl-4-hydroxy-2-methoxybenzene sulfonic acid	Benzenoids	9.607±0.338	1.529±0.069	0.24005	-2.0586	0.27	1.210	Down
417.2712	Alfentanyl	Benzenoids	2.007±0.144	0.5098±0.0327	0.38439	-1.3794	44.99	1.044	Down
266.1250	Anisomycin	Benzenoids	38.84±1.62	6.566±0.412	0.25538	-1.9693	1.13	1.190	Down
392.1302	Benzamide, 4-[(3ar,4r,7s,7as)-1,3,3a,4,7,7a-hexahydro-1,3-dioxo-4,7-methano-2h-isoindol-2-yl]-n-8-quinolinyl-, rel-	Benzenoids	1.476±0.063	0.2626±0.0059	0.26782	-1.9007	0.06	1.173	Down
377.0693	Benzenepropanamide, n-(6-chloro-2-benzothiazolyl)-3,4-dimethoxy-	Benzenoids	18.02±1.15	1.525±0.067	0.12829	-2.9625	0.72	1.402	Down

446.0851	Benzenesulfonamide, n-[[(1,1-dimethylethyl)amino]carbonyl]-2-(3-methoxyphenoxy)-5-nitro-	Benzenoids	0.1790±0.0136	0.02041±0.00057	0.17243	-2.5359	2.12	1.315	Down
354.9838	Benzenesulfonic acid, 2-[(5-bromo-2-hydroxyphenyl)methylene]hydrazide	Benzenoids	0.3494±0.0184	1.528±0.077	6.5963	2.7217	3.66	1.084	Up
387.1512	Cetirizine	Benzenoids	0.1351±0.0059	#NUM!±0.00	0.28602	-1.8058	14.06	3.354	Down
287.0410	Ciprofibrate	Benzenoids	23.55±1.28	4.154±0.312	0.26597	-1.9106	2.93	1.176	Down
397.1157	Daunomycinone	Benzenoids	8.189±0.147	2.099±0.158	0.38717	-1.369	15.00	1.041	Down
441.3075	Di(2-nonyl) phthalate	Benzenoids	3.346±0.199	0.4434±0.0208	0.20037	-2.3193	0.62	1.269	Down
273.1083	Dipropyl phthalate	Benzenoids	44.39±0.63	11.24±0.27	0.382	-1.3884	0.96	1.046	Down
169.0584	Ethyl 2,4-dihydroxy-6-methylbenzoate	Benzenoids	6.236±0.111	22.46±1.06	5.4307	2.4411	0.25	1.010	Up
159.0279	Fenfluramine	Benzenoids	3.861±0.387	0.8188±0.0048	0.32084	-1.6401	14.19	1.110	Down
297.0882	Flunixin	Benzenoids	7.740±0.172	0.8484±0.0800	0.16548	-2.5953	3.35	1.328	Down
231.0511	Fluometuron	Benzenoids	69.56±6.66	18.62±0.38	0.40533	-1.3028	28.56	1.023	Down
316.1006	Flusilazole	Benzenoids	#NUM!±0.00	0.1572±0.0162	8.92	3.157	6.98	3.371	Up
494.2590	Imatinib	Benzenoids	#NUM!±0.00	0.3211±0.0187	8.1069	3.0191	1.20	3.455	Up
194.1177	Isoproterenol	Benzenoids	1.208±0.119	#NUM!±0.00	0.22333	-2.1628	7.08	3.604	Down
412.1104	Mandipropamid	Benzenoids	7.696±0.137	0.3599±0.0244	0.070532	-3.8256	0.21	1.562	Down
343.1615	Methanone, 1-naphthalenyl(1-pentyl-1h-indazol-3-yl)-	Benzenoids	0.1111±0.0061	0.7521±0.0087	10.236	3.3556	0.34	1.235	Up
399.1467	N,n'-dicarbobenzyloxy-l-ornithine	Benzenoids	6.084±0.195	1.086±0.049	0.269	-1.8943	0.42	1.172	Down
215.0562	Nepodin	Benzenoids	25.51±0.82	5.852±0.267	0.34624	-1.5301	17.46	1.083	Down
122.0964	Phenylethylamine	Benzenoids	30.06±1.98	2.630±0.063	0.13244	-2.9166	0.22	1.393	Down
200.1025	Pyrimethanil	Benzenoids	26.68±1.00	3.538±0.115	0.20011	-2.3211	0.01	1.269	Down
475.2152	Sildenafil	Benzenoids	4.171±0.310	0.3022±0.0036	0.10965	-3.189	0.25	1.446	Down

387.1992	Sufentanyl	Benzenoids	4.780±0.105	0.9351±0.0490	0.2955	-1.7588	2.40	1.140	Down
405.1483	Sulfinpyrazone	Benzenoids	1.019±0.043	9.543±0.184	14.159	3.8237	0.43	1.335	Up
223.1443	Zectran	Benzenoids	0.04378±0.00111	0.2358±0.0199	8.1316	3.0235	4.28	1.157	Up
399.0951	4'-demethylpodophyllotoxin	Lignans, neolignans and related compounds	32.53±1.30	6.544±0.294	0.30371	-1.7192	0.45	1.130	Down
233.1495	(+)-costunolide	Lipids and lipid-like molecules	25.13±1.80	6.910±0.518	0.4156	-1.2667	37.15	1.014	Down
311.2229	(9z,12e)-15,16-dihydroxyoctadeca-9,12-dienoic acid	Lipids and lipid-like molecules	117.8±2.1	8.038±0.391	0.10319	-3.2766	0.14	1.463	Down
357.2497	.beta.-estradiol 17-valerate	Lipids and lipid-like molecules	110.5±5.1	30.68±0.54	0.4206	-1.2495	3.40	1.010	Down
401.2165	1,2-dihydrodesoxymetasone	Lipids and lipid-like molecules	7.729±0.370	0.8107±0.0500	0.15819	-2.6602	5.69	1.340	Down
688.4907	1,2-dipalmitoleoyl-sn-glycero-3-phospho ethanolamine	Lipids and lipid-like molecules	2.882±0.040	11.07±0.19	5.8037	2.537	0.47	1.035	Up
295.2280	12(13)-epoxy-9z-octadecenoic acid	Lipids and lipid-like molecules	41.28±1.91	7.661±0.638	0.2804	-1.8345	26.39	1.158	Down
371.2300	15(r),19(r)-hydroxyprostaglandin f1.alpha.	Lipids and lipid-like molecules	5.817±0.114	1.582±0.108	0.41	-1.2863	8.10	1.018	Down
424.3061	17-phenyltrinorprostaglandin f2.alpha. cyclopropyl methyl amide	Lipids and lipid-like molecules	5.225±0.202	37.87±2.55	10.945	3.4521	0.26	1.256	Up
608.4658	1-lignoceroyl-2-hydroxy-sn-glycero-3-ph osphocholine	Lipids and lipid-like molecules	0.09495±0.00928	3.214±0.056	51.261	5.6798	0.23	1.676	Up
468.3087	1-myristoyl-sn-glycero-3-phosphocholine	Lipids and lipid-like molecules	3.324±0.320	13.95±0.44	6.3414	2.6648	1.97	1.070	Up
546.3544	1-stearoyl-2-hydroxy-sn-glycero-3-phosp hocholine	Lipids and lipid-like molecules	0.5798±0.0442	3.867±0.242	10.079	3.3333	0.77	1.230	Up
145.0506	2,2-Dimethylsuccinic acid	Lipids and lipid-like	51.02±2.40	8.648±0.398	0.25604	-1.9656	2.81	1.189	Down

		molecules							
175.0612	2-Isopropylmalic acid	Lipids and lipid-like molecules	130.5±8.3	7.735±0.750	0.089194	-3.4869	4.77	1.500	Down
117.0557	2-methyl-3-hydroxybutyric acid	Lipids and lipid-like molecules	11.80±0.69	2.763±0.099	0.35334	-1.5009	21.47	1.075	Down
630.3465	3-deoxyaconitine	Lipids and lipid-like molecules	1.019±0.072	0.1445±0.0058	0.21502	-2.2174	4.71	1.247	Down
543.2779	3-hydroxystanozolol glucuronide	Lipids and lipid-like molecules	4.153±0.075	0.2016±0.0191	0.07326	-3.7708	0.58	1.553	Down
431.1902	6.beta.-hydroxyeplerenone	Lipids and lipid-like molecules	0.1452±0.0077	#NUM!±0.00	0.28289	-1.8217	18.98	3.362	Down
293.2123	9-oxo-10(e),12(e)-octadecadienoic acid	Lipids and lipid-like molecules	29.08±1.64	5.996±0.597	0.3112	-1.6841	20.77	1.122	Down
327.1781	Acitretin	Lipids and lipid-like molecules	17.95±0.61	2.593±0.025	0.21826	-2.1959	0.03	1.242	Down
301.1760	Adrenosterone	Lipids and lipid-like molecules	12.54±0.54	2.761±0.048	0.33339	-1.5847	0.69	1.098	Down
427.2560	Andrastin d	Lipids and lipid-like molecules	1.867±0.126	0.3958±0.0219	0.32091	-1.6398	2.58	1.111	Down
628.3626	Bulleyaconi cine a	Lipids and lipid-like molecules	1.589±0.022	0.1889±0.0050	0.1798	-2.4756	0.99	1.303	Down
405.1695	Chlormadinone acetate	Lipids and lipid-like molecules	4.894±0.230	#NUM!±0.00	0.047385	-4.3994	0.00	3.756	Down
465.3043	Cholesteryl sulfate	Lipids and lipid-like molecules	0.4531±0.0143	2.621±0.164	8.7324	3.1264	2.21	1.182	Up
407.2955	Cholic acid	Lipids and lipid-like molecules	2.081±0.074	47.78±3.76	34.709	5.1172	0.24	1.579	Up
443.2251	Cinobufagin	Lipids and lipid-like	398.0±5.5	78.33±2.72	0.29688	-1.752	1.34	1.138	Down

		molecules							
327.2179	Cis-4,7,10,13,16,19-docosahexaenoic acid	Lipids and lipid-like molecules	32.88±2.74	5.179±0.262	0.23787	-2.0718	2.15	1.213	Down
187.1437	Costunolide	Lipids and lipid-like molecules	31.36±2.22	6.107±0.130	0.29435	-1.7644	2.62	1.141	Down
297.1675	Exemestane	Lipids and lipid-like molecules	3.977±0.077	0.9062±0.0657	0.34395	-1.5397	18.04	1.085	Down
427.1329	Gardenoside	Lipids and lipid-like molecules	0.3402±0.0176	2.996±0.126	13.289	3.7322	1.60	1.317	Up
219.1744	Germacrone	Lipids and lipid-like molecules	9.224±0.502	1.511±0.097	0.24739	-2.0151	2.78	1.201	Down
309.1675	Gestrinone	Lipids and lipid-like molecules	25.46±2.58	1.380±0.135	0.082097	-3.6065	5.31	1.523	Down
283.1267	Gibberellic acid	Lipids and lipid-like molecules	3.917±0.106	0.7548±0.0248	0.29076	-1.7821	2.37	1.145	Down
315.1675	Gibberellin a9	Lipids and lipid-like molecules	0.8112±0.0429	0.2130±0.0130	0.39591	-1.3368	106.85	1.032	Down
466.3028	Glycocholic acid	Lipids and lipid-like molecules	1.224±0.018	0.3277±0.0213	0.40411	-1.3072	37.91	1.024	Down
495.2601	Leukotriene d4	Lipids and lipid-like molecules	7.080±0.187	0.7616±0.0450	0.16242	-2.6222	0.19	1.333	Down
513.6652	Linoleoyl coenzyme a	Lipids and lipid-like molecules	3.285±0.179	0.8961±0.0435	0.41167	-1.2804	43.56	1.016	Down
1028.3369	Linoleoyl-CoA	Lipids and lipid-like molecules	0.4858±0.0376	#NUM!±0.00	0.10673	-3.228	3.88	3.503	Down
365.1056	Maltose	Lipids and lipid-like molecules	22.27±0.81	84.51±1.59	5.7339	2.5195	0.69	1.031	Up
477.1693	Obacunone	Lipids and lipid-like	1.008±0.061	4.187±0.074	6.2714	2.6488	5.68	1.066	Up

		molecules							
143.1077	Octanoic acid	Lipids and lipid-like molecules	6.343±0.254	1.618±0.122	0.38462	-1.3785	9.21	1.044	Down
297.1530	Ostruthin	Lipids and lipid-like molecules	43.26±2.38	456.7±8.0	15.944	3.995	0.38	1.370	Up
714.5078	Pe 34:2	Lipids and lipid-like molecules	23.99±1.61	123.5±5.7	7.7622	2.9565	2.65	1.142	Up
738.5080	Pe 36:4	Lipids and lipid-like molecules	179.7±9.2	818.1±43.6	6.8658	2.7794	2.46	1.098	Up
639.4085	Phorbol 12-myristate 13-acetate	Lipids and lipid-like molecules	4.310±0.409	0.9708±0.0940	0.34012	-1.5559	67.00	1.089	Down
833.5185	Pi 34:2	Lipids and lipid-like molecules	9.720±0.335	67.95±3.48	10.548	3.3989	0.38	1.244	Up
173.0922	Thymol	Lipids and lipid-like molecules	4.775±0.488	0.7496±0.0421	0.23766	-2.073	18.38	1.213	Down
494.3090	Tiamulin	Lipids and lipid-like molecules	0.3013±0.0018	0.05452±0.00415	0.27329	-1.8715	23.34	1.167	Down
195.0763	Verbenalin	Lipids and lipid-like molecules	84.87±4.87	24.09±1.06	0.42958	-1.219	22.88	1.001	Down
560.0795	Adenosine 5'-diphosphoribose	Nucleosides, nucleotides, and analogues	0.1029±0.0001	3.697±0.079	54.206	5.7604	0.02	1.689	Up
462.0669	Adenylosuccinate	Nucleosides, nucleotides, and analogues	0.8736±0.0803	#NUM!±0.00	0.00879 2	-6.8296	0.05	3.569	Down
464.0819	Adenylosuccinic acid	Nucleosides, nucleotides, and analogues	0.9471±0.0109	#NUM!±0.00	0.01641 4	-5.929	0.01	3.578	Down
558.0644	Adp-ribose	Nucleosides, nucleotides, and analogues	0.2528±0.0033	5.458±0.251	32.59	5.0264	0.07	1.564	Up
252.1092	Deoxyadenosine	Nucleosides, nucleotides,	13.14±0.76	2.704±0.092	0.31097	-1.6852	3.40	1.122	Down

		and analogues							
809.0125	Uridine 5'-diphosphate (UDP)	Nucleosides, nucleotides, and analogues	11.87±0.42	2.765±0.039	0.35238	-1.5048	0.60	1.077	Down
263.0877	.alpha.-L-Glu-L-Asp	Organic acids and derivatives	7.437±0.260	2.041±0.089	0.41447	-1.2707	21.71	1.014	Down
86.0603	.gamma.-aminobutyric acid	Organic acids and derivatives	753.1±36.7	140.4±5.5	0.28085	-1.8321	0.37	1.157	Down
209.0125	1,2,4-benzenetricarboxylic acid	Organic acids and derivatives	1.504±0.018	0.2585±0.0075	0.25992	-1.9439	1.05	1.185	Down
365.0531	2s-amino-4-phosphonobutyric acid	Organic acids and derivatives	5.769±0.184	1.096±0.061	0.28739	-1.7989	3.43	1.150	Down
140.0707	3-amino-2,3-dihydrobenzoic acid	Organic acids and derivatives	119.1±7.3	29.41±1.53	0.37229	-1.4255	6.66	1.056	Down
348.0394	4-hydroxytriamterene sulfate	Organic acids and derivatives	#NUM!±0.00	0.2313±0.0211	8.4289	3.0753	4.42	3.417	Up
114.0664	5-aminolevulinic acid	Organic acids and derivatives	53.38±3.92	13.18±1.10	0.37265	-1.4241	32.30	1.056	Down
148.0760	Albizziin	Organic acids and derivatives	7.013±0.429	1.966±0.164	0.42388	-1.2383	49.62	1.007	Down
115.0401	Alpha-ketoisovaleric acid	Organic acids and derivatives	3.668±0.265	23.21±0.66	9.5549	3.2562	2.39	1.213	Up
117.9968	Aminomalonic acid	Organic acids and derivatives	0.06721±0.00253	0.6596±0.0349	14.792	3.8867	0.71	1.349	Up
393.1945	Asp-Met-Lys	Organic acids and derivatives	5.152±0.278	1.138±0.114	0.33474	-1.5789	87.63	1.096	Down
231.0977	Asp-Pro	Organic acids and derivatives	8.158±0.549	2.155±0.021	0.39923	-1.3247	5.45	1.029	Down
425.2148	Benazepril	Organic acids and	10.37±0.83	1.498±0.102	0.21773	-2.1994	4.36	1.242	Down

		derivatives								
240.0656	Captopril	Organic acids and derivatives	0.01295±0.00072	0.3406±0.0231	39.595	5.3073	0.38	1.614	Up
248.0930	Cys-Gln	Organic acids and derivatives	1.197±0.014	7.525±0.267	9.4937	3.247	0.21	1.210	Up
441.2095	Cys-Tyr-Arg	Organic acids and derivatives	33.66±0.53	2.683±0.193	0.12051	-3.0527	0.89	1.420	Down
312.9849	Dicloxacillin	Organic acids and derivatives	5.134±0.174	0.5645±0.0138	0.16611	-2.5898	1.40	1.326	Down
223.0747	Dl-cystathionine	Organic acids and derivatives	69.51±4.43	18.33±0.48	0.39941	-1.3241	3.18	1.030	Down
166.0865	DL-phenylalanine	Organic acids and derivatives	673.2±21.7	#NUM!±0.00	0.2105	-2.2481	0.03	4.247	Down
130.0496	D-pyroglutamic acid	Organic acids and derivatives	4.048±0.102	32.70±1.00	12.206	3.6095	0.01	1.290	Up
275.1352	Gln-gln	Organic acids and derivatives	14.97±0.47	2.611±0.042	0.2628	-1.928	0.59	1.180	Down
613.1598	Glutathione, oxidized	Organic acids and derivatives	22.74±1.43	0.8162±0.0504	0.054192	-4.2058	0.11	1.628	Down
334.1401	Glu-Trp	Organic acids and derivatives	0.2474±0.0251	0.9790±0.0079	5.9587	2.575	20.82	1.048	Up
459.2199	Hc toxin	Organic acids and derivatives	9.466±0.938	1.035±0.070	0.16474	-2.6017	27.49	1.327	Down
293.1346	His-His	Organic acids and derivatives	1.984±0.050	0.5534±0.0204	0.42158	-1.2461	14.93	1.009	Down
269.1610	His-Leu	Organic acids and derivatives	14.45±0.70	3.538±0.056	0.37046	-1.4326	1.11	1.058	Down
229.1546	Ile-Pro	Organic acids and	51.41±2.83	8.075±0.490	0.2372	-2.0758	1.38	1.214	Down

		derivatives								
306.1298	Imazamox	Organic acids and derivatives	48.45±3.04	13.31±0.41	0.41485	-1.2693	3.05	1.014	Down
212.0058	Indoxyl sulfate	Organic acids and derivatives	#NUM!±0.00	0.3981±0.0144	7.8032	2.9641	0.22	3.480	Up
188.0918	L-abrine	Organic acids and derivatives	23.98±2.30	6.196±0.249	0.39061	-1.3562	44.83	1.036	Down
154.0510	L-carnosine	Organic acids and derivatives	11.36±0.34	3.171±0.144	0.42293	-1.2415	2.57	1.008	Down
427.0956	L-cysteine-glutathione disulfide	Organic acids and derivatives	1.321±0.053	0.03398±0.00061	0.038867	-4.6853	0.28	1.708	Down
239.0196	L-cystine	Organic acids and derivatives	13.96±0.49	3.608±0.130	0.39128	-1.3537	4.65	1.038	Down
401.2762	Leu-Ile-Arg	Organic acids and derivatives	2.187±0.141	0.5546±0.0435	0.38378	-1.3816	82.20	1.044	Down
260.1971	Lys-Leu	Organic acids and derivatives	9.660±0.332	2.566±0.017	0.4022	-1.314	0.31	1.028	Down
248.1494	Mefenoxam	Organic acids and derivatives	29.54±1.68	#NUM!±0.00	0.18328	-2.4479	0.06	3.942	Down
102.0550	N-(.beta.-ketocaproyl)-dl-homoserine lactone	Organic acids and derivatives	5.447±0.417	0.3729±0.0108	0.10341	-3.2736	1.36	1.461	Down
244.1545	N-3-hydroxyoctanoyl-l-homoserine lactone	Organic acids and derivatives	20.21±1.40	5.551±0.235	0.41538	-1.2675	6.42	1.014	Down
198.0891	N-acetylhistidine	Organic acids and derivatives	60.94±5.23	16.00±0.83	0.3969	-1.3332	20.90	1.032	Down
116.0706	N-benzyl-d-methionine methyl ester	Organic acids and derivatives	3.795±0.168	0.8230±0.0126	0.32783	-1.609	1.03	1.103	Down
210.1338	N-octanoyl-l-homoserine lactone	Organic acids and	5.907±0.584	0.8554±0.0484	0.21898	-2.1911	6.64	1.240	Down

		derivatives							
118.0863	Norvaline	Organic acids and derivatives	80.96±8.27	2.036±0.207	0.03803 3	-4.7166	0.98	1.713	Down
277.1228	Pantetheine	Organic acids and derivatives	120.2±8.3	0.5456±0.0397	0.00685	-7.1897	0.03	2.073	Down
294.1451	Phe-gln	Organic acids and derivatives	1.593±0.095	0.4228±0.0206	0.40155	-1.3163	27.94	1.027	Down
359.1829	Pyroglu-thr-lys	Organic acids and derivatives	0.1030±0.0050	0.6351±0.0196	9.3278	3.2215	2.22	1.204	Up
88.0404	Sarcosine	Organic acids and derivatives	315.2±3.7	43.28±1.65	0.20728	-2.2703	0.20	1.258	Down
421.2426	Tris(2-butoxyethyl) phosphate	Organic acids and derivatives	#NUM!±0.00	0.1974±0.0145	8.2312	3.0411	1.84	3.399	Up
302.1502	Trp-Pro	Organic acids and derivatives	15.80±1.59	1.091±0.081	0.10432	-3.2608	2.35	1.459	Down
210.1126	2,2-bis[hydroxymethyl]-2,2',2"-nitrilotrie thanol	Organic nitrogen compounds	3.339±0.208	0.9285±0.0505	0.42003	-1.2514	13.79	1.010	Down
104.1071	Choline	Organic nitrogen compounds	4586±364	1302±24	0.42866	-1.2221	8.51	1.001	Down
134.1176	Ethyldiethanolamine	Organic nitrogen compounds	4.197±0.188	0.9781±0.0440	0.35312	-1.5018	10.12	1.077	Down
262.1288	Methapyrilene	Organic nitrogen compounds	38.11±1.16	2.569±0.061	0.1019	-3.2947	0.02	1.466	Down
184.0733	Miltefosine	Organic nitrogen compounds	143.1±1.7	36.61±0.27	0.3857	-1.3745	1.05	1.042	Down
206.1653	N1-(1-methyl-4-piperidinyl)-1,4-benzene diamine	Organic nitrogen compounds	#NUM!±0.00	0.1341±0.0136	57.55	5.8467	0.60	3.353	Up
516.3030	Oleyloxyethylphosphorylcholine	Organic nitrogen	#NUM!±0.00	0.07411±0.00164	7.6864	2.9423	0.31	3.282	Up

		compounds							
421.0754	.alpha.,.alpha.'-trehalose 6-phosphate	Organic oxygen compounds	9.040±0.199	1.479±0.079	0.24717	-2.0164	0.41	1.201	Down
535.1518	3-deoxy-d-glycero-d-galacto-2-nonulosonic acid	Organic oxygen compounds	6.737±0.596	0.7833±0.0353	0.17582	-2.5078	1.77	1.309	Down
195.0875	4',6'-dimethoxy-2'-hydroxyacetophenone	Organic oxygen compounds	35.66±2.68	9.563±0.557	0.40618	-1.2998	12.56	1.024	Down
540.0540	Cyclic adenosine diphosphate ribose	Organic oxygen compounds	18.95±1.12	3.711±0.189	0.29559	-1.7583	13.53	1.139	Down
180.0688	D-mannosamine	Organic oxygen compounds	0.4765±0.0280	2.502±0.018	7.9398	2.9891	0.11	1.150	Up
259.0130	D-mannose 6-phosphate	Organic oxygen compounds	65.47±2.22	10.45±0.48	0.24135	-2.0508	1.23	1.209	Down
461.1512	Forsythoside e	Organic oxygen compounds	0.8042±0.0390	0.2213±0.0156	0.41479	-1.2695	94.98	1.013	Down
637.1533	Leiocarposide	Organic oxygen compounds	0.09020±0.00712	0.8274±0.0156	13.85	3.7918	1.25	1.329	Up
746.2126	Lnnt	Organic oxygen compounds	0.8881±0.0811	0.2232±0.0014	0.37956	-1.3976	29.35	1.047	Down
447.1588	N,n'-diacetylchitobiose	Organic oxygen compounds	0.9065±0.0434	6.471±0.048	10.787	3.4312	0.13	1.252	Up
465.1696	N-acetylglucosamine	Organic oxygen compounds	0.4625±0.0143	1.947±0.050	6.3437	2.6653	0.14	1.070	Up
251.0776	Orcinol .beta.-d-glucoside	Organic oxygen compounds	24.30±2.37	3.051±0.109	0.1896	-2.399	2.31	1.285	Down
499.1645	Primeverin	Organic oxygen compounds	0.1147±0.0064	0.6137±0.0264	8.1091	3.0195	0.73	1.156	Up
195.1227	Tetraethylene glycol	Organic oxygen	65.47±5.85	15.26±0.54	0.35271	-1.5034	10.69	1.076	Down

		compounds							
189.1236	.alpha.-ethyltryptamine	Organoheterocyclic compounds	5.798±0.238	172.9±6.2	45.096	5.4949	0.00	1.645	Up
204.0689	1-(2,8-dihydroxyquinolin-5-yl)ethan-1-one	Organoheterocyclic compounds	29.36±0.75	1.975±0.130	0.10156	-3.2996	0.29	1.467	Down
372.1897	1-pentyl-3-(4-methoxynaphthoyl)indole	Organoheterocyclic compounds	0.1980±0.0081	#NUM!±0.00	0.29086	-1.7816	2.49	3.399	Down
217.1046	2-(2',3',4'-trihydroxybutyl)quinoxaline	Organoheterocyclic compounds	29.76±0.62	3.527±0.058	0.17897	-2.4822	0.08	1.304	Down
178.1077	3'-hydroxyrepaglinide	Organoheterocyclic compounds	4.009±0.304	#NUM!±0.00	0.21854	-2.1941	1.08	3.735	Down
557.2573	4-hydroxyatorvastatin lactone	Organoheterocyclic compounds	31.03±1.89	3.115±0.320	0.15153	-2.7223	5.15	1.354	Down
144.0302	4-hydroxyquinoline	Organoheterocyclic compounds	35.56±1.21	5.675±0.388	0.24065	-2.055	1.60	1.209	Down
213.0172	8-chlorotheophylline	Organoheterocyclic compounds	33.76±0.71	4.410±0.117	0.19734	-2.3413	0.66	1.274	Down
152.0820	8-methylcaffeine	Organoheterocyclic compounds	110.2±8.4	16.84±0.20	0.23118	-2.1129	1.60	1.223	Down
266.1114	Albendazole	Organoheterocyclic compounds	0.07488±0.00235	0.9412±0.0672	18.936	4.243	0.55	1.420	Up
228.1343	Ametryne	Organoheterocyclic compounds	7.977±0.209	1.386±0.113	0.26274	-1.9283	13.49	1.181	Down
129.0408	Ammelide	Organoheterocyclic compounds	#NUM!±0.00	1.223±0.080	8.1668	3.0298	0.48	3.606	Up
320.0989	Amoxicillin	Organoheterocyclic compounds	0.5811±0.0047	0.08936±0.00792	0.23219	-2.1066	17.05	1.222	Down
559.2728	Atorvastatin	Organoheterocyclic	17.05±0.50	3.205±0.255	0.28376	-1.8172	10.93	1.154	Down

		compounds							
413.1971	Bisindolylmaleimide i	Organoheterocyclic compounds	54.09±5.12	9.275±0.689	0.25968	-1.9452	17.96	1.185	Down
195.0877	Caffeine	Organoheterocyclic compounds	28.88±1.48	3.326±0.088	0.17397	-2.5231	0.01	1.312	Down
214.0687	Carbendazim	Organoheterocyclic compounds	6.551±0.144	0.9469±0.0719	0.21782	-2.1988	3.83	1.241	Down
233.0304	Cephalexin	Organoheterocyclic compounds	20.04±0.83	3.180±0.220	0.23957	-2.0615	10.42	1.211	Down
356.1559	Difloxacin	Organoheterocyclic compounds	0.7872±0.0790	#NUM!±0.00	0.006761	-7.2086	0.06	3.557	Down
233.1174	Encecalin	Organoheterocyclic compounds	0.4869±0.0101	0.1043±0.0070	0.32269	-1.6318	4.29	1.108	Down
399.0093	Ethiprole	Organoheterocyclic compounds	2.367±0.072	0.1290±0.0045	0.082347	-3.6021	0.02	1.523	Down
303.1054	Hematoxylin	Organoheterocyclic compounds	8.928±0.746	1.042±0.073	0.17607	-2.5057	2.74	1.308	Down
254.1501	Irgarol	Organoheterocyclic compounds	3.333±0.250	0.8986±0.0305	0.40656	-1.2985	107.42	1.020	Down
121.0397	Isoniazid	Organoheterocyclic compounds	8.446±0.566	30.32±2.94	5.4181	2.4378	6.63	1.008	Up
362.1562	Levofloxacin	Organoheterocyclic compounds	1.344±0.052	0.3694±0.0316	0.41593	-1.2656	48.43	1.014	Down
427.2302	Lovatatin	Organoheterocyclic compounds	264.0±5.4	10.00±0.85	0.057133	-4.1295	0.71	1.615	Down
248.1494	Meperidine	Organoheterocyclic compounds	43.84±3.25	297.3±8.5	10.228	3.3544	2.15	1.235	Up
350.2691	Methanone,	Organoheterocyclic	0.4134±0.0140	1.718±0.022	6.2674	2.6479	1.65	1.065	Up

	(1-pentyl-1h-indol-3-yl)tricyclo[3.3.1.13, 7]dec-1-yl-	compounds							
326.1236	N-desmethyldanofloxacin	Organoheterocyclic compounds	2.233±0.148	0.2930±0.0176	0.19834	-2.334	1.51	1.272	Down
144.0656	N-ethylmaleimide	Organoheterocyclic compounds	19.95±0.64	2.983±0.120	0.22613	-2.1448	0.98	1.231	Down
163.1231	Nicotine	Organoheterocyclic compounds	117.9±7.2	33.19±0.78	0.42507	-1.2342	11.38	1.004	Down
282.0951	Nitrazepam	Organoheterocyclic compounds	11.93±0.42	2.983±0.279	0.37731	-1.4062	55.68	1.051	Down
153.0660	Nudifloramide	Organoheterocyclic compounds	0.3175±0.0224	2.012±0.036	9.5656	3.2579	0.44	1.213	Up
149.0929	Omeprazole sulfone n-oxide	Organoheterocyclic compounds	3.772±0.265	0.7119±0.0683	0.28504	-1.8108	18.45	1.153	Down
262.0509	Oxolinic acid	Organoheterocyclic compounds	6.549±0.265	1.410±0.092	0.32467	-1.6229	17.01	1.106	Down
181.0720	Paraxanthine	Organoheterocyclic compounds	39.01±2.55	4.484±0.191	0.17392	-2.5235	1.46	1.312	Down
358.1775	Pitavastatin	Organoheterocyclic compounds	1.277±0.016	0.1498±0.0087	0.17796	-2.4903	4.38	1.307	Down
168.0656	Pyridoxal	Organoheterocyclic compounds	30.00±0.74	7.601±0.559	0.38251	-1.3864	27.92	1.046	Down
170.0812	Pyridoxine	Organoheterocyclic compounds	461.3±23.7	62.04±4.41	0.20295	-2.3008	9.53	1.264	Down
144.0667	Quinolin-2-ol	Organoheterocyclic compounds	#NUM!±0.00	0.9598±0.0703	7.9223	2.9859	0.91	3.579	Up
131.0454	Quinoxaline	Organoheterocyclic compounds	8.722±0.827	1.563±0.022	0.27112	-1.883	6.25	1.169	Down

238.0935	Sepiapterin	Organoheterocyclic compounds	322.0±4.6	80.12±1.84	0.37536	-1.4137	6.57	1.053	Down
181.0530	Theophylline	Organoheterocyclic compounds	51.26±2.09	13.82±1.10	0.40661	-1.2983	24.64	1.022	Down
358.1974	Trans-epoxysuccinyl-l-leucylamido(4-guanidino)butane	Organoheterocyclic compounds	10.64±0.34	2.645±0.063	0.37621	-1.4104	2.41	1.053	Down
111.0200	Uracil	Organoheterocyclic compounds	185.4±16.1	8.740±0.394	0.071163	-3.8127	0.31	1.560	Down
485.2356	(2r,3r,4s,5s,6r)-2-[1,7-bis(4-hydroxyphenyl)heptan-3-yloxy]-6-(hydroxymethyl)oxane-3,4,5-triol	Phenylpropanoids and polyketides	19.66±0.50	0.8992±0.0831	0.069108	-3.855	0.51	1.568	Down
313.0911	3,7,3'-trimethoxyflavone	Phenylpropanoids and polyketides	5.687±0.199	1.107±0.064	0.29387	-1.7667	1.10	1.142	Down
249.0405	7,8-dihydroxy-4-methylcoumarin-3-acetic acid	Phenylpropanoids and polyketides	2.002±0.025	#NUM!±0.00	0.098901	-3.3379	0.04	3.660	Down
221.0956	Benzyl cinnamate	Phenylpropanoids and polyketides	0.1980±0.0077	0.03193±0.00071	0.2433	-2.0392	7.68	1.206	Down
530.2593	Epothilone b	Phenylpropanoids and polyketides	#NUM!±0.00	0.6667±0.0537	8.3115	3.0551	1.01	3.538	Up
287.0876	Isosakuranetin	Phenylpropanoids and polyketides	3.234±0.169	0.7447±0.0511	0.34784	-1.5235	6.57	1.082	Down
285.0696	Maackiaine	Phenylpropanoids and polyketides	2.729±0.158	10.20±0.39	5.6598	2.5008	0.48	1.025	Up
303.0510	Morin	Phenylpropanoids and polyketides	0.5632±0.0385	#NUM!±0.00	0.17924	-2.48	0.80	3.519	Down
357.0633	Orientin	Phenylpropanoids and polyketides	1.283±0.126	#NUM!±0.00	0.16261	-2.6205	3.14	3.611	Down
395.1663	Rotenone	Phenylpropanoids and	2.952±0.128	11.38±0.78	5.8185	2.5407	0.57	1.036	Up

		polyketides							
681.1296	Rutarensin	Phenylpropanoids and polyketides	#NUM!±0.00	0.08041±0.00278	7.7503	2.9543	0.17	3.292	Up
377.0692	Scopolin	Phenylpropanoids and polyketides	2.336±0.035	0.6087±0.0406	0.39392	-1.344	7.86	1.035	Down
395.1198	Tangeritin	Phenylpropanoids and polyketides	0.6491±0.0145	2.631±0.099	6.1136	2.612	0.79	1.056	Up
