# Supplementary material for: Untargeted Metabolomic Analysis Combined with Chemometrics Revealed the Effects of Different Cooking Methods on Lentinus edodes
Source: Molecules. 2023 Aug 11;28(16):6009. doi: 10.3390/molecules28166009 (PMC10458448; doi:10.3390/molecules28166009)
Supplement: Supplementary file 1 [file molecules-28-06009-s001.zip › Table S4.pdf]

Table S4 List of differential metabolites between Steaming and Control

m/z	Name	SuperClass	Area (10^7) Control	Area (10^7) Steaming	Fold Change (FC)	log2(FC)	P value (10^-6)	VIP	Type
205.1334	Caulophylline	Alkaloids and derivatives	1.940±0.134	0.6058±0.0615	0.40028	-1.3209	299.51	1.1709	Down
421.1222	(5-benzoyloxy-4,6-dihydroxy-3-methoxycyclohexen-1-yl)methyl benzoate	Benzenoids	2.321±0.109	13.51±0.31	7.4613	2.8994	0.02	1.4445	Up
145.0609	.beta.-naphthol	Benzenoids	11.49±0.40	3.320±0.242	0.36898	-1.4384	12.28	1.2125	Down
253.1799	1-benzhydrylpiperazine	Benzenoids	17.46±1.59	3.321±0.103	0.24385	-2.0359	4.56	1.4008	Down
178.0719	2,2-bis(4-chlorophenyl)ethanol	Benzenoids	31.19±2.23	2.195±0.168	0.090302	-3.4691	2.29	1.7723	Down
208.0968	2-chloro-2',6'-diethylacetanilide	Benzenoids	0.06858±0.00637	1.729±0.134	32.428	5.0192	0.66	1.9542	Up
168.0306	2-methoxy-5-nitrophenol	Benzenoids	2.312±0.039	0.5718±0.0144	0.31695	-1.6577	5.95	1.2861	Down
227.0198	3,5-dinitrosalicylate	Benzenoids	11.13±0.74	2.265±0.265	0.26071	-1.9395	32.11	1.3733	Down
256.0827	3-hydroxymethylmefenamic acid	Benzenoids	641.1±41.8	225.4±11.4	0.45081	-1.1494	4.61	1.1121	Down
105.0699	4-methylbenzyl alcohol	Benzenoids	72.25±3.97	5.835±0.166	0.10365	-3.2703	0.02	1.7261	Down
266.1250	Anisomycin	Benzenoids	38.84±1.62	8.469±0.065	0.27962	-1.8385	0.04	1.3428	Down
404.1335	Azoxystrobin	Benzenoids	0.2330±0.0074	0.7864±0.0612	4.319	2.1107	27.97	1.1983	Up

392.1302	Benzamide, 4-[(3ar,4r,7s,7as)-1,3,3a,4,7,7a-hexahydro-1,3-dioxo-4,7-methano-2h-isoindol-2-yl]-n-8-quinolinyl-, rel-	Benzenoids	1.476±0.063	0.4905±0.0173	0.42519	-1.2338	4.81	1.1418	Down
377.0693	Benzenepropanamide, n-(6-chloro-2-benzothiazolyl)-3,4-dimethoxy-	Benzenoids	18.02±1.15	3.712±0.162	0.26466	-1.9178	1.24	1.3675	Down
354.9838	Benzenesulfonic acid, 2-[(5-bromo-2-hydroxyphenyl)methylene]hydrazide	Benzenoids	0.3494±0.0184	1.594±0.072	5.8366	2.5451	9.21	1.3406	Up
287.0410	Ciprofibrate	Benzenoids	23.55±1.28	7.116±0.287	0.38755	-1.3676	8.30	1.1899	Down
397.1157	Daunomycinone	Benzenoids	8.189±0.147	2.732±0.140	0.42733	-1.2266	9.21	1.1400	Down
317.1112	Dibutyl phthalate	Benzenoids	0.1847±0.0059	1.113±0.061	7.7407	2.9525	1.93	1.4579	Up
159.0279	Fenfluramine	Benzenoids	3.861±0.387	0.3598±0.0197	0.11955	-3.0643	1.79	1.6752	Down
297.0882	Flunixin	Benzenoids	7.740±0.172	2.020±0.154	0.33445	-1.5801	13.61	1.2613	Down
316.1006	Flusilazole	Benzenoids	#NUM!±0.00	0.1438±0.0091	6.9363	2.7942	1.69	4.0977	Up
153.0193	Gentisic acid	Benzenoids	73.00±5.72	220.7±7.8	3.8799	1.956	1.03	1.1452	Up
494.2590	Imatinib	Benzenoids	#NUM!±0.00	0.9453±0.0325	20.257	4.3403	0.07	4.3615	Up
412.1104	Mandipropamid	Benzenoids	7.696±0.137	0.3187±0.0106	0.053095	-4.2353	0.14	1.9420	Down
343.1615	Methanone, 1-naphthalenyl(1-pentyl-1h-indazol-3-yl)-	Benzenoids	0.1111±0.0061	1.056±0.044	12.199	3.6087	0.45	1.6328	Up
279.1188	Oxadixyl	Benzenoids	2.047±0.096	0.7306±0.0264	0.4572	-1.1289	72.13	1.1031	Down

					5					
276.0846	Paraoxon	Benzenoids	23.54±1.17	4.032±0.194	0.21964	-2.1868	0.60	1.4454	Down
122.0964	Phenylethylamine	Benzenoids	30.06±1.98	2.548±0.046	0.10881	-3.2001	0.07	1.7092	Down
200.1025	Pyrimethanil	Benzenoids	26.68±1.00	3.611±0.021	0.17343	-2.5276	0.04	1.5389	Down
475.2152	Sildenafil	Benzenoids	4.171±0.310	1.065±0.066	0.32745	-1.6107	12.69	1.2710	Down
313.0299	Spirodiclofen	Benzenoids	1.531±0.093	3.693±0.036	3.0975	1.6311	2.24	1.0214	Up
387.1992	Sufentanyl	Benzenoids	4.780±0.105	1.439±0.116	0.38577	-1.3742	104.07	1.1922	Down
405.1483	Sulfinpyrazone	Benzenoids	1.019±0.043	9.446±0.685	11.903	3.5732	2.91	1.6232	Up
223.1443	Zectran	Benzenoids	0.04378±0.00111	0.2282±0.0100	6.6761	2.739	1.72	1.3978	Up
488.2518	(2e,6e,11e,13e)-18-(2,6-dioxopiperidin-4-yl)-9-hydroxy-8-methoxy-10,12,14-trimethyl-15-oxooctadeca-2,6,11,13-tetraenoic acid	Lipids and lipid-like molecules	#NUM!±0.00	0.1793±0.0083	10.746	3.4257	0.73	4.1295	Up
311.2229	(9z,12e)-15,16-dihydroxyoctadeca-9,12-dienoic acid	Lipids and lipid-like molecules	117.8±2.1	11.96±0.18	0.13037	-2.9393	0.04	1.6460	Down
476.2782	1-(9z,12z-octadecadienoyl)-2-hydroxy-sn-glycero-3-phosphoethanolamine	Lipids and lipid-like molecules	308.4±14.1	95.36±2.50	0.39606	-1.3362	28.34	1.1784	Down
401.2165	1,2-dihydrodesoxymetasone	Lipids and lipid-like molecules	7.729±0.370	1.843±0.070	0.30553	-1.7106	37.23	1.3029	Down
688.4907	1,2-dipalmitoleoyl-sn-glycero-3-phosphoethanolamine	Lipids and lipid-like molecules	2.882±0.040	9.826±0.296	4.3776	2.1301	1.30	1.2048	Up
295.2280	12(13)-epoxy-9z-octadecenoic acid	Lipids and lipid-like	41.28±1.91	9.117±0.404	0.2830	-1.8209	1.58	1.3371	Down

		molecules			5				
331.2342	17alpha-hydroxyprogesterone	Lipids and lipid-like molecules	0.5421±0.0153	1.439±0.034	3.4025	1.7666	7.01	1.0749	Up
424.3061	17-phenyltrinorprostaglandin f2.alpha. cyclopropyl methyl amide	Lipids and lipid-like molecules	5.225±0.202	55.94±2.96	13.727	3.779	0.21	1.6751	Up
608.4658	1-lignoceroyl-2-hydroxy-sn-glycero-3-phosphocholine	Lipids and lipid-like molecules	0.09495±0.0092 8	2.124±0.095	28.706	4.8433	0.49	1.9193	Up
468.3087	1-myristoyl-sn-glycero-3-phosphoch oline	Lipids and lipid-like molecules	3.324±0.320	9.621±0.197	3.715	1.8934	5.68	1.1232	Up
740.5224	1-palmitoyl-2-oleoyl-sn-glycero-3-ph osphoethanolamine	Lipids and lipid-like molecules	152.4±2.8	427.4±13.5	3.5964	1.8466	1.71	1.1043	Up
546.3544	1-stearoyl-2-hydroxy-sn-glycero-3-p hosphocholine	Lipids and lipid-like molecules	0.5798±0.0442	3.650±0.217	8.0711	3.0128	3.12	1.4753	Up
594.3770	2-(5-oxovaleryl)phosphatidylcholine	Lipids and lipid-like molecules	#NUM!±0.00	0.05134±0.0015 4	14.12	3.8197	0.16	3.9462	Up
145.0506	2,2-Dimethylsuccinic acid	Lipids and lipid-like molecules	51.02±2.40	13.42±0.34	0.3366 6	-1.5706	4.12	1.2569	Down
349.1835	2,3-dinor-8-isoprostaglandin-f2.alpha .	Lipids and lipid-like molecules	8.059±0.380	3.091±0.123	0.4912 5	-1.0255	165.47	1.0650	Down
175.0612	2-Isopropylmalic acid	Lipids and lipid-like molecules	130.5±8.3	21.75±1.88	0.2129 9	-2.2311	38.54	1.4568	Down
716.5225	2-linoleoyl-1-palmitoyl-sn-glycero-3-phosphoethanolamine	Lipids and lipid-like molecules	24.45±0.72	80.76±2.85	4.2318	2.0813	0.94	1.1888	Up
630.3465	3-deoxyaconitine	Lipids and lipid-like molecules	1.019±0.072	0.3801±0.0097	0.4793	-1.061	68.66	1.0785	Down
543.2779	3-hydroxystanozolol glucuronide	Lipids and lipid-like molecules	4.153±0.075	0.5908±0.0297	0.1824 2	-2.4547	2.40	1.5198	Down
375.2244	5(s),14(r)-lipoxin b4	Lipids and lipid-like	0.3783±0.0330	1.061±0.103	3.6009	1.8484	184.83	1.1026	Up

		molecules							
241.2038	5alpha-pregnan-3,20-dione	Lipids and lipid-like molecules	0.3181±0.0041	1.069±0.075	4.3075	2.1069	4.24	1.1964	Up
251.1797	5-androsten-3.beta.,16.alpha.-diol-17-one	Lipids and lipid-like molecules	2.036±0.146	5.034±0.133	3.1643	1.6619	42.64	1.0357	Up
293.2123	9-oxo-10(e),12(e)-octadecadienoic acid	Lipids and lipid-like molecules	29.08±1.64	7.539±0.104	0.33241	-1.589	0.19	1.2641	Down
204.1229	Acetylcarnitine	Lipids and lipid-like molecules	351.1±8.8	129.8±10.0	0.4732	-1.0795	24.14	1.0854	Down
327.1781	Acitretin	Lipids and lipid-like molecules	17.95±0.61	6.806±0.148	0.48667	-1.039	0.22	1.0714	Down
303.2001	Aleuritic acid	Lipids and lipid-like molecules	10.88±0.36	35.26±0.99	4.1435	2.0509	1.40	1.1794	Up
255.1705	Beta-estradiol	Lipids and lipid-like molecules	0.3517±0.0240	1.841±0.030	6.7141	2.7472	4.50	1.4002	Up
411.2217	Betamethasone 9,11-epoxide 21-propionate	Lipids and lipid-like molecules	0.09691±0.00278	0.3039±0.0027	4.0226	2.0081	0.51	1.1635	Up
393.3154	Bis(2-ethylhexyl) adipate	Lipids and lipid-like molecules	23.94±0.90	55.80±1.55	2.9891	1.5797	0.00	1.0007	Up
628.3626	Bulleyaconi cine a	Lipids and lipid-like molecules	1.589±0.022	0.1860±0.0053	0.15022	-2.7348	1.21	1.5938	Down
405.1695	Chlormadinone acetate	Lipids and lipid-like molecules	4.894±0.230	#NUM!±0.00	0.040214	-4.6362	0.00	4.5792	Down
465.3043	Cholesteryl sulfate	Lipids and lipid-like molecules	0.4531±0.0143	2.115±0.025	5.9708	2.5779	1.68	1.3509	Up
407.2955	Cholic acid	Lipids and lipid-like molecules	2.081±0.074	26.93±0.47	16.61	4.054	0.16	1.7414	Up
443.2251	Cinobufagin	Lipids and lipid-like	398.0±5.5	120.7±5.1	0.3882	-1.3651	11.90	1.1886	Down

		molecules			1					
327.2179	Cis-4,7,10,13,16,19-docosahexaenoic acid	Lipids and lipid-like molecules	32.88±2.74	12.71±0.73	0.49524	-1.0138	51.01	1.0601	Down
187.1437	Costunolide	Lipids and lipid-like molecules	31.36±2.22	10.20±0.65	0.41751	-1.2601	17.21	1.1531	Down
149.0961	Cuminaldehyde	Lipids and lipid-like molecules	18.89±0.61	2.540±0.049	0.17208	-2.5389	0.25	1.5415	Down
206.1387	Dexpanthenol	Lipids and lipid-like molecules	3.317±0.229	0.3697±0.0123	0.14319	-2.804	0.84	1.6114	Down
423.1979	Fludrocortisone	Lipids and lipid-like molecules	0.4449±0.0044	1.382±0.065	3.9858	1.9949	6.06	1.1577	Up
427.1329	Gardenoside	Lipids and lipid-like molecules	0.3402±0.0176	3.936±0.105	14.82	3.8894	0.77	1.7029	Up
219.1744	Germacrone	Lipids and lipid-like molecules	9.224±0.502	2.892±0.143	0.40208	-1.3145	17.05	1.1707	Down
309.1675	Gestrinone	Lipids and lipid-like molecules	25.46±2.58	6.107±0.047	0.3081	-1.6985	10.58	1.2984	Down
283.1267	Gibberellic acid	Lipids and lipid-like molecules	3.917±0.106	1.444±0.169	0.47174	-1.0839	395.43	1.0846	Down
495.2601	Leukotriene d4	Lipids and lipid-like molecules	7.080±0.187	1.559±0.024	0.2826	-1.8232	0.70	1.3385	Down
520.3399	Lpc 18:2	Lipids and lipid-like molecules	3505±120	821.7±40.3	0.30011	-1.7365	2.38	1.3104	Down
365.1056	Maltose	Lipids and lipid-like molecules	22.27±0.81	56.14±1.44	3.2298	1.6914	1.60	1.0456	Up
209.1536	Methyl dihydrojasmonate	Lipids and lipid-like molecules	1.368±0.060	3.830±0.066	3.5852	1.8421	2.87	1.1041	Up
477.1693	Obacunone	Lipids and lipid-like	1.008±0.061	3.737±0.248	4.7455	2.2466	13.52	1.2438	Up

		molecules							
143.1077	Octanoic acid	Lipids and lipid-like molecules	6.343±0.254	1.979±0.149	0.40045	-1.3203	70.98	1.1733	Down
423.2583	Ophiobolin a	Lipids and lipid-like molecules	0.1194±0.0064	0.9042±0.0928	9.6965	3.2775	6.69	1.5463	Up
714.5078	Pe 34:2	Lipids and lipid-like molecules	23.99±1.61	105.7±4.6	5.6469	2.4975	7.09	1.3249	Up
742.5357	Pe 36:2	Lipids and lipid-like molecules	2.688±0.094	6.941±0.057	3.3074	1.7257	5.07	1.0597	Up
738.5080	Pe 36:4	Lipids and lipid-like molecules	179.7±9.2	714.1±28.3	5.0886	2.3473	5.31	1.2779	Up
639.4085	Phorbol 12-myristate 13-acetate	Lipids and lipid-like molecules	4.310±0.409	1.563±0.019	0.46474	-1.1055	59.03	1.0937	Down
833.5185	Pi 34:2	Lipids and lipid-like molecules	9.720±0.335	33.47±0.99	4.4109	2.1411	1.32	1.2095	Up
553.2965	Proscillaridin a	Lipids and lipid-like molecules	0.2954±0.0102	1.263±0.062	5.4778	2.4536	0.65	1.3111	Up
359.2405	Prostaglandin f2.alpha. 1,15-lactone	Lipids and lipid-like molecules	1.228±0.118	2.980±0.046	3.1183	1.6408	25.02	1.0248	Up
392.3312	Prostaglandin f2.alpha. diethylamide	Lipids and lipid-like molecules	7.903±0.704	43.35±1.83	7.0428	2.8161	0.86	1.4206	Up
173.0922	Thymol	Lipids and lipid-like molecules	4.775±0.488	1.054±0.113	0.28409	-1.8156	116.28	1.3340	Down
314.0640	2'-Deoxyadenosine 5'-monophosphate (dAMP)	Nucleosides, nucleotides, and analogues	#NUM!±0.00	0.3622±0.0433	22.288	4.4782	1.64	4.2285	Up
560.0795	Adenosine 5'-diphosphoribose	Nucleosides, nucleotides, and	0.1029±0.0001	6.061±0.114	75.377	6.2361	0.00	2.1969	Up

		analogues								
462.0669	Adenylosuccinate	Nucleosides, nucleotides, and analogues	0.8736±0.0803	0.02845±0.00273	0.041793	-4.5806	1.44	2.0135	Down
464.0819	Adenylosuccinic acid	Nucleosides, nucleotides, and analogues	0.9471±0.0109	0.05275±0.00154	0.071347	-3.809	0.30	1.8494	Down
558.0644	Adp-ribose	Nucleosides, nucleotides, and analogues	0.2528±0.0033	9.550±0.119	48.412	5.5973	0.02	2.0739	Up
304.0341	Cytidine 2',3'-cyclic phosphate	Nucleosides, nucleotides, and analogues	0.2886±0.0021	0.8441±0.0373	3.7487	1.9064	7.11	1.1265	Up
344.0401	Guanosine 3',5'-cyclic monophosphate	Nucleosides, nucleotides, and analogues	0.4630±0.0405	1.578±0.168	4.3614	2.1248	118.18	1.2033	Up
606.0745	Uridine diphosphate-n-acetylglucosamine	Nucleosides, nucleotides, and analogues	1.979±0.106	5.488±0.320	3.5525	1.8289	2.41	1.0986	Up
86.0603	.gamma.-aminobutyric acid	Organic acids and derivatives	753.1±36.7	186.2±6.1	0.31724	-1.6563	0.35	1.2860	Down
348.0394	4-hydroxytriamterene sulfate	Organic acids and derivatives	#NUM!±0.00	2.557±0.128	79.107	6.3057	0.02	4.4945	Up
117.9968	Aminomalonic acid	Organic acids and derivatives	0.06721±0.00253	0.4411±0.0292	8.4023	3.0708	2.70	1.4916	Up
231.0977	Asp-Pro	Organic acids and derivatives	8.158±0.549	3.082±0.065	0.48508	-1.0437	15.56	1.0722	Down
424.2171	Calpain inhibitor ii	Organic acids and	0.1329±0.0038	0.3702±0.0333	3.5763	1.8385	62.95	1.0976	Up

		derivatives							
240.0656	Captopril	Organic acids and derivatives	0.01295±0.00072	0.5494±0.0286	54.265	5.7619	0.43	2.1067	Up
248.0930	Cys-Gln	Organic acids and derivatives	1.197±0.014	11.49±0.57	12.27	3.6171	0.17	1.6365	Up
441.2095	Cys-Tyr-Arg	Organic acids and derivatives	33.66±0.53	6.208±0.466	0.23646	-2.0803	10.63	1.4149	Down
312.9849	Dicloxacillin	Organic acids and derivatives	5.134±0.174	1.670±0.082	0.4167	-1.2629	28.89	1.1523	Down
130.0496	D-pyroglutamic acid	Organic acids and derivatives	4.048±0.102	24.01±1.45	7.6065	2.9272	0.94	1.4509	Up
295.1291	gamma-L-Glutamyl-L-phenylalanine	Organic acids and derivatives	3.132±0.130	9.324±0.525	3.814	1.9313	1.02	1.1362	Up
275.1352	Gln-gln	Organic acids and derivatives	14.97±0.47	4.603±0.137	0.39352	-1.3455	12.18	1.1816	Down
304.1619	Glu-Arg	Organic acids and derivatives	1.076±0.047	5.041±0.375	6.0112	2.5876	4.07	1.3509	Up
407.1891	Glu-Met-Lys	Organic acids and derivatives	0.06948±0.00497	0.5139±0.0143	9.4691	3.2432	2.61	1.5395	Up
613.1598	Glutathione, oxidized	Organic acids and derivatives	22.74±1.43	2.788±0.047	0.15711	-2.6702	0.31	1.5760	Down
334.1401	Glu-Trp	Organic acids and derivatives	0.2474±0.0251	2.505±0.035	12.929	3.6925	4.90	1.6567	Up
459.2199	Hc toxin	Organic acids and derivatives	9.466±0.938	1.103±0.056	0.14918	-2.7448	20.87	1.5947	Down
229.1546	Ile-Pro	Organic acids and derivatives	51.41±2.83	15.34±0.90	0.38224	-1.3875	4.73	1.1966	Down
318.1815	Ile-Trp	Organic acids and	2.945±0.165	7.596±0.122	3.3053	1.7248	0.54	1.0598	Up

		derivatives							
212.0058	Indoxyl sulfate	Organic acids and derivatives	#NUM!±0.00	0.4371±0.0117	7.2785	2.8636	0.30	4.2555	Up
173.0092	Isocitrate	Organic acids and derivatives	43.81±3.11	17.09±0.47	0.49992	-1.0002	6.77	1.0550	Down
177.1125	L-canavanine	Organic acids and derivatives	3.280±0.171	1.025±0.082	0.40174	-1.3157	129.69	1.1719	Down
427.0956	L-cysteine-glutathione disulfide	Organic acids and derivatives	1.321±0.053	0.1265±0.0055	0.12287	-3.0248	2.47	1.6666	Down
128.0353	L-pyroglutamic acid	Organic acids and derivatives	503.0±9.0	1351±40	3.4466	1.7852	1.16	1.0811	Up
102.0550	N-(.beta.-ketocaproyl)-dl-homoserine lactone	Organic acids and derivatives	5.447±0.417	1.642±0.079	0.38598	-1.3734	44.80	1.1891	Down
154.0976	N-acetylhistamine	Organic acids and derivatives	18.18±1.11	45.63±1.14	3.2164	1.6855	0.27	1.0442	Up
210.1338	N-octanoyl-l-homoserine lactone	Organic acids and derivatives	5.907±0.584	1.000±0.075	0.21713	-2.2034	9.54	1.4496	Down
118.0863	Norvaline	Organic acids and derivatives	80.96±8.27	3.223±0.119	0.051092	-4.2907	0.57	1.9527	Down
277.1228	Pantetheine	Organic acids and derivatives	120.2±8.3	0.5447±0.0095	0.005805	-7.4284	0.01	2.5277	Down
577.2337	Pantethine	Organic acids and derivatives	0.3166±0.0087	0.8696±0.0179	3.5222	1.8165	1.41	1.0936	Up
297.1270	Phe-met	Organic acids and derivatives	0.8858±0.0198	2.120±0.143	3.0653	1.616	13.19	1.0145	Up
381.2111	Phe-Ser-Lys	Organic acids and derivatives	0.4416±0.0303	1.385±0.045	4.0291	2.0105	5.75	1.1634	Up
352.1657	Phe-trp	Organic acids and	0.3841±0.0288	1.836±0.034	6.1312	2.6162	1.22	1.3618	Up

		derivatives							
359.1829	Pyroglu-thr-lys	Organic acids and derivatives	0.1030±0.0050	0.8164±0.0487	10.179	3.3476	3.96	1.5654	Up
461.1982	Quinapril	Organic acids and derivatives	2.244±0.094	0.8360±0.0240	0.4771 3	-1.0675	45.57	1.0806	Down
88.0404	Sarcosine	Organic acids and derivatives	315.2±3.7	85.67±4.17	0.3481	-1.5224	1.73	1.2420	Down
429.2507	Trandolapril	Organic acids and derivatives	0.4449±0.0171	1.282±0.020	3.7012	1.888	1.63	1.1195	Up
421.2426	Tris(2-butoxyethyl) phosphate	Organic acids and derivatives	#NUM!±0.00	0.5130±0.0641	18.133	4.1805	2.33	4.2769	Up
302.1502	Trp-Pro	Organic acids and derivatives	15.80±1.59	3.036±0.306	0.2466 2	-2.0196	31.77	1.3972	Down
238.1286	3-(cyclohexylamino)-2-hydroxy-1-pr opanesulfonic acid	Organic nitrogen compounds	730.5±22.8	129.5±4.3	0.2275 5	-2.1358	0.03	1.4313	Down
134.1176	Ethyldiethanolamine	Organic nitrogen compounds	4.197±0.188	1.089±0.136	0.3329 1	-1.5868	219.92	1.2622	Down
262.1288	Methapyrilene	Organic nitrogen compounds	38.11±1.16	4.623±0.095	0.1555 9	-2.6841	0.07	1.5804	Down
184.0733	Miltefosine	Organic nitrogen compounds	143.1±1.7	25.51±0.72	0.2279 6	-2.1332	0.07	1.4291	Down
206.1653	N1-(1-methyl-4-piperidinyl)-1,4-benz enediamine	Organic nitrogen compounds	#NUM!±0.00	0.2225±0.0080	81.075	6.3412	0.02	4.1604	Up
516.3030	Oleyloxyethylphosphorylcholine	Organic nitrogen compounds	#NUM!±0.00	0.1722±0.0067	15.169	3.9231	0.17	4.1237	Up
421.0754	.alpha.,.alpha.'-trehalose 6-phosphate	Organic oxygen compounds	9.040±0.199	2.950±0.049	0.4188 3	-1.2556	0.71	1.1515	Down
290.2076	1-heptanone,	Organic oxygen	1.449±0.049	8.132±0.078	7.2009	2.8482	0.58	1.4292	Up

	1-(4-methoxyphenyl)-2-(1-pyrrolidinyl)-	compounds								
151.0519	3,4-dihydroxyacetophenone	Organic oxygen compounds	#NUM!±0.00	2.328±0.280	7.3006	2.868	8.17	4.4815	Up
535.1518	3-deoxy-d-glycero-d-galacto-2-nonulosonic acid	Organic oxygen compounds	6.737±0.596	1.806±0.109	0.34415	-1.5389	23.37	1.2477	Down
489.1695	5-azacytidine	Organic oxygen compounds	6.862±0.231	19.20±1.47	3.5864	1.8425	3.26	1.1021	Up
385.1313	Catalpol	Organic oxygen compounds	0.1245±0.0096	0.3144±0.0101	3.2378	1.695	5.77	1.0472	Up
365.1062	Coniferin	Organic oxygen compounds	10.07±0.18	3.162±0.160	0.40307	-1.3109	12.34	1.1711	Down
540.0540	Cyclic adenosine diphosphate ribose	Organic oxygen compounds	18.95±1.12	6.701±0.334	0.45369	-1.1402	158.07	1.1087	Down
259.0130	D-mannose 6-phosphate	Organic oxygen compounds	65.47±2.22	20.78±1.45	0.40738	-1.2956	66.21	1.1652	Down
229.0354	D-ribose 1-phosphate	Organic oxygen compounds	27.59±2.41	4.677±0.422	0.21687	-2.2051	31.62	1.4477	Down
339.0042	Fructose 1,6-diphosphate	Organic oxygen compounds	0.3248±0.0053	1.110±0.043	4.3746	2.1292	2.68	1.2059	Up
637.1533	Leiocarposide	Organic oxygen compounds	0.09020±0.00712	0.2544±0.0287	3.6105	1.8522	102.95	1.1025	Up
447.1588	N,n'-diacetylchitobiose	Organic oxygen compounds	0.9065±0.0434	8.411±0.125	11.894	3.5721	0.13	1.6245	Up
465.1696	N-acetylglucosamine	Organic oxygen compounds	0.4625±0.0143	1.873±0.137	5.1975	2.3778	2.77	1.2858	Up
251.0776	Orcinol .beta.-d-glucoside	Organic oxygen compounds	24.30±2.37	6.833±0.192	0.36066	-1.4713	22.29	1.2235	Down

499.1645	Primeverin	Organic oxygen compounds	0.1147±0.0064	0.3728±0.0228	4.1816	2.0641	8.53	1.1807	Up
195.1227	Tetraethylene glycol	Organic oxygen compounds	65.47±5.85	18.28±0.95	0.35896	-1.4781	30.27	1.2267	Down
151.0965	Triethylene glycol monobutyl ether	Organic oxygen compounds	5.721±0.159	2.025±0.219	0.4538	-1.1399	416.46	1.1071	Down
189.1236	.alpha.-ethyltryptamine	Organoheterocyclic compounds	5.798±0.238	126.5±8.3	27.958	4.8052	0.16	1.9100	Up
204.0689	1-(2,8-dihydroxyquinolin-5-yl)ethan-1-one	Organoheterocyclic compounds	29.36±0.75	5.783±0.451	0.25277	-1.9841	14.83	1.3868	Down
363.0928	1-methyluric acid	Organoheterocyclic compounds	0.2318±0.0155	1.079±0.023	5.9516	2.5733	2.45	1.3491	Up
217.1046	2-(2',3',4'-trihydroxybutyl)quinoxaline	Organoheterocyclic compounds	29.76±0.62	4.514±0.301	0.19451	-2.362	5.27	1.4946	Down
557.2573	4-hydroxyatorvastatin lactone	Organoheterocyclic compounds	31.03±1.89	6.540±0.554	0.27053	-1.8862	28.87	1.3565	Down
144.0302	4-hydroxyquinoline	Organoheterocyclic compounds	35.56±1.21	11.38±0.36	0.4093	-1.2888	0.22	1.1614	Down
208.0619	4-morpholinopropanesulfonic acid	Organoheterocyclic compounds	0.3112±0.0026	0.7607±0.0623	3.1322	1.6472	25.45	1.0250	Up
213.0172	8-chlorotheophylline	Organoheterocyclic compounds	33.76±0.71	5.087±0.032	0.1931	-2.3726	0.10	1.4971	Down
152.0820	8-methylcaffeine	Organoheterocyclic compounds	110.2±8.4	31.92±2.40	0.37108	-1.4302	21.81	1.2110	Down
266.1114	Albendazole	Organoheterocyclic compounds	0.07488±0.00235	0.4846±0.0083	8.2918	3.0517	1.09	1.4873	Up
228.1343	Ametryne	Organoheterocyclic compounds	7.977±0.209	2.085±0.197	0.3352	-1.5769	26.99	1.2604	Down

129.0408	Ammelide	Organoheterocyclic compounds	#NUM!±0.00	2.682±0.127	15.21	3.9269	0.11	4.5008	Up
320.0989	Amoxicillin	Organoheterocyclic compounds	0.5811±0.0047	0.2217±0.0068	0.48886	-1.0325	44.88	1.0682	Down
567.1683	Benzeneacetamide, 4-(4,9-diethoxy-1,3-dihydro-1-oxo-2h-benz[f]isoindol-2-yl)-n-(phenylsulfonyl)-	Organoheterocyclic compounds	0.1589±0.0153	0.4641±0.0473	3.7476	1.906	104.14	1.1204	Up
195.0877	Caffeine	Organoheterocyclic compounds	28.88±1.48	6.057±0.753	0.26858	-1.8966	37.39	1.3604	Down
214.0687	Carbendazim	Organoheterocyclic compounds	6.551±0.144	2.374±0.036	0.46433	-1.1068	8.99	1.0962	Down
217.0973	Carboline base + 4h, carboxylic acid	Organoheterocyclic compounds	0.5937±0.0057	1.868±0.041	4.0282	2.0101	1.60	1.1649	Up
356.1559	Difloxacin	Organoheterocyclic compounds	0.7872±0.0790	0.01899±0.00138	0.030934	-5.0146	1.04	2.0990	Down
399.0093	Ethiprole	Organoheterocyclic compounds	2.367±0.072	0.2405±0.0073	0.13025	-2.9406	0.20	1.6455	Down
236.0587	Ethyl 8-fluoro-4-hydroxyquinoline-3-carboxylate	Organoheterocyclic compounds	0.1845±0.0032	0.9891±0.0144	6.8572	2.7776	0.46	1.4102	Up
303.1054	Hematoxylin	Organoheterocyclic compounds	8.928±0.746	2.612±0.121	0.37505	-1.4149	17.38	1.2056	Down
254.1501	Irgarol	Organoheterocyclic compounds	3.333±0.250	0.9151±0.0204	0.35152	-1.5083	76.20	1.2348	Down
427.2302	Lovatatin	Organoheterocyclic compounds	264.0±5.4	21.32±1.43	0.10345	-3.273	3.22	1.7265	Down
248.1494	Meperidine	Organoheterocyclic	43.84±3.25	153.0±17.8	4.4693	2.16	78.57	1.2128	Up

		compounds							
350.2691	Methanone, (1-pentyl-1h-indol-3-yl)tricyclo[3.3.1.13,7]dec-1-yl-	Organoheterocyclic compounds	0.4134±0.0140	1.345±0.035	4.1616	2.0571	4.99	1.1817	Up
326.1236	N-desmethyldanofloxacin	Organoheterocyclic compounds	2.233±0.148	0.4777±0.0099	0.27454	-1.8649	1.30	1.3507	Down
153.0660	Nudifloramide	Organoheterocyclic compounds	0.3175±0.0224	1.922±0.022	7.7551	2.9551	0.74	1.4607	Up
181.0720	Paraxanthine	Organoheterocyclic compounds	39.01±2.55	8.093±0.159	0.26634	-1.9087	0.97	1.3640	Down
168.0656	Pyridoxal	Organoheterocyclic compounds	30.00±0.74	11.37±1.17	0.48503	-1.0438	217.59	1.0706	Down
170.0812	Pyridoxine	Organoheterocyclic compounds	461.3±23.7	138.1±5.7	0.38312	-1.3841	30.12	1.1940	Down
144.0667	Quinolin-2-ol	Organoheterocyclic compounds	#NUM!±0.00	0.9948±0.0478	6.9655	2.8002	0.17	4.3683	Up
131.0454	Quinoxaline	Organoheterocyclic compounds	8.722±0.827	3.097±0.024	0.45576	-1.1336	46.94	1.1052	Down
111.0200	Uracil	Organoheterocyclic compounds	185.4±16.1	15.91±0.85	0.11008	-3.1833	0.76	1.7047	Down
235.1190	Zolpidem	Organoheterocyclic compounds	7.011±0.342	35.89±0.85	6.5614	2.714	0.02	1.3908	Up
217.0973	4,4'-thiodianiline	Organosulfur compounds	3.238±0.091	38.57±1.43	15.278	3.9334	0.04	1.7127	Up
485.2356	(2r,3r,4s,5s,6r)-2-[1,7-bis(4-hydroxyphenyl)heptan-3-yloxy]-6-(hydroxymethyl)oxane-3,4,5-triol	Phenylpropanoids and polyketides	19.66±0.50	1.383±0.097	0.090407	-3.4674	1.20	1.7730	Down
377.0757	3',4',5,7-tetrahydroxy-3,6,8-trimethox	Phenylpropanoids and	0.6035±0.0505	1.775±0.210	3.7577	1.9098	140.65	1.1225	Up

	yflavone	polyketides							
313.0911	3,7,3'-trimethoxyflavone	Phenylpropanoids and polyketides	5.687±0.199	1.883±0.066	0.42492	-1.2347	11.49	1.1436	Down
249.0405	7,8-dihydroxy-4-methylcoumarin-3-acetic acid	Phenylpropanoids and polyketides	2.002±0.025	0.6745±0.0167	0.43177	-1.2117	13.94	1.1350	Down
358.0909	Casticin	Phenylpropanoids and polyketides	0.08054±0.00498	0.3412±0.0116	5.4312	2.4413	4.02	1.3073	Up
530.2593	Epothilone b	Phenylpropanoids and polyketides	#NUM!±0.00	2.023±0.197	21.413	4.4204	0.75	4.4632	Up
615.1714	Fortunellin	Phenylpropanoids and polyketides	0.4049±0.0053	0.9791±0.0651	3.0992	1.6319	28.97	1.0199	Up
285.0696	Maackiaine	Phenylpropanoids and polyketides	2.729±0.158	7.062±0.113	3.3212	1.7317	0.55	1.0616	Up
395.1663	Rotenone	Phenylpropanoids and polyketides	2.952±0.128	17.59±0.73	7.6504	2.9355	0.17	1.4534	Up
681.1296	Rutarensin	Phenylpropanoids and polyketides	#NUM!±0.00	0.2414±0.0081	19.758	4.3044	0.16	4.1720	Up
463.1327	Tectoridin	Phenylpropanoids and polyketides	0.8737±0.0223	4.379±0.397	6.4165	2.6818	3.86	1.3796	Up
