# Supplementary material for: Untargeted Metabolomic Analysis Combined with Chemometrics Revealed the Effects of Different Cooking Methods on Lentinus edodes
Source: Molecules. 2023 Aug 11;28(16):6009. doi: 10.3390/molecules28166009 (PMC10458448; doi:10.3390/molecules28166009)
Supplement: Supplementary file 1 [file molecules-28-06009-s001.zip › Table S5.pdf]

Table S5 List of differential metabolites between Air-frying and Control

m/z	Name	SuperClass	Area(10^7) Control	Area(10^7) Air-frying	Fold Change (FC)	log2(FC)	P value (10^-6)	VIP	Type
324.1899	Lsd	Alkaloids and derivatives	4.147±0.282	10.83±0.73	3.1742	1.6664	5.89	1.058	Up
421.1222	(5-benzoyloxy-4,6-dihydroxy-3-methoxy cyclohexen-1-yl)methyl benzoate	Benzenoids	2.321±0.109	11.91±0.84	6.2478	2.6433	0.52	1.381	Up
145.0609	.beta.-naphthol	Benzenoids	11.49±0.40	2.589±0.228	0.27423	-1.8665	69.50	1.319	Down
253.1799	1-benzhydrylpiperazine	Benzenoids	17.46±1.59	6.217±0.427	0.4333	-1.2065	64.70	1.096	Down
178.0719	2,2-bis(4-chlorophenyl)ethanol	Benzenoids	31.19±2.23	3.200±0.204	0.12497	-3.0003	0.17	1.630	Down
188.9355	2,4-dichlorobenzoic acid	Benzenoids	14.22±0.48	104.6±2.7	8.9783	3.1664	0.18	1.526	Up
110.0601	2-aminophenol	Benzenoids	3.251±0.268	22.85±0.74	8.5805	3.1011	1.44	1.508	Up
208.0968	2-chloro-2',6'-diethylacetanilide	Benzenoids	0.06858±0.00637	0.4802±0.0339	8.5769	3.1005	9.87	1.505	Up
168.0306	2-methoxy-5-nitrophenol	Benzenoids	2.312±0.039	0.6400±0.0114	0.33742	-1.5674	20.24	1.224	Down
227.1754	3,3'-dimethyl-4,4'-diaminodiphenylmethane	Benzenoids	1.819±0.024	47.17±3.58	31.599	4.9818	0.01	1.949	Up
256.0827	3-hydroxymethylmefenamic acid	Benzenoids	641.1±41.8	206.7±22.7	0.39218	-1.3504	18.96	1.151	Down
245.1536	Amprenavir	Benzenoids	2.036±0.036	4.601±0.397	2.7473	1.458	8.00	2.203	Up
266.1250	Anisomycin	Benzenoids	38.84±1.62	11.27±1.39	0.35276	-1.5032	41.71	1.202	Down
377.0693	Benzenepropanamide, n-(6-chloro-2-benzothiazolyl)-3,4-dimethoxy-	Benzenoids	18.02±1.15	5.635±0.256	0.38183	-1.389	12.37	1.164	Down
354.9838	Benzenesulfonic acid, 2-[(5-bromo-2-hydroxyphenyl)methylene]hydrazide	Benzenoids	0.3494±0.0184	0.8438±0.0049	2.9384	1.555	50.43	1.014	Up
397.1157	Daunomycinone	Benzenoids	8.189±0.147	2.136±0.065	0.31793	-1.6532	0.44	1.252	Down
441.3075	Di(2-nonyl) phthalate	Benzenoids	3.346±0.199	0.7215±0.0137	0.26336	-1.9249	2.63	1.338	Down

317.1112	Dibutyl phthalate	Benzenoids	0.1847±0.0059	1.936±0.023	12.772	3.6749	0.71	1.656	Up
386.1065	Flamprop-isopropyl	Benzenoids	0.1580±0.0035	3.415±0.168	26.4	4.7225	0.43	1.894	Up
152.0562	Flufenacet	Benzenoids	5.139±0.223	39.45±2.02	9.3616	3.2267	0.05	1.542	Up
297.0882	Flunixin	Benzenoids	7.740±0.172	2.291±0.038	0.36065	-1.4713	1.45	1.192	Down
316.1006	Flusilazole	Benzenoids	#NUM!±0.00	54.76±2.71	2508.6	11.293	0.00	4.846	Up
494.2590	Imatinib	Benzenoids	#NUM!±0.00	2.200±0.047	44.814	5.4859	0.02	4.442	Up
412.1104	Mandipropamid	Benzenoids	7.696±0.137	1.976±0.044	0.31293	-1.6761	1.71	1.260	Down
343.1615	Methanone, 1-naphthalenyl(1-pentyl-1h-indazol-3-yl)-	Benzenoids	0.1111±0.0061	1.433±0.042	15.7	3.9727	0.40	1.728	Up
215.0562	Nepodin	Benzenoids	25.51±0.82	9.449±0.633	0.45071	-1.1497	77.42	1.075	Down
184.0616	N-hydroxy-4-aminobiphenyl	Benzenoids	10.01±0.82	65.27±2.37	7.9502	2.991	0.43	1.480	Up
387.1046	Pamoic acid	Benzenoids	3.251±0.152	7.923±0.647	2.9655	1.5683	7.68	1.018	Up
475.2152	Sildenafil	Benzenoids	4.171±0.310	1.546±0.148	0.45039	-1.1508	29.11	1.077	Down
387.1992	Sufentanyl	Benzenoids	4.780±0.105	1.740±0.047	0.44281	-1.1752	7.86	1.086	Down
405.1483	Sulfinpyrazone	Benzenoids	1.019±0.043	6.106±0.228	7.326	2.873	7.54	1.446	Up
223.1443	Zectran	Benzenoids	0.04378±0.00111	10.42±1.23	288.6	8.1729	0.05	2.526	Up
488.2518	(2e,6e,11e,13e)-18-(2,6-dioxopiperidin-4-yl)-9-hydroxy-8-methoxy-10,12,14-trimethyl-15-oxooctadeca-2,6,11,13-tetraenoic acid	Lipids and lipid-like molecules	#NUM!±0.00	0.2071±0.0091	11.788	3.5592	1.84	4.120	Up
311.2229	(9z,12e)-15,16-dihydroxyoctadeca-9,12-dienoic acid	Lipids and lipid-like molecules	117.8±2.1	17.17±0.58	0.17807	-2.4895	0.09	1.500	Down
111.0805	.alpha.-cyperone	Lipids and lipid-like molecules	3.289±0.134	0.8463±0.0049	0.31377	-1.6722	19.86	1.258	Down
502.3292	1-(1z-hexadecenyl)-sn-glycero-3-phosphocholine	Lipids and lipid-like molecules	15.64±0.60	3.710±0.304	0.28854	-1.7931	4.40	1.296	Down
476.2782	1-(9z,12z-octadecadienoyl)-2-hydroxy-sn	Lipids and lipid-like	308.4±14.1	95.35±4.19	0.37707	-1.4071	114.91	1.170	Down

	-glycero-3-phosphoethanolamine	molecules								
401.2165	1,2-dihydrodesoxymetasone	Lipids and lipid-like molecules	7.729±0.370	2.322±0.120	0.36606	-1.4498	160.49	1.185	Down
688.4907	1,2-dipalmitoleoyl-sn-glycero-3-phospho ethanolamine	Lipids and lipid-like molecules	2.882±0.040	10.62±0.32	4.505	2.1715	7.25	1.233	Up
295.2280	12(13)-epoxy-9z-octadecenoic acid	Lipids and lipid-like molecules	41.28±1.91	8.591±0.300	0.25356	-1.9796	2.22	1.353	Down
297.1675	17alpha-ethynylestradiol	Lipids and lipid-like molecules	17.11±1.51	5.977±0.194	0.42725	-1.2269	112.39	1.105	Down
331.2342	17alpha-hydroxyprogesterone	Lipids and lipid-like molecules	0.5421±0.0153	1.647±0.172	3.6885	1.883	10.75	1.136	Up
424.3061	17-phenyltrinorprostaglandin f2.alpha. cyclopropyl methyl amide	Lipids and lipid-like molecules	5.225±0.202	20.24±2.16	4.7141	2.237	1.43	1.256	Up
608.4658	1-lignoceroyl-2-hydroxy-sn-glycero-3-ph osphocholine	Lipids and lipid-like molecules	0.09495±0.00928	1.752±0.094	22.468	4.4898	0.44	1.845	Up
740.5224	1-palmitoyl-2-oleoyl-sn-glycero-3-phosp hoethanolamine	Lipids and lipid-like molecules	152.4±2.8	450.2±14.8	3.603	1.8492	7.28	1.124	Up
546.3544	1-stearoyl-2-hydroxy-sn-glycero-3-phosp hocholine	Lipids and lipid-like molecules	0.5798±0.0442	1.427±0.174	2.99	1.5802	37.53	1.024	Up
145.0506	2,2-Dimethylsuccinic acid	Lipids and lipid-like molecules	51.02±2.40	14.52±0.32	0.34652	-1.529	12.50	1.210	Down
175.0612	2-Isopropylmalic acid	Lipids and lipid-like molecules	130.5±8.3	24.46±0.80	0.22745	-2.1364	13.22	1.397	Down
716.5225	2-linoleoyl-1-palmitoyl-sn-glycero-3-pho sphoethanolamine	Lipids and lipid-like molecules	24.45±0.72	84.12±3.12	4.1952	2.0687	8.63	1.200	Up
577.2837	3-deacetylsalannin	Lipids and lipid-like molecules	1.647±0.066	0.5468±0.0179	0.40545	-1.3024	57.11	1.134	Down
543.2779	3-hydroxystanozolol glucuronide	Lipids and lipid-like	4.153±0.075	0.6574±0.0185	0.19317	-2.3721	4.36	1.467	Down

		molecules								
297.2403	7,8-dehydropregnenolone	Lipids and lipid-like molecules	0.3977±0.0071	0.1241±0.0084	0.37951	-1.3978	4.68	1.166	Down
293.2123	9-oxo-10(e),12(e)-octadecadienoic acid	Lipids and lipid-like molecules	29.08±1.64	8.290±0.683	0.347	-1.527	8.28	1.211	Down
810.1338	Acetyl-coa	Lipids and lipid-like molecules	1.311±0.039	0.2102±0.0133	0.19493	-2.359	0.82	1.462	Down
327.1781	Acitretin	Lipids and lipid-like molecules	17.95±0.61	5.360±0.228	0.36516	-1.4534	51.37	1.187	Down
255.1705	Beta-estradiol	Lipids and lipid-like molecules	0.3517±0.0240	1.887±0.108	6.5226	2.7054	3.38	1.400	Up
411.2217	Betamethasone 9,11-epoxide 21-propionate	Lipids and lipid-like molecules	0.0969±0.0028	0.4348±0.0414	5.4608	2.4491	2.07	1.322	Up
628.3626	Bulleyaconi cine a	Lipids and lipid-like molecules	1.589±0.022	0.3468±0.0040	0.26628	-1.909	7.19	1.333	Down
405.1695	Chlormadinone acetate	Lipids and lipid-like molecules	4.894±0.230	1.438±0.115	0.35814	-1.4814	0.78	1.196	Down
465.3043	Cholesteryl sulfate	Lipids and lipid-like molecules	0.4531±0.0143	1.340±0.030	3.6024	1.849	17.68	1.125	Up
407.2955	Cholic acid	Lipids and lipid-like molecules	2.081±0.074	12.33±0.90	7.2183	2.8516	0.80	1.440	Up
443.2251	Cinobufagin	Lipids and lipid-like molecules	398.0±5.5	156.7±10.2	0.47949	-1.0604	8.64	1.043	Down
149.0961	Cuminaldehyde	Lipids and lipid-like molecules	18.89±0.61	2.637±0.031	0.16983	-2.5579	0.32	1.516	Down
293.2113	Desogestrel	Lipids and lipid-like molecules	1.419±0.014	0.3998±0.0352	0.34451	-1.5374	84.31	1.215	Down
206.1387	Dexpanthenol	Lipids and lipid-like	3.317±0.229	15.78±0.70	5.7992	2.5358	0.30	1.349	Up

		molecules							
297.1675	Exemestane	Lipids and lipid-like molecules	3.977±0.077	1.591±0.060	0.48786	-1.0355	21.31	1.034	Down
423.1979	Fludrocortisone	Lipids and lipid-like molecules	0.4449±0.0044	3.240±0.065	8.8658	3.1483	0.52	1.522	Up
427.1329	Gardenoside	Lipids and lipid-like molecules	0.3402±0.0176	1.306±0.053	4.6577	2.2196	6.12	1.253	Up
219.1744	Germacrone	Lipids and lipid-like molecules	9.224±0.502	3.315±0.342	0.44	-1.1844	650.04	1.091	Down
309.1675	Gestrinone	Lipids and lipid-like molecules	25.46±2.58	5.133±0.163	0.24635	-2.0212	17.33	1.365	Down
283.1267	Gibberellic acid	Lipids and lipid-like molecules	3.917±0.106	1.377±0.144	0.42688	-1.2281	42.10	1.104	Down
315.1675	Gibberellin a9	Lipids and lipid-like molecules	0.8112±0.0429	3.660±0.141	5.4968	2.4586	14.57	1.326	Up
495.2601	Leukotriene d4	Lipids and lipid-like molecules	7.080±0.187	1.137±0.123	0.1963	-2.3489	39.83	1.462	Down
########	Linoleoyl-CoA	Lipids and lipid-like molecules	0.4858±0.0376	0.1925±0.0204	0.48406	-1.0467	2522.9 0	1.040	Down
520.3399	Lpc 18:2	Lipids and lipid-like molecules	3505±120	710.1±68.9	0.24605	-2.023	3.05	1.365	Down
365.1056	Maltose	Lipids and lipid-like molecules	22.27±0.81	58.95±5.72	3.2164	1.6854	6.39	1.063	Up
143.1077	Octanoic acid	Lipids and lipid-like molecules	6.343±0.254	1.633±0.129	0.31316	-1.675	0.35	1.259	Down
423.2583	Ophiobolin a	Lipids and lipid-like molecules	0.1194±0.0064	1.586±0.154	16.142	4.0127	0.89	1.737	Up
309.1295	Paclitaxel	Lipids and lipid-like	2.999±0.285	29.49±1.50	11.947	3.5786	4.83	1.633	Up

		molecules							
714.5078	Pe 34:2	Lipids and lipid-like molecules	23.99±1.61	107.6±7.6	5.4826	2.4549	32.59	1.323	Up
742.5357	Pe 36:2	Lipids and lipid-like molecules	2.688±0.094	7.181±0.677	3.2681	1.7085	210.69	1.068	Up
738.5080	Pe 36:4	Lipids and lipid-like molecules	179.7±9.2	684.5±27.7	4.6412	2.2145	14.32	1.249	Up
639.4085	Phorbol 12-myristate 13-acetate	Lipids and lipid-like molecules	4.310±0.409	1.057±0.107	0.29828	-1.7453	25.18	1.281	Down
833.5185	Pi 34:2	Lipids and lipid-like molecules	9.720±0.335	27.29±0.80	3.4222	1.7749	6.81	1.097	Up
553.2965	Proscillaridin a	Lipids and lipid-like molecules	0.2954±0.0102	3.507±0.078	14.471	3.8551	0.03	1.699	Up
359.2405	Prostaglandin f2.alpha. 1,15-lactone	Lipids and lipid-like molecules	1.228±0.118	3.345±0.060	3.3299	1.7355	25.44	1.081	Up
392.3312	Prostaglandin f2.alpha. diethylamide	Lipids and lipid-like molecules	7.903±0.704	44.69±3.51	6.8858	2.7836	0.98	1.422	Up
201.1133	Sebacic acid	Lipids and lipid-like molecules	4.789±0.092	16.18±0.59	4.1151	2.0409	5.01	1.191	Up
143.0817	Succinic acid n,n-dimethylhydrazide	Lipids and lipid-like molecules	0.1924±0.0130	26.10±0.67	165.46	7.3703	0.02	2.394	Up
173.0922	Thymol	Lipids and lipid-like molecules	4.775±0.488	1.763±0.085	0.45046	-1.1505	52.99	1.076	Down
311.1468	Trans-crocetin	Lipids and lipid-like molecules	4.704±0.393	0.8295±0.0390	0.21492	-2.2181	6.03	1.422	Down
314.0640	2'-Deoxyadenosine 5'-monophosphate (dAMP)	Nucleosides, nucleotides, and analogues	#NUM!±0.00	0.5566±0.0351	32.551	5.0246	0.05	4.258	Up
560.0795	Adenosine 5'-diphosphoribose	Nucleosides, nucleotides,	0.1029±0.0001	0.5506±0.0068	6.5151	2.7038	1.45	1.399	Up

		and analogues							
346.0557	Adenosine 5'-phosphosulfate	Nucleosides, nucleotides, and analogues	28.60±0.89	69.60±3.87	2.9674	1.5692	22.88	1.017	Up
462.0669	Adenylosuccinate	Nucleosides, nucleotides, and analogues	0.8736±0.0803	0.1595±0.0050	0.2229	-2.1655	6.98	1.407	Down
464.0819	Adenylosuccinic acid	Nucleosides, nucleotides, and analogues	0.9471±0.0109	0.1741±0.0113	0.22355	-2.1613	3.48	1.406	Down
558.0644	Adp-ribose	Nucleosides, nucleotides, and analogues	0.2528±0.0033	0.7768±0.0613	3.7548	1.9087	74.99	1.143	Up
304.0341	Cytidine 2',3'-cyclic phosphate	Nucleosides, nucleotides, and analogues	0.2886±0.0021	1.029±0.069	4.3514	2.1215	32.64	1.217	Up
588.0753	Gdp-l-fucose	Nucleosides, nucleotides, and analogues	0.4848±0.0165	1.287±0.058	3.2242	1.6889	0.46	1.067	Up
344.0401	Guanosine 3',5'-cyclic monophosphate	Nucleosides, nucleotides, and analogues	0.4630±0.0405	2.697±0.074	7.0852	2.8248	17.63	1.435	Up
809.0125	Uridine 5'-diphosphate (UDP)	Nucleosides, nucleotides, and analogues	11.87±0.42	4.021±0.197	0.41261	-1.2772	0.02	1.124	Down
290.0859	Zidovudine	Nucleosides, nucleotides, and analogues	0.8287±0.0358	10.22±0.48	15.024	3.9092	0.15	1.712	Up
86.0603	.gamma.-aminobutyric acid	Organic acids and derivatives	753.1±36.7	241.6±16.0	0.39006	-1.3583	0.13	1.152	Down
381.9605	3,3'-diiodo-l-thyronine	Organic acids and derivatives	0.2255±0.0104	0.5628±0.0101	3.0382	1.6032	52.55	1.033	Up
348.0394	4-hydroxytriamterene sulfate	Organic acids and derivatives	#NUM!±0.00	3.044±0.304	89.326	6.481	0.04	4.485	Up
117.9968	Aminomalonic acid	Organic acids and derivatives	0.06721±0.00253	7.760±0.509	140.35	7.1329	0.08	2.354	Up
231.0977	Asp-Pro	Organic acids and	8.158±0.549	1.289±0.081	0.19266	-2.3759	0.25	1.467	Down

		derivatives								
424.2171	Calpain inhibitor ii	Organic acids and derivatives	0.1329±0.0038	0.8154±0.0307	7.4809	2.9032	0.81	1.455	Up
240.0656	Captopril	Organic acids and derivatives	0.01295±0.00072	0.1400±0.0009	13.15	3.717	1.82	1.667	Up
248.0930	Cys-Gln	Organic acids and derivatives	1.197±0.014	9.038±0.410	9.1985	3.2014	0.41	1.536	Up
441.2095	Cys-Tyr-Arg	Organic acids and derivatives	33.66±0.53	9.283±0.527	0.33686	-1.5698	68.36	1.226	Down
312.9849	Dicloxacillin	Organic acids and derivatives	5.134±0.174	1.736±0.092	0.41167	-1.2804	16.48	1.124	Down
130.0496	D-pyroglutamic acid	Organic acids and derivatives	4.048±0.102	11.80±0.37	3.5601	1.8319	11.77	1.117	Up
276.1191	Gamma-glu-glu	Organic acids and derivatives	37.01±2.10	124.0±12.1	4.078	2.0278	2.05	1.187	Up
295.1291	gamma-L-Glutamyl-L-phenylalanine	Organic acids and derivatives	3.132±0.130	16.22±0.46	6.3244	2.6609	1.89	1.385	Up
275.1352	Gln-gln	Organic acids and derivatives	14.97±0.47	4.944±0.212	0.40125	-1.3174	1.85	1.137	Down
304.1619	Glu-Arg	Organic acids and derivatives	1.076±0.047	13.39±1.55	15.159	3.9221	0.90	1.713	Up
407.1891	Glu-Met-Lys	Organic acids and derivatives	0.06948±0.00497	0.4282±0.0090	7.5131	2.9094	8.69	1.457	Up
613.1598	Glutathione, oxidized	Organic acids and derivatives	22.74±1.43	4.168±0.449	0.22265	-2.1671	2.81	1.408	Down
334.1401	Glu-Trp	Organic acids and derivatives	0.2474±0.0251	4.103±0.190	20.128	4.3312	2.45	1.811	Up
459.2199	Hc toxin	Organic acids and	9.466±0.938	1.886±0.178	0.24283	-2.042	145.82	1.369	Down

		derivatives							
229.1546	Ile-Pro	Organic acids and derivatives	51.41±2.83	17.74±0.67	0.42027	-1.2506	0.76	1.114	Down
318.1815	Ile-Trp	Organic acids and derivatives	2.945±0.165	10.03±1.05	4.1437	2.0509	7.82	1.194	Up
212.0058	Indoxyl sulfate	Organic acids and derivatives	#NUM!±0.00	0.5355±0.0402	8.5	3.0875	9.40	4.252	Up
154.0510	L-carnosine	Organic acids and derivatives	11.36±0.34	4.532±0.389	0.48657	-1.0393	7.23	1.036	Down
427.0956	L-cysteine-glutathione disulfide	Organic acids and derivatives	1.321±0.053	0.2551±0.0228	0.23462	-2.0916	9.60	1.385	Down
239.0196	L-cystine	Organic acids and derivatives	13.96±0.49	4.052±0.294	0.3539	-1.4986	2.51	1.202	Down
409.3103	Leupeptin	Organic acids and derivatives	13.60±0.42	35.09±1.72	3.1445	1.6528	1.11	1.050	Up
132.0125	L-homocystine	Organic acids and derivatives	0.4513±0.0116	53.15±3.23	143.57	7.1656	0.12	2.359	Up
128.0353	L-pyroglutamic acid	Organic acids and derivatives	503.0±9.0	1229±36	2.9709	1.5709	5.04	1.020	Up
469.2395	Met-Tyr-Arg	Organic acids and derivatives	0.08579±0.00306	0.2873±0.0217	4.0722	2.0258	10.09	1.186	Up
154.0976	N-acetylhistamine	Organic acids and derivatives	18.18±1.11	62.71±6.76	4.1922	2.0677	2.12	1.201	Up
226.0936	N-acetyl-p-fluoro-dl-phenylalanine	Organic acids and derivatives	187.5±15.9	76.29±3.54	0.49567	-1.0125	42.92	1.023	Down
316.1880	Nateglinide	Organic acids and derivatives	7.665±0.174	19.64±0.70	3.1249	1.6438	1.99	1.047	Up
290.0883	N-fructosyl pyroglutamate	Organic acids and	1.841±0.007	27.16±1.93	17.922	4.1637	0.03	1.772	Up

		derivatives							
210.1338	N-octanoyl-l-homoserine lactone	Organic acids and derivatives	5.907±0.584	2.239±0.217	0.46178	-1.1147	120.23	1.064	Down
118.0863	Norvaline	Organic acids and derivatives	80.96±8.27	4.684±0.408	0.070464	-3.827	0.63	1.824	Down
277.1228	Pantetheine	Organic acids and derivatives	120.2±8.3	41.98±2.00	0.42499	-1.2345	5.50	1.107	Down
577.2337	Pantethine	Organic acids and derivatives	0.3166±0.0087	0.7660±0.0780	2.9418	1.5567	12.42	1.013	Up
278.1148	Phe-asn	Organic acids and derivatives	0.6026±0.0043	1.646±0.092	3.3243	1.733	0.68	1.082	Up
192.0667	Phenaceturic acid	Organic acids and derivatives	1.903±0.072	8.547±0.364	5.4753	2.4529	0.42	1.324	Up
381.2111	Phe-Ser-Lys	Organic acids and derivatives	0.4416±0.0303	2.074±0.024	5.7362	2.5201	1.85	1.344	Up
267.1341	Phe-thr	Organic acids and derivatives	1.234±0.114	2.945±0.099	2.9097	1.5408	21.78	1.007	Up
352.1657	Phe-trp	Organic acids and derivatives	0.3841±0.0288	1.832±0.056	5.8197	2.541	2.75	1.350	Up
359.1829	Pyroglu-thr-lys	Organic acids and derivatives	0.1030±0.0050	0.3375±0.0333	3.9864	1.9951	24.08	1.173	Up
330.2037	Thr-Val-Leu	Organic acids and derivatives	4.144±0.182	10.12±0.18	2.9808	1.5757	10.30	1.020	Up
421.2426	Tris(2-butoxyethyl) phosphate	Organic acids and derivatives	#NUM!±0.00	1.249±0.063	42.021	5.3931	0.01	4.367	Up
302.1502	Trp-Pro	Organic acids and derivatives	15.80±1.59	3.886±0.234	0.29989	-1.7375	9.04	1.278	Down
329.1499	Tyr-Phe	Organic acids and	0.4980±0.0095	1.555±0.023	3.8005	1.9262	7.87	1.153	Up

		derivatives							
231.0987	Val-Asp	Organic acids and derivatives	0.7354±0.0368	1.740±0.059	2.8861	1.5291	16.70	1.001	Up
88.0758	2-amino-2-methyl-1,3-propanediol	Organic nitrogen compounds	4.453±0.157	54.74±3.26	14.977	3.9046	0.00	1.711	Up
172.0492	Crimidine	Organic nitrogen compounds	106.5±6.3	32.34±1.54	0.37039	-1.4329	54.12	1.178	Down
253.1798	Dipyridamole	Organic nitrogen compounds	7.079±0.385	2.470±0.114	0.42678	-1.2284	93.04	1.107	Down
262.1288	Methapyrilene	Organic nitrogen compounds	38.11±1.16	8.548±0.703	0.27317	-1.8721	3.33	1.321	Down
184.0733	Miltefosine	Organic nitrogen compounds	143.1±1.7	30.31±2.77	0.25725	-1.9588	0.75	1.346	Down
206.1653	N1-(1-methyl-4-piperidinyl)-1,4-benzene diamine	Organic nitrogen compounds	#NUM!±0.00	7.919±0.466	2739	11.419	0.00	4.608	Up
516.3030	Oleyloxyethylphosphorylcholine	Organic nitrogen compounds	#NUM!±0.00	0.4075±0.0356	34.052	5.0896	0.10	4.215	Up
318.3004	Phytosphingosine	Organic nitrogen compounds	11.90±0.35	29.41±1.52	3.0123	1.5908	12.15	1.025	Up
290.2076	1-heptanone, 1-(4-methoxyphenyl)-2-(1-pyrrolidinyl)-	Organic oxygen compounds	1.449±0.049	4.559±0.364	3.831	1.9377	4.46	1.154	Up
99.0918	2-hexenal	Organic oxygen compounds	2.544±0.033	55.76±3.45	26.648	4.7359	0.01	1.898	Up
151.0519	3,4-dihydroxyacetophenone	Organic oxygen compounds	#NUM!±0.00	130.4±6.0	388.81	8.6029	0.00	4.950	Up
535.1518	3-deoxy-d-glycero-d-galacto-2-nonuloso nic acid	Organic oxygen compounds	6.737±0.596	2.578±0.248	0.46567	-1.1026	83.09	1.059	Down
180.0688	D-mannosamine	Organic oxygen	0.4765±0.0280	33.46±0.82	85.555	6.4188	0.01	2.228	Up

		compounds							
259.0130	D-mannose 6-phosphate	Organic oxygen compounds	65.47±2.22	19.31±1.19	0.36044	-1.4722	76.36	1.194	Down
229.0354	D-ribose 1-phosphate	Organic oxygen compounds	27.59±2.41	8.094±0.111	0.35729	-1.4848	77.39	1.193	Down
339.0042	Fructose 1,6-diphosphate	Organic oxygen compounds	0.3248±0.0053	1.085±0.082	4.0535	2.0192	3.79	1.185	Up
447.1588	N,n'-diacetylchitobiose	Organic oxygen compounds	0.9065±0.0434	13.73±0.62	18.455	4.2059	0.04	1.781	Up
465.1696	N-acetylglucosamine	Organic oxygen compounds	0.4625±0.0143	2.070±0.069	5.4596	2.4488	3.83	1.322	Up
251.0776	Orcinol .beta.-d-glucoside	Organic oxygen compounds	24.30±2.37	6.889±0.423	0.34532	-1.534	29.02	1.211	Down
499.1645	Primeverin	Organic oxygen compounds	0.1147±0.0064	0.4877±0.0111	5.1991	2.3783	2.67	1.300	Up
318.1164	Prunasin	Organic oxygen compounds	0.07341±0.00245	4.373±0.476	72.322	6.1764	0.15	2.183	Up
195.1227	Tetraethylene glycol	Organic oxygen compounds	65.47±5.85	16.85±1.40	0.31458	-1.6685	113.36	1.256	Down
189.1236	.alpha.-ethyltryptamine	Organoheterocyclic compounds	5.798±0.238	58.62±3.51	12.344	3.6257	0.82	1.642	Up
204.0689	1-(2,8-dihydroxyquinolin-5-yl)ethan-1-one	Organoheterocyclic compounds	29.36±0.75	9.150±0.311	0.38019	-1.3952	5.70	1.167	Down
101.1074	1-methylpiperazine	Organoheterocyclic compounds	44.71±3.82	16.89±1.08	0.45947	-1.1219	16.35	1.065	Down
363.0928	1-methyluric acid	Organoheterocyclic compounds	0.2318±0.0155	0.8486±0.0744	4.4504	2.1539	7.22	1.228	Up
372.1897	1-pentyl-3-(4-methoxynaphthoyl)indole	Organoheterocyclic	0.1980±0.0081	0.6116±0.0702	3.7481	1.9062	19.26	1.143	Up

		compounds							
217.1046	2-(2',3',4'-trihydroxybutyl)quinoxaline	Organoheterocyclic compounds	29.76±0.62	3.448±0.263	0.1416	-2.8201	9.17	1.587	Down
557.2573	4-hydroxyatorvastatin lactone	Organoheterocyclic compounds	31.03±1.89	10.27±0.83	0.40555	-1.302	355.31	1.134	Down
144.0302	4-hydroxyquinoline	Organoheterocyclic compounds	35.56±1.21	13.18±1.00	0.45004	-1.1519	0.68	1.077	Down
208.0619	4-morpholinopropanesulfonic acid	Organoheterocyclic compounds	0.3112±0.0026	2.486±0.294	9.7088	3.2793	1.53	1.555	Up
129.1024	4-piperidinecarboxamide	Organoheterocyclic compounds	1.070±0.015	6.399±0.202	7.2934	2.8666	2.77	1.444	Up
232.0793	6-hydroxymelatonin	Organoheterocyclic compounds	15.79±0.61	5.893±0.178	0.45508	-1.1358	118.68	1.072	Down
213.0172	8-chlorotheophylline	Organoheterocyclic compounds	33.76±0.71	3.844±0.098	0.13883	-2.8487	0.94	1.593	Down
266.1114	Albendazole	Organoheterocyclic compounds	0.07488±0.00235	0.3520±0.0428	5.7084	2.5131	10.91	1.341	Up
298.1013	Albendazole sulfone	Organoheterocyclic compounds	1.179±0.043	3.689±0.391	3.808	1.929	5.23	1.152	Up
228.1343	Ametryne	Organoheterocyclic compounds	7.977±0.209	2.674±0.291	0.40741	-1.2954	21.31	1.131	Down
129.0408	Ammelide	Organoheterocyclic compounds	#NUM!±0.00	2.909±0.087	15.679	3.9708	0.04	4.479	Up
219.0916	Benzamide, n-1h-indol-5-yl-	Organoheterocyclic compounds	1.714±0.119	10.23±0.32	7.2771	2.8634	0.68	1.444	Up
326.1346	Bromosporine	Organoheterocyclic compounds	0.3118±0.0020	0.7590±0.0772	2.9742	1.5725	288.53	1.015	Up
195.0877	Caffeine	Organoheterocyclic	28.88±1.48	8.013±0.727	0.33762	-1.5665	9.64	1.224	Down

		compounds								
214.0687	Carbendazim	Organoheterocyclic compounds	6.551±0.144	2.282±0.270	0.42341	-1.2399	63.99	1.109	Down
217.0973	Carboline base + 4h, carboxylic acid	Organoheterocyclic compounds	0.5937±0.0057	7.274±0.445	14.915	3.8986	0.36	1.710	Up
233.0304	Cephalexin	Organoheterocyclic compounds	20.04±0.83	7.761±0.501	0.47255	-1.0815	635.51	1.052	Down
356.1559	Difloxacin	Organoheterocyclic compounds	0.7872±0.0790	0.2090±0.0080	0.32393	-1.6262	92.92	1.240	Down
382.1432	Enrofloxacin	Organoheterocyclic compounds	0.8103±0.0288	2.655±0.324	3.9934	1.9976	66.15	1.170	Up
399.0093	Ethiprole	Organoheterocyclic compounds	2.367±0.072	0.2962±0.0241	0.15241	-2.714	0.60	1.558	Down
236.0587	Ethyl 8-fluoro-4-hydroxyquinoline-3-carboxylate	Organoheterocyclic compounds	0.1845±0.0032	7.343±0.175	48.431	5.5979	0.04	2.074	Up
312.1538	Imazaquin	Organoheterocyclic compounds	0.9720±0.0260	2.415±0.035	3.0258	1.5973	3.65	1.031	Up
254.1501	Irgarol	Organoheterocyclic compounds	3.333±0.250	16.74±1.30	6.0974	2.6082	6.75	1.372	Up
427.2302	Lovatatin	Organoheterocyclic compounds	264.0±5.4	32.73±2.35	0.15112	-2.7263	7.20	1.561	Down
350.2691	Methanone, (1-pentyl-1h-indol-3-yl)tricyclo[3.3.1.13,7]dec-1-yl-	Organoheterocyclic compounds	0.4134±0.0140	1.344±0.108	3.9752	1.991	60.68	1.171	Up
388.1847	Methanone, [1-(5-hydroxypentyl)-1h-indol-3-yl](4-methoxy-1-naphthalenyl)-	Organoheterocyclic compounds	0.7107±0.0560	1.738±0.079	2.985	1.5777	7.53	1.021	Up

452.2485	NCGC00381123-01	Organoheterocyclic compounds	0.4094±0.0303	1.825±0.089	5.426	2.4399	26.24	1.321	Up
326.1236	N-desmethyldanofloxacin	Organoheterocyclic compounds	2.233±0.148	0.7293±0.0307	0.39831	-1.328	6.23	1.142	Down
153.0660	Nudifloramide	Organoheterocyclic compounds	0.3175±0.0224	1.298±0.079	4.9705	2.3134	1.06	1.282	Up
181.0720	Paraxanthine	Organoheterocyclic compounds	39.01±2.55	9.846±0.969	0.3069	-1.7041	11.77	1.268	Down
168.0656	Pyridoxal	Organoheterocyclic compounds	30.00±0.74	10.34±1.00	0.42044	-1.25	589.71	1.115	Down
144.0667	Quinolin-2-ol	Organoheterocyclic compounds	#NUM!±0.00	7.550±0.713	50.184	5.6492	0.02	4.601	Up
131.0454	Quinoxaline	Organoheterocyclic compounds	8.722±0.827	3.283±0.285	0.45884	-1.1239	70.21	1.067	Down
288.1056	Rutaecarpine	Organoheterocyclic compounds	1.853±0.025	93.34±4.87	61.286	5.9375	0.01	2.139	Up
181.0530	Theophylline	Organoheterocyclic compounds	51.26±2.09	9.403±0.660	0.22343	-2.1621	0.96	1.407	Down
111.0200	Uracil	Organoheterocyclic compounds	185.4±16.1	42.08±2.13	0.27639	-1.8552	3.80	1.315	Down
235.1190	Zolpidem	Organoheterocyclic compounds	7.011±0.342	22.84±2.60	3.9601	1.9855	3.86	1.172	Up
415.2562	Propanoic acid, 2-[[4-[2-[[(cyclohexylamino)carbonyl](4-cyclohexylbutyl)amino]ethyl]phenyl]thio]-2-methyl-	Organosulfur compounds	0.7379±0.0110	2.199±0.133	3.6325	1.8609	43.60	1.128	Up
485.2356	(2r,3r,4s,5s,6r)-2-[1,7-bis(4-hydroxyphenyl)heptan-3-yloxy]-6-(hydroxymethyl)ox	Phenylpropanoids and polyketides	19.66±0.50	2.495±0.295	0.15559	-2.6841	38.27	1.553	Down

	ane-3,4,5-triol									
377.0757	3',4',5,7-tetrahydroxy-3,6,8-trimethoxyflavone	Phenylpropanoids and polyketides	0.6035±0.0505	1.729±0.155	3.4794	1.7988	58.86	1.104	Up
313.0911	3,7,3'-trimethoxyflavone	Phenylpropanoids and polyketides	5.687±0.199	2.215±0.070	0.47508	-1.0738	0.92	1.049	Down
221.0956	Benzyl cinnamate	Phenylpropanoids and polyketides	0.1980±0.0077	10.57±0.91	64.99	6.0222	0.21	2.154	Up
358.0909	Casticin	Phenylpropanoids and polyketides	0.08054±0.00498	0.2518±0.0237	3.8269	1.9362	122.06	1.149	Up
530.2593	Epothilone b	Phenylpropanoids and polyketides	#NUM!±0.00	1.282±0.045	12.914	3.6909	0.06	4.371	Up
449.0936	Marein	Phenylpropanoids and polyketides	0.2838±0.0091	2.652±0.198	11.377	3.508	0.17	1.615	Up
395.1663	Rotenone	Phenylpropanoids and polyketides	2.952±0.128	26.64±2.53	10.98	3.4568	0.12	1.602	Up
681.1296	Rutarensin	Phenylpropanoids and polyketides	#NUM!±0.00	0.4287±0.0159	33.339	5.0592	0.04	4.222	Up
463.1327	Tectoridin	Phenylpropanoids and polyketides	0.8737±0.0223	9.306±0.986	12.956	3.6956	0.86	1.660	Up
