# Supplementary material for: Untargeted Metabolomic Analysis Combined with Chemometrics Revealed the Effects of Different Cooking Methods on Lentinus edodes
Source: Molecules. 2023 Aug 11;28(16):6009. doi: 10.3390/molecules28166009 (PMC10458448; doi:10.3390/molecules28166009)
Supplement: Supplementary file 1 [file molecules-28-06009-s001.zip › Table S6.pdf]

Table S6 List of differential metabolites between Roasting and Control

m/z	Name	SuperClass	Area (10^7) Control	Area (10^7) Roasting	Fold Change (FC)	log2(FC)	P value (10^-6)	VIP	Type
324.1899	Lsd	Alkaloids and derivatives	4.147±0.282	14.02±1.16	4.00	2.00	14.18	1.057	Up
340.1847	Methylergonovine	Alkaloids and derivatives	0.5030±0.0255	1.595±0.056	3.75	1.91	35.49	1.060	Up
218.1387	Pentazocine	Alkaloids and derivatives	191.9±10.5	412.8±22.3	2.55	1.35	0.81	1.056	Up
421.1222	(5-benzoyloxy-4,6-dihydroxy-3-methoxy cyclohexen-1-yl)methyl benzoate	Benzenoids	2.321±0.109	16.87±1.09	8.60	3.11	0.04	1.060	Up
145.0609	.beta.-naphthol	Benzenoids	11.49±0.40	2.733±0.228	0.28	-1.83	6.58	1.059	Down
253.1799	1-benzhydrylpiperazine	Benzenoids	17.46±1.59	5.878±0.711	0.40	-1.33	276.45	1.052	Down
178.0719	2,2-bis(4-chlorophenyl)ethanol	Benzenoids	31.19±2.23	2.798±0.166	0.11	-3.23	0.37	1.061	Down
188.9355	2,4-dichlorobenzoic acid	Benzenoids	14.22±0.48	66.56±1.92	5.56	2.47	2.26	1.060	Up
110.0601	2-aminophenol	Benzenoids	3.251±0.268	8.579±0.180	3.13	1.65	9.77	1.059	Up
208.0968	2-chloro-2',6'-diethylacetanilide	Benzenoids	0.06858±0.00637	0.4967±0.0367	8.58	3.10	0.92	1.059	Up
168.0306	2-methoxy-5-nitrophenol	Benzenoids	2.312±0.039	0.3923±0.0068	0.20	-2.32	2.35	1.061	Down
227.1754	3,3'-dimethyl-4,4'-diaminodiphenylmetha ne	Benzenoids	1.819±0.024	0.5438±0.0251	0.35	-1.49	15.89	1.060	Down
227.0198	3,5-dinitrosalicylate	Benzenoids	11.13±0.74	3.532±0.333	0.38	-1.41	43.45	1.057	Down
256.0827	3-hydroxymethylmefenamic acid	Benzenoids	641.1±41.8	204.3±15.6	0.38	-1.40	7.12	1.058	Down
105.0699	4-methylbenzyl alcohol	Benzenoids	72.25±3.97	17.35±1.05	0.28	-1.82	0.09	1.060	Down
289.0236	5-benzoyl-4-hydroxy-2-methoxybenzene sulfonic acid	Benzenoids	9.607±0.338	2.855±0.084	0.35	-1.51	0.08	1.061	Down
266.1250	Anisomycin	Benzenoids	38.84±1.62	13.08±1.09	0.40	-1.33	57.64	1.057	Down
404.1335	Azoxystrobin	Benzenoids	0.2330±0.0074	0.9224±0.0666	4.68	2.23	22.27	1.059	Up
377.0693	Benzenepropanamide, n-(6-chloro-2-benzothiazolyl)-3,4-dimeth	Benzenoids	18.02±1.15	2.424±0.193	0.16	-2.65	4.86	1.060	Down

	oxy-								
354.9838	Benzenesulfonic acid, 2-[(5-bromo-2-hydroxyphenyl)methylene]hydrazide	Benzenoids	0.3494±0.0184	3.566±0.190	12.09	3.60	4.90	1.061	Up
204.1339	Crotamiton	Benzenoids	425.6±11.7	795.2±43.9	2.21	1.14	0.74	1.055	Up
397.1157	Daunomycinone	Benzenoids	8.189±0.147	1.731±0.068	0.25	-2.00	2.96	1.061	Down
441.3075	Di(2-nonyl) phthalate	Benzenoids	3.346±0.199	0.9549±0.0491	0.34	-1.56	40.89	1.058	Down
317.1112	Dibutyl phthalate	Benzenoids	0.1847±0.0059	2.105±0.012	13.51	3.76	0.74	1.061	Up
386.1065	Flamprop-isopropyl	Benzenoids	0.1580±0.0035	0.3676±0.0168	2.76	1.46	22.86	1.059	Up
152.0562	Flufenacet	Benzenoids	5.139±0.223	46.59±0.93	10.75	3.43	0.34	1.061	Up
297.0882	Flunixin	Benzenoids	7.740±0.172	2.694±0.214	0.41	-1.28	24.83	1.058	Down
316.1006	Flusilazole	Benzenoids	ND	11.05±1.20	492.24	8.94	0.09	1.061	Up
376.0642	Haloxyfop-methyl	Benzenoids	3.883±0.102	1.108±0.047	0.34	-1.56	24.40	1.060	Down
494.2590	Imatinib	Benzenoids	ND	2.018±0.151	39.94	5.32	0.27	1.061	Up
412.1104	Mandipropamid	Benzenoids	7.696±0.137	2.721±0.101	0.42	-1.26	4.38	1.060	Down
343.1615	Methanone, 1-naphthalenyl(1-pentyl-1h-indazol-3-yl)-	Benzenoids	0.1111±0.0061	0.5887±0.0219	6.29	2.65	5.06	1.061	Up
318.1559	M-hydroxycocaine	Benzenoids	0.1165±0.0034	0.2679±0.0115	2.73	1.45	49.14	1.059	Up
215.0562	Nepodin	Benzenoids	25.51±0.82	10.37±0.30	0.48	-1.06	118.75	1.060	Down
184.0616	N-hydroxy-4-aminobiphenyl	Benzenoids	10.01±0.82	22.85±1.97	2.71	1.44	24.84	1.052	Up
387.1046	Pamoic acid	Benzenoids	3.251±0.152	6.544±0.303	2.38	1.25	11.84	1.057	Up
122.0964	Phenylethylamine	Benzenoids	30.06±1.98	6.727±0.243	0.27	-1.91	1.84	1.060	Down
297.1172	Rac-didemethylcitalopram	Benzenoids	3.552±0.144	1.366±0.092	0.46	-1.13	134.25	1.057	Down
475.2152	Sildenafil	Benzenoids	4.171±0.310	1.732±0.048	0.49	-1.02	66.71	1.057	Down
313.0299	Spirodiclofen	Benzenoids	1.531±0.093	3.785±0.137	2.93	1.55	2.65	1.058	Up
387.1992	Sufentanyl	Benzenoids	4.780±0.105	1.883±0.079	0.47	-1.10	60.31	1.060	Down
405.1483	Sulfinpyrazone	Benzenoids	1.019±0.043	8.431±0.063	9.81	3.29	1.27	1.061	Up

223.1443	Zectran	Benzenoids	0.04378±0.00111	0.9761±0.0407	26.45	4.73	0.41	1.061	Up
488.2518	(2e,6e,11e,13e)-18-(2,6-dioxopiperidin-4-yl)-9-hydroxy-8-methoxy-10,12,14-trimethyl-15-oxooctadeca-2,6,11,13-tetraenoic acid	Lipids and lipid-like molecules	ND	0.3405±0.0166	18.85	4.24	0.51	1.061	Up
311.2229	(9z,12e)-15,16-dihydroxyoctadeca-9,12-dienoic acid	Lipids and lipid-like molecules	117.8±2.1	16.03±1.13	0.16	-2.63	0.13	1.061	Down
362.3265	(r)-(+)-arachidonyl-1'-hydroxy-2'-propylamide	Lipids and lipid-like molecules	3.916±0.082	6.902±0.214	2.09	1.06	7.76	1.059	Up
173.0810	(r)-butyrylcarnitine	Lipids and lipid-like molecules	4.870±0.311	8.294±0.089	2.02	1.01	26.00	1.055	Up
476.2782	1-(9z,12z-octadecadienoyl)-2-hydroxy-sn-glycero-3-phosphoethanolamine	Lipids and lipid-like molecules	308.4±14.1	101.0±6.2	0.39	-1.37	142.80	1.058	Down
401.2165	1,2-dihydrodesoxymetasone	Lipids and lipid-like molecules	7.729±0.370	2.478±0.165	0.38	-1.40	73.77	1.058	Down
688.4907	1,2-dipalmitoleoyl-sn-glycero-3-phosphoethanolamine	Lipids and lipid-like molecules	2.882±0.040	11.74±0.14	4.84	2.27	0.88	1.061	Up
295.2280	12(13)-epoxy-9z-octadecenoic acid	Lipids and lipid-like molecules	41.28±1.91	8.876±0.360	0.25	-1.97	3.05	1.060	Down
331.2342	17alpha-hydroxyprogesterone	Lipids and lipid-like molecules	0.5421±0.0153	1.696±0.023	3.71	1.89	10.77	1.061	Up
424.3061	17-phenyltrinorprostaglandin f2.alpha. cyclopropyl methyl amide	Lipids and lipid-like molecules	5.225±0.202	36.14±3.35	8.20	3.04	4.02	1.059	Up
366.2367	17-phenyltrinorprostaglandin f2a methylamide	Lipids and lipid-like molecules	2.316±0.037	6.690±0.576	3.42	1.77	23.10	1.056	Up
608.4658	1-lignoceroyl-2-hydroxy-sn-glycero-3-phosphocholine	Lipids and lipid-like molecules	0.09495±0.00928	3.015±0.072	37.70	5.24	0.38	1.061	Up
468.3087	1-myristoyl-sn-glycero-3-phosphocholin	Lipids and lipid-like	3.324±0.320	8.798±0.168	3.14	1.65	16.98	1.058	Up

	e	molecules							
784.5126	1-palmitoyl-2-arachidonoyl-sn-glycero-3-phosphoserine	Lipids and lipid-like molecules	17.28±1.20	41.67±0.87	2.85	1.51	71.66	1.057	Up
740.5224	1-palmitoyl-2-oleoyl-sn-glycero-3-phosphoethanolamine	Lipids and lipid-like molecules	152.4±2.8	478.8±25.8	3.72	1.89	0.90	1.059	Up
496.3400	1-palmitoyl-sn-glycero-3-phosphocholine	Lipids and lipid-like molecules	64.89±4.25	128.2±3.3	2.34	1.23	21.98	1.057	Up
482.3243	1-pentadecanoyl-sn-glycero-3-phosphocholine	Lipids and lipid-like molecules	11.57±0.37	23.91±1.58	2.45	1.29	6.13	1.055	Up
546.3544	1-stearoyl-2-hydroxy-sn-glycero-3-phosphocholine	Lipids and lipid-like molecules	0.5798±0.0442	2.783±0.301	5.69	2.51	18.64	1.057	Up
594.3770	2-(5-oxovaleryl)phosphatidylcholine	Lipids and lipid-like molecules	ND	0.02421±0.00081	6.15	2.62	1.55	1.061	Up
145.0506	2,2-Dimethylsuccinic acid	Lipids and lipid-like molecules	51.02±2.40	13.86±1.23	0.32	-1.64	14.93	1.058	Down
349.1835	2,3-dinor-8-isoprostaglandin-f2.alpha.	Lipids and lipid-like molecules	8.059±0.380	3.255±0.100	0.48	-1.07	55.71	1.059	Down
447.1621	21-carboxylic acid triamcinolone acetonide	Lipids and lipid-like molecules	0.6828±0.0468	0.2072±0.0171	0.36	-1.48	288.67	1.057	Down
175.0612	2-Isopropylmalic acid	Lipids and lipid-like molecules	130.5±8.3	19.46±1.23	0.18	-2.51	7.50	1.060	Down
716.5225	2-linoleoyl-1-palmitoyl-sn-glycero-3-phosphoethanolamine	Lipids and lipid-like molecules	24.45±0.72	93.15±2.08	4.51	2.17	1.85	1.060	Up
577.2837	3-deacetylsalannin	Lipids and lipid-like molecules	1.647±0.066	0.6877±0.0797	0.50	-1.01	801.59	1.048	Down
543.2779	3-hydroxystanozolol glucuronide	Lipids and lipid-like molecules	4.153±0.075	0.7814±0.0551	0.22	-2.16	11.91	1.060	Down
375.2244	5(s),14(r)-lipoxin b4	Lipids and lipid-like	0.3783±0.0330	0.8095±0.0945	2.53	1.34	834.66	1.036	Up

		molecules								
241.2038	5alpha-pregnan-3,20-dione	Lipids and lipid-like molecules	0.3181±0.0041	1.170±0.062	4.36	2.13	9.25	1.060	Up
251.1797	5-androsten-3.beta.,16.alpha.-diol-17-one	Lipids and lipid-like molecules	2.036±0.146	4.591±0.165	2.67	1.42	82.99	1.055	Up
431.1902	6.beta.-hydroxyeplerenone	Lipids and lipid-like molecules	0.1452±0.0077	0.6003±0.0186	4.90	2.29	19.99	1.061	Up
297.2403	7,8-dehydropregnenolone	Lipids and lipid-like molecules	0.3977±0.0071	0.1323±0.0147	0.39	-1.34	188.09	1.054	Down
293.2123	9-oxo-10(e),12(e)-octadecadienoic acid	Lipids and lipid-like molecules	29.08±1.64	8.770±0.757	0.36	-1.49	41.62	1.057	Down
810.1338	Acetyl-coa	Lipids and lipid-like molecules	1.311±0.039	0.2860±0.0042	0.26	-1.95	4.19	1.061	Down
327.1781	Acitretin	Lipids and lipid-like molecules	17.95±0.61	6.891±0.644	0.46	-1.13	125.70	1.055	Down
303.2001	Aleuritic acid	Lipids and lipid-like molecules	10.88±0.36	18.56±1.52	2.01	1.01	72.06	1.041	Up
427.2560	Andrastin d	Lipids and lipid-like molecules	1.867±0.126	4.402±0.362	2.80	1.49	60.64	1.051	Up
255.1705	Beta-estradiol	Lipids and lipid-like molecules	0.3517±0.0240	0.8631±0.0279	2.91	1.54	94.71	1.057	Up
411.2217	Betamethasone 9,11-epoxide 21-propionate	Lipids and lipid-like molecules	0.09691±0.00278	1.402±0.047	17.14	4.10	0.45	1.061	Up
628.3626	Bulleyaconi cine a	Lipids and lipid-like molecules	1.589±0.022	0.3460±0.0189	0.26	-1.95	13.07	1.060	Down
405.1695	Chlormadinone acetate	Lipids and lipid-like molecules	4.894±0.230	0.7977±0.0289	0.19	-2.37	3.27	1.060	Down
465.3043	Cholesteryl sulfate	Lipids and lipid-like	0.4531±0.0143	2.473±0.150	6.46	2.69	3.60	1.060	Up

		molecules							
407.2955	Cholic acid	Lipids and lipid-like molecules	2.081±0.074	12.37±0.97	7.05	2.82	5.51	1.059	Up
443.2251	Cinobufagin	Lipids and lipid-like molecules	398.0±5.5	163.8±1.5	0.49	-1.04	39.29	1.061	Down
147.0299	Citramalate	Lipids and lipid-like molecules	17.77±0.81	83.39±6.66	5.56	2.48	14.20	1.059	Up
395.2269	Deoxycorticosterone acetate	Lipids and lipid-like molecules	1.106±0.098	3.783±0.260	4.05	2.02	11.95	1.058	Up
293.2113	Desogestrel	Lipids and lipid-like molecules	1.419±0.014	0.4442±0.0151	0.37	-1.43	20.43	1.060	Down
206.1387	Dexpanthenol	Lipids and lipid-like molecules	3.317±0.229	0.5103±0.0145	0.18	-2.45	3.09	1.060	Down
423.1979	Fludrocortisone	Lipids and lipid-like molecules	0.4449±0.0044	5.716±0.076	15.21	3.93	0.07	1.061	Up
417.1921	Flunisolide	Lipids and lipid-like molecules	0.2050±0.0116	0.3840±0.0067	2.22	1.15	104.73	1.056	Up
427.1329	Gardenoside	Lipids and lipid-like molecules	0.3402±0.0176	2.642±0.104	9.17	3.20	1.88	1.060	Up
219.1744	Germacrone	Lipids and lipid-like molecules	9.224±0.502	3.360±0.106	0.43	-1.21	2.29	1.059	Down
309.1675	Gestrinone	Lipids and lipid-like molecules	25.46±2.58	7.125±0.840	0.33	-1.59	148.57	1.054	Down
283.1267	Gibberellic acid	Lipids and lipid-like molecules	3.917±0.106	1.067±0.094	0.32	-1.63	110.01	1.058	Down
315.1675	Gibberellin a9	Lipids and lipid-like molecules	0.8112±0.0429	3.079±0.089	4.49	2.17	8.93	1.060	Up
339.1103	Lactobionic acid	Lipids and lipid-like	2.389±0.069	4.358±0.053	2.16	1.11	4.05	1.060	Up

		molecules							
495.2601	Leukotriene d4	Lipids and lipid-like molecules	7.080±0.187	1.167±0.111	0.20	-2.36	17.14	1.059	Down
365.1056	Maltose	Lipids and lipid-like molecules	22.27±0.81	63.65±1.18	3.39	1.76	8.60	1.060	Up
406.1323	N-acetyl-d-lactosamine	Lipids and lipid-like molecules	1.737±0.026	3.394±0.198	2.32	1.21	15.72	1.055	Up
143.1077	Octanoic acid	Lipids and lipid-like molecules	6.343±0.254	2.065±0.182	0.39	-1.37	70.61	1.057	Down
423.2583	Ophiobolin a	Lipids and lipid-like molecules	0.1194±0.0064	3.273±0.270	32.43	5.02	0.63	1.061	Up
309.1295	Paclitaxel	Lipids and lipid-like molecules	2.999±0.285	11.96±0.83	4.72	2.24	50.62	1.056	Up
802.5600	Pc 33:2	Lipids and lipid-like molecules	1.968±0.157	3.757±0.171	2.25	1.17	208.13	1.048	Up
816.5757	Pc 34:2	Lipids and lipid-like molecules	6.343±0.204	13.85±0.92	2.58	1.37	9.64	1.054	Up
714.5078	Pe 34:2	Lipids and lipid-like molecules	23.99±1.61	122.9±1.8	6.08	2.60	6.29	1.060	Up
742.5357	Pe 36:2	Lipids and lipid-like molecules	2.688±0.094	7.926±0.664	3.49	1.80	32.94	1.056	Up
738.5080	Pe 36:4	Lipids and lipid-like molecules	179.7±9.2	755.9±45.7	4.97	2.31	4.04	1.058	Up
639.4085	Phorbol 12-myristate 13-acetate	Lipids and lipid-like molecules	4.310±0.409	1.232±0.036	0.34	-1.56	47.69	1.058	Down
833.5185	Pi 34:2	Lipids and lipid-like molecules	9.720±0.335	27.33±0.43	3.33	1.74	12.46	1.060	Up
553.2965	Proscillaridin a	Lipids and lipid-like	0.2954±0.0102	5.458±0.291	21.87	4.45	0.09	1.061	Up

		molecules							
359.2405	Prostaglandin f2.alpha. 1,15-lactone	Lipids and lipid-like molecules	1.228±0.118	3.948±0.171	3.81	1.93	11.62	1.058	Up
392.3312	Prostaglandin f2.alpha. diethylamide	Lipids and lipid-like molecules	7.903±0.704	39.69±2.16	5.96	2.58	5.63	1.060	Up
201.1133	Sebacic acid	Lipids and lipid-like molecules	4.789±0.092	19.21±1.27	4.74	2.25	0.77	1.059	Up
143.0817	Succinic acid n,n-dimethylhydrazide	Lipids and lipid-like molecules	0.1924±0.0130	2.153±0.015	13.28	3.73	0.53	1.061	Up
173.0922	Thymol	Lipids and lipid-like molecules	4.775±0.488	1.652±0.073	0.41	-1.29	97.63	1.056	Down
314.0640	2'-Deoxyadenosine 5'-monophosphate (dAMP)	Nucleosides, nucleotides, and analogues	ND	0.3858±0.0337	22.00	4.46	1.36	1.061	Up
560.0795	Adenosine 5'-diphosphoribose	Nucleosides, nucleotides, and analogues	0.1029±0.0001	1.431±0.045	16.50	4.04	0.49	1.061	Up
370.0527	Adenosine 5'-monophosphate	Nucleosides, nucleotides, and analogues	2.821±0.231	5.144±0.176	2.16	1.11	64.60	1.051	Up
346.0557	Adenosine 5'-phosphosulfate	Nucleosides, nucleotides, and analogues	28.60±0.89	82.39±0.81	3.41	1.77	4.88	1.060	Up
462.0669	Adenylosuccinate	Nucleosides, nucleotides, and analogues	0.8736±0.0803	0.1944±0.0072	0.26	-1.92	25.82	1.058	Down
464.0819	Adenylosuccinic acid	Nucleosides, nucleotides, and analogues	0.9471±0.0109	0.1795±0.0039	0.22	-2.15	3.92	1.061	Down
558.0644	Adp-ribose	Nucleosides, nucleotides, and analogues	0.2528±0.0033	2.261±0.071	10.59	3.41	0.70	1.061	Up
304.0341	Cytidine 2',3'-cyclic phosphate	Nucleosides, nucleotides, and analogues	0.2886±0.0021	1.275±0.079	5.23	2.39	11.59	1.060	Up
252.1092	Deoxyadenosine	Nucleosides, nucleotides,	13.14±0.76	30.82±0.87	2.78	1.48	21.40	1.059	Up

		and analogues							
588.0753	Gdp-l-fucose	Nucleosides, nucleotides, and analogues	0.4848±0.0165	1.529±0.054	3.74	1.90	4.48	1.060	Up
344.0401	Guanosine 3',5'-cyclic monophosphate	Nucleosides, nucleotides, and analogues	0.4630±0.0405	3.120±0.110	7.96	2.99	7.91	1.060	Up
809.0125	Uridine 5'-diphosphate (UDP)	Nucleosides, nucleotides, and analogues	11.87±0.42	4.828±0.061	0.48	-1.05	3.64	1.060	Down
290.0859	Zidovudine	Nucleosides, nucleotides, and analogues	0.8287±0.0358	14.55±0.87	20.74	4.37	0.03	1.061	Up
86.0603	.gamma.-aminobutyric acid	Organic acids and derivatives	753.1±36.7	255.0±15.6	0.40	-1.32	2.11	1.059	Down
381.9605	3,3'-diiodo-l-thyronine	Organic acids and derivatives	0.2255±0.0104	0.6984±0.0294	3.67	1.87	39.53	1.060	Up
348.0394	4-hydroxytriamterene sulfate	Organic acids and derivatives	ND	3.205±0.211	91.65	6.52	0.03	1.061	Up
237.1237	Ala-phe	Organic acids and derivatives	4.110±0.048	7.817±0.280	2.25	1.17	15.86	1.059	Up
276.1345	Ala-Trp	Organic acids and derivatives	4.362±0.100	7.458±0.385	2.03	1.02	27.81	1.054	Up
117.9968	Aminomalonic acid	Organic acids and derivatives	0.06721±0.00253	1.770±0.105	31.16	4.96	0.53	1.061	Up
286.1773	Arg-Leu	Organic acids and derivatives	4.177±0.326	13.31±0.26	3.78	1.92	10.70	1.059	Up
231.0977	Asp-Pro	Organic acids and derivatives	8.158±0.549	1.428±0.074	0.21	-2.26	8.53	1.060	Down
424.2171	Calpain inhibitor ii	Organic acids and derivatives	0.1329±0.0038	1.397±0.063	12.45	3.64	1.56	1.061	Up
240.0656	Captopril	Organic acids and	0.01295±0.00072	0.2288±0.0058	20.88	4.38	0.98	1.061	Up

		derivatives							
248.0930	Cys-Gln	Organic acids and derivatives	1.197±0.014	10.70±0.15	10.59	3.40	0.23	1.061	Up
441.2095	Cys-Tyr-Arg	Organic acids and derivatives	33.66±0.53	8.447±0.595	0.30	-1.75	11.45	1.060	Down
312.9849	Dicloxacillin	Organic acids and derivatives	5.134±0.174	1.852±0.068	0.43	-1.23	49.00	1.060	Down
130.0496	D-pyroglutamic acid	Organic acids and derivatives	4.048±0.102	11.39±0.48	3.33	1.74	6.17	1.060	Up
276.1191	Gamma-glu-glu	Organic acids and derivatives	37.01±2.10	129.6±1.0	4.16	2.06	1.22	1.060	Up
295.1291	gamma-L-Glutamyl-L-phenylalanine	Organic acids and derivatives	3.132±0.130	15.53±0.93	5.87	2.55	0.02	1.060	Up
275.1352	Gln-gln	Organic acids and derivatives	14.97±0.47	4.480±0.062	0.35	-1.50	3.82	1.061	Down
304.1619	Glu-Arg	Organic acids and derivatives	1.076±0.047	13.16±0.22	14.52	3.86	0.42	1.061	Up
407.1891	Glu-Met-Lys	Organic acids and derivatives	0.06948±0.00497	0.3455±0.0278	5.89	2.56	22.07	1.058	Up
613.1598	Glutathione, oxidized	Organic acids and derivatives	22.74±1.43	4.042±0.053	0.21	-2.25	0.49	1.061	Down
334.1401	Glu-Trp	Organic acids and derivatives	0.2474±0.0251	4.057±0.107	19.36	4.28	2.98	1.060	Up
459.2199	Hc toxin	Organic acids and derivatives	9.466±0.938	1.799±0.112	0.22	-2.15	59.53	1.059	Down
288.2032	Ile-Arg	Organic acids and derivatives	7.176±0.229	15.64±0.94	2.58	1.37	0.05	1.056	Up
318.1815	Ile-Trp	Organic acids and	2.945±0.165	12.47±0.17	5.03	2.33	0.67	1.061	Up

		derivatives							
212.0058	Indoxyl sulfate	Organic acids and derivatives	ND	0.4881±0.0398	7.50	2.91	1.31	1.061	Up
173.0092	Isocitrate	Organic acids and derivatives	43.81±3.11	18.24±1.12	0.49	-1.02	11.59	1.056	Down
177.1125	L-canavanine	Organic acids and derivatives	3.280±0.171	1.240±0.041	0.45	-1.16	60.19	1.059	Down
427.0956	L-cysteine-glutathione disulfide	Organic acids and derivatives	1.321±0.053	0.2409±0.0028	0.22	-2.21	9.19	1.061	Down
239.0196	L-cystine	Organic acids and derivatives	13.96±0.49	4.213±0.083	0.36	-1.48	4.02	1.060	Down
203.1391	Leu-Ala	Organic acids and derivatives	15.01±0.84	37.10±2.97	2.93	1.55	43.37	1.055	Up
279.1705	Leu-Phe	Organic acids and derivatives	16.90±0.72	37.77±1.30	2.65	1.41	11.75	1.059	Up
231.1705	Leu-Val	Organic acids and derivatives	44.83±3.07	140.6±3.7	3.73	1.90	4.01	1.060	Up
132.0125	L-homocystine	Organic acids and derivatives	0.4513±0.0116	6.707±0.187	17.60	4.14	0.26	1.061	Up
405.2141	Lincomycin	Organic acids and derivatives	3.709±0.294	9.564±0.446	3.05	1.61	10.54	1.057	Up
128.0353	L-pyroglutamic acid	Organic acids and derivatives	503.0±9.0	1141±63	2.68	1.42	2.21	1.057	Up
469.2395	Met-Tyr-Arg	Organic acids and derivatives	0.08579±0.00306	0.4565±0.0200	6.30	2.66	6.77	1.061	Up
102.0550	N-(.beta.-ketocaproyl)-dl-homoserine lactone	Organic acids and derivatives	5.447±0.417	2.037±0.044	0.44	-1.17	96.06	1.057	Down
154.0976	N-acetylhistamine	Organic acids and	18.18±1.11	45.40±0.53	2.96	1.57	8.02	1.059	Up

		derivatives							
316.1880	Nateglinide	Organic acids and derivatives	7.665±0.174	25.39±1.81	3.91	1.97	1.47	1.058	Up
290.0883	N-fructosyl pyroglutamate	Organic acids and derivatives	1.841±0.007	11.17±0.10	7.18	2.84	0.49	1.061	Up
210.1338	N-octanoyl-l-homoserine lactone	Organic acids and derivatives	5.907±0.584	2.096±0.090	0.42	-1.25	96.01	1.056	Down
118.0863	Norvaline	Organic acids and derivatives	80.96±8.27	3.726±0.302	0.05	-4.20	0.90	1.060	Down
277.1228	Pantetheine	Organic acids and derivatives	120.2±8.3	24.23±1.84	0.24	-2.07	2.07	1.059	Down
577.2337	Pantethine	Organic acids and derivatives	0.3166±0.0087	0.9799±0.0320	3.67	1.88	7.67	1.061	Up
278.1148	Phe-asn	Organic acids and derivatives	0.6026±0.0043	2.148±0.068	4.22	2.08	3.43	1.061	Up
297.1270	Phe-met	Organic acids and derivatives	0.8858±0.0198	2.342±0.153	3.13	1.65	23.24	1.058	Up
192.0667	Phenaceturic acid	Organic acids and derivatives	1.903±0.072	3.768±0.119	2.34	1.23	0.89	1.059	Up
381.2111	Phe-Ser-Lys	Organic acids and derivatives	0.4416±0.0303	3.110±0.212	8.33	3.06	1.19	1.060	Up
267.1341	Phe-thr	Organic acids and derivatives	1.234±0.114	3.370±0.197	3.24	1.70	23.78	1.056	Up
352.1657	Phe-trp	Organic acids and derivatives	0.3841±0.0288	2.448±0.138	7.56	2.92	1.91	1.060	Up
213.1235	Pro-pro	Organic acids and derivatives	14.14±0.78	24.00±1.46	2.01	1.01	1.27	1.049	Up
359.1829	Pyroglu-thr-lys	Organic acids and	0.1030±0.0050	0.4003±0.0055	4.62	2.21	12.19	1.061	Up

		derivatives							
251.1038	Ser-Phe	Organic acids and derivatives	9.256±0.176	24.76±1.90	3.16	1.66	1.79	1.057	Up
231.1351	Thr-Leu	Organic acids and derivatives	30.01±1.67	66.39±2.99	2.63	1.39	19.34	1.057	Up
330.2037	Thr-Val-Leu	Organic acids and derivatives	4.144±0.182	11.46±0.53	3.28	1.71	7.21	1.059	Up
421.2426	Tris(2-butoxyethyl) phosphate	Organic acids and derivatives	ND	1.522±0.098	49.76	5.64	0.22	1.061	Up
302.1502	Trp-Pro	Organic acids and derivatives	15.80±1.59	4.830±0.123	0.36	-1.46	26.37	1.058	Down
295.1655	Tyr-Ile	Organic acids and derivatives	6.300±0.338	16.17±1.20	3.04	1.60	1.85	1.056	Up
329.1499	Tyr-Phe	Organic acids and derivatives	0.4980±0.0095	1.929±0.100	4.58	2.20	10.56	1.060	Up
231.0987	Val-Asp	Organic acids and derivatives	0.7354±0.0368	2.268±0.149	3.65	1.87	15.49	1.058	Up
249.1269	Val-met	Organic acids and derivatives	3.384±0.281	8.469±0.660	2.97	1.57	46.80	1.054	Up
217.1548	Val-Val	Organic acids and derivatives	4.749±0.293	15.63±1.79	3.90	1.96	15.82	1.055	Up
88.0758	2-amino-2-methyl-1,3-propanediol	Organic nitrogen compounds	4.453±0.157	13.86±0.83	3.69	1.88	0.05	1.059	Up
104.1071	Choline	Organic nitrogen compounds	4586±364	1887±102	0.49	-1.04	12.32	1.057	Down
172.0492	Crimidine	Organic nitrogen compounds	106.5±6.3	37.38±3.87	0.42	-1.26	653.92	1.053	Down
262.1288	Methapyrilene	Organic nitrogen	38.11±1.16	4.501±0.053	0.14	-2.84	0.09	1.061	Down

		compounds							
184.0733	Miltefosine	Organic nitrogen compounds	143.1±1.7	21.03±1.32	0.17	-2.53	0.18	1.061	Down
206.1653	N1-(1-methyl-4-piperidinyl)-1,4-benzene diamine	Organic nitrogen compounds	ND	0.01949±0.00181	6.56	2.71	7.57	1.061	Up
516.3030	Oleyloxyethylphosphorylcholine	Organic nitrogen compounds	ND	0.3899±0.0061	31.74	4.99	0.09	1.061	Up
318.3004	Phytosphingosine	Organic nitrogen compounds	11.90±0.35	38.59±2.48	3.85	1.94	16.36	1.059	Up
282.2791	Sphingosine	Organic nitrogen compounds	1.132±0.099	2.068±0.019	2.17	1.12	101.70	1.052	Up
290.2076	1-heptanone, 1-(4-methoxyphenyl)-2-(1-pyrrolidinyl)-	Organic oxygen compounds	1.449±0.049	6.327±0.138	5.18	2.37	2.01	1.060	Up
99.0918	2-hexenal	Organic oxygen compounds	2.544±0.033	5.341±0.242	2.48	1.31	17.84	1.058	Up
151.0519	3,4-dihydroxyacetophenone	Organic oxygen compounds	ND	160.7±6.5	466.67	8.87	0.02	1.061	Up
617.1323	3.alpha.,4.beta.-galactotriose	Organic oxygen compounds	0.5642±0.0388	0.2314±0.0286	0.49	-1.04	1304.10	1.044	Down
535.1518	3-deoxy-d-glycero-d-galacto-2-nonulosonic acid	Organic oxygen compounds	6.737±0.596	1.659±0.025	0.29	-1.77	11.69	1.059	Down
489.1695	5-azacytidine	Organic oxygen compounds	6.862±0.231	16.31±1.12	2.81	1.49	0.50	1.057	Up
347.0952	Acetohexamide	Organic oxygen compounds	2.665±0.206	6.518±0.539	2.89	1.53	153.67	1.050	Up
385.1313	Catalpol	Organic oxygen compounds	0.1245±0.0096	0.4332±0.0076	4.13	2.05	4.72	1.059	Up
422.1062	Cyclosulfamuron	Organic oxygen	2.042±0.029	3.507±0.206	2.04	1.03	29.85	1.052	Up

		compounds							
180.0688	D-mannosamine	Organic oxygen compounds	0.4765±0.0280	6.731±0.144	16.75	4.07	0.06	1.061	Up
259.0130	D-mannose 6-phosphate	Organic oxygen compounds	65.47±2.22	24.11±2.22	0.44	-1.20	42.54	1.056	Down
229.0354	D-ribose 1-phosphate	Organic oxygen compounds	27.59±2.41	6.629±0.236	0.28	-1.81	37.18	1.058	Down
289.0331	D-Ribose 5-phosphate	Organic oxygen compounds	13.66±0.85	35.65±0.49	3.10	1.63	6.17	1.059	Up
339.0042	Fructose 1,6-diphosphate	Organic oxygen compounds	0.3248±0.0053	0.7763±0.0506	2.83	1.50	89.02	1.058	Up
647.1854	Laminaritetraose	Organic oxygen compounds	4.410±0.169	1.720±0.062	0.46	-1.11	24.84	1.060	Down
637.1533	Leiocarposide	Organic oxygen compounds	0.09020±0.00712	0.2107±0.0158	2.76	1.47	88.13	1.051	Up
447.1588	N,n'-diacetylchitobiose	Organic oxygen compounds	0.9065±0.0434	20.01±1.22	26.11	4.71	0.03	1.061	Up
465.1696	N-acetylglucosamine	Organic oxygen compounds	0.4625±0.0143	5.690±0.285	14.56	3.86	0.20	1.061	Up
251.0776	Orcinol .beta.-d-glucoside	Organic oxygen compounds	24.30±2.37	7.751±0.046	0.38	-1.40	25.26	1.058	Down
499.1645	Primeverin	Organic oxygen compounds	0.1147±0.0064	0.5588±0.0258	5.79	2.53	6.64	1.060	Up
318.1164	Prunasin	Organic oxygen compounds	0.07341±0.00245	0.5849±0.0612	9.43	3.24	10.35	1.059	Up
195.1227	Tetraethylene glycol	Organic oxygen compounds	65.47±5.85	17.63±1.22	0.32	-1.65	7.66	1.058	Down
151.0965	Triethylene glycol monobutyl ether	Organic oxygen	5.721±0.159	2.214±0.013	0.46	-1.12	12.81	1.061	Down

		compounds							
189.1236	.alpha.-ethyltryptamine	Organoheterocyclic compounds	5.798±0.238	58.76±1.34	12.02	3.59	0.29	1.061	Up
204.0689	1-(2,8-dihydroxyquinolin-5-yl)ethan-1-one	Organoheterocyclic compounds	29.36±0.75	6.950±0.461	0.28	-1.83	6.90	1.060	Down
101.1074	1-methylpiperazine	Organoheterocyclic compounds	44.71±3.82	15.57±1.31	0.41	-1.28	32.06	1.056	Down
363.0928	1-methyluric acid	Organoheterocyclic compounds	0.2318±0.0155	0.9028±0.0499	4.62	2.21	19.70	1.058	Up
372.1897	1-pentyl-3-(4-methoxynaphthoyl)indole	Organoheterocyclic compounds	0.1980±0.0081	0.8308±0.0790	4.97	2.31	24.66	1.057	Up
217.1046	2-(2',3',4'-trihydroxybutyl)quinoxaline	Organoheterocyclic compounds	29.76±0.62	3.934±0.141	0.16	-2.68	0.04	1.061	Down
557.2573	4-hydroxyatorvastatin lactone	Organoheterocyclic compounds	31.03±1.89	7.769±0.542	0.30	-1.75	16.66	1.059	Down
144.0302	4-hydroxyquinoline	Organoheterocyclic compounds	35.56±1.21	12.92±0.38	0.43	-1.22	5.19	1.060	Down
208.0619	4-morpholinopropanesulfonic acid	Organoheterocyclic compounds	0.3112±0.0026	1.365±0.075	5.21	2.38	7.34	1.060	Up
129.1024	4-piperidinecarboxamide	Organoheterocyclic compounds	1.070±0.015	2.418±0.174	2.68	1.42	19.43	1.056	Up
213.0172	8-chlorotheophylline	Organoheterocyclic compounds	33.76±0.71	6.149±0.719	0.22	-2.21	22.92	1.058	Down
190.0476	8-hydroxyquinoline-5-carboxylic acid	Organoheterocyclic compounds	1.709±0.089	3.120±0.103	2.16	1.11	5.49	1.056	Up
266.1114	Albendazole	Organoheterocyclic compounds	0.07488±0.00235	0.3207±0.0183	5.08	2.34	9.53	1.060	Up
298.1013	Albendazole sulfone	Organoheterocyclic	1.179±0.043	3.387±0.115	3.41	1.77	0.78	1.060	Up

		compounds							
228.1343	Ametryne	Organoheterocyclic compounds	7.977±0.209	2.372±0.055	0.35	-1.50	7.49	1.061	Down
129.0408	Ammelide	Organoheterocyclic compounds	ND	2.959±0.124	15.50	3.95	0.10	1.061	Up
219.0916	Benzamide, n-1h-indol-5-yl-	Organoheterocyclic compounds	1.714±0.119	3.535±0.152	2.45	1.29	8.12	1.056	Up
567.1683	Benzeneacetamide, 4-(4,9-diethoxy-1,3-dihydro-1-oxo-2h-benz[f]isoindol-2-yl)-n-(phenylsulfonyl)-	Organoheterocyclic compounds	0.1589±0.0153	0.3324±0.0406	2.47	1.31	189.50	1.037	Up
326.1346	Bromosporine	Organoheterocyclic compounds	0.3118±0.0020	0.6642±0.0253	2.52	1.33	39.59	1.060	Up
195.0877	Caffeine	Organoheterocyclic compounds	28.88±1.48	9.083±0.516	0.37	-1.42	12.90	1.059	Down
217.0973	Carboline base + 4h, carboxylic acid	Organoheterocyclic compounds	0.5937±0.0057	6.827±0.526	13.61	3.77	0.95	1.060	Up
112.0506	Cytosine	Organoheterocyclic compounds	159.3±1.9	374.8±34.5	2.79	1.48	17.51	1.053	Up
358.1741	Danofloxacin	Organoheterocyclic compounds	0.4123±0.0365	2.067±0.123	5.93	2.57	24.31	1.058	Up
382.1432	Enrofloxacin	Organoheterocyclic compounds	0.8103±0.0288	3.081±0.104	4.50	2.17	0.42	1.061	Up
399.0093	Ethiprole	Organoheterocyclic compounds	2.367±0.072	0.3561±0.0076	0.18	-2.49	1.25	1.061	Down
236.0587	Ethyl 8-fluoro-4-hydroxyquinoline-3-carboxylate	Organoheterocyclic compounds	0.1845±0.0032	1.847±0.075	11.87	3.57	0.62	1.061	Up
303.1054	Hematoxylin	Organoheterocyclic	8.928±0.746	3.178±0.095	0.42	-1.24	13.72	1.058	Down

		compounds							
312.1538	Imazaquin	Organoheterocyclic compounds	0.9720±0.0260	2.955±0.236	3.60	1.85	8.87	1.058	Up
427.2302	Lovatatin	Organoheterocyclic compounds	264.0±5.4	28.25±1.90	0.13	-2.98	3.56	1.060	Down
350.2691	Methanone, (1-pentyl-1h-indol-3-yl)tricyclo[3.3.1.13, 7]dec-1-yl-	Organoheterocyclic compounds	0.4134±0.0140	1.515±0.083	4.35	2.12	17.14	1.060	Up
388.1847	Methanone, [1-(5-hydroxypentyl)-1h-indol-3-yl](4-methoxy-1-naphthalenyl)-	Organoheterocyclic compounds	0.7107±0.0560	2.225±0.160	3.72	1.89	26.30	1.057	Up
435.1446	Methotrexate	Organoheterocyclic compounds	0.4918±0.0254	0.9521±0.0962	2.30	1.20	468.90	1.041	Up
234.1338	Metolachlor-morpholinone	Organoheterocyclic compounds	3.035±0.056	5.414±0.382	2.11	1.08	62.59	1.051	Up
452.2485	NCGC00381123-01	Organoheterocyclic compounds	0.4094±0.0303	2.619±0.212	7.56	2.92	9.60	1.058	Up
326.1236	N-desmethyldanofloxacin	Organoheterocyclic compounds	2.233±0.148	0.4858±0.0108	0.26	-1.95	4.87	1.060	Down
153.0660	Nudifloramide	Organoheterocyclic compounds	0.3175±0.0224	1.337±0.088	4.99	2.32	12.32	1.059	Up
181.0720	Paraxanthine	Organoheterocyclic compounds	39.01±2.55	9.773±0.529	0.30	-1.75	11.46	1.059	Down
378.1163	Pyridine, 4-[4-(4-fluorophenyl)-2-[4-(methylsulfinyl)phenyl]-1h-imidazol-5-yl]-	Organoheterocyclic compounds	0.5544±0.0260	1.111±0.064	2.38	1.25	89.40	1.055	Up
168.0656	Pyridoxal	Organoheterocyclic compounds	30.00±0.74	12.30±1.00	0.48	-1.05	196.32	1.055	Down

170.0812	Pyridoxine	Organoheterocyclic compounds	461.3±23.7	185.7±18.4	0.48	-1.07	389.94	1.053	Down
144.0667	Quinolin-2-ol	Organoheterocyclic compounds	ND	1.769±0.133	11.47	3.52	1.47	1.061	Up
131.0454	Quinoxaline	Organoheterocyclic compounds	8.722±0.827	3.400±0.101	0.46	-1.11	45.22	1.056	Down
288.1056	Rutaecarpine	Organoheterocyclic compounds	1.853±0.025	28.36±1.61	18.13	4.18	0.46	1.061	Up
176.0919	Tenofovir	Organoheterocyclic compounds	37.23±2.25	350.9±37.6	11.18	3.48	3.16	1.059	Up
181.0530	Theophylline	Organoheterocyclic compounds	51.26±2.09	10.97±0.09	0.25	-1.98	0.49	1.061	Down
209.0091	Trimellitic acid anhydride	Organoheterocyclic compounds	18.94±1.49	5.431±0.064	0.34	-1.55	3.97	1.060	Down
111.0200	Uracil	Organoheterocyclic compounds	185.4±16.1	66.30±6.05	0.42	-1.24	242.09	1.053	Down
264.1266	Zaleplon	Organoheterocyclic compounds	4.125±0.108	7.388±0.369	2.12	1.09	39.98	1.055	Up
235.1190	Zolpidem	Organoheterocyclic compounds	7.011±0.342	25.95±1.62	4.38	2.13	0.08	1.059	Up
217.0973	4,4'-thiodianiline	Organosulfur compounds	3.238±0.091	22.37±1.36	8.18	3.03	0.01	1.060	Up
415.2562	Propanoic acid, 2-[[4-[2-[[(cyclohexylamino)carbonyl](4-cyclohexylbutyl)amino]ethyl]phenyl]thio]-2-methyl-	Organosulfur compounds	0.7379±0.0110	2.837±0.141	4.55	2.19	3.21	1.060	Up
291.1053	(+)-catechin	Phenylpropanoids and polyketides	21.55±0.94	55.57±1.08	3.06	1.61	12.36	1.059	Up
485.2356	(2r,3r,4s,5s,6r)-2-[1,7-bis(4-hydroxyphen	Phenylpropanoids and	19.66±0.50	2.095±0.189	0.13	-2.98	4.54	1.060	Down

	yl)heptan-3-yloxy]-6-(hydroxymethyl)oxane-3,4,5-triol	polyketides							
377.0757	3',4',5,7-tetrahydroxy-3,6,8-trimethoxyflavone	Phenylpropanoids and polyketides	0.6035±0.0505	2.220±0.264	4.36	2.12	142.77	1.054	Up
303.0689	3,7,3',4',5'-pentahydroxyflavone	Phenylpropanoids and polyketides	1.090±0.105	2.075±0.047	2.25	1.17	543.48	1.049	Up
313.0911	3,7,3'-trimethoxyflavone	Phenylpropanoids and polyketides	5.687±0.199	2.197±0.028	0.46	-1.13	12.23	1.060	Down
239.0776	7-hydroxyflavone	Phenylpropanoids and polyketides	6.258±0.559	12.15±0.41	2.30	1.20	335.58	1.051	Up
342.1071	Benzoic acid, 2-[[(2z)-3-(3,4-dimethoxyphenyl)-1-oxo-2-propen-1-yl]amino]-3-hydroxy-	Phenylpropanoids and polyketides	0.7839±0.0502	1.345±0.125	2.03	1.02	1504.90	1.040	Up
221.0956	Benzyl cinnamate	Phenylpropanoids and polyketides	0.1980±0.0077	0.9531±0.0403	5.70	2.51	10.11	1.060	Up
358.0909	Casticin	Phenylpropanoids and polyketides	0.08054±0.00498	0.2511±0.0043	3.70	1.89	12.35	1.060	Up
530.2593	Epothilone b	Phenylpropanoids and polyketides	ND	1.395±0.087	13.65	3.77	1.31	1.061	Up
285.0696	Maackiaine	Phenylpropanoids and polyketides	2.729±0.158	5.676±0.193	2.47	1.30	14.37	1.057	Up
449.0936	Marein	Phenylpropanoids and polyketides	0.2838±0.0091	2.385±0.014	9.97	3.32	0.44	1.061	Up
389.1202	Polydatin	Phenylpropanoids and polyketides	1.302±0.032	0.5385±0.0649	0.49	-1.03	779.59	1.048	Down
395.1663	Rotenone	Phenylpropanoids and polyketides	2.952±0.128	60.05±3.63	24.17	4.60	0.83	1.061	Up
681.1296	Rutarensin	Phenylpropanoids and	ND	0.4337±0.0213	32.82	5.04	0.26	1.061	Up

		polyketides							
463.1327	Tectoridin	Phenylpropanoids and polyketides	0.8737±0.0223	13.56±0.33	18.40	4.20	0.10	1.061	Up
